# AI-assisted evidence screening method for systematic reviews in environmental research: integrating ChatGPT with domain knowledge

**DOI:** 10.1186/s13750-025-00358-5

**Published:** 2025-04-15

**Authors:** Chen Zuo, Xiaohao Yang, Josh Errickson, Jiayang Li, Yi Hong, Runzi Wang

**Affiliations:** 1 School for Environment and Sustainability, University of Michigan, Ann Arbor, USA; 2 School of Information, University of Michigan, Ann Arbor, USA; 3 Consulting for Statistics Computing & Analytics Research, University of Michigan, Ann Arbor, USA; 4 Department of Landscape Architecture, College of Design, Construction and Planning, University of Florida, Gainesville, USA; 5 Cooperative Institute for Great Lakes Research, University of Michigan, Ann Arbor, USA; 6 Department of Human Ecology, College of Agricultural and Environmental Sciences, University of California, Davis, USA

**Keywords:** Literature review, Literature screening, Eligibility criteria, Interdisciplinary study, Large Language model, Fine tuning

## Abstract

Systematic reviews (SRs) in environmental science is challenging due to diverse methodologies, terminologies, and study designs across disciplines. A major limitation is that inconsistent application of eligibility criteria in evidence-screening affects the reproducibility and transparency of SRs. To explore the potential role of Artificial Intelligence (AI) in applying eligibility criteria, we developed and evaluated an AI-assisted evidence-screening framework using a case study SR on the relationship between stream fecal coliform concentrations and land use and land cover (LULC). The SR incorporates publications from hydrology, ecology, public health, landscape, and urban planning, reflecting the interdisciplinary nature of environmental research. We fine-tuned ChatGPT-3.5 Turbo model with expert-reviewed training data for title, abstract, and full-text screening of 120 articles. The AI model demonstrated substantial agreement at title/abstract review and moderate agreement at full-text review with expert reviewers and maintained internal consistency, suggesting its potential for structured screening assistance. The findings provide a structured framework for applying eligibility criteria consistently, improving evidence screening efficiency, reducing labor and costs, and informing large language models (LLMs) integration in environmental SRs. Combining AI with domain knowledge provides an exploratory step to evaluate feasibility of AI-assisted evidence screening, especially for diverse, large volume, and interdisciplinary studies. Additionally, AI-assisted screening has the potential to provide a structured approach for managing disagreement among researchers with diverse domain knowledge, though further validation is needed.

**Supplementary Information:**

The online version contains supplementary material available at 10.1186/s13750-025-00358-5.

## Introduction

Environmental science research employs diverse methodologies to investigate interactions between human-nature systems. It encompasses various study designs, data types, analytical methods, spatiotemporal scales, and research contexts [1–3]. The field requires interdisciplinary collaboration among experts in ecology, hydrology, biology, engineering, landscape, urban planning, and social science [4]. Researchers across disciplines apply distinct methodologies and terminologies for similar research questions [4, 5], complicating synthesis. The interdisciplinary nature of environmental science presents challenges in establishing consistent eligibility criteria and synthesize evidence in systematic reviews (SRs) compared to experimental fields such as clinical medicine [6].

Evidence screening, the process of identifying relevant studies for inclusion, is fundamental to the rigor, transparency, and reproducibility of SRs [7–9]. The first step in evidence screening involves defining eligibility criteria [10]. According to Cochrane Guidelines, eligibility criteria should be applied by multiple reviewers working independently and in duplicate to ensure consistency [7]. Discrepancies should be resolved through arbitration and consensus exercises at each stage to achieve agreement among reviewers [7, 11]. This process is also essential for SRs in environmental science that aim to provide evidence on environmental research questions through comprehensive, rigorous, transparent, and reproducible synthesis reviews of existing studies [7, 12, 13].

However, the interdisciplinary nature of environmental science complicates the evidence-screening process [14, 15]. Variability in study designs and analytical methods challenges in defining consistent eligibility criteria [16]. Further, reviewers from different disciplines may interpret the same eligibility criteria differently, causing inconsistent and unreliable evidence screening outcomes [17, 18]. Traditionally, SRs in environmental science rely on manual screening, applying predefined criteria for consistency. However, manual screening is not only time-consuming and labor-intensive but also prone to human error [16], especially when an SR involves a large volume of diverse and context-specific studies.

Artificial Intelligence (AI) offers potential to streamline SRs’ processes, particularly in automating evidence screening through machine learning and natural language processing [19–21]. While many AI tools can classify evidence, summarize texts, and assist in screening, they are often limited in specialized domain knowledge [19, 22, 23]. Recent advances in Large Language Models (LLMs) enhance contextual understanding, and fine-tuning with domain knowledge improves their performance to specific research needs [24–26]. Therefore, integrating LLMs with domain expertise has potential to provide a structural and efficient the screening process.

Our case study SR investigates the influence of land use and land cover (LULC) on stream fecal coliform contamination, providing insights into the applicability of AI-assisted screening in environmental research. Specifically, we aim to quantify the relationship between different types of LULC and the levels of fecal coliform in streams, as well as to explore methodological considerations for AI-assisted evidence screening. The study reflects key challenges in environmental science SRs, including interdisciplinary differences in terminology, methods, and data interpretation across hydrology, public health, landscape, and urban planning. The variations in spatiotemporal scales lead to different research methodologies (e.g., statistical or mechanistic models) to study fecal coliform contamination. Interdisciplinary in natural and soci-economic conditions contributes to variation in research findings, sometimes leading to contradictory conclusions. Therefore, this SR provides an opportunity to assess AI-assisted literature screening in environmental research and its potential in addressing inconsistencies in eligibility criteria application in SRs. To assess AI-assisted evidence screening, we fine-tuned ChatGPT-3.5 Turbo model with the expertise of environmental researchers to explore two questions:

1. How does ChatGPT-3.5 Turbo perform in evidence screening?
2. How does the consistency of ChatGPT-3.5 Turbo in evidence screening compare to that of human reviewers?

## Method

The research team comprised six members: three domain expert reviewers responsible for literature screening and three technical specialists supporting model development and analysis. The three expert reviewers (‘R1’, ‘R2’, and ‘R3’) led the research by utilizing domain knowledge to define eligibility criteria, independently screen sample articles based on titles and abstracts, and full-text. R1 is a Ph.D. student in environmental science. R2 and R3 are environmental scientists with expertise in land use and hydrology, respectively. The technology supporters included an expert with prior SR experience, a Ph.D. student in data science, and a statistician. They were responsible for formulating the ChatGPT fine-tuning process, applying the model for evidence selection, and conducting statistical analysis of the results.

We built on the ChatGPT-3.5 Turbo and fine-tuned it using screening outcomes from the expert reviewers to enhance its ability to assess studies. Zotero (version 6.0.36) and Excel were used for article management, while RStudio (version 4.1.2) was utilized for statistical analysis. The study adhered to the PRISMA 2020 protocols [11] and Cochrane Handbook guidelines [7].

### Search strategy

We conducted our article search using the Scopus, Web of Science, ProQuest, and PubMed databases. The search queries incorporated a combination of keywords, including “land use” (and synonyms), “fecal coliform” (and spelling variants, and the subset of fecal coliform bacteria), and “stream” (and synonyms) (Table 1). These keywords were combined using “AND” to create search queries for each database (details in Appendix Table A1).

**Table 1:** The search keywords and boolean operators

Serial Number	Keywords
1	**“land use”** OR “land cover” OR landuse OR landcover
2	**fecal** OR faecal OR coliform* OR “E. coli” OR “escherichia coli”
3	**stream** OR streamlet OR river OR riverine OR rivulet OR creek OR brook OR watercourse OR waterway OR tributary OR branch OR flow

### Workflow

**Fig. 1:**
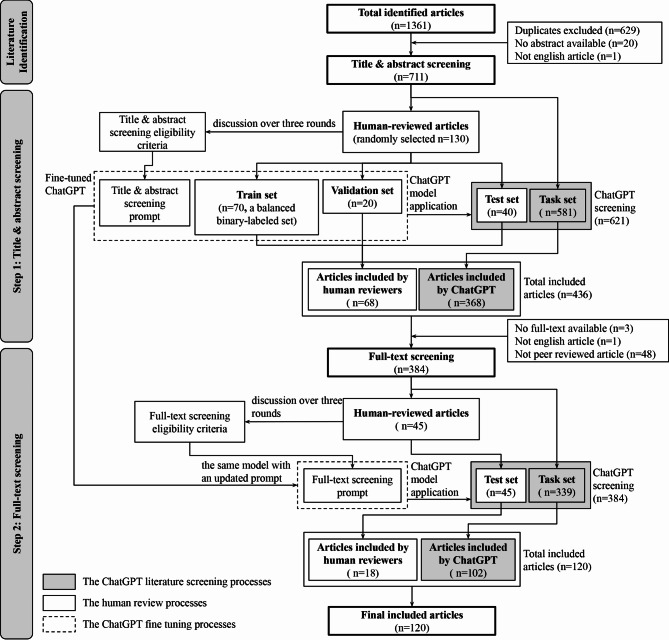
Method workflow

The workflow comprised three main stages: a pre-screening stage (Literature identification), followed by a two-step screening process — Step 1 (Title and abstract screening), and Step 2 (Full-text screening) (see Fig. 1). Initially, on March 19, 2024, a total of 1,361 articles were searched. After removing duplicated and non-English articles and those without abstracts, 711 articles were identified (see Appendix Table A2) to enter the screening process. In Step 1, reviewers randomly selected 130 articles (using the “sample_n()” function from the “dplyr” package in R) and determined “include” or “exclude” through group discussion. This process, repeated over four rounds, helped establish eligibility criteria. The criteria were then translated into a ChatGPT prompt as domain knowledge. Articles reviewed by human reviewers were used as a training dataset for fine-tuning ChatGPT-3.5 Turbo, which then screened the remaining articles. Step 2 followed a similar process but without model fine-tuning.

### Literature screening

In Step 1 (title & abstract screening), three reviewers independently assessed 130 randomly selected articles to evaluate relevance, resolve discrepancies, and refine eligibility criteria. After four rounds of iterative discussion, reviewers established consensus-based final versions of the eligibility criteria (Table 2; the criteria’s four versions in Appendix Table A3) and created a binary-labeled dataset (i.e., “Yes” or “No” for relevance) for article inclusion (see Appendix Table A4).

**Table 2:** The eligibility criteria of step 1 title & abstract screening

Criteria Number	Criteria Information
Criteria 1.1	The title or abstract should contain either the term ‘land use’ or the term ‘land cover’. This requirement can also be satisfied if the title or abstract mentions more than one type of land use or land cover.
Criteria 1.2	The research method or results should contain either the term ‘land use’, or the term ‘land cover’. This requirement can also be satisfied if the research method or results mention more than one type of land use or land cover.
Criteria 1.3	The research method or results should contain exactly one of the terms in the Fecal Coliform Contamination List.
Criteria 1.4	The research method or results should contain the direct relationship between land use/land cover or the types of land use/land cover and Fecal coliform or one of the terms in the Fecal Coliform Contamination List.

Note of Table 1: the criteria in Step 1 are based solely on the title and abstract, not a full-text review

Subsequently, ChatGPT-3.5 Turbo was fine-tuned for our research question. The final versions of the eligibility criteria were translated into a prompt (see Appendix A), and the binary-labeled set of 130 articles was split into a 70-article training set (randomly selecting 35 “Yes” and 35 “No” articles), a 20-article validation set, and a 40-article test set (see Appendix Table A4).

We applied a light fine-tuning process to the existing ChatGPT-3.5 Turbo model, adjusting key hyperparameters (specifically, epochs, batch size, learning rate, temperature, and top_p) to optimize performance on a specific dataset [27]. Epochs determine the number of passes through the data, where too few can lead to underfitting and too many to overfitting [28]. Batch size controls how many examples are processed before updates, with larger sizes speeding up training but requiring more memory, while smaller sizes add randomness and help escape local minima [29]. Learning rate dictates the step size for weight updates, where a high rate may lead to suboptimal solutions or divergence, and a low rate can slow down training [30]. Temperature controls the randomness of the model’s response [31]. Top_p sampling selects tokens based on cumulative probability, with lower values producing more focused outputs and higher values increasing variability [32].

After fine-tuning, we accounted for the model’s stochastic nature [33] by performing 15 runs and using the majority result as the final output (i.e., if more than 8 runs resulted in “Yes,” the answer was “Yes”; otherwise, it was “No”). The fine-tuned model was then used to screen the 581-article task set (see Appendix Table A5) with a temperature setting of 0.4 and top_p setting 0.8. We evaluated the model’s performance using Cohen’s Kappa and Fleiss’s Kappa statistics on a 40-article test set (see Appendix TableA6). Cohen’s Kappa measures agreement between two raters [34], while Fleiss’s Kappa extends this to multiple raters [35].

In Step 2, 384 articles that passed screening in Step 1 and had full-text availability underwent full-text screening (see Appendix Table A5 and A6). Because Step 2 involves full-text screening, focusing on the results and discussion sections for more comprehensive information, we updated our criteria prompt accordingly (see Appendix B). Three reviewers independently evaluated 45 randomly selected articles for inclusion based on the updated eligibility criteria through three rounds of an interactive process (Table 3; the criteria’s three versions in Appendix Table A7). This process resulted in a binary-labeled dataset (see Appendix Table A8), serving as the test set for the full-text screening model, which employs the same fine-tuned ChatGPT model, temperature and top_p setting as in Step 1 but with the updated criteria prompt. The refined model was then utilized to screen the remaining 339 articles (see Appendix Table A5). Finally, 120 articles passed screening in Step 2 (see Appendix Table A9 and A10). We also evaluated the model’s performance on a 45-article test set (see Appendix Table A10).

**Table 3:** The eligibility criteria of step 2 Full-text screening

Criteria Number	Criteria Information
Criteria 2.1	The research results should contain either the term ‘land use’ or the term ‘land cover’. This requirement can also be satisfied if the research results mention more than one type of land use or land cover.
Criteria 2.2	The research results should contain exactly one of the terms in the Fecal Coliform Contamination List.
Criteria 2.3	The research results should contain the statistical relationship between land use/land cover or the types of land use/land cover and Fecal coliform or one of the terms in the Fecal Coliform Contamination List.

### ROI analysis

We analyzed the Return on Investment (ROI) of AI-assisted versus manual screening by comparing costs and time savings. ROI was assessed by comparing costs and time for manually reviewing versus using ChatGPT for task set articles in Steps 1 and 2, respectively. In manual screening, reviewers screened each article in ~ 3 min (Step 1) and ~ 5 min (Step 2), with two 1-hour discussions per step to resolve disagreements (2 h total per step). The AI-assisted method used ChatGPT, with a reviewer supervising results and refining prompts, and a computer science expert fine-tuning the model. We tracked ChatGPT’s token usage, subscription fees, and labor costs.

## Results

### ChatGPT-3.5 turbo fine-tuning

We selected a small batch size of 2 to update the model at a low frequency and a learning rate of 0.2 to minimize overfitting due to the limited dataset size in the fine-tuning process. The model was trained for 3 epochs indicating the dataset was processed in three full cycles. While there are no standard configurations for GPT fine-tuning, we selected these hyperparameters to balance domain knowledge learning and model generalization.

**Fig. 2:**
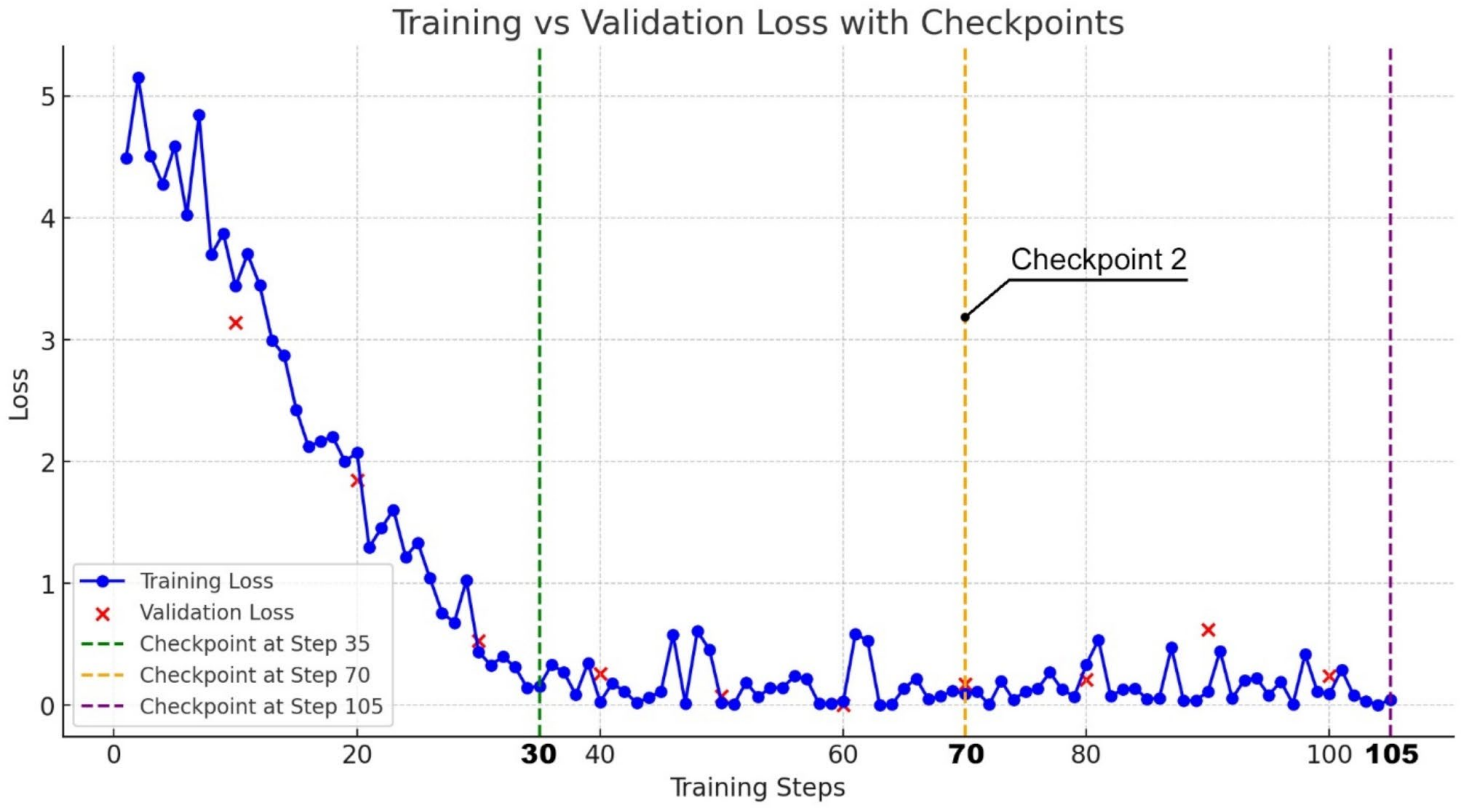
Training vs. validation loss curve with checkpoints

During the finetuning process (Fig. 2), the training loss (blue line) showed a consistent decline, which indicated effective learning. The validation loss (red ‘X’ marks) initially dropped but became unstable later at around step 90 suggesting potential overfitting. We saved model checkpoints at Steps 35, 70, and 105 (green, orange, and purple dashed lines), and selected Checkpoint 2 at Step 70 (orange line) for the SR task due to its balance of training and validation performance. In summary, our fine-tuning approach balanced consistency in the training progress and generalization, with Checkpoint 2 providing optimal performance for the SR task.

### Evaluation of ChatGPT-3.5 Turbo’s agreement with human reviewers and internal consistency

The fine-tuned ChatGPT-3.5 Turbo’s predictions aligned significantly with human reviewers’ consensus conclusion, demonstrating substantial agreement (Cohen’s Kappa score is 0.79) in Step 1 and moderate agreement (Cohen’s Kappa score is 0.61) in Step 2 (Tables 4 and 5).

**Table 4:** ChatGPT-3.5 Turbo’s prediction performance of steps 1 and 2

Screening step	Cohen’s Kappa Score	*P*-value	Balanced Accuracy	95% CI
Step 1 Title & abstract screening	0.79	< 0.05	0.89	0.79 ~ 0.99
Step 2 Full-text screening	0.61	< 0.05	0.82	0.67 ~ 0.91

**Table 5:** The confusion matrix of steps 1 and 2 between ChatGPT-3.5 Turbo prediction and reviewers’ consensus

	Reference (Reviewers’ consensus conclusion)				
**Prediction** (GPT3.5’s majority answer)		Yes	No		
	Yes	**22 | 11 (TP)**	*3 | 1 (FP)*	25 | 12	Positive Predictive Value: 88% | 91.7%
	No	*1 | 7 (FN)*	**14 | 26 (TN)**	15 | 33	Negative Predictive Value: 93.3% | 78.8%
		23 | 18	17 | 27		
		Sensitivity: 95.7% | 61.1%	Specificity: 82.4% | 96.3%		Accuracy: 90.0% | 82.2%

Note of Table 4: Values in each cell are represented as ‘Step 1 value | Step 2 value’; ‘TP’ is the number of true positive articles; ‘FP’ is the number of false positive articles; ‘TN’ is the number of true negative articles; ‘FN’ is the number of false negative articles; ‘Positive Predictive Value’ is $\:\frac{TP}{TP+FP}$; ‘Negative Predictive Value’ is $\:\frac{TN}{TN+FN}$; ‘Sensitivity’ is $\:\frac{TP}{TP+FN}$; ‘Specificity’ is $\:\frac{TN}{TN+FP}$; ‘Accuracy’ is $\:\frac{TP+TN}{TP+TN+FP+FN}$

**Fig. 3:**
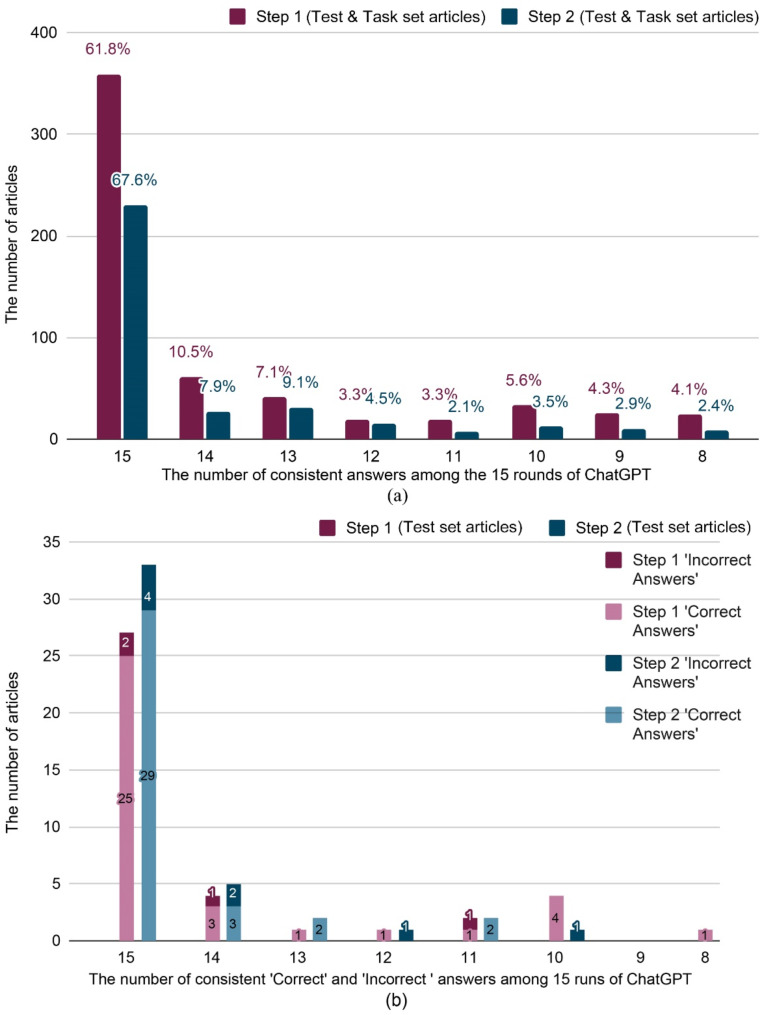
The consistency of ChatGPT-3.5 Turbo answers. (**a**) The percentage of consistent answers across 15 runs. (**b**) The number of correct and incorrect answers across 15 runs in the test set

The model also demonstrated substantial internal consistency across the 15 runs, with Fleiss’s Kappa of 0.81 and 0.78 in Steps 1 and 2, respectively. Over 90% of articles received consistent answers in at least 10 out of 15 runs for both steps (Fig. 3a, the run results of screening articles in Steps 1 and 2 are in Appendix Table A5 and A9). In the test set, over 75% of articles were consistently correctly predicted in at least 10 runs (Fig. 3b, the run results of test set in Steps 1 and 2 are in Appendix Table A6 and A10).

**Fig. 4:**
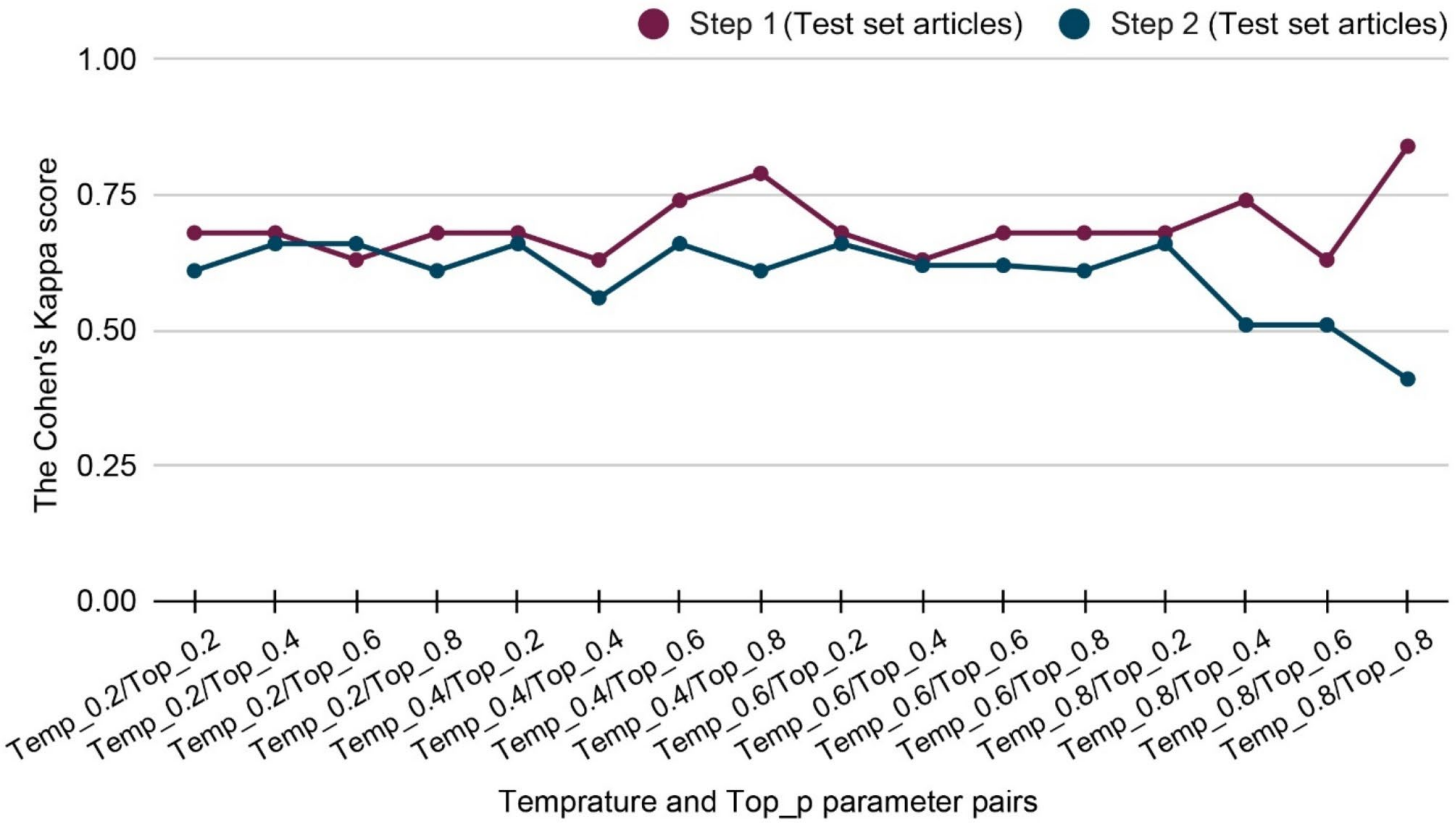
ChatGPT-3.5 Turbo performance across temperature and top_p parameter settings in Steps 1 and 2

We tested 16 parameter pairs by varying temperature and top_p (0.2–0.8 in 0.2 increments) using the test dataset from Steps 1 and 2. Most combinations produced high and consistency kappa values with minimal performance variations (Fig. 4, see Appendix Table A11 and A12). ChatGPT demonstrated stable performance across a wide parameter range, suggesting strong internal consistency.

### Comparison of ChatGPT-3.5 turbo and human reviewers in evidence screening: variability and agreement

**Fig. 5:**
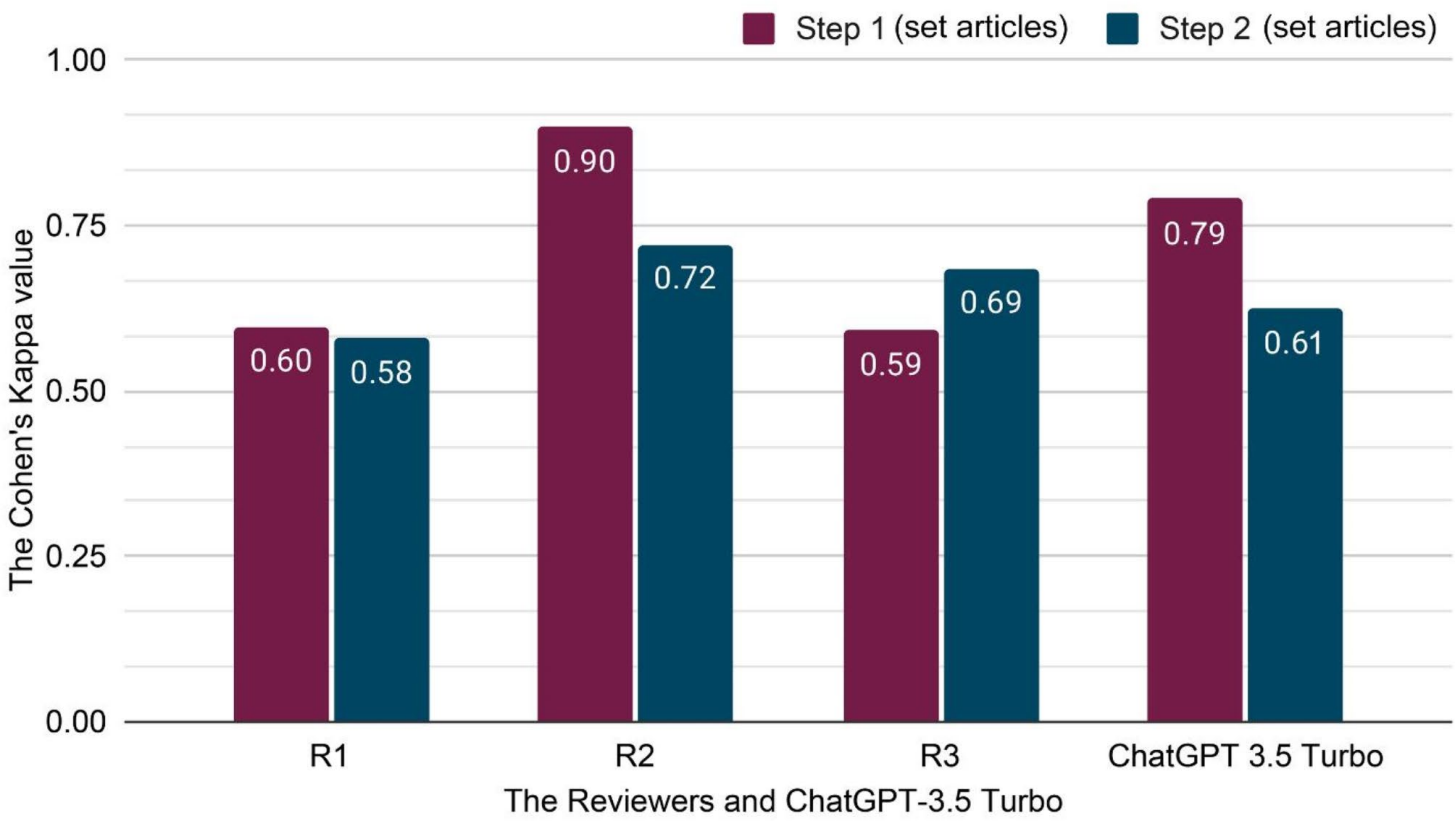
The reviewer and ChatGPT-3.5 Turbo performance in Steps 1 and 2

Overall, ChatGPT-3.5 Turbo’s performance remained comparable to that of the human reviewers in both steps. We evaluated the agreement between both individual reviewers and ChatGPT-3.5 Turbo’s responses against the consensus conclusions by using test set in Steps 1 and 2 (Fig. 5, see Appendix Table A6 and A10). Human reviewers’ performance demonstrated significant variability across individual reviewers and the two steps. Specifically, in Step 1, Reviewer ‘R2’ performed better (Cohen’s Kappa score = 0.90) than ‘R1’, ‘R3’, and ChatGPT, where, ChatGPT’s Kappa score was 0.79 and ‘R1’ and ‘R3’’s Kappa were under 0.60 and 0.59. In Step 2, however, ‘R2’s’ Kappa dropped to 0.72, though it still exceeded the performance of the other reviewers and ChatGPT. During this stage, ChatGPT’s Kappa was at 0.61, closely matching ‘R1’ (0.58) and against ‘R3’ (0.69).

**Fig. 6:**
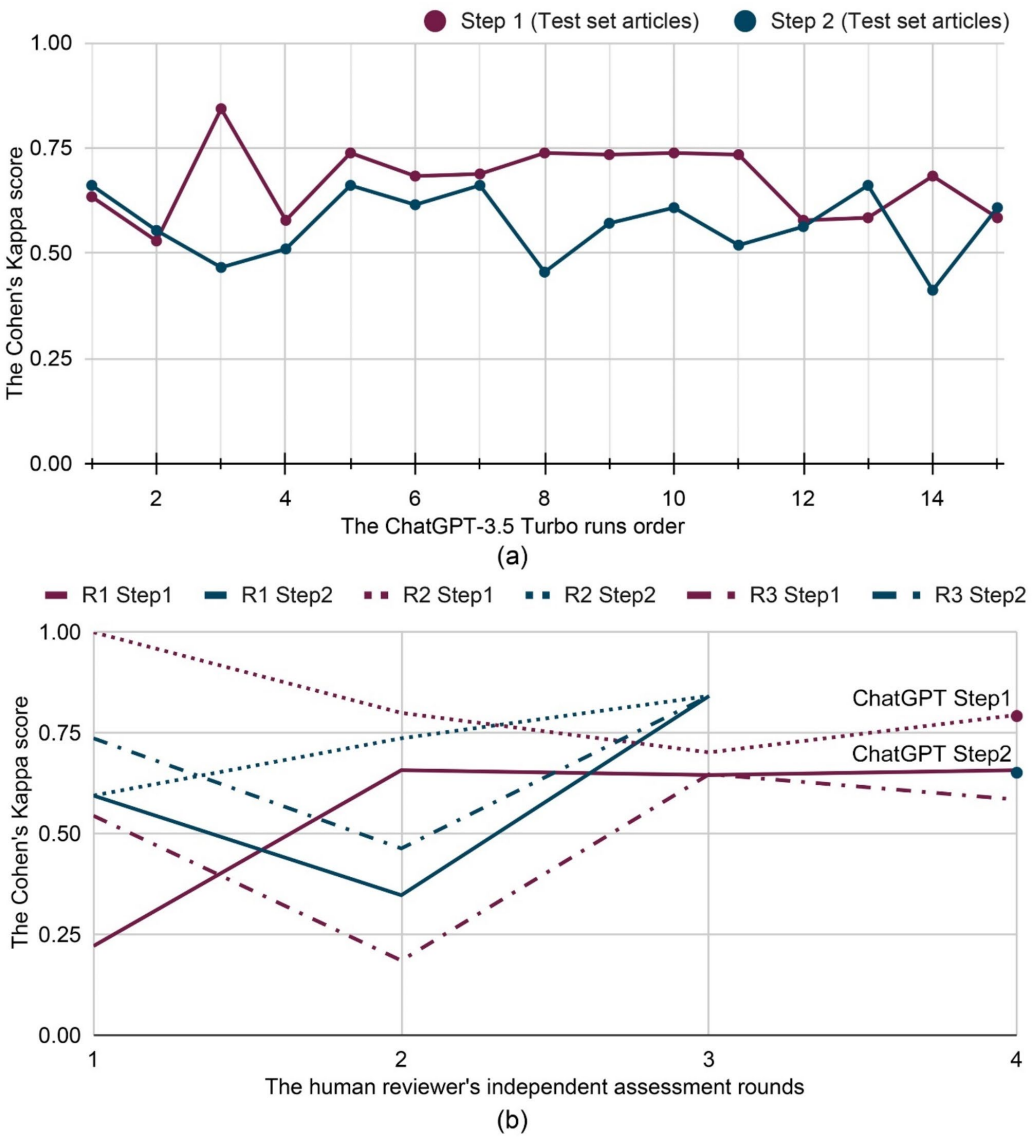
The human reviewer and ChatGPT-3.5 Turbo performance variability. (**a**) ChatGPT-3.5 Turbo prediction performance in each individual run in Steps 1 and 2. (**b**) The reviewer’s performance in different independent assessment rounds in Steps 1 and 2

Compared to reviewers, ChatGPT demonstrated a more stable performance, with Cohen’s Kappa scores ranging from 0.53 to 0.84 in Step 1 and from 0.46 to 0.66 in Step 2 (Fig. 6a, details in Appendix Table A13). In contrast, the Cohen’s Kappa scores of the three human reviewers range from 0.24 to 1.00 in Step 1 and 0.35 to 0.84 in Step 2 (Fig. 6b). Specifically, Reviewer ‘R2’ started with a perfect Kappa score of 1.00 in Step 1, but had a lower score of 0.60 in Step 2, while reviewers ‘R1’ and ‘R3’ demonstrated variable improvements (see Appendix Table A14). The relatively stable performance of GPT, compared to the fluctuating performance of human reviewers, suggests that GPT can potentially produce more reliable evidence in SRs.

Our findings also highlight the persistent variability in reviewer agreement, indicating that inter-reviewer agreements did not stabilize consistently over time. Although there was an overall improvement in reviewer agreement, with Fleiss’s Kappa increasing from 0.44 in Step 1 to 0.8 in Step 2, significant variability remained in all the rounds (see Appendix Table A15). The Cohen’s Kappa scores ranged from 0.22 to 0.73 across reviewer pairs (see Appendix Table A16). Additionally, the good performance of ‘R2’ might be due to the stronger domain knowledge on this topic, which influenced the review process disproportionately.

### ROI analysis between human reviewers and ChatGPT-3.5 turbo

**Fig. 7:**
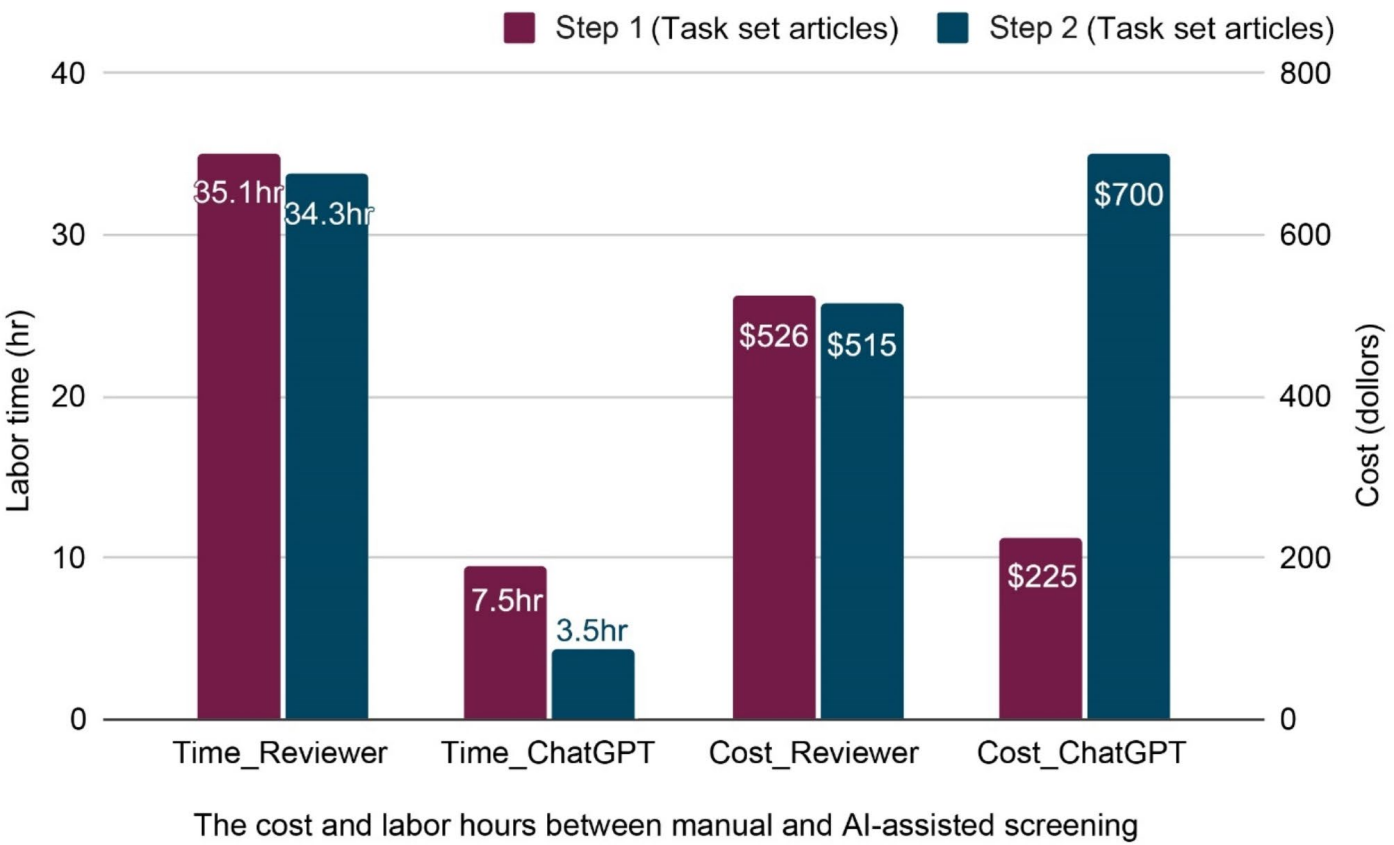
The ROI analysis results between reviewers and ChatGPT 3.5 Turbo

We compared costs and labor hours for manual and AI-assisted screening (Fig. 7). In the manual approach, Step 1 required screening 581 articles (3 min each, 29.1 h) plus two one-hour group discussions with three reviewers (6 h), totaling 35.1 h and $526. Step 2 involved screening 339 articles (5 min each, 28.3 h) with two group discussions (6 h), totaling 34.3 h and $515. Overall, the manual method costed 69.4 h and $1,041; In the AI-assisted workflow, Step 1 required 5 h for prompt refinement, 2 h for model fine-tuning, and 2.5 h for ChatGPT screening process. Costs included a $55 token fee and a $20 membership fee, totaling 7.5 h and $150. Step 2 spends 1 h for prompt refinement, 2.5 h for screening, and token costs increased to $700. Overall, the AI-assisted method costed 11 h and $925.

ROI analysis indicates that AI-assisted screening enhances efficiency. AI reduced screening time per article from 4.5 min to 0.55 min, resulting in 8× improvement and 87.8% time savings. With a time-based ROI of 7.16, each AI-assisted hour saves over 7 manual hours. AI also increased screening throughput from 13 to 108 articles per hour, saved $0.11 per article, and reduced screening costs by 10%. The overall ROI is 10.7%, indicating that AI-assisted screening provided a net gain compared to manual screening.

## Discussion

### Potential role of ChatGPT-3.5 turbo in structuring evidence screening for systematic reviews

**Fig. 8:**
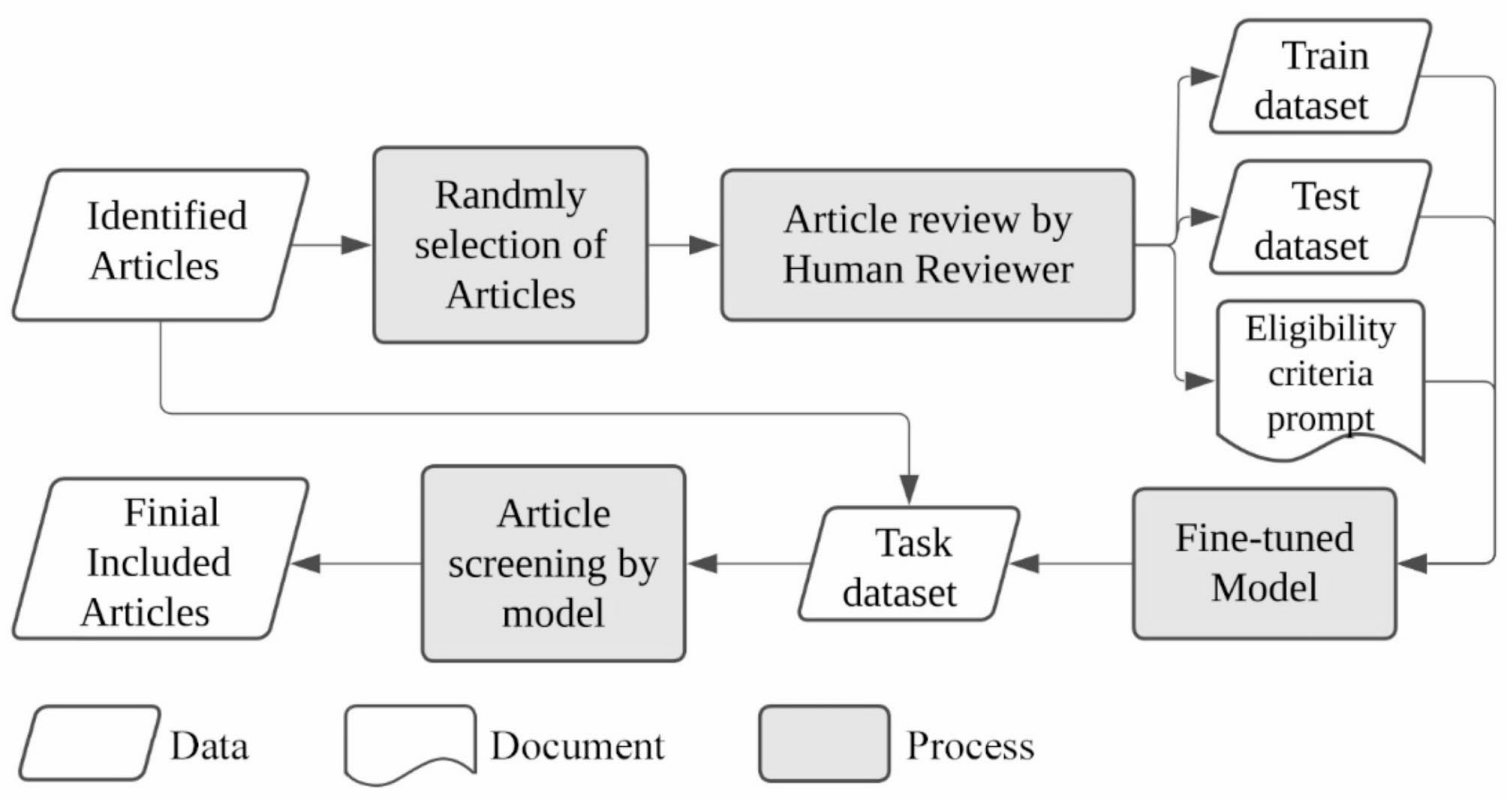
The framework of AI-assisted in evidence screening

This case study demonstrates how integrating ChatGPT-3.5 Turbo into the evidence screening process contributes a framework for AI-assisted SRs in environmental science (see Fig. 8). The AI model exhibited moderate to substantial agreement with reviewers, as reflected by Cohen’s Kappa scores, indicating its potential as a screening aid. However, while AI contributes to consistency in the screening process, we want to note that consistency does not inherently translate to greater accuracy. The overall reliability of SRs depends on human-defined criteria, validation protocols, and model fine-tuning decisions.

Our results indicate that fine-tuning ChatGPT-3.5 Turbo with domain-specific knowledge enables consistent application of eligibility criteria, reducing variability in screening decisions. Human reviewers’ judgments showed inconsistencies due to varying interpretations of eligibility criteria [36]. Differences in disciplinary perspectives may lead to variability in how eligibility criteria are applied, which can influence screening outcomes in SRs [37]. For instance, variations in defining LULC and key concepts like “direct relationship” and “statistical relationship” with fecal coliform contamination create screening discrepancies. However, AI-assisted screening may reduce discrepancies in applying criteria, though its impact on bias reduction requires further investigation. In our case, Reviewer ‘R2’ exhibited higher agreement with the consensus than ‘R1’ and ‘R3’, indicating that individual expertise influenced screening decisions. This finding suggests that an expert’s interpretation of the eligibility criteria may outweigh others, impacting the screening outcomes and introducing potential bias [16, 36], reinforcing the need for consistent eligibility criteria application that can be realized by AI-assisted automating repetitive screening [38, 39].

### Necessity of integrating domain knowledge with AI in environmental research

Several critical issues for defining eligibility criteria emerged in this case study SR, underscoring the importance of integrating human effort with ChatGPT-3.5 Turbo. First, the diverse classifications of LULC types across studies led to varying interpretations of LULC criteria (i.e., Criteria 1.1, 1.2, and 2.1). This issue was exacerbated by the reviewers’ different backgrounds and perspectives. For instance, some reviewers considered riparian zones as a type of land cover, while others did not define them as LULC. Reviewers’ opinions also varied on whether landscape ecology metrics (e.g., the number of patches, shape index) and best management practices related to land use meet the LULC criteria.

Second, the criteria of defining a “direct relationship” between LULC and fecal coliform contamination (Criteria 1.4) was also challenging for human reviewers to achieve consistency due to variations in research designs. For example, some studies focused on water quality index or ecological indicators like macroinvertebrates, where fecal coliform was not a direct indicator but rather a component of overall water quality assessment. Some research analyzed pollution sources associated with various LULC types without directly examining the impact of LULC on fecal coliform concentrations. Reviewers had different opinions on whether to include literature in these situations.

Third, defining a “statistical relationship” between LULC and fecal coliform contamination (Criteria 2.3) posed challenges in full-text screening. Some studies compared fecal coliform concentrations across different LULC types without quantifying LULC percentages or areas. Some studies summarized fecal coliform levels and LULC characteristics at individual sites but did not assess the quantitative relationship. Expert reviewers differed in judgments on whether these approaches can reflect a “statistical relationship” between LULC and fecal coliform contamination.

All three issues required consensus among all expert reviewers on how to define eligibility criteria, and the discrepancies were only able to be resolved through iterative discussions and arbitration. Through this process, reviewers refined eligibility criteria, which were then integrated into ChatGPT’s screening framework. Additionally, fine-tuning improved ChatGPT’s alignment with domain-specific eligibility criteria, reinforcing its ability to apply criteria consistently. Consequently, compared to the agreement between individual reviewers, ChatGPT-3.5 Turbo demonstrated a stronger alignment with the overall reviewer consensus., suggesting its potential as a supplementary screening tool.

### Limitations

Several limitations exist in our methodology. First, fine-tuning with human expertise enhances ChatGPT’s alignment with eligibility criteria but narrows the model’s versatility, making it less effective for broader applications [40]. Other environmental science SRs need to retrain or adapt the model using their own eligibility criteria to ensure relevance. Second, model performance depends on training dataset quality [25], which may not fully capture the complexity of relevant literature, especially with a limited sample size. Also, the inherent stochastic nature of ChatGPT may introduce variability across multiple runs [33], impacting consistency. Third, the model is constrained to text-based data and cannot effectively process non-textual inputs [33]. Overall, while the ChatGPT model demonstrates significant potential in structuring evidence screening, careful consideration of model training and validation are essential to ensure consistent performance and generalizability.

## Conclusion and future application in environmental science

This study presents an evidence-screening framework for SRs by integrating fine-tuned ChatGPT-3.5 Turbo with human expertise, though further validation is required for this exploratory step. AI-assisted methods can apply eligibility criteria consistently, improve efficiency, reduce labor demands, and manage disagreements in evidence screening for environmental SRs. To enhance AI-assisted screening across diverse environmental research domains, the following considerations should be addressed: (1) Improving training data quality and refining criteria with deeper domain expertise, (2) Advancing AI techniques to incorporate image data for more precise screening, and (3) Creating specialized language models tailored to the diverse methodologies and knowledge bases across different environmental science subfields. With continued collaboration between humans and AI, these advancements will enhance the model’s adaptability and effectiveness in SRs.

## Electronic supplementary material

Below is the link to the electronic supplementary material.

Supplementary Material 1: Text appendix show the Title & screening prompt and Full-text screening prompt. Tables show a search queries for datasets; an articles identified results; the article screening results of Steps 1 and 2; the criteria’s different versions in Steps 1 and 2; the screening results of ChatGPT-3.5 Turbo in Steps 1 and 2; the review results of human reviewers in Steps 1 and 2; the human reviewer and ChatGPT’s screening result of test set articles in Steps 1 and 2; the Cohen’s Kappa score of ChatGPT-3.5 Turbo at Steps 1 and 2; the Cohen’s Kappa score of human reviewers at Steps 1 and 2.

Supplementary Material 2

Supplementary Material 3

Supplementary Material 4

Supplementary Material 5

Supplementary Material 6

Supplementary Material 7

Supplementary Material 8

Supplementary Material 9

Supplementary Material 10

Supplementary Material 11

Supplementary Material 12

Supplementary Material 13

Supplementary Material 14

Supplementary Material 15

Supplementary Material 16

## Data Availability

The fine-turned ChatGPT 3.5 Turbo model and raw data are available on GitHub (https://github.com/billbillbilly/GPT_Pytools.git).
