# Supplementary material for: AI-assisted evidence screening method for systematic reviews in environmental research: integrating ChatGPT with domain knowledge
Source: Environ Evid. 2025 Apr 15;14:5. doi: 10.1186/s13750-025-00358-5 (PMC11998256; doi:10.1186/s13750-025-00358-5)
Supplement: Supplementary file 2 — Supplementary Material 2 [file 13750_2025_358_MOESM2_ESM.docx]

**Table A1.** The Search queries for databases

| **Database** | **Search Queries** |
| --- | --- |
| Scopus | TITLE-ABS-KEY(( "land use" OR "landuse" OR "land cover" OR "landcover" ) AND ( "E. coli" OR "escherichia coli" OR "fecal" OR "faecal" OR "coliform*" ) AND ( "stream" OR "streamlet" OR "river" OR "riverine" OR "rivulet" OR "creek" OR "brook" OR "watercourse" OR "waterway" OR "tributary" OR "branch" OR "flow" )) AND PUBYEAR > 1999 AND PUBYEAR < 2025 AND ( LIMIT-TO ( DOCTYPE , "ar" ) OR LIMIT-TO ( DOCTYPE , "re" ) ) AND ( LIMIT-TO ( PUBSTAGE , "final" ) ) AND ( LIMIT-TO ( SRCTYPE , "j" ) ) AND ( LIMIT-TO ( LANGUAGE , "English" ) ) |
| Web of Science | TI=(( "land use" OR "landuse" OR "land cover" OR "landcover" ) AND ( "E. coli" OR "escherichia coli" OR "fecal" OR "faecal" OR "coliform*" ) AND ( "stream" OR "streamlet" OR "river" OR "riverine" OR "rivulet" OR "creek" OR "brook" OR "watercourse" OR "waterway" OR "tributary" OR "branch" OR "flow" )) OR AB=(( "land use" OR "landuse" OR "land cover" OR "landcover" ) AND ( "E. coli" OR "escherichia coli" OR "fecal" OR "faecal" OR "coliform*" ) AND ( "stream" OR "streamlet" OR "river" OR "riverine" OR "rivulet" OR "creek" OR "brook" OR "watercourse" OR "waterway" OR "tributary" OR "branch" OR "flow" )) OR AK=(( "land use" OR "landuse" OR "land cover" OR "landcover" ) AND ( "E. coli" OR "escherichia coli" OR "fecal" OR "faecal" OR "coliform*" ) AND ( "stream" OR "streamlet" OR "river" OR "riverine" OR "rivulet" OR "creek" OR "brook" OR "watercourse" OR "waterway" OR "tributary" OR "branch" OR "flow" )) and Preprint Citation Index (Exclude – Database) and 2024 or 2023 or 2022 or 2021 or 2020 or 2019 or 2018 or 2017 or 2016 or 2015 or 2014 or 2013 or 2012 or 2011 or 2010 or 2009 or 2008 or 2007 or 2006 or 2005 or 2004 or 2003 or 2002 or 2001 or 2000 (Publication Years) and Article or Dissertation Thesis or Review Article (Document Types) and English (Languages) |
| ProQuest | (((((((((((abstract(("land use" OR "landuse" OR "land cover" OR "landcover") AND ("E. coli" OR "escherichia coli" OR "fecal" OR "faecal" OR "coliforms" OR "coliform") AND ("stream" OR "streamlet" OR "river" OR "riverine" OR "rivulet" OR "creek" OR "brook" OR "watercourse" OR "waterway" OR "tributary" OR "branch" OR "flow")) OR title(("land use" OR "landuse" OR "land cover" OR "landcover") AND ("E. coli" OR "escherichia coli" OR "fecal" OR "faecal" OR "coliforms" OR "coliform") AND ("stream" OR "streamlet" OR "river" OR "riverine" OR "rivulet" OR "creek" OR "brook" OR "watercourse" OR "waterway" OR "tributary" OR "branch" OR "flow"))) AND at.exact("Literature Review" OR "Essay" OR "Conference Paper" OR "Article")) AND la.exact("English")) AND at.exact("Literature Review" OR "Essay" OR "Conference Paper" OR "Article")) AND la.exact("English")) AND at.exact("Literature Review" OR "Essay" OR "Conference Paper" OR "Article")) AND la.exact("English")) AND at.exact("Literature Review" OR "Essay" OR "Conference Paper" OR "Article")) AND la.exact("English")) AND at.exact("Literature Review" OR "Essay" OR "Conference Paper" OR "Article")) AND la.exact("English")) AND PEER(yes) AND PEER(yes) |
| PubMed | (("land use"[Title/Abstract] OR "landuse"[Title/Abstract] OR "land cover"[Title/Abstract] OR "landcover"[Title/Abstract]) AND ("e coli"[Title/Abstract] OR "escherichia coli"[Title/Abstract] OR "fecal"[Title/Abstract] OR "faecal"[Title/Abstract] OR "coliform*"[Title/Abstract]) AND ("stream"[Title/Abstract] OR "streamlet"[Title/Abstract] OR "river"[Title/Abstract] OR "riverine"[Title/Abstract] OR "rivulet"[Title/Abstract] OR "creek"[Title/Abstract] OR "brook"[Title/Abstract] OR "watercourse"[Title/Abstract] OR "waterway"[Title/Abstract] OR "tributary"[Title/Abstract] OR "branch"[Title/Abstract] OR "flow"[Title/Abstract] )) AND ((excludepreprints[Filter]) AND (fft[Filter]) AND (english[Filter])) |
