# Supplementary material for: AI-assisted evidence screening method for systematic reviews in environmental research: integrating ChatGPT with domain knowledge
Source: Environ Evid. 2025 Apr 15;14:5. doi: 10.1186/s13750-025-00358-5 (PMC11998256; doi:10.1186/s13750-025-00358-5)
Supplement: Supplementary file 3 — Supplementary Material 3 [file 13750_2025_358_MOESM3_ESM.docx]

**Table A2.** The identified 711 articles

| **Unique ID** | **Title** | **DOI** |
| --- | --- | --- |
| 1 | !Cuba! river water chemistry reveals rapid chemical weathering, the echo of uplift, and the promise of more sustainable agriculture | 10.1130/GSATG419A.1 |
| 2 | Escherichia coli concentration, multiscale monitoring over the decade 2011–2021 in the Mekong River basin, Lao PDR | 10.5194/essd-14-2883-2022 |
| 3 | A 22-Site Comparison of Land-Use Practices, E-coli and Enterococci Concentrations | 10.3390/ijerph192113907 |
| 4 | A Basis Water Quality Monitoring Plan For Rehabilitation And Protection | 10.22034/gjesm.2022.02 |
| 5 | A biological and nitrate isotopic assessment framework to understand eutrophication in aquatic ecosystems | 10.1016/j.scitotenv.2020.136909 |
| 6 | A case study characterizing animal fecal sources in surface water using a mitochondrial DNA marker | 10.1007/s10661-017-6107-z |
| 7 | A comparison of soil Staphylococcus aureus and fecal indicator bacteria concentrations across land uses in a Hawaiian watershed | 10.1002/jeq2.20380 |
| 8 | A comprehensive method for ampliconbased and metagenomic characterization of viruses, bacteria, and eukaryotes in freshwater samples | 10.1186/s40168-016-0166-1 |
| 9 | A geographical approach to tracking Escherichia coli and other water quality constituents in a Texas coastal plains watershed | 10.1007/s10661-012-2895-3 |
| 10 | A geospatial analysis of land use and stormwater management on fecal coliform contamination in North Carolina streams | 10.1016/j.scitotenv.2017.02.093 |
| 11 | A hydrochemically guided landscape classification system for modelling spatial variation in multiple water quality indices: Process-attribute mapping | 10.1016/j.scitotenv.2019.03.492 |
| 12 | A load model based on antecedent dry periods for pollutants in stormwater | 10.2175/106143007X220888 |
| 13 | A Multivariate and Spatiotemporal Analysis of Water Quality in Code River, Indonesia | 10.1155/2020/8897029 |
| 14 | A multivariate statistical approach to the integration of different land-uses, seasons, and water quality as water resources management tool | 10.1007/s10661-019-7647-1 |
| 15 | A novel approach for assessing watershed susceptibility using weighted overlay and analytical hierarchy process (AHP) methodology: a case study in Eagle Creek Watershed, USA | 10.1007/s11356-019-06355-9 |
| 16 | A review of agricultural land use by shorebirds with special reference to habitat conservation in the Fraser River Delta, British Columbia | 10.4141/P06-137 |
| 17 | A Review of On-farm Roadway Runoff Characterisation and Potential Management Options for Ireland | 10.1007/s11270-021-05027-0 |
| 18 | A short review of fecal indicator bacteria in tropical aquatic ecosystems: Knowledge gaps and future directions | 10.3389/fmicb.2015.00308 |
| 19 | A Socio-ecological Systems Perspective on Planning for Informality | NA |
| 20 | A spatial assessment of baseline nutrient and water quality values in the Ashepoo-Combahee-Edisto (ACE) Basin, South Carolina, USA | 10.1016/j.marpolbul.2015.07.035 |
| 21 | A spatial-statistical approach for modeling the effect of non-point source pollution on different water quality parameters in the Velhas river watershed--Brazil | 10.1016/j.jenvman.2006.12.009 |
| 22 | A Spatiotemporal Analysis of Water Quality and Land Use in Tambayakbayan River, Yogyakarta | 10.1088/1755-1315/933/1/012045 |
| 23 | A study focused on the risk of illness from Escherichia coli in recreational-use water, using the Red Cedar Watershed as a model | NA |
| 24 | A systematic assessment of watershed-scale nonpoint source pollution during rainfall-runoff events in the Miyun Reservoir watershed | 10.1007/s11356-017-0946-6 |
| 25 | A watershed study assessing effects of commercial hog operations on microbial water quality in North Carolina, USA | 10.1016/j.scitotenv.2022.156085 |
| 26 | Abiotic and biotic changes at the basin scale in a tropical dry forest landscape after Hurricanes Jova and Patricia in Jalisco, Mexico | 10.1016/j.foreco.2017.10.015 |
| 27 | Abundance, diversity, and host assignment of total, intracellular, and extracellular antibiotic resistance genes in riverbed sediments | 10.1016/j.watres.2022.118363 |
| 28 | Achieving sustainable water and land use systems in highly developed tropical landscapes | 10.1088/1748-9326/ac8ab6 |
| 29 | Actinomycetes in the Elbow River Basin, Alberta, Canada | 10.2166/wqrj.2003.007 |
| 30 | Adaptive variations of sediment microbial communities and indication of fecal-associated bacteria to nutrients in a regulated urban river | 10.3390/W12051344 |
| 31 | Adopting basic quality tools and landscape analysis for applied limnology: an approach for freshwater reservoir management | 10.1007/s40899-022-00655-8 |
| 32 | Advances in modeling, sampling, and assessing the anthropogenic contamination potential of fractured bedrock aquifers | NA |
| 33 | Advancing understanding of land use and physicochemical impacts on fecal contamination in mixed-land-use watersheds | 10.3390/W12041094 |
| 34 | Agricultural land use changes stream dissolved organic matter via altering soil inputs to streams | 10.1016/j.scitotenv.2021.148968 |
| 35 | An applied ecological approach for the assessment of anthropogenic disturbances in urban wetlands and the contributor river | 10.1016/j.ecocom.2020.100852 |
| 36 | An assessment of groundwater potential and vulnerability in the Upper Manyame Sub-Catchment of Zimbabwe | 10.1016/j.pce.2018.03.003 |
| 37 | An Assessment of In-situ Water Quality Parameters and its variation with Landsat 8 Level 1 Surface Reflectance datasets | 10.1080/03067319.2021.1954175 |
| 38 | An assessment of water quality and urbanization in the Gills Creek Watershed | NA |
| 39 | An evaluation of a Lake Houston tributary, Cypress Creek, for contamination and water quality | NA |
| 40 | An INCA model for pathogens in rivers and catchments: Model structure, sensitivity analysis and application to the River Thames catchment, UK | 10.1016/j.scitotenv.2016.01.128 |
| 41 | An integrated watershed modeling methodology for water quality restoration | NA |
| 42 | Analysis of Escherichia coli and enterococci concentrations patterns in a Pennsylvania creek using empirical orthogonal functions | 10.2134/jeq2019.05.0191 |
| 43 | Analysis of the HSPF water quality parameter uncertainty in predicting peak in-stream fecal coliform concentrations | NA |
| 44 | Analytical and detection sources of pollution based environmetric techniques in malacca river, Malaysia | 10.15666/aeer/1501_485499 |
| 45 | Anthropogenic influence on surface water quality of the Nhue and Day sub-river systems in Vietnam | 10.1007/s10653-009-9279-9 |
| 46 | Antimicrobial resistance of Escherichia coli isolated from freshwaters and hospital effluents in Belgium | 10.1111/lam.13625 |
| 47 | Application of Coagulation and Foam Concentration Method to Quantify Waterborne Pathogens in River Water Samples | 10.3390/w14223642 |
| 48 | Application of host-specific genetic markers for microbial source tracking of faecal water contamination in an agricultural catchment | 10.1080/09064710.2014.941392 |
| 49 | Application of indexes to assess the water quality of coastal basin of the Sapucaia in Sergipe | 10.1590/S1413-41522017159832 |
| 50 | Application of Microbial Source Tracking Techniques to Characterize Fecal Pollution Entering Taihu Lake (China) | NA |
| 51 | Application of Multivariate Statistical Methodology to Model Factors Influencing Fate and Transport of Fecal Pollution in Surface Waters | NA |
| 52 | Applications of source-tracking and site-specific models for water quality assessment of tidal waters: Upper inlet creek, mount pleasant, South Carolina | NA |
| 53 | Applying MAR analysis to identify human and non-human fecal sources in small Kentucky watersheds | 10.1007/s11270-008-9761-5 |
| 54 | Applying the Manning equation to determine the critical distance in non-point source pollution using remotely sensed data and cartographic modelling | 10.1117/12.2028480 |
| 55 | Assessing environmental contamination of River Ganga using correlation and multivariate analysis | 10.7508/pj.2015.03.003 |
| 56 | Assessing land-cover effects on stream water quality in metropolitan areas using the water quality index | 10.3390/w12113294 |
| 57 | Assessing Natural and Anthropogenic Drivers of Regional Water Quality using Hierarchical Modeling | NA |
| 58 | Assessing performance of local materials for the treatment of dry weather flows in open drains: Results of semi-controlled field experiment research in Bangalore, India | 10.1016/j.ecoleng.2021.106506 |
| 59 | Assessing the impact of modern recharge on a sandstone aquifer beneath a suburb of Doncaster, UK | 10.1007/s10040-006-0028-1 |
| 60 | Assessing the impacts of watershed indexes and precipitation on spatial in-stream E. coli concentrations | 10.1016/j.ecolind.2012.05.023 |
| 61 | Assessing the service of water quality regulation by quantifying the effects of land use on water quality and public health in central Veracruz, Mexico | 10.1016/j.ecoser.2016.09.001 |
| 62 | Assessing the yield and load of contaminants with stream order: Would policy requiring livestock to be fenced out of high-order streams decrease catchment contaminant loads? | 10.2134/jeq2017.05.0212 |
| 63 | Assessing Water Quality Dynamics in Tidally Influenced Blackwater Rivers along a Rural-Urban Gradient | 10.3390/w15234154 |
| 64 | Assessing water use and quality through youth participatory research in a rural Andean watershed | 10.1016/j.jenvman.2009.04.014 |
| 65 | Assessment of characteristics, water quality and groundwater vulnerability in Pakis District, East Java Province, Indonesia | 10.24425/jwld.2022.140382 |
| 66 | Assessment of emerging hydrological, water quality issues and policy discussion on water sharing of transboundary Kabul River | 10.2166/wp.2017.119 |
| 67 | Assessment of fecal coliform and Escherichia coli across a land cover gradient in west Georgia streams | 10.1007/s12403-012-0073-z |
| 68 | Assessment of Physico-Chemical and Microbiological Parameters of Mthatha River in Eastern Cape, South Africa | 10.1080/15275922.2023.2297419 |
| 69 | Assessment of the impacts of landscape patterns on water quality in Trondheim rivers and Fjord, Norway | 10.2166/ws.2022.181 |
| 70 | Assessment of water quality and identification of pollution risk locations in Tiaoxi River (Taihu Watershed), China | 10.3390/w10020183 |
| 71 | Assessment of water quality based on statistical analysis of physical-chemical, biomonitoring and land use data: Manso River supply reservoir | 10.1016/j.scitotenv.2023.169554 |
| 72 | Assessment of water quality in association with land use in the Tillamook Bay Watershed, Oregon, USA | 10.1007/s11270-005-2443-7 |
| 73 | Assessment of Water Quality of Four Mahoning River Sub-Watersheds, Northeast Ohio | NA |
| 74 | Assessment of water quality, benthic invertebrates, and periphyton in the Threemile Creek Basin, Mobile, Alabama, 1999-2003. | NA |
| 75 | Assessment, modelling and management of land use and water quality in the upper Taieri River catchment | 10.1080/00288233.2013.822004 |
| 76 | Associations among pathogenic bacteria, parasites, and environmental and land use factors in multiple mixed-use watersheds | 10.1016/j.watres.2011.06.021 |
| 77 | Bacteria modeling with SWAT for assessment and remediation studies: A review | NA |
| 78 | Bacterial capture by peptide-mimetic oligoacyllysine surfaces | 10.1128/AEM.00532-10 |
| 79 | Bacterial community composition and structure in an Urban River impacted by different pollutant sources | 10.1016/j.scitotenv.2016.05.168 |
| 80 | Bacterial community structure is indicative of chemical inputs in the Upper Mississippi River | 10.3389/fmicb.2014.00524 |
| 81 | Bacterial indicators of faecal pollution in the waters of the El-Kabir River and Akkar watershed in Syria and Lebanon | 10.1111/j.1440-1770.2005.00265.x |
| 82 | Bacterial loadings Watershed Model in Copano Bay | NA |
| 83 | Bacterial pathogens in Hawaiian coastal streams--associations with fecal indicators, land cover, and water quality | 10.1016/j.watres.2011.03.033 |
| 84 | Bacterial source tracking and shellfish contamination in a coastal catchment | 10.2166/wst.2003.0676 |
| 85 | Bacterial source tracking and survival of Escherichia coli | NA |
| 86 | Bacterial total maximum daily load (TMDL): Development and evaluation of a new classification scheme for impaired waterbodies of Texas | NA |
| 87 | Bacterial, fungal and algal population of pennar river: A fresh water wetland in Kottayam district, Kerala | NA |
| 88 | Bacteriological quality of South African irrigation water and its role as a source of contamination on irrigated lettuce | NA |
| 89 | Beach sands along the California coast are diffuse sources of fecal bacteria to coastal waters | 10.1021/es062822n |
| 90 | Bile acids combined with fecal sterols: a multiple biomarker approach for deciphering fecal pollution using river sediments | 10.1007/s11368-016-1592-1 |
| 91 | Biogeochemical and Microbial Indicators of Land-Use Change in a Northern Gulf of Mexico Estuary | NA |
| 92 | Biogeochemical impacts of sewage effluents in predominantly rural river catchments: Are point source inputs distinct to background diffuse pollution? | 10.1016/j.jenvman.2022.114891 |
| 93 | Biogeochemical influence of Chinese privet in riparian forests in west Georgia and the influences of oyster harvesting in Apalachicola Bay, Florida | NA |
| 94 | Biogeographic patterns of potential pathogenic bacteria in the middle and lower reaches of the Yangtze River as well as its two adjoining lakes, China | 10.3389/fmicb.2022.972243 |
| 95 | Biomonitoring and Water Quality Evaluation of River Beas in Mid Himalayan Zone, India | 10.55003/cast.2022.05.22.002 |
| 96 | Biomonitoring in the Anthropocene: Environmental DNA (eDNA) Assessments of Changing Ecosystems | NA |
| 97 | BSLC: A tool for bacteria source characterization for watershed management | NA |
| 98 | Calibration and sensitivity analysis of a novel water flow and pollution model for future city planning: Future Urban Stormwater Simulation (FUSS) | 10.2166/wst.2022.046 |
| 99 | Causal connections between water quality and land use in a rural tropical island watershed: rural tropical island watershed analysis | 10.1007/s10393-010-0299-9 |
| 100 | Changes in chemical and physical propertiesof stream water across an urban-rural gradient in western Georgia | 10.1007/s11252-005-1422-5 |
| 101 | Changes in Land Use Land Cover (LULC), Surface Water Quality and Modelling Surface Discharge in Beaver Creek Watershed, Northeast Tennessee and Southwest Virginia | NA |
| 102 | Changes in land use/management and water quality in the Long Creek watershed | 10.1111/j.1752-1688.2002.tb04374.x |
| 103 | Characterisation of sludge produced by the agri-food industry and recycling options for its agricultural uses in a typical Mediterranean area, the Segura River basin (Spain) | 10.1016/j.wasman.2018.10.020 |
| 104 | Characteristics of urban development and associated stormwater quality | NA |
| 105 | Characterization and prediction of stormwater runoff quality in sub-tropical rural catchments | 10.1134/S0097807817020129 |
| 106 | Characterization of and Relations Among Precipitation, Streamflow, Suspended-Sediment, and Water-Quality Data at the U.S. Army Garrison Fort Carson and Pinon Canyon Maneuver Site, Colorado, Water Years 2016-18 | 10.3133/sir20225018 |
| 107 | Characterization of nonpoint source microbial contamination in an urbanizing watershed serving as a municipal water supply | 10.1016/j.watres.2012.09.009 |
| 108 | Characterization of rain and roof drainage water quality in Xanthi, Greece | 10.1007/s10661-006-9254-1 |
| 109 | Characterization of sources and loadings of fecal pollutants using microbial source tracking assays in urban and rural areas of the Grand River Watershed, Southwestern Ontario | 10.1016/j.watres.2014.01.003 |
| 110 | Characterization of Water Quality Pollution in Mixed Land Use Watersheds | NA |
| 111 | Characterizing Differences in Sources of and Contributions to Fecal Contamination of Sediment and Surface Water with the Microbial FIT Framework | 10.1021/acs.est.2c00224 |
| 112 | Characterizing relationships among fecal indicator bacteria, microbial source tracking markers, and associated waterborne pathogen occurrence in stream water and sediments in a mixed land use watershed | 10.1016/j.watres.2016.05.014 |
| 113 | Chemical and microbiological indicators to assess the impact of agricultural activities on groundwater in the Pampean agro-ecosystem | 10.1007/s40899-023-00933-z |
| 114 | Chronic urban hotspots and agricultural drainage drive microbial pollution of karst water resources in rural developing regions | 10.1016/j.scitotenv.2020.140898 |
| 115 | Classification and prediction of fecal coliform in stream waters using decision trees (Dts) for upper green river watershed, kentucky, usa | 10.3390/w13192790 |
| 116 | Climate and land-use change impact on faecal indicator bacteria in a temperate maritime catchment (the River Conwy, Wales) | 10.1016/j.jhydrol.2017.08.011 |
| 117 | Climate change and land use drivers of fecal bacteria in tropical hawaiian rivers | 10.2134/jeq2014.01.0025 |
| 118 | Climate change mitigation for agriculture: water quality benefits and costs | 10.2166/wst.2008.906 |
| 119 | Climate warming, environmental degradation and pollution as drivers of antibiotic resistance | 10.1016/j.envpol.2024.123649 |
| 120 | CLUES model calibration and its implications for estimating contaminant attenuation | 10.1016/j.agwat.2019.105853 |
| 121 | CLUES model calibration: residual analysis to investigate potential sources of model error | 10.1080/00288233.2019.1697708 |
| 122 | Coherence among different microbial source tracking markers in a small agricultural stream with or without livestock exclusion practices | 10.1128/AEM.01626-13 |
| 123 | Coliform status of water bodies from two Districts in Ghana, West Africa: Implications for rural water resources management | 10.2166/wp.2010.013 |
| 124 | Combining land use information and small stream sampling with PCR-based methods for better characterization of diffuse sources of human fecal pollution | 10.1021/es2003167 |
| 125 | Comparing the Fate and Transport of MS2 Bacteriophage and Sodium Fluorescein in a Karstic Chalk Aquifer | 10.3390/pathogens13020168 |
| 126 | Comparison of Biofiltration Media in Treating Industrial Stormwater Runoff | NA |
| 127 | Comparison of qPCR and amplicon sequencing based methods for fecal source tracking in a mixed land use estuarine watershed | 10.1039/c9ew00719a |
| 128 | Comparison of the performance of decision tree (DT) algorithms and extreme learning machine (ELM) model in the prediction of water quality of the Upper Green River watershed | 10.1002/wer.1642 |
| 129 | Complexity and uncertainty in human and ecological risk assessment | NA |
| 130 | Compositions of first flush and composite storm water runoff in small urban and rural watersheds, north-central Texas | 10.1080/15730620600578678 |
| 131 | Comprehensive Evaluation of Bacteroidales for Identification of Fecal Contamination Sources in Freshwater | NA |
| 132 | Confirming the Source of High-Sulfate Concentrations in Dead Horse Creek, Winkler, Manitoba, Canada, Using a Dual-Isotope Bayesian Probability Mixing Model | 10.1007/s11270-020-04887-2 |
| 133 | Connecting microbial, nutrient, physiochemical, and land use variables for the evaluation of water quality within mixed use watersheds | 10.1016/j.watres.2022.118526 |
| 134 | Conservation biology of the Cross River gorilla (Gorilla gorilla diehli) | NA |
| 135 | Conservation genetics of neotropical otters (lontra longicaudis) in méxico | NA |
| 136 | Contamination with bacterial zoonotic pathogen genes in U.S. streams influenced by varying types of animal agriculture | 10.1016/j.scitotenv.2016.04.087 |
| 137 | Controls on the chemical and isotopic compositions of urban stormwater in a semiarid zone | 10.1016/j.jhydrol.2004.02.010 |
| 138 | Converting treatment wetlands into treatment gardens: Use of ornamental plants for greywater treatment | 10.1016/j.scitotenv.2020.140889 |
| 139 | Coupled Dynamics of Fecal Indicator Bacteria in Sandy Sediments and the Water Column: a 3-Year High-Frequency Study at a Pennsylvania Creek | 10.1007/s11270-023-06371-z |
| 140 | COVID-19 Lockdown Pandemic Period Effects in Highly Impacted Aquatic Ecosystems | 10.1002/etc.5551 |
| 141 | Cryptosporidium genotyping and land use mapping for hazard identification and source tracking in a small mixed Rural-Urban watershed in Southeastern Brazil | 10.2166/wh.2018.143 |
| 142 | Cryptosporidium source tracking in the potomac river watershed | 10.1128/AEM.01345-08 |
| 143 | Current state of water quality indicators in urban streams in New Zealand | 10.1080/00288330.2020.1753787 |
| 144 | Decadal and seasonal water quality trends downstream of urban and rural areas in Southern Alberta rivers | 10.2166/wqrjc.2012.033 |
| 145 | Deciphering the influence of multiple anthropogenic inputs on taxonomic and functional profiles of the microbial communities in Yitong River, Northeast China | 10.1007/s11356-021-18386-2 |
| 146 | Demonstrating an Integrated Antibiotic Resistance Gene Surveillance Approach in Puerto Rican Watersheds Post-Hurricane Maria | 10.1021/acs.est.0c05567 |
| 147 | Detangling Seasonal Relationships of Fecal Contamination Sources and Correlates with Indicators in Michigan Watersheds | 10.1128/spectrum.00415-22 |
| 148 | Detection and Monitoring of Microbes of Concern in Animal Production Environment | NA |
| 149 | Detection of Helicobacter pylori and fecal indicator bacteria in five North American rivers | 10.2166/wh.2005.054 |
| 150 | Detection of human enteric viruses in stream water with RT-PCR and cell culture | 10.2166/wh.2004.0004 |
| 151 | Detection of human-associated bacteria in water from Akiyoshi-do Cave, Japan | 10.1002/wer.1355 |
| 152 | Detection of SARS-CoV-2 in urban stormwater: An environmental reservoir and potential interface between human and animal sources | 10.1016/j.scitotenv.2021.151046 |
| 153 | Determinants of spatio-temporal variability of water quality in the Barotse Floodplain, western Zambia | 10.7809/b-e.00310 |
| 154 | Determination effects of impervious areas on urban watershed | 10.1007/s11356-014-3345-2 |
| 155 | Determination of urban groundwater pollution in alluvial aquifer using linked process models considering urban water cycle | 10.1016/j.jhydrol.2009.08.025 |
| 156 | Determination ofWater Quality of Rivers under Various Land Use Activities Using Physico-chemical Parameters and Bacterial Populations in Northern Peninsular Malaysia | NA |
| 157 | Determining hot spots of fecal contamination in a tropical watershed by combining land-use information and meteorological data with source-specific assays | 10.1021/es304066z |
| 158 | Determining overall water quality related to anthropogenic influences across freshwater systems of Thailand | 10.1080/07900627.2016.1142862 |
| 159 | Determining the degree of fecal pollution in natural waterways feeding the Mississippi River in Hancock County, Illinois | NA |
| 160 | Determining the primary sources of fecal pollution using microbial source tracking assays combined with land-use information in the Edwards Aquifer | 10.1016/j.watres.2020.116211 |
| 161 | Developing alternative regression models for describing water quality using a self-organizing map | 10.1080/19443994.2015.1112981 |
| 162 | Development and application of ecosystem health indicators in the North American Great Lakes Basin | NA |
| 163 | Development and application of exceedance model for surface water quality parameters | 10.15244/pjoes/124116 |
| 164 | Development and evaluation of the bacterial fate and transport module for the Agricultural Policy/Environmental eXtender (APEX) model | 10.1016/j.scitotenv.2017.09.231 |
| 165 | Development of a national-scale framework to characterise transfers of N, P and Escherichia coli from land to water | 10.1080/00288233.2020.1713822 |
| 166 | Development of a pathogen transport model for Irish catchments using SWAT | 10.1016/j.agwat.2009.08.017 |
| 167 | Development of a process-based model to predict pathogen budgets for the Sydney drinking water catchment | 10.2166/wh.2007.013b |
| 168 | Development of a risk-based index for source water protection planning, which supports the reduction of pathogens from agricultural activity entering water resources | 10.1016/j.jenvman.2006.12.048 |
| 169 | Development of Multiple Regression Models to Predict Sources of Fecal Pollution | 10.2175/106143017X14839994523901 |
| 170 | Development of regression-based models to predict fecal bacteria at the Illinois River Basin, Arkansas and Oklahoma | NA |
| 171 | Diffuse and point pollution impacts on the pathogen indicator organism level in the Geum River, Korea | 10.1016/j.scitotenv.2005.01.021 |
| 172 | Dissolved Inorganic Nitrogen, Soluble Reactive Phosphorous, and Microbial Pollutant Loading from Tropical Rural Watersheds in Hawai'i to the Coastal Ocean During Non-Storm Conditions | 10.1007/s12237-010-9352-8 |
| 173 | Distribution and diversity of Escherichia coli populations in the South Nation River drainage basin, eastern Ontario, Canada | 10.1128/AEM.02288-09 |
| 174 | Diverse Land Use and the Impact on (Irrigation) Water Quality and Need for Measures - A Case Study of a Norwegian River | 10.3390/ijerph120606979 |
| 175 | DNA fingerprinting using BOX-A1R and (GTG)(5) primers identify spatial variations of fecal contamination along Pasig River, Philippines | 10.1007/s10661-022-10504-y |
| 176 | Do reductions in agricultural field drainage during the growing season impact bacterial densities and loads in small tile-fed watersheds? | 10.1016/j.watres.2018.11.074 |
| 177 | Does land use affect pathogen presence in New Zealand drinking water supplies? | 10.1016/j.watres.2020.116229 |
| 178 | Earlier detection of rumors in online social networks using certainty-factor-based convolutional neural networks | 10.1007/s13278-020-00634-x |
| 179 | Ecological Status of Aquatic Communities in Selected Streams in the Milwaukee Metropolitan Sewerage District Planning Area of Wisconsin, 2004-13. | NA |
| 180 | Ecological water health assessment using benthic macroinvertebrate communities (case study: the Ghezel Ozan River in Zanjan Province, Iran) | 10.1007/s10661-019-7894-1 |
| 181 | Ecology of Tigers in Churia Habitat and a Non-Invasive Genetic Approach to Tiger Conservation in Terai Arc, Nepal | NA |
| 182 | Ecosystem dynamics and pollution effects in an Ozark cave stream | 10.1111/j.1752-1688.2003.tb04434.x |
| 183 | Ecosystemic assessment of surface water quality in the Virilla River: Towards sanitation processes in Costa Rica | 10.3390/w10070845 |
| 184 | Effect of agricultural activities on surface water quality from paramo ecosystems | 10.1007/s11356-022-21709-6 |
| 185 | Effect of development on water quality for seven streams in North Carolina | 10.1007/s10661-012-3024-z |
| 186 | Effect of human development on bacteriological water quality in coastal watersheds | 10.1890/1051-0761(2000)010[1047:EOHDOB]2.0.CO;2 |
| 187 | Effect of land use and hydrological processes on Escherichia coli concentrations in streams of tropical, humid headwater catchments | 10.1038/srep32974 |
| 188 | Effect of land use and land cover changes on water quality in the Nawuni Catchment of the White Volta Basin, Northern Region, Ghana | 10.1007/s13201-020-01272-6 |
| 189 | Effect of streambed bacteria release on E. coli concentrations: Monitoring and modeling with the modified SWAT | 10.1016/j.ecolmodel.2010.03.005 |
| 190 | Effects of agricultural and urban land cover on New Zealand's estuarine water quality | 10.1080/00288330.2020.1729819 |
| 191 | Effects of agricultural land cover on water quality at the watershed scale in the Lower Kaskaskia River watershed | NA |
| 192 | EFFECTS OF AGRICULTURAL MANAGEMENT, LAND USE, AND WATERSHED SCALE ON E-COLI CONCENTRATIONS IN RUNOFF AND STREAMFLOW | NA |
| 193 | Effects of agricultural management, land use, and watershed scale on E. coli concentrations in runoff and streamflow | NA |
| 194 | Effects of anthropic actions and forest areas on a neotropical aquatic ecosystem | 10.1016/j.scitotenv.2019.07.122 |
| 195 | Effects of bathing intensity, rainfall events, and location on the recreational water quality of stream pools in southern Ecuador | 10.1016/j.chemosphere.2019.125442 |
| 196 | Effects of changing land use on the microbial water quality of tidal creeks | 10.1016/j.marpolbul.2008.08.019 |
| 197 | Effects of future climate and land use scenarios on riverine source water quality | 10.1016/j.scitotenv.2014.06.087 |
| 198 | Effects of human activities on rivers located in protected areas of the Atlantic Forest | 10.1590/S2179-975X2014000100008 |
| 199 | Effects of hurricanes, land use, and water management on nutrient and microbial pollution: St. Lucie Estuary, Southeast Florida | 10.2112/JCOASTRES-D-12-00070.1 |
| 200 | Effects of hydrological regime and land use on in-stream Escherichia coli concentration in the Mekong basin, Lao PDR | 10.1038/s41598-021-82891-0 |
| 201 | Effects of land use and land cover changes on water quality in the uMngeni river catchment, South Africa | 10.1016/j.pce.2018.03.013 |
| 202 | Effects of land use and land cover on water quality of low-order streams in Southeastern Brazil: Watershed versus riparian zone | 10.1016/j.catena.2018.04.027 |
| 203 | Effects of Land Use Changes on Water Quality and Flooding in Upper Nan River, Thailand | 10.12982/CMJS.2024.008 |
| 204 | Effects of land use in the Ohio River basin on the distribution of coliform and antibiotic resistant bacteria in the Ohio River | NA |
| 205 | Effects of land use on the number of coliform bacteria in boyong river, sleman | 10.1088/1755-1315/1089/1/012075 |
| 206 | Effects of land uses on fecal indicator bacteria in the water and soil of a tropical watershed | 10.1264/jsme2.ME11115 |
| 207 | Effects of Land-Use Change on Benthic Macroinvertebrates in the Upper Reaches of the Apies-Pienaar Catchment | NA |
| 208 | Effects of low-impact-development (LID) practices on streamflow, runoff quantity, and runoff quality in the Ipswich River Basin, Massachusetts: A summary of field and modeling studies | NA |
| 209 | Effects of shelter belts on fence-line pacing of deer and associated impacts on water and soil quality | 10.1111/j.1475-2743.2006.00024.x |
| 210 | Effects of urbanisation on the quality of the urban runoff for Delhi watershed | 10.1080/15730620701780348 |
| 211 | Effects of urbanization on water quality and hydrology in the Lower Kaskaskia River Watershed in southern Illinois, United States | NA |
| 212 | Efficacy of Bacteroides measurements for reducing the statistical uncertainty associated with hydrologic flow and fecal loads in a mixed use watershed | 10.2134/jeq2006.0496 |
| 213 | Elucidating the effects of land cover and usage on background Escherichia coli sources in edge-of-field runoff | 10.2134/jeq2019.02.0051 |
| 214 | Enhancing Britain’s Rivers: An Interdisciplinary Analysis of Selected Issues Arising from Implementation of the Water Framework Directive | NA |
| 215 | Entire catchment and buffer zone approaches to modeling linkage between river water quality and land cover - A case study of Yamaguchi Prefecture, Japan | 10.1007/s11769-008-0085-6 |
| 216 | Environmental analysis of groundwater in Mecosta County, Michigan | 10.1007/s10661-007-9608-3 |
| 217 | Environmental DNA clarifies impacts of combined sewer overflows on the bacteriology of an urban river and resulting risks to public health | 10.1016/j.scitotenv.2023.164282 |
| 218 | Environmental factors controlling contamination of alternative water supply points in the Lefock semi-urban watershed, Cameroon Western Highlands | 10.1007/s12665-022-10699-w |
| 219 | Environmental fragility as an indicator of the risk of contamination by human action in watersheds used for public supply in western Parana, Brazil | 10.1007/s12665-022-10619-y |
| 220 | Environmental Impacts on Enterococcus in Shem Creek, South Carolina, and Characterization of Changing Land Uses | NA |
| 221 | Environmental risk factors in the incidence of Johnes disease | 10.3109/1040841X.2013.867830 |
| 222 | Environmental variables likely influence the periphytic diatom community in a subtropical lotic environment | 10.1016/j.limno.2019.125718 |
| 223 | Escherichia coli loading at or near base flow in a mixed-use watershed | 10.2134/jeq2006.0243 |
| 224 | Escherichia coli Reduction by Bivalves in an Impaired River Impacted by Agricultural Land Use | 10.1021/acs.est.6b03043 |
| 225 | Estimating daily potential E. coli loads in rural Texas watersheds using Spatially Explicit Load Enrichment Calculation Tool (SELECT) | 10.21423/twj.v3i1.6164 |
| 226 | Estimating Potential E. coli Sources in a Watershed Using Spatially Explicit Modeling Techniques | 10.1111/j.1752-1688.2012.00649.x |
| 227 | Estrogenic activity in the environment: Municipal wastewater effluent, river, ponds, and wetlands | 10.2134/jeq2004.0464 |
| 228 | Estuarine habitat quality reflects urbanization at large spatial scales in South Carolina's coastal zone | 10.1016/j.scitotenv.2007.09.036 |
| 229 | Evaluating land use impacts on water quality: perspectives for watershed management | 10.1007/s40899-023-00968-2 |
| 230 | Evaluating the impacts of coastal development on the sinuosity and water quality of tidal creek headwaters in the southeast | NA |
| 231 | Evaluating the Impacts of Environmental and Anthropogenic Factors on Water Quality in the Bumbu River Watershed, Papua New Guinea | 10.3390/w15030489 |
| 232 | Evaluating the influence of septic systems and watershed characteristics on stream faecal pollution in suburban watersheds in Georgia, USA | 10.1111/jam.12614 |
| 233 | Evaluation and assessment of water quality in Likangala River and its catchment area | 10.1016/j.pce.2011.07.070 |
| 234 | Evaluation of a multivariate analysis modeling approach identifying sources and patterns of nonpoint fecal pollution in a mixed use watershed | 10.1016/j.jenvman.2020.111413 |
| 235 | Evaluation of land use and water quality in an agricultural watershed in the USA indicates multiple sources of bacterial impairment | 10.1007/s10661-013-3340-y |
| 236 | Evaluation of recirculating sand filter in a cold climate | 10.2166/wst.2005.0375 |
| 237 | Evaluation of statistical models for predicting Escherichia coli particle attachment in fluvial systems | 10.1016/j.watres.2013.09.003 |
| 238 | Evaluation of the distribution of fecal indicator bacteria in a river system depending on different types of land use in the southern watershed of the Baltic Sea | 10.1007/s11356-015-4442-6 |
| 239 | Evaluation of the impacts of land use in water quality and the role of nature-based solutions: A citizen science-based study | 10.3390/su131910519 |
| 240 | Evaluation of the Water Quality Status and Pollution Load Carrying Capacity of Way Umpu River, Way Kanan District, Lampung Province, Indonesia, Based on Land Use | 10.1155/2023/2689879 |
| 241 | Evidence for coexistence of distinct escherichia coli populations in various aquatic environments and their survival in estuary water | 10.1128/AEM.00698-13 |
| 242 | Experimental and modelling evidence of splash effects on manure borne Escherichia coli washoff | 10.1007/s11356-021-13011-8 |
| 243 | Exploitation and Management of Natural Resources by Rural Communities in the Caete River Basin in northern Brazil | NA |
| 244 | F-specific RNA coliphages: Occurrence, types, and survival in natural waters | 10.1016/S0043-1354(01)00547-4 |
| 245 | Factors affecting surface-water and ground-water quality within tribal lands of eastern Nebraska | NA |
| 246 | Factors and Mechanisms Affecting Seasonal Changes in the Prevalence of Microbiological Indicators of Water Quality and Nutrient Concentrations in Waters of the Biaka River Catchment, Southern Poland | 10.1007/s11270-016-2931-y |
| 247 | Factors Associated With E. coli Levels in and Salmonella Contamination of Agricultural Water Differed Between North and South Florida Waterways | 10.3389/frwa.2021.750673 |
| 248 | Factors Influencing Safe Managed Decentralized Wastewater in Citarum Watershed | 10.14456/ea.2023.11 |
| 249 | Factors influencing the bacteriological water quality in mountainous surface and groundwaters | 10.1002/aheh.200300532 |
| 250 | Factors related to occurrence and distribution of selected bacterial and protozoan pathogens in Pennsylvania streams | 10.1016/j.watres.2012.10.006 |
| 251 | Faecal bacterial loads during flood events in Northwestern Mediterranean coastal rivers | 10.1016/j.jhydrol.2011.05.047 |
| 252 | Faecal contamination of water and sediment in the rivers of the Scheldt drainage network | 10.1007/s10661-011-1918-9 |
| 253 | Faecal indicator organism concentrations and catchment export coefficients in the UK | 10.1016/j.watres.2008.01.017 |
| 254 | Faecal pollution source identification in an urbanising catchment using antibiotic resistance profiling, discriminant analysis and partial least squares regression | 10.1016/j.watres.2008.12.017 |
| 255 | Faecal-indicator concentrations in waters draining lowland pastoral catchments in the UK: relationships with land use and farming practices | 10.1016/S0043-1354(01)00394-3 |
| 256 | Fecal bacteria in the waters of an upland area in Derbyshire, England: The influence of agricultural land use | 10.2134/jeq2000.00472425002900040032x |
| 257 | Fecal Coliform Concentrations in the Upper Cohansey River Watershed Predicted by Air Temperature, Discharge, and Land Use | NA |
| 258 | Fecal coliform export from four coastal North Carolina areas | 10.1111/j.1752-1688.2008.00185.x |
| 259 | Fecal coliform predictive model using genetic algorithm-based radial basis function neural networks (GA-RBFNNs) | 10.1007/s00521-019-04520-2 |
| 260 | Fecal coliform source assessment in a small, mixed land use watershed | 10.1111/j.1752-1688.2002.tb04369.x |
| 261 | Fecal contamination and high nutrient levels pollute the watersheds of Wujiang, China | 10.3390/w13040457 |
| 262 | Fecal contamination of water from a dog park and water potential changes affecting bacterial survival | NA |
| 263 | Fecal indicator bacteria removal by river networks | NA |
| 264 | Fecal indicator concentrations of surface runoff in rural Watersheds, Korea | 10.5004/dwt.2010.1892 |
| 265 | Fecal indicators and antibiotic resistance genes exhibit diurnal trends in the Chattahoochee River: Implications for water quality monitoring | 10.3389/fmicb.2022.1029176 |
| 266 | Fecal pollution source characterization in the surface waters of recharge and contributing zones of a karst aquifer using general and host-associated fecal genetic markers | 10.1039/d2em00418f |
| 267 | Fecal pollution source tracking toolbox for identification, evaluation and characterization of fecal contamination in receiving urban surface waters and groundwater | 10.1016/j.scitotenv.2015.07.155 |
| 268 | Fecal sterol and runoff analysis for nonpoint source tracking | 10.2134/jeq2015.06.0312 |
| 269 | Field and modelling studies of Escherichia coli loads in tropical streams of montane agro-ecosystems | 10.1016/j.jher.2015.03.003 |
| 270 | Find, Inform, and Test (FIT): A Spatial Modeling Framework to Estimate Contributions of Spatially Distributed Sources to Microbial Contaminants in the Environment | NA |
| 271 | Fine-scale genetic structure in an eastern Alpine black grouse Tetrao tetrix metapopulation | 10.1111/jav.01681 |
| 272 | Fish assemblages and water quality in pampean streams (Argentina) along an urbanization gradient | 10.1007/s10750-021-04657-z |
| 273 | Flow regulation by dams impacts more than land use on water quality and benthic communities in high-gradient streams in a semi-arid region | 10.1016/j.scitotenv.2023.163468 |
| 274 | Food web transfer of plastics to an apex riverine predator | 10.1111/gcb.15139 |
| 275 | Fourier Landscape Pattern Indices for Predicting South Carolina Watershed Fecal Coliform | 10.1515/jlecol-2017-0007 |
| 276 | Freshwater salinization increases survival of Escherichia coli and risk of bacterial impairment | 10.1016/j.watres.2021.116812 |
| 277 | Generic modelling of faecal indicator organism concentrations in the UK | 10.3390/w3020682 |
| 278 | Genetic fecal source identification in urban streams impacted by municipal separate storm sewer system discharges | 10.1371/journal.pone.0278548 |
| 279 | Genome-wide single nucleotide polymorphism (SNP) markers from fecal samples reveal anthropogenic impacts on connectivity: case of a small carnivore in the central Indian landscape | 10.1111/acv.12770 |
| 280 | Geo-environmental impacts of hydrogeological setting and anthropogenic activities on water quality in the Quaternary aquifer southeast of the Nile Delta, Egypt | 10.1016/j.jafrearsci.2020.103947 |
| 281 | Geochemical characterization and health risk assessment in two diversified environmental settings (Southern Italy) | 10.1007/s10653-021-00930-1 |
| 282 | Geographic information of helminthiasis in Thailand | NA |
| 283 | Geographic setting influences Great Lakes beach microbiological water quality | 10.1021/es402299a |
| 284 | Geospatial Tools to Inform Land Use Change Decisions: From Local Installation to Watershed Scale | NA |
| 285 | Geostatistical Prediction of Microbial Water Quality Throughout a Stream Network Using Meteorology, Land Cover, and Spatiotemporal Autocorrelation | 10.1021/acs.est.8b01178 |
| 286 | GIS and artificial neural network-based water quality model for a stream network in the upper green river basin, Kentucky, USA | 10.1061/(ASCE)EE.1943-7870.0000801 |
| 287 | Grazing intensity is a poor indicator of waterborne Escherichia coli O157 activity | 10.1016/j.anaerobe.2011.04.021 |
| 288 | Groundwater and factors affecting its quality: Examples from the Rovaniemi district of Northern Finland and western Nicaragua | NA |
| 289 | Groupwise modeling study of bacterially impaired watersheds in Texas: Clustering analysis | 10.1111/j.1752-1688.2006.tb04511.x |
| 290 | Growing season surface water loading of fecal indicator organisms within a rural watershed | 10.1016/j.watres.2008.12.006 |
| 291 | Habitat and host factors associated with liver fluke (Fasciola hepatica) diagnoses in wild red deer (Cervus elaphus) in the Scottish Highlands | 10.1186/s13071-019-3782-3 |
| 292 | Habitat selection of endangered Amami rabbits on Tokuno-Shima Island in Japan as assessed by counting fecal pellet groups on roads | 10.1163/15707563-bja10109 |
| 293 | Health risk assessment related to waterborne pathogens from the river to the tap | 10.3390/ijerph120302967 |
| 294 | Heavy metals in the habitat and throughout the food chain of the Neotropical otter, Lontra longicaudis, in protected Mexican wetlands | 10.1007/s10661-012-2623-z |
| 295 | High spatial resolution landscape indicators show promise in explaining water quality in urban streams | 10.1016/j.ecolind.2019.03.013 |
| 296 | How can we improve understanding of faecal indicator dynamics in karst systems under changing climatic, population, and land use stressors? - Research opportunities in SW China | 10.1016/j.scitotenv.2018.07.292 |
| 297 | How do different modalities of land use practices impact the environmental features and macroinvertebrates? An assessment of mountain streams from Patagonia, Argentina | 10.1016/j.envadv.2024.100511 |
| 298 | HSPF modeling of nonpoint sources in Tickfaw River watershed | NA |
| 299 | HuBac and nifH source tracking markers display a relationship to land use but not rainfall | 10.1016/j.watres.2012.09.016 |
| 300 | Human and animal microbial source tracking in a tropical river with multiple land use activities | 10.1016/j.ijheh.2019.01.005 |
| 301 | Human development is linked to multiple water body impairments along the California coast | 10.1007/BF02786537 |
| 302 | Human Fecal Contamination Corresponds to Changes in the Freshwater Bacterial Communities of a Large River Basin | 10.1128/Spectrum.01200-21 |
| 303 | Human Fecal Pollution Monitoring and Microbial Risk Assessment for Water Reuse Potential in a Coastal Industrial-Residential Mixed-Use Watershed | 10.3389/fmicb.2021.647602 |
| 304 | Human health risk assessment and environmental distribution of trace elements, glyphosate, fecal coliform and total coliform in Atlantic Rainforest mountain rivers (South America) | 10.1016/j.microc.2015.05.004 |
| 305 | Human source identification by using a human-associated Escherichia coli genetic marker in the Mae Klong River, Thailand | 10.2166/wh.2022.296 |
| 306 | Human-Dominated Land Use Change in a Phosphate Mining Area and Its Impact on the Water Environment | 10.3390/w14071074 |
| 307 | Humans and Hoofed Livestock Are the Main Sources of Fecal Contamination of Rivers Used for Crop Irrigation: A Microbial Source Tracking Approach | 10.3389/fmicb.2022.768527 |
| 308 | Hydrochemical evidence of the depth of penetration of anthropogenic recharge in sandstone aquifers underlying two mature cities in the UK | 10.1016/j.apgeochem.2006.06.015 |
| 309 | Hydrochemical indices as a proxy for assessing land-use impacts on water resources: a sustainable management perspective and case study of Can Tho City, Vietnam | 10.1007/s11069-023-05957-4 |
| 310 | Hydrochemistry of shallow groundwater and springs used for potable supply in Southern Brazil | 10.1007/s12665-018-7254-4 |
| 311 | Hydrogeomorphological controls on groundwater quality in the Rattaphum Catchment (Songkhla Lake Basin), Thailand | 10.1007/s11267-005-7410-1 |
| 312 | Hydrological Regime and Water Shortage as Drivers of the Seasonal Incidence of Diarrheal Diseases in a Tropical Montane Environment | 10.1371/journal.pntd.0005195 |
| 313 | Hydrology, water quality, and channel morphology across an urban-rural land use gradient in western Georgia, United States of America | NA |
| 314 | Hydrometeorology and flood pulse dynamics drive diarrheal disease outbreaks and increase vulnerability to climate change in surface-water-dependent populations: A retrospective analysis | 10.1371/journal.pmed.1002688 |
| 315 | Identification and remediation of microbial contaminants in the headwaters of an agricultural watershed | NA |
| 316 | Identifying fecal pollution sources using 3M Petrifilm count plates and antibiotic resistance analysis in the Horse Creek Watershed in Aiken County, SC (USA) | 10.1007/s10661-014-3999-8 |
| 317 | Identifying sources of fecal pollution in the Colville River using library-independent genetic markers | 10.3955/0029-344X-82.2.120 |
| 318 | Impact of changes of land use on water quality, from tropical forest to anthropogenic occupation: A multivariate approach | 10.3390/w10111518 |
| 319 | Impact of Construction and Functioning of a Newly Built Ski Slope on the Quality of Nearby Stream Water | 10.3390/app13020763 |
| 320 | Impact of Covid-19 Lockdown on Availability of Drinking Water in the Arsenic-Affected Ganges River Basin | 10.3390/ijerph18062832 |
| 321 | Impact of home industries on water quality in a tributary of the Marimba River, Harare: Implications for urban water management | 10.1016/j.pce.2003.08.034 |
| 322 | Impact of land use and urban runoff on the contamination of the Sarno River basin in Southwestern Italy | 10.1023/A:1011908019933 |
| 323 | Impact of land use on fecal coliform levels in surface waters of Fairfax County, Virginia | NA |
| 324 | Impact of land use on the faecal microbial quality of hill-country streams | 10.1080/00288330.2004.9517284 |
| 325 | Impact of land use on water quality in the Likangala catchment, southern Malawi | 10.2989/16085914.2015.1077777 |
| 326 | Impact of land uses, drought, flood, wildfire, and cascading events on water quality and microbial communities: A review and analysis | 10.1016/j.jhydrol.2020.125707 |
| 327 | Impact of land-use/land-cover dynamics on water quality in the Upper Lilongwe River basin, Malawi | 10.1007/s42108-021-00125-5 |
| 328 | Spatio-seasonal variation of water quality influenced by land use and land cover in Lake Muhazi | 10.1007/s11356-020-12285-8 |
| 329 | Impact of urbanization and agriculture on the occurrence of bacterial pathogens and stx genes in coastal waterbodies of central California | 10.1016/j.watres.2010.11.032 |
| 330 | Impact of urbanization on the water quality of the Uberaba River and tributaries. | NA |
| 331 | Impact on water quality of land uses along Thamalakane-Boteti River: An outlet of the Okavango Delta | 10.1016/j.pce.2008.06.035 |
| 332 | Impacts of droughts and heatwaves on river water quality worldwide | 10.1016/j.jhydrol.2023.130590 |
| 333 | Impacts of global change on the concentrations and dilution of combined sewer overflows in a drinking water source | 10.1016/j.scitotenv.2014.11.059 |
| 334 | Impacts of Land-Based Recreation on Water Quality | 10.3375/043.040.0209 |
| 335 | Impacts of manure management practices on stream microbial loading into Conesus Lake, NY | 10.1016/j.jglr.2009.01.002 |
| 336 | Impacts of tropical forest cover on water quality in agricultural watersheds in southeastern Brazil | 10.1016/j.ecolind.2018.06.030 |
| 337 | Impacts of urbanization on the prevalence of antibiotic-resistant Escherichia coli in the Chaophraya River and its tributaries | 10.2166/wst.2015.502 |
| 338 | Implementation and integration of microbial source tracking in a river watershed monitoring plan | 10.1016/j.scitotenv.2020.139573 |
| 339 | Implementing landscape indices to predict stream water quality in an agricultural setting: An assessment of the Lake and River Enhancement (LARE) protocol in the Mississinewa River watershed, East-Central Indiana | 10.1016/j.ecolind.2010.03.007 |
| 340 | Implications of demographic changes and land transformations on surface water quality of rural and urban subbasins of Upper Bhima River basin, Maharashtra, India | 10.1007/s10668-018-0187-y |
| 341 | Importance of land use factors in the prediction of water quality of the Upper Green River watershed, Kentucky, USA, using random forest | 10.1007/s10668-023-03630-1 |
| 342 | Improved management of farm dams increases vegetation cover, water quality, and macroinvertebrate biodiversity | 10.1002/ece3.8636 |
| 343 | Improving the economic and environmental performance of a New Zealand hill country farm catchment: 3. Short-term outcomes of land-use change | 10.1080/00288230809510444 |
| 344 | In-stream Escherichia coli modeling using high-temporal-resolution data with deep learning and process-based models | 10.5194/hess-25-6185-2021 |
| 345 | Inadequate riparian zone use directly decreases water quality of a low-order urban stream in Southern Brazil | 10.4136/ambi-agua.2451 |
| 346 | Incorporating water quality into land use scenario analysis with random forest models | 10.1177/23998083221138842 |
| 347 | Index of state trophic river basins Ribeirao the big tank, Guarulhos (SP): Comparative analysis of rural areas and urban | NA |
| 348 | Indicator bacteria and associated water quality constituents in stormwater and snowmelt from four urban catchments | 10.1016/j.jhydrol.2016.05.006 |
| 349 | Influence of climate change, tidal mixing, and watershed urbanization on historical water quality in Newport Bay, a saltwater wetland and tidal embayment in southern California | 10.1021/es0504789 |
| 350 | Influence of climate variables on the concentration of Escherichia coli in the Rhine, Meuse, and Drentse Aa during 1985-2010 | 10.1007/s10113-013-0492-9 |
| 351 | Influence of intensive agriculture on benthic macroinvertebrate assemblages and water quality in the aconcagua river basin (Central chile) | 10.3390/w13040492 |
| 352 | Influence of land use and nutrient flux on metabolic activity of E. coli O157 in river water | 10.1007/s11270-012-1090-z |
| 353 | Influence of Land Use Land Cover on River Water Quality in Rural North Wales, UK | 10.1111/1752-1688.12904 |
| 354 | Influence of land use on the chemistry and microbial abundance in groundwater | NA |
| 355 | Influence of land use on water quality in a tropical landscape: a multi-scale analysis | 10.1007/s10980-011-9642-y |
| 356 | Influence of Physicochemical Factors on Bacterial Communities Along the Lower Mekong River Assessed by Illumina Next-Generation Sequencing | 10.1007/s11270-018-3973-0 |
| 357 | Influence of rainy season and land use on drinking water quality in a karst landscape, State of Yucatan, Mexico | 10.1016/j.apgeochem.2018.09.020 |
| 358 | INFLUENCE OF WATERSHED LAND USE ON WATER QUALITY IN THE STATE OF SANTA CATARINA, BRAZIL | 10.24057/2071-9388-2021-015 |
| 359 | Influences of external factors on Escherichia coli distribution, concentration, sources, and fate in secondary environments | NA |
| 360 | Influential factors in surface water quality in catchments within the pampa biome with different land use | 10.1590/0100-67622015000600017 |
| 361 | Inland lake indicator bacteria: Long-term impervious surface and weather influences and a predictive bayesian model | 10.1080/07438141.2012.716500 |
| 362 | Innate immunity and stress physiology of eastern hellbenders (Cryptobranchus alleganiensis) from two stream reaches with differing habitat quality | 10.1016/j.ygcen.2011.08.006 |
| 363 | Inputs of nutrients and fecal bacteria to freshwaters from irrigated agriculture: case studies in Australia and New Zealand | 10.1007/s00267-011-9644-1 |
| 364 | Inside or Outside: Quantifying Extrapolation Across River Networks | 10.1029/2018WR023378 |
| 365 | Instream coliform gradients in the Holtemme, a small headwater stream in the Elbe River Basin, Northern Germany | 10.1007/s11707-017-0648-x |
| 366 | Integral assessment of pollution in the Suquia River (Cordoba, Argentina) as a contribution to lotic ecosystem restoration programs | 10.1016/j.scitotenv.2011.08.037 |
| 367 | Integrated approach for quantitative estimation of particulate organic carbon sources in a complex river system | 10.1016/j.watres.2021.117194 |
| 368 | Integrating environmental and socio-economic indicators of a linked catchment-coastal system using variable environmental intensity | 10.1007/s00267-010-9539-6 |
| 369 | Integration of remote sensing data and in situ measurements to monitor the water quality of the Ismailia Canal, Nile Delta, Egypt | 10.1007/s10653-019-00466-5 |
| 370 | Integrative Survey of 68 Non-overlapping Upstate New York Watersheds Reveals Stream Features Associated With Aquatic Fecal Contamination | 10.3389/fmicb.2021.684533 |
| 371 | Intra-event variability of bacterial composition in stormwater runoff from mixed land use and land cover catchment | 10.12989/mwt.2019.10.1.029 |
| 372 | Investigating Escherichia coli in a Mixed Land-Use Watershed in West Virginia, USA | NA |
| 373 | Investigating Landscape-stream Water Quality Relationships and Stream Water Quality Preservation Strategies in the Texas Gulf Region Using a Hybrid of Machine Learning and Hydrological Modeling Approach | NA |
| 374 | Investigating the Relationship between Surface Water Pollution and Onsite Wastewater Treatment Systems | 10.1021/acs.est.2c09590 |
| 375 | Investigation of relationships between fecal contamination, cattle grazing, human recreation, and microbial source tracking markers in a mixed-land-use rangeland watershed | 10.1016/j.watres.2021.116921 |
| 376 | Irrigation Water Quality of a Community Garden Complex in the State of Piauí, Northeastern Brazil | 10.1007/s11270-020-04486-1 |
| 377 | Isolating the impact of septic systems on fecal pollution in streams of suburban watersheds in Georgia, United States | 10.1016/j.watres.2016.11.007 |
| 378 | Jump run creek shellfish restoration project | NA |
| 379 | Lake Tuscaloosa and the North River: An analysis of, and plans to improve, water quality | NA |
| 380 | Land cover impacts on stream nutrients and fecal coliform in the lower Piedmont of West Georgia | 10.1016/j.jhydrol.2006.05.031 |
| 381 | Land use and environmental variables influence tetracycline-resistant bacteria occurrence in southeastern coastal plain streams | 10.2134/jeq2019.03.0139 |
| 382 | Land use and hydroclimatic influences on Faecal Indicator Organisms in two large Scottish catchments: towards land use-based models as screening tools | 10.1016/j.scitotenv.2011.11.090 |
| 383 | Land use and land cover changes in Zezere watershed (Portugal)--Water quality implications | 10.1016/j.scitotenv.2015.04.092 |
| 384 | Land use and monitoring of water resources of Ipe stream, Ilha Solteira, SP | 10.1590/S1415-43662013000100009 |
| 385 | Land use and water quality in a rural cloud forest region (Intag, Ecuador) | 10.1002/rra.2634 |
| 386 | Land use and water quality in Guangzhou, China: A survey of ecological and social vulnerability in four urban units of the rapidly developing megacity | NA |
| 387 | Land use and water quality relationships in the Lower Little Bow River watershed, Alberta, Canada | 10.2166/wqrj.2003.037 |
| 388 | Land use as a critical determinant of faecal and antimicrobial resistance gene pollution in riverine systems | 10.1016/j.scitotenv.2023.162052 |
| 389 | Land use effects on water quality in the urban agglomeration of Cuiaba and Varzea Grande, Mato Grosso State, central Brazil | 10.1080/1573062X.2010.484496 |
| 390 | Land use impact on the water quality of large tropical river: Mun River Basin, Thailand | 10.1007/s10661-019-7779-3 |
| 391 | Land use impacts on parasitic infection: a cross-sectional epidemiological study on the role of irrigated agriculture in schistosome infection in a dammed landscape | 10.1186/s40249-021-00816-5 |
| 392 | Land Use Land Cover Changes in Detection of Water Quality: A Study Based on Remote Sensing and Multivariate Statistics | 10.1155/2017/7515130 |
| 393 | LAND USE PRACTICES AND ELEVATED LEVELS OF ESCHERICHIA COLI IN THE COOSAWATTEE RIVER, GEORGIA | NA |
| 394 | Land Use, Weather, and Water Quality Factors Associated With Fecal Contamination of Northeastern Streams That Span an Urban-Rural Gradient | 10.3389/frwa.2021.741676 |
| 395 | Land-use & Water Quality in the Headwaters of the Alafia River Watershed | NA |
| 396 | Land-use change caused microbial pollution in a karst underground river, Chongqing, China | 10.1007/s12665-016-5530-8 |
| 397 | Land-Use Impact on Water Quality of the Opak Sub-Watershed, Yogyakarta, Indonesia | 10.3390/su14074346 |
| 398 | Land-use impacts and water quality targets in the intensive dairying catchment of the Toenepi Stream, New Zealand | 10.1080/00288330.2006.9517407 |
| 399 | Land-use related changes to sedimentary organic matter in tidal creeks of the northern Gulf of Mexico | 10.1002/lno.10453 |
| 400 | Land-use-mediated Escherichia coli concentrations in a contemporary Appalachian watershed | 10.1007/s12665-018-7948-7 |
| 401 | Land-use/land-cover and water qualty in the Cape Fear river basin, North Carolina: Spatial-temporal relationships | NA |
| 402 | Landscape and seasonal factors influence Salmonella and Campylobacter prevalence in a rural mixed use watershed | 10.1016/j.watres.2013.07.028 |
| 403 | Landscape drivers and social dynamics shaping microbial contamination risk in three Maya communities in Southern Belize, Central America | 10.3390/w10111678 |
| 404 | Landscape genetics of northern bobwhite and swamp rabbits in Illinois | NA |
| 405 | Landscape-Scale Factors Affecting the Prevalence of Escherichia coli in Surface Soil Include Land Cover Type, Edge Interactions, and Soil pH | 10.1128/AEM.02714-17 |
| 406 | Large-scale implementation of standardized quantitative real-time PCR fecal source identification procedures in the Tillamook Bay Watershed | 10.1371/journal.pone.0216827 |
| 407 | Learning hierarchical Bayesian networks to assess the interaction effects of controlling factors on spatiotemporal patterns of fecal pollution in streams | 10.1016/j.scitotenv.2021.152520 |
| 408 | Level and transport pattern of faecal coliform bacteria from tropical urban catchments | 10.2166/wst.2013.048 |
| 409 | Levels and patterns of fecal indicator bacteria in stormwater runoff from homogenous land use sites and urban watersheds | 10.2166/wh.2010.056 |
| 410 | Linkages between tidal creek ecosystems and the landscape and demographic attributes of their watersheds | 10.1016/S0022-0981(03)00357-5 |
| 411 | Linking fecal bacteria in rivers to landscape, geochemical, and hydrologic factors and sources at the basin scale | 10.1073/pnas.1415836112 |
| 412 | Linking land-use type and stream water quality using spatial data of fecal indicator bacteria and heavy metals in the Yeongsan river basin | 10.1016/j.watres.2010.05.009 |
| 413 | Linking the uptake of best management practices on dairy farms to catchment water quality improvement over a 20-year period | 10.1016/j.scitotenv.2023.164963 |
| 414 | Macroinvertebrate indices versus microbial fecal pollution characteristics for water quality monitoring reveals contrasting results for an Ethiopian river | 10.1016/j.ecolind.2019.105733 |
| 415 | Managed aquifer recharge implementation criteria to achieve water sustainability | 10.1016/j.scitotenv.2021.144992 |
| 416 | Mapping of critical source areas for diffuse fecal bacterial pollution in extensively grazed watersheds | 10.1016/j.watres.2010.04.039 |
| 417 | Mapping of Escherichia coli Sources Connected to Waterways in the Ruamahanga Catchment, New Zealand | 10.1021/acs.est.5b05167 |
| 418 | Mapping the potential risk of Escherichia coli leaching through soils of the Waikato River catchment, New Zealand | 10.1071/SR18228 |
| 419 | Mapping the spatial distribution of the rumen fluke calicophoron daubneyi in a mediterranean area | 10.3390/pathogens10091122 |
| 420 | Mapping the spatial distribution of water quality as a function of land use and occupation and rainfall in the Para River Basin, MG | 10.1590/S1413-415220200369 |
| 421 | Measurement and modeling of denitrification in sand-bed streams under various land uses | 10.2134/jeq2013.06.0249 |
| 422 | Microarray assessment of virulence, antibiotic, and heavy metal resistance in an agricultural watershed creek | 10.2134/jeq2011.0172 |
| 423 | Microbial Find, Inform, and Test Model for Identifying Spatially Distributed Contamination Sources: Framework Foundation and Demonstration of Ruminant Bacteroides Abundance in River Sediments | 10.1021/acs.est.1c01602 |
| 424 | Microbial pollution source identification in rural / urban mixed watersheds | NA |
| 425 | Microbial source tracking (MST) in Chattahoochee River National Recreasion Area: Seasonal and precipitation in MST marker concentrations, and associations with e. coli levels, pathogenic marker presence, and land use | 10.1016/j.watres.2019.115435 |
| 426 | Microbial source tracking to elucidate the impact of land-use and physiochemical water quality on fecal contamination in a mixed land-use watershed | 10.1016/j.scitotenv.2023.162181 |
| 427 | Microbial source tracking: a forensic technique for microbial source identification? | 10.1039/b617059e |
| 428 | Microbial source-tracking reveals origins of fecal contamination in a recoveringwatershed | 10.3390/w11102162 |
| 429 | Microbial water pollution: a screening tool for initial catchment-scale assessment and source apportionment | 10.1016/j.scitotenv.2009.07.033 |
| 430 | Microbial Water Quality and Influences of Fecal Accumulation from a Dog Exercise Area | NA |
| 431 | Microbiological quality assessment of watershed associated with animal-based agriculture in Santa Catarina, Brazil | 10.1007/s11270-009-0254-y |
| 432 | Mitochondrial activity in fern spores of Cyathea costaricensis as an indicator of the impact of land use and water quality in rivers running through cloud forests | 10.1016/j.chemosphere.2017.09.094 |
| 433 | Model-based analysis of the potential of macroinvertebrates as indicators for microbial pathogens in rivers | 10.3390/w10040375 |
| 434 | Modeling bacteria fate and transport in watersheds to support TMDLs | NA |
| 435 | Modeling spatiotemporal bacterial variability with meteorological and watershed land-use characteristics | 10.1016/j.watres.2016.05.024 |
| 436 | Modeling the dispersion of E. coli in waterbodies due to urban sources: A spatial approach | 10.3390/w9090665 |
| 437 | Modeling the Impact of Land Use Change on Basin-scale Transfer of Fecal Indicator Bacteria: SWAT Model Performance | 10.2134/jeq2017.11.0456 |
| 438 | Modeling the relationship between land use and surface water quality | 10.1006/jema.2002.0593 |
| 439 | Modelling faecal bacteria pathways in receiving waters | 10.1680/maen.2007.160.4.143 |
| 440 | Modelling faecal indicator concentrations in large rural catchments using land use and topographic data | 10.1046/j.1365-2672.2003.01877.x |
| 441 | Modelling microbiological water quality in the Seine river drainage network: Past, present and future situations | 10.5194/hess-11-1581-2007 |
| 442 | Modelling of faecal indicator bacteria (FIB) in the Red River basin (Vietnam) | 10.1007/s10661-016-5528-4 |
| 443 | Modelling the hydrologic effects of land-use and climate changes | 10.1504/IJRAM.2006.009543 |
| 444 | Modelling the impact of future socio-economic and climate change scenarios on river microbial water quality | 10.1016/j.ijheh.2017.11.006 |
| 445 | Modelling the seasonal impacts of a wastewater treatment plant on water quality in a Mediterranean stream using microbial indicators | 10.1016/j.jenvman.2020.110220 |
| 446 | Models of total and presumed wildlife sources of fecal coliform bacteria in coastal ponds | 10.1016/j.jenvman.2005.12.010 |
| 447 | Molecular tracers of soot and sewage contamination in streams supplying New York City drinking water | 10.1899/0887-3593(2006)025[0928:MTOSAS]2.0.CO;2 |
| 448 | Monitoring and evaluation of the water quality of the Lower Neches River, Texas, USA | 10.1016/j.wse.2023.10.002 |
| 449 | Monitoring and predicting the fecal indicator bacteria concentrations from agricultural, mixed land use and urban stormwater runoff | 10.1016/j.scitotenv.2016.01.026 |
| 450 | Monitoring bacterial indicators of water quality in a tidally influenced delta: A Sisyphean pursuit | 10.1016/j.scitotenv.2016.10.179 |
| 451 | Monitoring of Spunky Bottoms restored wetland in Southern Illinois for biotic and abiotic pollution indicators | NA |
| 452 | Motueka River plume facilitates transport of ruminant faecal contaminants into shellfish growing waters, Tasman Bay, New Zealand | 10.1080/00288330.2011.587822 |
| 453 | Multi-scale landscape factors influencing stream water quality in the state of Oregon | 10.1007/s10661-008-0489-x |
| 454 | Multi-year microbial source tracking study characterizing fecal contamination in an urban watershed | 10.2175/106143016X14798353399412 |
| 455 | Multiple modes of water quality impairment by fecal contamination in a rapidly developing coastal area: southwest Brunswick County, North Carolina | 10.1007/s10661-015-5081-6 |
| 456 | Multiscale spatiotemporal variability of fecal indicator bacteria and associated particle size distributions in the sandy bottom sediments of a Pennsylvania creek | 10.1002/jeq2.20531 |
| 457 | Native forest cover safeguards stream water quality under a changing climate | 10.1002/eap.2414 |
| 458 | Next-generation sequencing reveals fecal contamination and potentially pathogenic bacteria in a major inflow river of Taihu Lake | 10.1016/j.envpol.2019.113108 |
| 459 | Non-point source fecal contamination from aging wastewater infrastructure is a primary driver of antibiotic resistance in surface waters | 10.1016/j.watres.2022.118853 |
| 460 | Nonpoint source reduction to the nearshore zone via watershed management practices: Nutrient fluxes, fate, transport and biotic responses - Background and objectives | 10.1016/j.jglr.2008.08.002 |
| 461 | Occurrence and distribution of fecal indicator bacteria with respect to urban and rural land uses | NA |
| 462 | Occurrence and distribution of microbiological indicators in groundwater and stream water | 10.2175/106143000X137220 |
| 463 | Occurrence and distribution of viruses and picoplankton in tropical freshwater bodies determined by flow cytometry | 10.1016/j.watres.2018.11.022 |
| 464 | Occurrence of microbial indicators, pathogenic bacteria and viruses in tropical surface waters subject to contrasting land use | 10.1016/j.watres.2018.11.058 |
| 465 | Occurrence of traditional and alternative fecal indicators in tropical urban environments under different land use patterns | 10.1128/AEM.00287-18 |
| 466 | Optimization of the water quality monitoring network in a basin with intensive agriculture using artificial intelligence algorithms | 10.2166/ws.2023.336 |
| 467 | Participatory approach for more robust water resource management: Case study of the santa rosa sub-watershed of the Philippines | 10.3390/W12041172 |
| 468 | Particle-attached riverine bacteriome shifts in a pollutant-resistant and pathogenic community during a Mediterranean extreme storm event | 10.1016/j.scitotenv.2020.139047 |
| 469 | Pathogen transport and fate modeling in the Upper Salem River Watershed using SWAT model | 10.1016/j.jenvman.2014.12.042 |
| 470 | Patterns and drivers of fecal coliform exports in a typhoon-affected watershed: insights from 10-year observations and SWAT model | 10.1016/j.jclepro.2023.137044 |
| 471 | Patterns in water quality on Canadian shores of Lake Ontario: Correspondence with proximity to land and level of urbanization | 10.1016/j.jglr.2011.12.005 |
| 472 | Patterns of genetic diversity in African forest elephants living in a human-modified landscape in southwest Gabon | 10.1111/csp2.76 |
| 473 | Patterns of Host-Associated Fecal Indicators Driven by Hydrology, Precipitation, and Land Use Attributes in Great Lakes Watersheds | 10.1021/acs.est.8b01945 |
| 474 | Phosphorus and thermotolerant coliforms's loads in brazilian watersheds with limited data: Considerations on the integrated analysis of water quality and quantity | 10.1590/2318-0331.241920170137 |
| 475 | Physical, Chemical, and Microbial Quality of Floodwaters in Houston Following Hurricane Harvey | 10.1021/acs.est.9b00792 |
| 476 | Physico-chemical and biological characteristics of mountainous streams under different land uses of mid hills of Himachal Pradesh | NA |
| 477 | Physiographic Environment Classification: a Controlling Factor Classification of Landscape Susceptibility to Waterborne Contaminant Loss | 10.1007/s00267-024-01950-0 |
| 478 | Pine afforestation and stream health: a comparison of land-use in two soft rock catchments, East Cape, New Zealand | NA |
| 479 | Point and non-point microbial source pollution: A case study of Delhi | 10.1016/j.pce.2008.09.005 |
| 480 | Pollution in Qaraaoun lake, Central Lebanon | NA |
| 481 | Population dynamics and genetic variability of Escherichia coli in a mixed land-use watershed | 10.2166/wh.2009.105 |
| 482 | Population genetics and structure of the sumatran tiger | NA |
| 483 | Population growth, land use and land cover transformations, and water quality nexus in the Upper Ganga River basin | 10.5194/hess-22-4745-2018 |
| 484 | Potential Impacts of Climate and Land Use Change on the Water Quality of Ganga River around the Industrialized Kanpur Region | 10.1038/s41598-020-66171-x |
| 485 | Potential pollutant sources in a Choptank River (USA) subwatershed and the influence of land use and watershed characteristics | 10.1016/j.scitotenv.2012.03.056 |
| 486 | Potential sources of ammonium-nitrogen in the coastal groundwater determined from a combined analysis of nitrogen isotope, biological and geological parameters, and land use | 10.3390/w13010025 |
| 487 | Precipitation-Driven Anthropogenic Pollutant Fluctuations Within Standing Water Sources of the Edwards Aquifer Region, Texas | 10.1177/11786221221108213 |
| 488 | Predicting diffuse microbial pollution risk across catchments: The performance of SCIMAP and recommendations for future development | 10.1016/j.scitotenv.2017.07.186 |
| 489 | Predicting faecal indicator fluxes using digital land use data in the UK's sentinel Water Framework Directive catchment: the Ribble study | 10.1016/j.watres.2005.07.006 |
| 490 | Predicting Fecal Indicator Bacteria Using Spatial Stream Network Models in A Mixed-Land-Use Suburban Watershed in New Jersey, USA | 10.3390/ijerph20064743 |
| 491 | Predicting fecal indicator organism contamination in Oregon coastal streams | 10.1016/j.envpol.2015.08.025 |
| 492 | Predicting Gene Flow Corridors and Wildlife Health Using Landscape Genomics and Non-Invasive Metagenomic Monitoring: Investigation of Elk (Cervus canadensis) in the Greater Yellowstone Ecosystem | NA |
| 493 | Predicting in-stream water quality constituents at the watershed scale using machine learning | 10.1016/j.jconhyd.2022.104078 |
| 494 | Predicting microbial pollution concentrations in UK rivers in response to land use change | 10.1016/j.watres.2010.07.062 |
| 495 | Predicting stream water quality under different urban development pattern scenarios with an interpretable machine learning approach | 10.1016/j.scitotenv.2020.144057 |
| 496 | Predicting the fate and transport of E. COLI in two texas river basins using a spatially referenced regression model | 10.1111/j.1752-1688.2009.00337.x |
| 497 | Predictive Models May Complement or Provide an Alternative to Existing Strategies for Assessing the Enteric Pathogen Contamination Status of Northeastern Streams Used to Provide Water for Produce Production | 10.3389/fsufs.2020.561517 |
| 498 | Prevalence and seasonal dynamics of blaCTX-M antibiotic resistance genes and fecal indicator organisms in the lower Lahn River, Germany | 10.1371/journal.pone.0232289 |
| 499 | Prevalence of antibiotic resistance in the tropical rivers of Sri Lanka and India | 10.1016/j.envres.2020.109765 |
| 500 | Quality of Surface Water in Missouri, Water Year 2020 | 10.3133/dr1153 |
| 501 | Quality of Surface Water in Missouri, Water Year 2021 | 10.3133/dr1179 |
| 502 | Quantification of fecal coliform inputs to aquatic systems through soil leaching | 10.1016/j.watres.2003.10.022 |
| 503 | Quantification of human-associated fecal indicators reveal sewage from urban watersheds as a source of pollution to Lake Michigan | 10.1016/j.watres.2016.05.056 |
| 504 | Quantification of Microbial Source Tracking and Pathogenic Bacterial Markers in Water and Sediments of Tiaoxi River (Taihu Watershed) | 10.3389/fmicb.2019.00699 |
| 505 | Quantification of Poultry and Human Fecal Contamination in the Tidal Creeks of the Virginia Eastern Shore Using a Multifaceted eDNA Method | NA |
| 506 | Quantifying escherichia coli and suspended particulate matter concentrations in a mixed-land use appalachian watershed | 10.3390/w12020532 |
| 507 | Quantifying the contribution of riparian soils to the provision of ecosystem services | 10.1016/j.scitotenv.2017.12.179 |
| 508 | Quantifying the effect of overland flow on Escherichia coli pulses during floods: Use of a tracer-based approach in an erosion-prone tropical catchment | 10.1016/j.jhydrol.2020.125935 |
| 509 | Quantifying the variability in Escherichia coli (E. coli) throughout storm events at a karst spring in northwestern Arkansas, United States | 10.1007/s12665-015-4416-5 |
| 510 | Quantitative multi-year elucidation of fecal sources of waterborne pathogen contamination in the South Nation River basin using bacteroidales microbial source tracking markers | 10.1016/j.watres.2013.02.009 |
| 511 | Quantitative PCR-based detection of pathogenic Leptospira in Hawai'ian coastal streams | 10.2166/wh.2011.064 |
| 512 | Rainfall driven e. coli transfer to the stream conduit network observed through increasing spatial scales in mixed land use paddy farming karst terrain | 10.1016/j.wroa.2019.100038 |
| 513 | Reach specificity in sediment E. coli population turnover and interaction with waterborne populations | 10.1016/j.scitotenv.2014.06.145 |
| 514 | Real time characterization and modeling of Escherichia coli contamination in urban streams | NA |
| 515 | Real-time consequences of riparian cattle trampling for mobilization of sediment, nutrients and bacteria in a British lowland river | 10.1080/15715124.2017.1402778 |
| 516 | Recreational Disturbance of River Sediments During Base Flow Deteriorates Microbial Water Quality | 10.1007/s41742-023-00557-3 |
| 517 | Regional assessment of concentrations and sources of pharmaceutically active compounds, pesticides, nitrate, and E. coli in post-glacial aquifer environments (Canada) | 10.1016/j.scitotenv.2016.11.061 |
| 518 | Regional variations of bovine and porcine fecal pollution as a function of landscape, nutrient, and hydrological factors | 10.2134/jeq2017.11.0438 |
| 519 | Relating watershed characteristics to elevated stream Escherichia coli levels in agriculturally dominated landscapes: An Iowa case study | 10.3390/w9030154 |
| 520 | Relations of the groundwater quality and disorderly occupation in an Amazon low-income neighborhood developed over a former dump area, Santarem/PA, Brazil | 10.1007/s10668-017-0040-8 |
| 521 | Relationship between land use and surface water quality in a rapidly developing watershed in southeast Louisiana | NA |
| 522 | Relationship between land use and water quality in a watershed impacted by iron ore tailings and domestic sewage | 10.4136/ambi-agua.2383 |
| 523 | Relationships between intra-aggregate pore structures and distributions of Escherichia coli within soil macro-aggregates | 10.1016/j.apsoil.2012.10.001 |
| 524 | Relationships Between Land Use and Mercury Contamination in Twelve Tributaries of the Lake St. Francis Region of the St. Lawrence River near Cornwall, Ontario | NA |
| 525 | Relationships between Land Use Patterns and Water Quality in the Pong River Basin, Northeast Thailand | 10.14456/ea.2022.35 |
| 526 | Respective contributions of point and non-point sources of E. coli and enterococci in a large urbanized watershed (the Seine river, France) | 10.1016/j.jenvman.2006.01.011 |
| 527 | Responses of stream macroinvertebrate communities and water quality of five dairy farming streams following adoption of mitigation practices | 10.1080/00288330.2016.1269814 |
| 528 | Riparian buffers: Disrupting the transport of E. coli from rural catchments to streams | 10.1016/j.watres.2022.118897 |
| 529 | Riparian protection and on-farm best management practices for restoration of a lowland stream in an intensive dairy farming catchment: A case study | 10.1080/00288330909510042 |
| 530 | RIVER WATER QUALITY IN NEW ZEALAND: AN INTRODUCTION AND OVERVIEW | NA |
| 531 | River water quality modelling for river basin and water resources management | NA |
| 532 | Roadside ditches as conduits of fecal indicator organisms and sediment: implications for water quality management | 10.1016/j.jenvman.2013.05.021 |
| 533 | Role of free-ranging mammals in the deposition of Escherichia coli into a Texas floodplain | 10.1071/WR13082 |
| 534 | Sanitary analyses of runoff water a river | NA |
| 535 | Scale of analysis drives the observed ratio of spatial to non-spatial variance in microbial water quality: insights from two decades of citizen science data | 10.1093/jambio/lxad210 |
| 536 | Scale-dependence of land use effects on water quality of streams in agricultural catchments | 10.1016/j.envpol.2003.10.018 |
| 537 | Scenario-based hydrological modeling for designing climate-resilient coastalwater resource management measures: Lessons from Brahmani river, Odisha, Eastern India | 10.3390/su13116339 |
| 538 | Searching for Balance between Hill Country Pastoral Farming and Nature | 10.3390/land12081482 |
| 539 | Seasonal variation of surface water quality and streamflow in Rispana: A tributary of Ganges river, India | 10.1002/tqem.22199 |
| 540 | Seasonality, richness and prevalence of intestinal parasites of three neotropical primates (Alouatta seniculus, Ateles hybridus and Cebus versicolor) in a fragmented forest in Colombia | 10.1016/j.ijppaw.2017.07.006 |
| 541 | Sediment and fecal indicator bacteria loading in a mixed land use watershed: Contributions from suspended sediment and bedload transport | 10.1002/jeq2.20166 |
| 542 | Semi-quantitative evaluation of fecal contamination potential by human and ruminant sources using multiple lines of evidence | 10.1016/j.watres.2011.03.037 |
| 543 | Sensitivity of streamflow and microbial water quality to future climate and land use change in the West of Ireland | 10.1007/s10113-015-0912-0 |
| 544 | Sewage and faecal sludge management; revisiting discharge standards in India | 10.1007/s13762-022-04688-6 |
| 545 | Simulating fecal coliform bacteria loading from an urbanizing watershed | 10.1081/ESE-120027732 |
| 546 | Soil and water quality linked to landuse pattern -a case study of Karuvannur river basin, Thrissur district, Kerala | NA |
| 547 | Sources and fate of Salmonella and fecal indicator bacteria in an urban creek | 10.1039/c1em10213c |
| 548 | Sources and management of urban stormwater pollution in rural catchments, Australia | 10.1016/j.jhydrol.2008.04.017 |
| 549 | Sources and persistence of fecal coliform bacteria in a rural watershed | 10.2166/wqrj.2003.004 |
| 550 | Sources of nutrients and fecal indicator bacteria to nearshore waters on the north shore of Kaua'i (Hawai'i, USA) | 10.1007/s12237-008-9055-6 |
| 551 | Spatial and hydrologic variation of Bacteroidales, adenovirus and enterovirus in a semi-arid, wastewater effluent-impacted watershed | 10.1016/j.watres.2015.02.023 |
| 552 | Spatial and seasonal variability of the water quality characteristics of a river in Northeast Brazil | 10.1007/s12665-019-8087-5 |
| 553 | Spatial and temporal analysis of land cover change, sedimentation and water quality in the Lake Issaqueena watershed, South Carolina | NA |
| 554 | Spatial and temporal bacterial quality of a lowland agricultural stream in northeast Scotland | 10.1016/S0048-9697(03)00061-5 |
| 555 | Spatial and temporal characterization of escherichia coli, suspended particulate matter and land use practice relationships in a mixed-land use contemporary watershed | 10.3390/W12051228 |
| 556 | Spatial and temporal distribution of Cryptosporidium and Giardia in a drinking water resource: implications for monitoring and risk assessment | 10.1016/j.scitotenv.2013.10.083 |
| 557 | Spatial and temporal drivers of zoonotic pathogen contamination of an agricultural watershed | 10.2134/jeq2011.0203 |
| 558 | Spatial and temporal dynamics of suspended particles and E. coli in a complex surface-water and karst groundwater system as a basis for an adapted water protection scheme, northern Vietnam | 10.1007/s10040-021-02356-6 |
| 559 | Spatial and Temporal Evaluation of Water Streams Using Quality Indexes: A Case Study | 10.3390/w14213526 |
| 560 | Spatial and temporal variability of surface water pollution in the Mekong Delta, Vietnam | 10.1016/j.scitotenv.2014.03.049 |
| 561 | Spatial and temporal variation of fecal indicator organisms in two creeks in beltsville, Maryland | 10.2166/wqrjc.2016.044 |
| 562 | Spatial and Temporal Variations in Microbiological Water Quality of the River Wiwi in Kumasi, Ghana | 10.1007/s12403-014-0128-4 |
| 563 | Spatial and Temporal Variations in Pollution Indicator Bacteria in the Lower Vaal River, South Africa | 10.2175/106143016X14733681695528 |
| 564 | Spatial aspects of surface water quality in the Jakara Basin, Nigeria using chemometric analysis | 10.1080/10934529.2012.673305 |
| 565 | Spatial Assessment and Analysis of Pollution Sources and Water Quality in the Bogue Falaya River and Abita River Watersheds, St. Tammany Parish, La | NA |
| 566 | Spatial assessment of water quality in the vicinity of Lake Alice National Wildlife Refuge, Upper Devils Lake Basin, North Dakota | 10.1007/s10661-014-4222-7 |
| 567 | Spatial characterization of pollution sources: An analysis of in-stream water quality data from the Potomac headwaters of West Virginia | NA |
| 568 | Spatial distribution of coliform bacteria in Batang Arau River, Padang, West Sumatera, Indonesia | 10.1088/1757-899X/602/1/012062 |
| 569 | Spatial pattern assessment of lake kivu basin rivers water quality using national sanitation foundation water quality and rivers pollution indices | 10.5004/dwt.2017.21566 |
| 570 | Spatial patterns of enzymatic activity in large water bodies: Ship-borne measurements of beta-D-glucuronidase activity as a rapid indicator of microbial water quality | 10.1016/j.scitotenv.2018.10.084 |
| 571 | Spatial patterns of water quality in the Cuiaba River basin, Central Brazil | 10.1007/s10661-005-9114-4 |
| 572 | Spatial scale of land-use impacts on riverine drinking source water quality | 10.1002/wrcr.20154 |
| 573 | Spatial variation of physico-chemical and hydrological parameters with land-use in Venkatapura catchment, Karnataka | NA |
| 574 | Spatial-temporal assessment of pollutions in Ekbatan lake using qualitative indices and statistical methods | 10.1007/s10661-019-7762-z |
| 575 | Spatially explicit pollutant load integrated in stream e. coli concentration modeling in a mixed land use catchment | 10.1016/j.watres.2018.07.021 |
| 576 | Spatio-temporal analysis of urban changes and surface water quality | 10.1016/j.jhydrol.2018.12.033 |
| 577 | Spatio-temporal distribution of fecal indicators in three rivers of the Haihe River Basin, China | 10.1007/s11356-015-5907-3 |
| 578 | Spatio-Temporal Variation of Water Quality in the Yan Oya River Basin, Sri Lanka | 10.1007/s11270-023-06151-9 |
| 579 | Spatiotemporal analysis of Cryptosporidium species/genotypes and relationships with other zoonotic pathogens in surface water from mixed-use watersheds | 10.1128/AEM.01924-12 |
| 580 | Spatiotemporal characteristics of the water quality in the Jinsha River Basin (Panzhihua, China) | 10.2166/ws.2020.258 |
| 581 | Spatiotemporal characterization of water chemistry and pollution sources of the Umhlatuzana, Umbilo and Amanzimnyama River catchments of Durban, KwaZulu-Natal, South Africa | 10.1007/s12665-015-4118-z |
| 582 | Spatiotemporal variability and key influencing factors of river fecal coliform within a typical complex watershed | 10.1016/j.watres.2020.115835 |
| 583 | Spatiotemporal Variation and the Role of Wildlife in Seasonal Water Quality Declines in the Chobe River, Botswana | 10.1371/journal.pone.0139936 |
| 584 | Spatiotemporal variation of bacterial water quality and the relationship with pasture land cover | 10.2166/wh.2017.101 |
| 585 | State and potential management to improve water quality in an agricultural catchment relative to a natural baseline | 10.1016/j.agee.2011.07.009 |
| 586 | Statewide empirical modeling of bacterial contamination of surface waters | 10.1111/j.1752-1688.2006.tb04477.x |
| 587 | Statistical assessment and neural network modeling of stream water quality observations of Green River watershed, KY, USA | 10.2166/ws.2019.058 |
| 588 | Statistical assessment of nonpoint source pollution in agricultural watersheds in the Lower Grand River watershed, MO, USA | 10.1007/s11356-018-3682-7 |
| 589 | Statistical investigations into indicator bacteria concentrations in Houston metropolitan watersheds | 10.2175/106143009X12487095236595 |
| 590 | Status and trends of fecal indicator bacteria in two urban watersheds | 10.2175/106143006X102033 |
| 591 | Storm water events in a small agricultural watershed: Characterization and evaluation of improvements in stream water microbiology following implementation of Best Management Practices | 10.1016/j.jglr.2008.12.002 |
| 592 | Stormflow dynamics and loads of Escherichia coli in a large mixed land use catchment | 10.1002/hyp.7480 |
| 593 | Straight Pipes and Household Wastewater Discharges into the Rural Alabama and Impact on Watershed Water Quality with Wetland Land-Uses | NA |
| 594 | Stream water quality changes following timber harvest in a coastal plain swamp forest | 10.1016/S0043-1354(01)00060-4 |
| 595 | Studies show buff ers can reduce bacteria and protect waterways | NA |
| 596 | Study of Spatial Distribution of Water Quality and Landscape Types Impact on Stream Water Quality in Butler County, OH | NA |
| 597 | Study of the impact of land use and hydrogeological settings on the shallow groundwater quality in a peri-urban area of Kampala, Uganda | 10.1016/j.scitotenv.2007.03.035 |
| 598 | Study of Water Quality in Bengalon River on Oil Palm Estate | 10.1051/e3sconf/20186804007 |
| 599 | Study on water quality of Mahabad Dam River and Lake for drinking and tourism purposes | NA |
| 600 | Surface water and contamination sources in urban river watersheds (Northern Portugal) | 10.12912/27197050/133327 |
| 601 | Surface water contamination risk assessment modeled by fuzzy-WRASTIC | 10.2175/106143016X14609975746361 |
| 602 | Surface Water Quality After the Woolsey Fire in Southern California | 10.1007/s11270-022-05844-x |
| 603 | Surface water quality along the central John Muir Trail in the Sierra Nevada Mountains: Coliforms and algae | 10.1089/ham.2009.1037 |
| 604 | Surface water quality and landscape gradients in the north carolina cape fear river basin: The key role of fecal coliform | 10.1353/sgo.2016.0045 |
| 605 | Surface water quality in rural communities in the state of Goi?s during the dry season and its relationship with land use and occupation | 10.1590/S1413-415220220215PT |
| 606 | Surface water quality in rural communities in the state of Goias during the dry season and its relationship with land use and occupation | 10.1590/S1413-415220220215 |
| 607 | Sustenance of Himalayan springs in an emerging water crisis | 10.1007/s10661-021-09731-6 |
| 608 | Temporal and Spatial Monitoring of Escherichia coli and Pathogen Indicators in the Shallow Groundwater and Vadose Zone, Abbotsford, British Columbia, Canada | NA |
| 609 | Temporal stability of E. coli and Enterococci concentrations in a Pennsylvania creek | 10.1007/s11356-019-07030-9 |
| 610 | The 'Black Waters' of Malaysia: Tracking water quality from the peat swamp forest to the sea | NA |
| 611 | The Changing Face of Water: A Dynamic Reflection of Antibiotic Resistance Across Landscapes | 10.3389/fmicb.2018.01894 |
| 612 | The Chao Phraya river basin: Water quality and anthropogenic influences | 10.2166/ws.2018.167 |
| 613 | The effect of anthropogenic pressure shown by microbiological and chemical water quality indicators on the main rivers of Podhale, southern Poland | 10.1007/s11356-017-8826-7 |
| 614 | The effects of precipitation, river discharge, land use and coastal circulation on water quality in coastal Maine | 10.1098/rsos.140429 |
| 615 | The effects of spatial variability of land use on stream water quality in a costal watershed | 10.1007/s10333-008-0122-1 |
| 616 | The effects on lowland habitat in the banks island bird sanctuary number 1, northwest territories, by the growing colony of lesser snow geese (Chen caerulescens caerulescens) | NA |
| 617 | The hydrology and geochemistry of urban and rural watersheds in east-central Missouri | NA |
| 618 | The impact of cattle farming best management practices on surface water nutrient concentrations, faecal bacteria and algal dominance in the Lake Oconee watershed | 10.1111/j.1747-6593.2012.00343.x |
| 619 | The impact of various land uses on the microbial and physicochemical quality of surface water bodies in developing countries: Prioritisation of water resources management areas | 10.1016/j.enmm.2017.10.006 |
| 620 | The INCA-Pathogens model: An application to the Loimijoki River basin in Finland | 10.1016/j.scitotenv.2016.05.043 |
| 621 | The influence of land-use composition on fecal contamination of riverine source water in southern British Columbia | 10.1029/2012WR012455 |
| 622 | The influence of rainfall on the incidence of microbial faecal indicators and the dominant sources of faecal pollution in a Florida river | 10.1111/j.1365-2672.2005.02554.x |
| 623 | The interactions of indicator bacteria and sediments in fresh water streams | NA |
| 624 | The Modelling Approach for Predicting Coastal Pollutions using Rainfall Distributions over Different Land Use/Land Cover | 10.2112/S185-003.1 |
| 625 | The modified SWAT model for predicting fecal coliforms in the Wachusett Reservoir Watershed, USA | 10.1016/j.watres.2012.05.057 |
| 626 | The Multiscale TROPIcal CatchmentS critical zone observatory M-TROPICS dataset II: Land use, hydrology and sediment production monitoring in Houay Pano, northern Lao PDR | 10.1002/hyp.14126 |
| 627 | The need for proper management leading to the sustainability of the Kelani River and its lower basin | 10.24425/jwld.2020.135026 |
| 628 | The relationship of land use practices to surface water quality in the Upper Oconee Watershed of Georgia | 10.1016/S0378-1127(99)00270-4 |
| 629 | The role of land use and environmental factors on microbial pollution of mountainous limestone aquifers | 10.1007/s00254-007-1002-5 |
| 630 | The role of riparian buffer management in reducing off-site impacts from grazed dairy systems | 10.1017/S1742170511000548 |
| 631 | The Settling of Resource Water Quality Objectives for the Modder-Riet River Catchment | NA |
| 632 | The use of selected water quality parameters to identify fecal coliform sources in support of the Sinking Creek total maximum daily load | NA |
| 633 | Tracing stream nitrate in a central Pennsylvania mixed land-use basin using stable isotopes, bacteria, and inorganic chemicals | NA |
| 634 | Tracing water perturbation using NO(3)(-), doc, particles size determination, and bacteria: A method development for karst aquifer water quality hazard assessment | 10.1016/j.scitotenv.2020.138512 |
| 635 | Tracking host sources of Cryptosporidium spp. in raw water for improved health risk assessment | 10.1128/AEM.02788-06 |
| 636 | Tracking Sources and Dissemination of Indicator Antibiotic Resistance Genes at a Watershed Scale | 10.1021/acsestwater.3c00394 |
| 637 | Tracking sources of bacterial contamination in stormwater discharges to Mission Bay, California | 10.2175/106143001x139605 |
| 638 | Tracking the sources of allochthonous organic matter along a subtropical fluvial-estuarine gradient using molecular proxies in view of land uses | 10.1016/j.chemosphere.2020.126435 |
| 639 | Transport and variability of fecal bacteria in carbonate conglomerate aquifers | 10.1111/j.1745-6584.2010.00741.x |
| 640 | Trend analysis of water quality in some rivers with different degress of development within the Sao Paulo State, Brazil | 10.1002/rra.1091 |
| 641 | Trends in water quality of five dairy farming streams in response to adoption of best practice and benefits of long-term monitoring at the catchment scale | 10.1071/MF12155 |
| 642 | Turbidity as an indicator of water quality in diverse watersheds of the upper Pecos River Basin | 10.3390/w2020273 |
| 643 | Twenty Years of Land Use and the Impact of Nitrate, E. Coli and Chlorophyll for Two Lakes in North Central Texas | NA |
| 644 | Understanding the effects of cattle grazing in english chalk streams | NA |
| 645 | Understanding the Impact of Land Use on Microbial Water Quality to Support Decisions for a Future Land Use Plan | NA |
| 646 | Understanding the spatiotemporal pollution dynamics of highly fragile montane watersheds of Kashmir Himalaya, India | 10.1016/j.envpol.2021.117335 |
| 647 | Uptake of nutrients and organic C in streams in New York City drinking-water-supply watersheds | 10.1899/0887-3593(2006)025[0998:UONAOC]2.0.CO;2 |
| 648 | Urban diffuse sources of faecal indicators | 10.2166/wst.2005.0590 |
| 649 | Urban Drool Water Quality in Denver, Colorado: Pollutant Occurrences and Sources in Dry-Weather Flows | NA |
| 650 | Urban growth and water quality in Thimphu, Bhutan | 10.4090/juee.2013.v7n1.082095 |
| 651 | Urban influences on stream chemistry and biology in the Big Brushy Creek watershed, South Carolina | 10.1007/s11270-007-9340-1 |
| 652 | Urban landscapes increase dispersal, gene flow, and pathogen transmission potential in banded mongoose (Mungos mungo) in northern Botswana | 10.1002/ece3.7487 |
| 653 | Urban Pollution of Bagmati River Corridor within the Densely Populated Kathmandu Valley in Nepal | 10.3233/AJW-150017 |
| 654 | Use of fallout radionuclides ((7)Be, (210)Pb) to estimate resuspension of Escherichia coli from streambed sediments during floods in a tropical montane catchment | 10.1007/s11356-015-5595-z |
| 655 | Using a weight-of-evidence approach for management of watersheds | 10.2166/wst.2006.450 |
| 656 | Using generalized additive mixed models to assess spatial, temporal, and hydrologic controls on bacteria and nitrate in a vulnerable agricultural aquifer | 10.1016/j.jconhyd.2015.08.010 |
| 657 | Using Microbial Source Tracking To Identify Contamination Sources in Port Jefferson Harbor, Setauket Harbor, and Conscience Bay on Long Island, New York | 10.3133/sir20215141 |
| 658 | Using microbiological tracers to assess the impact of winter land use restrictions on the quality of stream headwaters in a small catchment | 10.1016/j.scitotenv.2015.09.071 |
| 659 | Using multi-threshold regression techniques to assess river fecal pollution in the highly urbanized Tamsui River watershed | 10.1007/s10661-021-08893-7 |
| 660 | Using radical terraces for erosion control and water quality improvement in Rwanda: A case study in Sebeya catchment | 10.1016/j.envdev.2021.100649 |
| 661 | Using remote sensing to identify changes in land use and sources of fecal bacteria to support a watershed transport model | 10.3390/w6071925 |
| 662 | Using spatial-stream-network models and long-term data to understand and predict dynamics of faecal contamination in a mixed land-use catchment | 10.1016/j.scitotenv.2017.08.151 |
| 663 | Using watershed characteristics to enhance fecal source identification | 10.1016/j.jenvman.2023.117642 |
| 664 | Utilization of Tryptophan-like Fluorescence as a Proxy for E. coli Contamination in a Mixed-Land-Use Karst Basin | 10.3390/hydrology10040074 |
| 665 | Validating Microbial Source Tracking Markers and Assessing the Efficacy of Culturable E. coli and Enterococcus Assays in Ozark Streams, USA | 10.1007/s11270-023-06355-z |
| 666 | Variability of E. coli density and sources in an urban watershed | 10.2166/wh.2010.063 |
| 667 | Variability of escherichia coli concentrations in an Urban watershed in Texas | 10.1061/(ASCE)EE.1943-7870.0000290 |
| 668 | Variability of indicator bacteria at different time scales in the upper hoosic river watershed | 10.1021/es0601437 |
| 669 | Verifying the applicability of SWAT to simulate fecal contamination for watershed management of Selangor River, Malaysia | 10.1016/j.scitotenv.2021.145075 |
| 670 | Vertical flow constructed wetlands as green facades and gardens for on-site greywater treatment in buildings: Two-year mesocosm study on removal performance | 10.1016/j.scitotenv.2023.167362 |
| 671 | Vulnerability of Himalayan springs to climate change and anthropogenic impact: a review | 10.1007/s11629-018-5308-4 |
| 672 | Wastewater discharge through a stream into a mediterranean ramsar wetland: Evaluation and proposal of a nature-based treatment system | 10.3390/su13063540 |
| 673 | Water and sediment microbial quality of mountain and agricultural streams | 10.2134/jeq2017.12.0483 |
| 674 | Water Pollution and Water Quality Assessment of the Way Kuripan River in Bandar Lampung City (Sumatera, Indonesia) | 10.15244/pjoes/153432 |
| 675 | Water quality and restoration in a coastal subdivision stormwater pond | 10.1016/j.jenvman.2007.01.025 |
| 676 | Water quality and the effects of different pastoral animals | 10.1080/00480169.2008.36849 |
| 677 | Water quality and thermal regime of the Motueka River: Influences of land cover, geology and position in the catchment | 10.1080/00288330.2005.9517354 |
| 678 | Water Quality Assessment and Evaluation of Human Health Risk in Mutangwi River, Limpopo Province, South Africa | 10.3390/ijerph18136765 |
| 679 | Water quality assessment and meta model development in Melen watershed - Turkey | 10.1016/j.jenvman.2010.02.021 |
| 680 | Water quality assessment and the influence of landscape metrics at multiple scales in Poyang Lake basin | 10.1016/j.ecolind.2022.109096 |
| 681 | Water quality assessment of six rivers of the Pacific side of Guatemala | 10.1007/s12665-021-09505-w |
| 682 | Water quality assessment with emphasis in parameter optimisation using pattern recognition methods and genetic algorithm | 10.1016/j.watres.2017.12.010 |
| 683 | Water quality changes in a polluted stream over a twenty-five-year period | 10.2134/jeq2003.6540 |
| 684 | Water quality evaluation of two urban streams in Northwest Uruguay: are national regulations for urban stream quality sufficient? | 10.1007/s10661-020-08614-6 |
| 685 | Water quality in aguadas within a protected karstic rain forest: The role of the vegetation-soil-water interactions | 10.1016/j.ecoleng.2017.12.006 |
| 686 | Water quality in an urban environmental protection area in the Cerrado Biome, Brazil | 10.1007/s10661-019-7194-9 |
| 687 | Water quality in low-elevation streams and rivers of New Zealand: Recent state and trends in contrasting land-cover classes | 10.1080/00288330.2004.9517243 |
| 688 | Water quality in microbasins and springs of the mineral water region of Minas Gerais State, Brazil | 10.1007/s10661-022-09885-x |
| 689 | Water quality in New Zealand rivers: current state and trends | 10.1080/00288330.2016.1150309 |
| 690 | Water quality in part of the Submiddle San Francisco region due to seasonality and land use and occupation | 10.1080/15715124.2022.2068560 |
| 691 | Water quality in relation to watershed management in the lower San Gabriel River, southern California | NA |
| 692 | Water quality in shallow alluvial aquifers, Upper Colorado River Basin, Colorado, 1997 | 10.1111/j.1752-1688.2002.tb01541.x |
| 693 | Water quality in various land cover type in nanggala sub watershed | 10.1088/1755-1315/870/1/012027 |
| 694 | Water quality in watershed of the Jaboatao River (Pernambuco, Brazil): A case study | 10.1590/s1516-89132003000400026 |
| 695 | Water quality index in an urban watershed | 10.2495/SDP-V11-N6-1037-1043 |
| 696 | Water quality index in two land use situations in the mantiqueira range | 10.1590/S1413-70542013000400007 |
| 697 | Water Quality Index Using Modified Random Forest Technique: Assessing Novel Input Features | 10.32604/cmes.2022.019244 |
| 698 | Water quality indicators in the Mantiqueira Range region, Minas Gerais state | 10.1590/S0104-77602013000400020 |
| 699 | Water quality modification by land use types in watershed ecosystems of Southwestern Nigeria | NA |
| 700 | Water quality studies in Kranji catchment, Singapore: Use of organic tracers and polyethylene devices for identifying potential sewage sources | 10.2166/wpt.2012.040 |
| 701 | Water quality variation in tributaries of the Three Gorges Reservoir from 2000 to 2015 | 10.1016/j.watres.2021.116993 |
| 702 | Water transport, retention, and survival of Escherichia coli in unsaturated porous media: A comprehensive review of processes, models, and factors | 10.1080/10643389.2013.828363 |
| 703 | Watershed land use, surface water vulnerability and public health risks of two urban rivers, Ado-Ekiti, South-West Nigeria | 10.1007/s42452-020-03572-7 |
| 704 | Watershed planning in central Iowa: An integrated assessment of the Squaw Creek Watershed for prioritization of conservation practice establishment | NA |
| 705 | Watershed water quality modeling using integrated fuzzy modeling approach with HSPF model and radar rainfall data | NA |
| 706 | What matters most? Stakeholders' perceptions of river water quality | 10.1016/j.landusepol.2020.104824 |
| 707 | Whole catchment land cover effects on water quality in the Lower Kaskaskia River Watershed | 10.1007/s11270-011-0794-9 |
| 708 | Widespread detection of human- and ruminant-origin Bacteroidales markers in subtidal waters of the Salish Sea in Washington State | 10.2166/wh.2015.253 |
| 709 | Wildlife identified as major source of Escherichia coli in agriculturally dominated watersheds by BOX A1R-derived genetic fingerprints | 10.1016/j.jenvman.2005.12.013 |
| 710 | Year-Long Metagenomic Study of River Microbiomes Across Land Use and Water Quality | 10.3389/fmicb.2015.01405 |
| 711 | Fluctuating silicate:nitrate ratios and coastal plankton food webs | 10.1073/pnas.95.22.13048 |
