# Supplementary material for: AI-assisted evidence screening method for systematic reviews in environmental research: integrating ChatGPT with domain knowledge
Source: Environ Evid. 2025 Apr 15;14:5. doi: 10.1186/s13750-025-00358-5 (PMC11998256; doi:10.1186/s13750-025-00358-5)
Supplement: Supplementary file 4 — Supplementary Material 4 [file 13750_2025_358_MOESM4_ESM.docx]

**Table A3.** The criteria’s different versions of four rounds in Step 1

| **Versions** | **Criteria Number** | **Criteria Information** |
| --- | --- | --- |
| 1st Round | Criteria 1.1 | Does land use or land cover be mentioned in the research results or the discussion part? |
|  | Criteria 1.2 | Does more than one type of land use or land cover be mentioned in the abstract? |
|  | Criteria 1.3 | Does the word ‘land use’ or ‘land cover’ be mentioned in the title? |
|  | Criteria 1.4 | Does Fecal coliform be explicitly stated as the research subject? |
| 2nd Round | Criteria 1.1 | More than one type of land use or land cover is mentioned in the abstract or title, or the terms “land use” or “land cover” are directly mentioned in the abstract or title, or multiple types of land use or land cover are implied in the abstract. |
|  | Criteria 1.2 | Land use or land cover cannot only be mentioned in background knowledge, implication, policy suggestion, or future research sections. However, land use or land cover needs to be mentioned in the research method, results, or discussion. |
|  | Criteria 1.3 | Fecal coliform, the indicator of fecal coliform, or its related words must be mentioned. The related words of fecal coliform include but are not limited to, ‘E. coli’, ‘escherichia coli’, ‘enterococcus’, ‘fecal’, ‘faecal’, ‘coliform’, ‘coliforms’, ‘fecal bacteria’, 'manure borne pathogen', 'manure borne bacteria', 'waterborne pathogen bacteria', and also understand that words like manure, cattle waste, wildlife waste, human waste, septic tank imply the presence of Fecal coliform. |
|  | Criteria 1.4 | Fecal coliform, the indicator of fecal coliform, or its related words cannot only be mentioned in background knowledge, implication, policy suggestion, or future research sections. However, Fecal coliform, the indicator of fecal coliform, or its related words needs to be mentioned in the research method, results, or discussion. |
|  | Criteria 1.5 | The relationship between land use or land cover with Fecal coliform, the indicator of fecal coliform, or its related words should be mentioned or implied in the results or discussion. However, it cannot only be mentioned in background knowledge, implication, policy suggestion, or future research sections. |
| 3rd Round | Criteria 1.1 | The mention of multiple types of land use or land cover, or direct mention of “land use” or “land cover” in the title or abstract. |
|  | Criteria 1.2 | The land use, land cover, or multiple types of land use or land cover needs to be explicitly mentioned in the research method or results, not just in the background. |
|  | Criteria 1.3 | Fecal coliform, ‘E. coli’, ‘Escherichia coli’, or ‘Enterococci’ needs to be explicitly mentioned in the research method or results, not just in background. |
|  | Criteria 1.4 | The direct relationship between land use, land cover, or multiple types of land use or land cover with fecal coliform, ‘E. coli’, ‘Escherichia coli’, or ‘Enterococci’ needs to be demonstrated in the results, not just theorized in the background. |
| 4th Round | Criteria 1.1 | The title or abstract should contain either the term ‘land use’ or the term ‘land cover’. This requirement can also be satisfied if the title or abstract mentions more than one type of land use or land cover. |
|  | Criteria 1.2 | The research method or results should contain either the term ‘land use’, or the term ‘land cover’. This requirement can also be satisfied if the research method or results mention more than one type of land use or land cover. |
|  | Criteria 1.3 | The research method or results should contain exactly one of the terms in the Fecal Coliform Contamination List. |
|  | Criteria 1.4 | The research method or results should contain the direct relationship between land use/land cover or the types of land use/land cover and Fecal coliform or one of the terms in the Fecal Coliform Contamination List. |
