# Supplementary material for: AI-assisted evidence screening method for systematic reviews in environmental research: integrating ChatGPT with domain knowledge
Source: Environ Evid. 2025 Apr 15;14:5. doi: 10.1186/s13750-025-00358-5 (PMC11998256; doi:10.1186/s13750-025-00358-5)
Supplement: Supplementary file 5 — Supplementary Material 5 [file 13750_2025_358_MOESM5_ESM.docx]

**Table A4.** The reviewers’ results of 130 articles.

| **Unique ID** | **Title** | **Consensus Decision** | **R1** | **R2** | **R3** | **Rounds** | **Dataset** |
| --- | --- | --- | --- | --- | --- | --- | --- |
| 1 | !cuba! river water chemistry reveals rapid chemical weathering, the echo of uplift, and the promise of more sustainable agriculture | Yes | Yes | Yes | Yes | 2 | Train & Validation |
| 3 | a 22-site comparison of land-use practices, e-coli and enterococci concentrations | Yes | Yes | Yes | Yes | 1 | Train & Validation |
| 4 | a basis water quality monitoring plan for rehabilitation and protection | Yes | No | Yes | No | 1 | Train & Validation |
| 5 | a biological and nitrate isotopic assessment framework to understand eutrophication in aquatic ecosystems | No | No | No | Yes | 2 | Train & Validation |
| 6 | a case study characterizing animal fecal sources in surface water using a mitochondrial dna marker | No | No | Yes | Yes | 3 | Train & Validation |
| 7 | a comparison of soil staphylococcus aureus and fecal indicator bacteria concentrations across land uses in a hawaiian watershed | Yes | Yes | No | Yes | 4 | Train & Validation |
| 8 | a comprehensive method for ampliconbased and metagenomic characterization of viruses, bacteria, and eukaryotes in freshwater samples | No | No | No | No | 4 | Train & Validation |
| 10 | a geospatial analysis of land use and stormwater management on fecal coliform contamination in north carolina streams | Yes | Yes | Yes | Yes | 1 | Train & Validation |
| 71 | assessment of water quality based on statistical analysis of physical-chemical, biomonitoring and land use data: manso river supply reservoir | No | No | No | No | 4 | Train & Validation |
| 72 | assessment of water quality in association with land use in the tillamook bay watershed, oregon, usa | No | No | Yes | No | 4 | Train & Validation |
| 75 | assessment, modelling and management of land use and water quality in the upper taieri river catchment | No | No | Yes | No | 3 | Train & Validation |
| 76 | associations among pathogenic bacteria, parasites, and environmental and land use factors in multiple mixed-use watersheds | Yes | Yes | Yes | Yes | 2 | Train & Validation |
| 77 | bacteria modeling with swat for assessment and remediation studies: a review | Yes | Yes | Yes | Yes | 1 | Train & Validation |
| 78 | bacterial capture by peptide-mimetic oligoacyllysine surfaces | No | No | No | No | 2 | Train & Validation |
| 79 | bacterial community composition and structure in an urban river impacted by different pollutant sources | No | No | Yes | No | 3 | Train & Validation |
| 80 | bacterial community structure is indicative of chemical inputs in the upper mississippi river | No | Yes | No | No | 4 | Train & Validation |
| 83 | bacterial pathogens in hawaiian coastal streams-associations with fecal indicators, land cover, and water quality | Yes | Yes | Yes | Yes | 1 | Train & Validation |
| 92 | biogeochemical impacts of sewage effluents in predominantly rural river catchments: are point source inputs distinct to background diffuse pollution? | No | No | No | No | 4 | Train & Validation |
| 94 | biogeographic patterns of potential pathogenic bacteria in the middle and lower reaches of the yangtze river as well as its two adjoining lakes, china | No | No | No | No | 3 | Train & Validation |
| 95 | biomonitoring and water quality evaluation of river beas in mid himalayan zone, india | No | No | No | Yes | 2 | Train & Validation |
| 97 | bslc: a tool for bacteria source characterization for watershed management | Yes | Yes | Yes | Yes | 1 | Train & Validation |
| 99 | causal connections between water quality and land use in a rural tropical island watershed: rural tropical island watershed analysis | Yes | Yes | Yes | No | 1 | Train & Validation |
| 100 | changes in chemical and physical propertiesof stream water across an urban-rural gradient in western georgia | Yes | Yes | Yes | Yes | 2 | Train & Validation |
| 102 | changes in land use/management and water quality in the long creek watershed | Yes | Yes | Yes | No | 3 | Train & Validation |
| 103 | characterisation of sludge produced by the agri-food industry and recycling options for its agricultural uses in a typical mediterranean area, the segura river basin (spain) | No | No | No | No | 4 | Train & Validation |
| 141 | cryptosporidium genotyping and land use mapping for hazard identification and source tracking in a small mixed rural-urban watershed in southeastern brazil | Yes | No | No | Yes | 4 | Train & Validation |
| 142 | cryptosporidium source tracking in the potomac river watershed | No | No | No | No | 3 | Train & Validation |
| 143 | current state of water quality indicators in urban streams in new zealand | Yes | Yes | Yes | Yes | 1 | Train & Validation |
| 144 | decadal and seasonal water quality trends downstream of urban and rural areas in southern alberta rivers | Yes | Yes | Yes | Yes | 1 | Train & Validation |
| 145 | deciphering the influence of multiple anthropogenic inputs on taxonomic and functional profiles of the microbial communities in yitong river, northeast china | No | No | No | Yes | 2 | Train & Validation |
| 146 | demonstrating an integrated antibiotic resistance gene surveillance approach in puerto rican watersheds post-hurricane maria | No | No | Yes | No | 3 | Train & Validation |
| 147 | detangling seasonal relationships of fecal contamination sources and correlates with indicators in michigan watersheds | Yes | Yes | Yes | Yes | 4 | Train & Validation |
| 174 | diverse land use and the impact on (irrigation) water quality and need for measures - a case study of a norwegian river | No | No | No | Yes | 4 | Train & Validation |
| 175 | dna fingerprinting using box-a1r and (gtg)(5) primers identify spatial variations of fecal contamination along pasig river, philippines | Yes | Yes | Yes | No | 3 | Train & Validation |
| 176 | do reductions in agricultural field drainage during the growing season impact bacterial densities and loads in small tile-fed watersheds? | No | No | No | Yes | 2 | Train & Validation |
| 177 | does land use affect pathogen presence in new zealand drinking water supplies? | Yes | Yes | Yes | Yes | 1 | Train & Validation |
| 180 | ecological water health assessment using benthic macroinvertebrate communities (case study: the ghezel ozan river in zanjan province, iran) | Yes | Yes | Yes | No | 1 | Train & Validation |
| 182 | ecosystem dynamics and pollution effects in an ozark cave stream | No | No | No | Yes | 2 | Train & Validation |
| 183 | ecosystemic assessment of surface water quality in the virilla river: towards sanitation processes in costa rica | Yes | Yes | Yes | Yes | 3 | Train & Validation |
| 184 | effect of agricultural activities on surface water quality from paramo ecosystems | Yes | Yes | Yes | No | 4 | Train & Validation |
| 239 | evaluation of the impacts of land use in water quality and the role of nature-based solutions: a citizen science-based study | Yes | Yes | Yes | Yes | 4 | Train & Validation |
| 240 | evaluation of the water quality status and pollution load carrying capacity of way umpu river, way kanan district, lampung province, indonesia, based on land use | Yes | Yes | Yes | Yes | 3 | Train & Validation |
| 241 | evidence for coexistence of distinct escherichia coli populations in various aquatic environments and their survival in estuary water | No | No | No | Yes | 2 | Train & Validation |
| 242 | experimental and modelling evidence of splash effects on manure borne escherichia coli washoff | No | Yes | No | No | 1 | Train & Validation |
| 244 | f-specific rna coliphages: occurrence, types, and survival in natural waters | No | No | No | No | 1 | Train & Validation |
| 246 | factors and mechanisms affecting seasonal changes in the prevalence of microbiological indicators of water quality and nutrient concentrations in waters of the biaka river catchment, southern poland | Yes | No | Yes | Yes | 2 | Train & Validation |
| 247 | factors associated with e. coli levels in and salmonella contamination of agricultural water differed between north and south florida waterways | Yes | Yes | Yes | Yes | 3 | Train & Validation |
| 248 | factors influencing safe managed decentralized wastewater in citarum watershed | No | No | No | No | 4 | Train & Validation |
| 274 | food web transfer of plastics to an apex riverine predator | No | No | No | No | 4 | Train & Validation |
| 275 | fourier landscape pattern indices for predicting south carolina watershed fecal coliform | Yes | Yes | Yes | No | 3 | Train & Validation |
| 276 | freshwater salinization increases survival of escherichia coli and risk of bacterial impairment | No | No | No | Yes | 2 | Train & Validation |
| 277 | generic modelling of faecal indicator organism concentrations in the uk | Yes | Yes | Yes | Yes | 1 | Train & Validation |
| 278 | genetic fecal source identification in urban streams impacted by municipal separate storm sewer system discharges | Yes | Yes | Yes | Yes | 2 | Train & Validation |
| 279 | genome-wide single nucleotide polymorphism (snp) markers from fecal samples reveal anthropogenic impacts on connectivity: case of a small carnivore in the central indian landscape | No | No | No | No | 3 | Train & Validation |
| 280 | geo-environmental impacts of hydrogeological setting and anthropogenic activities on water quality in the quaternary aquifer southeast of the nile delta, egypt | Yes | Yes | Yes | No | 4 | Train & Validation |
| 288 | groundwater and factors affecting its quality: examples from the rovaniemi district of northern finland and western nicaragua | No | No | No | No | 4 | Train & Validation |
| 289 | groupwise modeling study of bacterially impaired watersheds in texas: clustering analysis | Yes | Yes | Yes | Yes | 3 | Train & Validation |
| 290 | growing season surface water loading of fecal indicator organisms within a rural watershed | Yes | Yes | Yes | Yes | 2 | Train & Validation |
| 291 | habitat and host factors associated with liver fluke (fasciola hepatica) diagnoses in wild red deer (cervus elaphus) in the scottish highlands | No | No | No | No | 1 | Train & Validation |
| 292 | habitat selection of endangered amami rabbits on tokuno-shima island in japan as assessed by counting fecal pellet groups on roads | No | No | No | No | 2 | Train & Validation |
| 293 | health risk assessment related to waterborne pathogens from the river to the tap | Yes | No | Yes | Yes | 3 | Train & Validation |
| 294 | heavy metals in the habitat and throughout the food chain of the neotropical otter, lontra longicaudis, in protected mexican wetlands | No | No | No | No | 4 | Train & Validation |
| 311 | hydrogeomorphological controls on groundwater quality in the rattaphum catchment (songkhla lake basin), thailand | No | Yes | No | Yes | 2 | Train & Validation |
| 312 | hydrological regime and water shortage as drivers of the seasonal incidence of diarrheal diseases in a tropical montane environment | No | No | No | No | 4 | Train & Validation |
| 314 | hydrometeorology and flood pulse dynamics drive diarrheal disease outbreaks and increase vulnerability to climate change in surface-water-dependent populations: a retrospective analysis | No | No | No | No | 3 | Train & Validation |
| 316 | identifying fecal pollution sources using 3m petrifilm count plates and antibiotic resistance analysis in the horse creek watershed in aiken county, sc (usa) | No | Yes | No | No | 1 | Train & Validation |
| 317 | identifying sources of fecal pollution in the colville river using library-independent genetic markers | Yes | No | Yes | No | 3 | Train & Validation |
| 318 | impact of changes of land use on water quality, from tropical forest to anthropogenic occupation: a multivariate approach | Yes | Yes | Yes | Yes | 2 | Train & Validation |
| 375 | investigation of relationships between fecal contamination, cattle grazing, human recreation, and microbial source tracking markers in a mixed-land-use rangeland watershed | No | No | No | No | 4 | Train & Validation |
| 376 | irrigation water quality of a community garden complex in the state of piaui, northeastern brazil | No | No | No | No | 3 | Train & Validation |
| 377 | isolating the impact of septic systems on fecal pollution in streams of suburban watersheds in georgia, united states | No | Yes | Yes | Yes | 2 | Train & Validation |
| 378 | jump run creek shellfish restoration project | No | Yes | No | No | 1 | Train & Validation |
| 380 | land cover impacts on stream nutrients and fecal coliform in the lower piedmont of west georgia | Yes | Yes | Yes | Yes | 1 | Train & Validation |
| 381 | land use and environmental variables influence tetracycline-resistant bacteria occurrence in southeastern coastal plain streams | Yes | No | Yes | Yes | 2 | Train & Validation |
| 382 | land use and hydroclimatic influences on faecal indicator organisms in two large scottish catchments: towards land use-based models as screening tools | Yes | Yes | Yes | Yes | 3 | Train & Validation |
| 383 | land use and land cover changes in zezere watershed (portugal)--water quality implications | Yes | Yes | Yes | Yes | 4 | Train & Validation |
| 413 | linking the uptake of best management practices on dairy farms to catchment water quality improvement over a 20-year period | No | Yes | No | No | 3 | Train & Validation |
| 414 | macroinvertebrate indices versus microbial fecal pollution characteristics for water quality monitoring reveals contrasting results for an ethiopian river | Yes | Yes | Yes | No | 1 | Train & Validation |
| 415 | managed aquifer recharge implementation criteria to achieve water sustainability | No | No | No | Yes | 2 | Train & Validation |
| 416 | mapping of critical source areas for diffuse fecal bacterial pollution in extensively grazed watersheds | No | No | No | No | 3 | Train & Validation |
| 417 | mapping of escherichia coli sources connected to waterways in the ruamahanga catchment, new zealand | No | Yes | No | No | 4 | Train & Validation |
| 456 | multiscale spatiotemporal variability of fecal indicator bacteria and associated particle size distributions in the sandy bottom sediments of a pennsylvania creek | Yes | No | Yes | Yes | 3 | Train & Validation |
| 457 | native forest cover safeguards stream water quality under a changing climate | Yes | Yes | Yes | Yes | 1 | Train & Validation |
| 458 | next-generation sequencing reveals fecal contamination and potentially pathogenic bacteria in a major inflow river of taihu lake | No | Yes | Yes | Yes | 2 | Train & Validation |
| 459 | non-point source fecal contamination from aging wastewater infrastructure is a primary driver of antibiotic resistance in surface waters | No | No | Yes | Yes | 3 | Train & Validation |
| 460 | nonpoint source reduction to the nearshore zone via watershed management practices: nutrient fluxes, fate, transport and biotic responses - background and objectives | No | No | No | Yes | 4 | Train & Validation |
| 462 | occurrence and distribution of microbiological indicators in groundwater and stream water | Yes | Yes | Yes | Yes | 1 | Train & Validation |
| 463 | occurrence and distribution of viruses and picoplankton in tropical freshwater bodies determined by flow cytometry | No | No | No | Yes | 2 | Test |
| 464 | occurrence of microbial indicators, pathogenic bacteria and viruses in tropical surface waters subject to contrasting land use | Yes | No | Yes | Yes | 3 | Train & Validation |
| 465 | occurrence of traditional and alternative fecal indicators in tropical urban environments under different land use patterns | Yes | Yes | Yes | Yes | 3 | Train & Validation |
| 467 | participatory approach for more robust water resource management: case study of the santa rosa sub-watershed of the philippines | Yes | Yes | Yes | Yes | 1 | Train & Validation |
| 468 | particle-attached riverine bacteriome shifts in a pollutant-resistant and pathogenic community during a mediterranean extreme storm event | No | No | No | No | 2 | Test |
| 469 | pathogen transport and fate modeling in the upper salem river watershed using swat model | No | No | No | No | 3 | Test |
| 470 | patterns and drivers of fecal coliform exports in a typhoon-affected watershed: insights from 10-year observations and swat model | Yes | Yes | Yes | Yes | 4 | Test |
| 499 | prevalence of antibiotic resistance in the tropical rivers of sri lanka and india | No | Yes | No | No | 3 | Test |
| 502 | quantification of fecal coliform inputs to aquatic systems through soil leaching | Yes | No | Yes | No | 1 | Test |
| 503 | quantification of human-associated fecal indicators reveal sewage from urban watersheds as a source of pollution to lake michigan | No | No | No | No | 2 | Test |
| 504 | quantification of microbial source tracking and pathogenic bacterial markers in water and sediments of tiaoxi river (taihu watershed) | Yes | Yes | Yes | Yes | 3 | Test |
| 506 | quantifying escherichia coli and suspended particulate matter concentrations in a mixed-land use appalachian watershed | Yes | Yes | Yes | No | 4 | Test |
| 511 | quantitative pcr-based detection of pathogenic leptospira in hawai'ian coastal streams | No | No | No | No | 3 | Test |
| 512 | rainfall driven e. coli transfer to the stream conduit network observed through increasing spatial scales in mixed land use paddy farming karst terrain | Yes | No | Yes | Yes | 1 | Test |
| 513 | reach specificity in sediment e. coli population turnover and interaction with waterborne populations | Yes | Yes | Yes | Yes | 2 | Test |
| 515 | real-time consequences of riparian cattle trampling for mobilization of sediment, nutrients and bacteria in a british lowland river | No | No | No | No | 3 | Test |
| 516 | recreational disturbance of river sediments during base flow deteriorates microbial water quality | Yes | No | Yes | Yes | 4 | Test |
| 533 | role of free-ranging mammals in the deposition of escherichia coli into a texas floodplain | No | No | No | No | 3 | Test |
| 534 | sanitary analyses of runoff water a river | Yes | Yes | Yes | No | 1 | Test |
| 535 | scale of analysis drives the observed ratio of spatial to non-spatial variance in microbial water quality: insights from two decades of citizen science data | No | Yes | Yes | Yes | 2 | Test |
| 536 | scale-dependence of land use effects on water quality of streams in agricultural catchments | Yes | Yes | Yes | Yes | 3 | Test |
| 537 | scenario-based hydrological modeling for designing climate-resilient coastalwater resource management measures: lessons from brahmani river, odisha, eastern india | No | No | No | No | 4 | Test |
| 607 | sustenance of himalayan springs in an emerging water crisis | No | No | No | No | 3 | Test |
| 609 | temporal stability of e. coli and enterococci concentrations in a pennsylvania creek | Yes | Yes | Yes | Yes | 1 | Test |
| 610 | the 'black waters' of malaysia: tracking water quality from the peat swamp forest to the sea | Yes | Yes | Yes | Yes | 2 | Test |
| 611 | the changing face of water: a dynamic reflection of antibiotic resistance across landscapes | Yes | No | Yes | Yes | 3 | Test |
| 612 | the chao phraya river basin: water quality and anthropogenic influences | Yes | Yes | Yes | Yes | 4 | Test |
| 642 | turbidity as an indicator of water quality in diverse watersheds of the upper pecos river basin | Yes | Yes | Yes | Yes | 3 | Test |
| 646 | understanding the spatiotemporal pollution dynamics of highly fragile montane watersheds of kashmir himalaya, india | Yes | Yes | Yes | Yes | 1 | Test |
| 647 | uptake of nutrients and organic c in streams in new york city drinking-water-supply watersheds | No | No | No | Yes | 2 | Test |
| 648 | urban diffuse sources of faecal indicators | No | No | No | No | 3 | Test |
| 650 | urban growth and water quality in thimphu, bhutan | No | No | No | No | 4 | Test |
| 664 | utilization of tryptophan-like fluorescence as a proxy for e. coli contamination in a mixed-land-use karst basin | No | No | No | No | 3 | Test |
| 665 | validating microbial source tracking markers and assessing the efficacy of culturable e. coli and enterococcus assays in ozark streams, usa | Yes | Yes | Yes | Yes | 1 | Test |
| 666 | variability of e. coli density and sources in an urban watershed | Yes | Yes | Yes | Yes | 2 | Test |
| 667 | variability of escherichia coli concentrations in an urban watershed in texas | Yes | Yes | Yes | Yes | 3 | Test |
| 668 | variability of indicator bacteria at different time scales in the upper hoosic river watershed | Yes | Yes | Yes | Yes | 4 | Test |
| 671 | vulnerability of himalayan springs to climate change and anthropogenic impact: a review | No | No | No | Yes | 3 | Test |
| 672 | wastewater discharge through a stream into a mediterranean ramsar wetland: evaluation and proposal of a nature-based treatment system | No | Yes | No | No | 1 | Test |
| 673 | water and sediment microbial quality of mountain and agricultural streams | Yes | Yes | Yes | Yes | 2 | Test |
| 674 | water pollution and water quality assessment of the way kuripan river in bandar lampung city (sumatera, indonesia) | Yes | Yes | No | Yes | 3 | Test |
| 675 | water quality and restoration in a coastal subdivision stormwater pond | Yes | No | Yes | No | 4 | Test |
| 710 | year-long metagenomic study of river microbiomes across land use and water quality | Yes | Yes | Yes | Yes | 1 | Test |
