# Supplementary material for: AI-assisted evidence screening method for systematic reviews in environmental research: integrating ChatGPT with domain knowledge
Source: Environ Evid. 2025 Apr 15;14:5. doi: 10.1186/s13750-025-00358-5 (PMC11998256; doi:10.1186/s13750-025-00358-5)
Supplement: Supplementary file 6 — Supplementary Material 6 [file 13750_2025_358_MOESM6_ESM.docx]

**Table A5.** The ChatGPT screening results of 581 articles in Step 1

| **Unique ID** | **Title** | **Majority Answer** | **1** | **2** | **3** | **4** | **5** | **6** | **7** | **8** | **9** | **10** | **11** | **12** | **13** | **14** | **15** |
| --- | --- | --- | --- | --- | --- | --- | --- | --- | --- | --- | --- | --- | --- | --- | --- | --- | --- |
| 2 | escherichia coli concentration, multiscale monitoring over the decade 2011-2021 in the mekong river basin, lao pdr | Yes | Yes | No | No | No | Yes | Yes | No | Yes | No | No | No | Yes | Yes | Yes | Yes |
| 9 | a geographical approach to tracking escherichia coli and other water quality constituents in a texas coastal plains watershed | Yes | Yes | Yes | Yes | Yes | Yes | Yes | Yes | Yes | Yes | Yes | Yes | Yes | Yes | Yes | Yes |
| 11 | a hydrochemically guided landscape classification system for modelling spatial variation in multiple water quality indices: process-attribute mapping | Yes | Yes | Yes | Yes | Yes | Yes | Yes | Yes | Yes | Yes | Yes | Yes | Yes | Yes | Yes | Yes |
| 12 | a load model based on antecedent dry periods for pollutants in stormwater | No | No | No | No | No | No | Yes | No | No | No | No | No | No | No | No | No |
| 13 | a multivariate and spatiotemporal analysis of water quality in code river, indonesia | Yes | Yes | Yes | Yes | Yes | Yes | Yes | Yes | Yes | Yes | Yes | Yes | Yes | Yes | Yes | Yes |
| 14 | a multivariate statistical approach to the integration of different land-uses, seasons, and water quality as water resources management tool | Yes | Yes | Yes | Yes | Yes | Yes | Yes | Yes | Yes | Yes | Yes | Yes | Yes | Yes | Yes | Yes |
| 15 | a novel approach for assessing watershed susceptibility using weighted overlay and analytical hierarchy process (ahp) methodology: a case study in eagle creek watershed, usa | Yes | Yes | No | Yes | Yes | No | No | Yes | Yes | No | Yes | Yes | Yes | Yes | Yes | No |
| 16 | a review of agricultural land use by shorebirds with special reference to habitat conservation in the fraser river delta, british columbia | No | No | No | No | No | No | No | No | No | No | No | No | No | No | No | No |
| 17 | a review of on-farm roadway runoff characterisation and potential management options for ireland | Yes | Yes | No | Yes | Yes | Yes | No | No | Yes | Yes | Yes | Yes | No | Yes | No | Yes |
| 18 | a short review of fecal indicator bacteria in tropical aquatic ecosystems: knowledge gaps and future directions | No | No | No | No | No | No | No | No | No | No | No | No | No | No | No | No |
| 19 | a socio-ecological systems perspective on planning for informality | No | No | No | No | No | No | No | No | No | No | No | No | No | No | No | No |
| 20 | a spatial assessment of baseline nutrient and water quality values in the ashepoo-combahee-edisto (ace) basin, south carolina, usa | Yes | Yes | Yes | Yes | No | Yes | No | Yes | No | No | No | No | Yes | Yes | Yes | No |
| 21 | a spatial-statistical approach for modeling the effect of non-point source pollution on different water quality parameters in the velhas river watershed--brazil | Yes | Yes | Yes | Yes | Yes | Yes | Yes | Yes | Yes | Yes | Yes | Yes | Yes | Yes | Yes | Yes |
| 22 | a spatiotemporal analysis of water quality and land use in tambayakbayan river, yogyakarta | Yes | Yes | Yes | No | Yes | Yes | Yes | No | Yes | Yes | Yes | Yes | Yes | Yes | Yes | Yes |
| 23 | a study focused on the risk of illness from escherichia coli in recreational-use water, using the red cedar watershed as a model | Yes | Yes | Yes | Yes | Yes | Yes | Yes | Yes | No | No | Yes | Yes | Yes | Yes | Yes | Yes |
| 24 | a systematic assessment of watershed-scale nonpoint source pollution during rainfall-runoff events in the miyun reservoir watershed | Yes | Yes | Yes | Yes | Yes | Yes | Yes | Yes | Yes | Yes | Yes | Yes | Yes | Yes | Yes | Yes |
| 25 | a watershed study assessing effects of commercial hog operations on microbial water quality in north carolina, usa | Yes | Yes | Yes | Yes | Yes | Yes | Yes | Yes | Yes | Yes | Yes | Yes | Yes | Yes | Yes | Yes |
| 26 | abiotic and biotic changes at the basin scale in a tropical dry forest landscape after hurricanes jova and patricia in jalisco, mexico | Yes | Yes | Yes | Yes | Yes | Yes | Yes | No | No | Yes | Yes | Yes | Yes | Yes | Yes | Yes |
| 27 | abundance, diversity, and host assignment of total, intracellular, and extracellular antibiotic resistance genes in riverbed sediments | No | No | No | No | No | No | No | No | No | No | No | No | No | No | No | No |
| 28 | achieving sustainable water and land use systems in highly developed tropical landscapes | Yes | Yes | Yes | Yes | Yes | Yes | Yes | Yes | Yes | Yes | Yes | Yes | Yes | Yes | Yes | Yes |
| 29 | actinomycetes in the elbow river basin, alberta, canada | No | No | No | No | No | No | No | No | No | No | No | No | No | No | No | No |
| 30 | adaptive variations of sediment microbial communities and indication of fecal-associated bacteria to nutrients in a regulated urban river | No | No | No | Yes | Yes | No | No | No | No | No | No | Yes | No | Yes | No | Yes |
| 31 | adopting basic quality tools and landscape analysis for applied limnology: an approach for freshwater reservoir management | No | No | No | No | No | No | No | No | No | No | No | No | No | No | No | No |
| 32 | advances in modeling, sampling, and assessing the anthropogenic contamination potential of fractured bedrock aquifers | No | No | No | No | No | No | No | No | No | No | No | No | No | No | No | No |
| 33 | advancing understanding of land use and physicochemical impacts on fecal contamination in mixed-land-use watersheds | Yes | Yes | Yes | Yes | Yes | Yes | Yes | Yes | Yes | Yes | Yes | Yes | Yes | Yes | Yes | Yes |
| 34 | agricultural land use changes stream dissolved organic matter via altering soil inputs to streams | No | No | No | No | No | No | No | No | No | No | No | No | No | No | No | No |
| 35 | an applied ecological approach for the assessment of anthropogenic disturbances in urban wetlands and the contributor river | No | No | Yes | Yes | Yes | Yes | No | No | No | No | Yes | No | No | No | No | No |
| 36 | an assessment of groundwater potential and vulnerability in the upper manyame sub-catchment of zimbabwe | No | No | No | No | Yes | Yes | No | Yes | No | No | No | Yes | Yes | No | Yes | Yes |
| 37 | an assessment of in-situ water quality parameters and its variation with landsat 8 level 1 surface reflectance datasets | No | Yes | No | No | No | Yes | Yes | No | Yes | No | No | Yes | Yes | No | No | No |
| 38 | an assessment of water quality and urbanization in the gills creek watershed | Yes | Yes | Yes | Yes | Yes | Yes | Yes | Yes | No | Yes | Yes | Yes | Yes | Yes | Yes | Yes |
| 39 | an evaluation of a lake houston tributary, cypress creek, for contamination and water quality | Yes | Yes | Yes | Yes | Yes | Yes | Yes | Yes | Yes | Yes | Yes | Yes | Yes | Yes | Yes | Yes |
| 40 | an inca model for pathogens in rivers and catchments: model structure, sensitivity analysis and application to the river thames catchment, uk | Yes | Yes | Yes | Yes | Yes | No | Yes | Yes | No | Yes | Yes | Yes | Yes | No | Yes | Yes |
| 41 | an integrated watershed modeling methodology for water quality restoration | Yes | Yes | No | No | Yes | Yes | Yes | Yes | Yes | Yes | Yes | No | Yes | Yes | Yes | Yes |
| 42 | analysis of escherichia coli and enterococci concentrations patterns in a pennsylvania creek using empirical orthogonal functions | Yes | Yes | Yes | Yes | Yes | Yes | Yes | Yes | Yes | Yes | Yes | Yes | Yes | Yes | Yes | Yes |
| 43 | analysis of the hspf water quality parameter uncertainty in predicting peak in-stream fecal coliform concentrations | Yes | Yes | Yes | Yes | Yes | Yes | Yes | Yes | Yes | Yes | Yes | Yes | Yes | Yes | Yes | Yes |
| 44 | analytical and detection sources of pollution based environmetric techniques in malacca river, malaysia | Yes | Yes | Yes | Yes | Yes | Yes | Yes | Yes | Yes | Yes | Yes | No | Yes | Yes | Yes | Yes |
| 45 | anthropogenic influence on surface water quality of the nhue and day sub-river systems in vietnam | Yes | Yes | Yes | Yes | Yes | Yes | Yes | No | Yes | Yes | No | Yes | No | Yes | Yes | Yes |
| 46 | antimicrobial resistance of escherichia coli isolated from freshwaters and hospital effluents in belgium | No | No | No | No | No | No | No | No | No | No | No | No | No | No | No | No |
| 47 | application of coagulation and foam concentration method to quantify waterborne pathogens in river water samples | No | No | Yes | Yes | Yes | No | No | Yes | Yes | No | No | No | No | No | Yes | Yes |
| 48 | application of host-specific genetic markers for microbial source tracking of faecal water contamination in an agricultural catchment | Yes | No | Yes | Yes | Yes | No | Yes | Yes | Yes | Yes | Yes | Yes | Yes | Yes | Yes | Yes |
| 49 | application of indexes to assess the water quality of coastal basin of the sapucaia in sergipe | Yes | Yes | Yes | No | Yes | No | Yes | Yes | No | Yes | No | Yes | No | Yes | Yes | No |
| 50 | application of microbial source tracking techniques to characterize fecal pollution entering taihu lake (china) | Yes | Yes | Yes | Yes | Yes | Yes | Yes | Yes | Yes | Yes | Yes | Yes | Yes | Yes | Yes | Yes |
| 51 | application of multivariate statistical methodology to model factors influencing fate and transport of fecal pollution in surface waters | No | No | No | No | No | No | No | No | No | No | No | No | No | No | No | No |
| 52 | applications of source-tracking and site-specific models for water quality assessment of tidal waters: upper inlet creek, mount pleasant, south carolina | Yes | Yes | Yes | Yes | Yes | Yes | Yes | Yes | Yes | Yes | Yes | Yes | Yes | Yes | Yes | Yes |
| 53 | applying mar analysis to identify human and non-human fecal sources in small kentucky watersheds | No | No | Yes | Yes | No | No | No | No | No | No | No | Yes | No | No | Yes | No |
| 54 | applying the manning equation to determine the critical distance in non-point source pollution using remotely sensed data and cartographic modelling | Yes | Yes | No | Yes | Yes | Yes | Yes | Yes | No | Yes | Yes | No | Yes | Yes | Yes | Yes |
| 55 | assessing environmental contamination of river ganga using correlation and multivariate analysis | Yes | No | Yes | Yes | Yes | No | Yes | Yes | Yes | Yes | Yes | No | Yes | No | Yes | No |
| 56 | assessing land-cover effects on stream water quality in metropolitan areas using the water quality index | Yes | Yes | Yes | Yes | Yes | Yes | Yes | Yes | Yes | Yes | Yes | Yes | Yes | Yes | Yes | Yes |
| 57 | assessing natural and anthropogenic drivers of regional water quality using hierarchical modeling | Yes | Yes | Yes | No | Yes | Yes | Yes | Yes | Yes | Yes | Yes | No | Yes | Yes | Yes | No |
| 58 | assessing performance of local materials for the treatment of dry weather flows in open drains: results of semi-controlled field experiment research in bangalore, india | No | Yes | No | No | Yes | Yes | No | No | No | No | No | Yes | No | Yes | No | No |
| 59 | assessing the impact of modern recharge on a sandstone aquifer beneath a suburb of doncaster, uk | No | No | No | No | No | No | No | No | No | No | No | No | No | No | No | No |
| 60 | assessing the impacts of watershed indexes and precipitation on spatial in-stream e. coli concentrations | Yes | Yes | Yes | Yes | Yes | Yes | Yes | Yes | Yes | Yes | Yes | Yes | Yes | Yes | Yes | Yes |
| 61 | assessing the service of water quality regulation by quantifying the effects of land use on water quality and public health in central veracruz, mexico | Yes | Yes | Yes | Yes | Yes | Yes | Yes | Yes | Yes | Yes | Yes | Yes | Yes | Yes | Yes | Yes |
| 62 | assessing the yield and load of contaminants with stream order: would policy requiring livestock to be fenced out of high-order streams decrease catchment contaminant loads? | Yes | Yes | Yes | Yes | Yes | Yes | Yes | Yes | Yes | Yes | Yes | Yes | Yes | Yes | Yes | Yes |
| 63 | assessing water quality dynamics in tidally influenced blackwater rivers along a rural-urban gradient | No | No | No | No | No | No | No | No | No | No | No | No | No | No | No | No |
| 64 | assessing water use and quality through youth participatory research in a rural andean watershed | Yes | Yes | Yes | Yes | Yes | Yes | Yes | Yes | Yes | Yes | No | Yes | Yes | Yes | Yes | No |
| 65 | assessment of characteristics, water quality and groundwater vulnerability in pakis district, east java province, indonesia | Yes | Yes | Yes | Yes | Yes | Yes | Yes | Yes | Yes | Yes | No | Yes | Yes | No | Yes | Yes |
| 66 | assessment of emerging hydrological, water quality issues and policy discussion on water sharing of transboundary kabul river | Yes | Yes | Yes | No | Yes | Yes | No | Yes | Yes | No | No | Yes | Yes | Yes | Yes | No |
| 67 | assessment of fecal coliform and escherichia coli across a land cover gradient in west georgia streams | Yes | Yes | Yes | Yes | Yes | Yes | Yes | Yes | Yes | Yes | Yes | Yes | Yes | Yes | Yes | Yes |
| 68 | assessment of physico-chemical and microbiological parameters of mthatha river in eastern cape, south africa | Yes | Yes | Yes | Yes | Yes | Yes | Yes | Yes | No | Yes | Yes | Yes | Yes | Yes | Yes | Yes |
| 69 | assessment of the impacts of landscape patterns on water quality in trondheim rivers and fjord, norway | No | No | No | No | No | No | No | No | No | No | Yes | No | No | No | No | No |
| 70 | assessment of water quality and identification of pollution risk locations in tiaoxi river (taihu watershed), china | Yes | Yes | Yes | Yes | Yes | Yes | No | No | No | Yes | No | Yes | Yes | Yes | Yes | No |
| 73 | assessment of water quality of four mahoning river sub-watersheds, northeast ohio | No | No | No | No | No | Yes | No | No | No | No | No | No | No | No | No | No |
| 74 | assessment of water quality, benthic invertebrates, and periphyton in the threemile creek basin, mobile, alabama, 1999-2003. | Yes | No | No | Yes | No | No | Yes | Yes | No | No | Yes | Yes | Yes | Yes | No | Yes |
| 81 | bacterial indicators of faecal pollution in the waters of the el-kabir river and akkar watershed in syria and lebanon | Yes | Yes | Yes | Yes | No | No | Yes | Yes | No | Yes | Yes | No | Yes | Yes | Yes | Yes |
| 82 | bacterial loadings watershed model in copano bay | Yes | Yes | Yes | Yes | Yes | Yes | Yes | Yes | Yes | Yes | Yes | Yes | Yes | Yes | Yes | Yes |
| 84 | bacterial source tracking and shellfish contamination in a coastal catchment | No | No | No | No | No | No | No | No | No | No | No | No | No | No | No | No |
| 85 | bacterial source tracking and survival of escherichia coli | No | No | Yes | Yes | Yes | No | Yes | No | Yes | No | No | No | No | No | No | No |
| 86 | bacterial total maximum daily load (tmdl): development and evaluation of a new classification scheme for impaired waterbodies of texas | Yes | Yes | No | Yes | Yes | Yes | Yes | Yes | Yes | Yes | Yes | Yes | Yes | Yes | Yes | Yes |
| 87 | bacterial, fungal and algal population of pennar river: a fresh water wetland in kottayam district, kerala | No | No | No | No | Yes | Yes | No | No | No | No | Yes | No | Yes | No | Yes | Yes |
| 88 | bacteriological quality of south african irrigation water and its role as a source of contamination on irrigated lettuce | Yes | No | Yes | Yes | Yes | Yes | No | No | No | Yes | Yes | Yes | No | Yes | Yes | No |
| 89 | beach sands along the california coast are diffuse sources of fecal bacteria to coastal waters | No | No | No | No | No | No | No | No | No | No | No | Yes | No | No | No | No |
| 90 | bile acids combined with fecal sterols: a multiple biomarker approach for deciphering fecal pollution using river sediments | No | No | No | No | No | No | No | No | No | No | No | No | No | No | No | No |
| 91 | biogeochemical and microbial indicators of land-use change in a northern gulf of mexico estuary | No | Yes | Yes | No | No | No | No | Yes | Yes | Yes | No | No | No | Yes | Yes | No |
| 93 | biogeochemical influence of chinese privet in riparian forests in west georgia and the influences of oyster harvesting in apalachicola bay, florida | No | No | Yes | Yes | No | No | Yes | No | Yes | No | Yes | Yes | No | No | No | Yes |
| 96 | biomonitoring in the anthropocene: environmental dna (edna) assessments of changing ecosystems | No | No | No | No | No | No | No | No | No | No | No | No | No | No | No | No |
| 98 | calibration and sensitivity analysis of a novel water flow and pollution model for future city planning: future urban stormwater simulation (fuss) | No | No | No | No | No | No | No | No | No | No | No | No | No | No | No | No |
| 101 | changes in land use land cover (lulc), surface water quality and modelling surface discharge in beaver creek watershed, northeast tennessee and southwest virginia | Yes | Yes | Yes | Yes | Yes | Yes | Yes | Yes | Yes | Yes | Yes | Yes | Yes | Yes | Yes | Yes |
| 104 | characteristics of urban development and associated stormwater quality | Yes | Yes | Yes | Yes | Yes | No | Yes | Yes | Yes | Yes | Yes | Yes | Yes | Yes | Yes | Yes |
| 105 | characterization and prediction of stormwater runoff quality in sub-tropical rural catchments | Yes | Yes | No | No | Yes | Yes | No | Yes | No | Yes | Yes | Yes | Yes | Yes | No | No |
| 106 | characterization of and relations among precipitation, streamflow, suspended-sediment, and water-quality data at the u.s. army garrison fort carson and pinon canyon maneuver site, colorado, water years 2016-18 | No | No | No | No | No | No | No | No | No | No | No | No | No | No | No | No |
| 107 | characterization of nonpoint source microbial contamination in an urbanizing watershed serving as a municipal water supply | Yes | Yes | Yes | Yes | Yes | Yes | Yes | Yes | Yes | Yes | Yes | Yes | Yes | Yes | Yes | Yes |
| 108 | characterization of rain and roof drainage water quality in xanthi, greece | No | No | No | No | No | No | No | No | No | No | No | No | No | No | No | No |
| 109 | characterization of sources and loadings of fecal pollutants using microbial source tracking assays in urban and rural areas of the grand river watershed, southwestern ontario | Yes | Yes | Yes | Yes | Yes | Yes | Yes | Yes | Yes | Yes | Yes | Yes | Yes | Yes | Yes | Yes |
| 110 | characterization of water quality pollution in mixed land use watersheds | Yes | Yes | Yes | Yes | Yes | Yes | Yes | Yes | Yes | Yes | Yes | Yes | Yes | Yes | Yes | Yes |
| 111 | characterizing differences in sources of and contributions to fecal contamination of sediment and surface water with the microbial fit framework | Yes | Yes | Yes | Yes | Yes | Yes | Yes | Yes | Yes | Yes | Yes | Yes | Yes | No | Yes | Yes |
| 112 | characterizing relationships among fecal indicator bacteria, microbial source tracking markers, and associated waterborne pathogen occurrence in stream water and sediments in a mixed land use watershed | Yes | Yes | Yes | Yes | Yes | Yes | Yes | Yes | Yes | Yes | Yes | Yes | Yes | Yes | Yes | Yes |
| 113 | chemical and microbiological indicators to assess the impact of agricultural activities on groundwater in the pampean agro-ecosystem | Yes | Yes | Yes | Yes | Yes | Yes | Yes | Yes | Yes | Yes | Yes | Yes | Yes | Yes | Yes | Yes |
| 114 | chronic urban hotspots and agricultural drainage drive microbial pollution of karst water resources in rural developing regions | Yes | Yes | Yes | Yes | Yes | Yes | Yes | Yes | Yes | Yes | Yes | Yes | Yes | Yes | Yes | Yes |
| 115 | classification and prediction of fecal coliform in stream waters using decision trees (dts) for upper green river watershed, kentucky, usa | No | Yes | No | No | No | No | No | Yes | No | No | Yes | Yes | Yes | Yes | Yes | No |
| 116 | climate and land-use change impact on faecal indicator bacteria in a temperate maritime catchment (the river conwy, wales) | Yes | Yes | Yes | Yes | Yes | Yes | Yes | Yes | Yes | Yes | Yes | Yes | Yes | Yes | Yes | Yes |
| 117 | climate change and land use drivers of fecal bacteria in tropical hawaiian rivers | Yes | Yes | Yes | Yes | Yes | Yes | Yes | Yes | Yes | Yes | Yes | Yes | Yes | Yes | Yes | Yes |
| 118 | climate change mitigation for agriculture: water quality benefits and costs | No | No | No | No | No | No | No | No | No | No | No | No | No | No | No | No |
| 119 | climate warming, environmental degradation and pollution as drivers of antibiotic resistance | No | No | No | No | No | No | No | No | No | No | No | No | No | No | No | No |
| 120 | clues model calibration and its implications for estimating contaminant attenuation | No | No | No | No | No | No | No | No | No | No | No | No | No | No | No | No |
| 121 | clues model calibration: residual analysis to investigate potential sources of model error | No | No | No | No | No | No | No | No | No | No | No | No | No | No | No | No |
| 122 | coherence among different microbial source tracking markers in a small agricultural stream with or without livestock exclusion practices | Yes | Yes | Yes | No | Yes | No | No | Yes | Yes | No | Yes | No | Yes | Yes | Yes | Yes |
| 123 | coliform status of water bodies from two districts in ghana, west africa: implications for rural water resources management | No | Yes | Yes | No | No | No | No | Yes | No | No | No | Yes | No | Yes | No | No |
| 124 | combining land use information and small stream sampling with pcr-based methods for better characterization of diffuse sources of human fecal pollution | Yes | Yes | Yes | Yes | Yes | Yes | Yes | Yes | Yes | Yes | Yes | Yes | Yes | Yes | Yes | Yes |
| 125 | comparing the fate and transport of ms2 bacteriophage and sodium fluorescein in a karstic chalk aquifer | No | No | No | No | No | No | No | No | No | No | No | No | No | No | No | No |
| 126 | comparison of biofiltration media in treating industrial stormwater runoff | No | No | No | No | No | No | No | No | No | No | No | No | No | No | No | No |
| 127 | comparison of qpcr and amplicon sequencing based methods for fecal source tracking in a mixed land use estuarine watershed | Yes | Yes | Yes | Yes | Yes | Yes | Yes | Yes | Yes | Yes | Yes | Yes | Yes | Yes | Yes | Yes |
| 128 | comparison of the performance of decision tree (dt) algorithms and extreme learning machine (elm) model in the prediction of water quality of the upper green river watershed | No | No | No | No | No | No | No | No | No | No | No | No | No | No | No | No |
| 129 | complexity and uncertainty in human and ecological risk assessment | No | No | No | Yes | No | No | No | No | No | No | No | No | Yes | No | No | No |
| 130 | compositions of first flush and composite storm water runoff in small urban and rural watersheds, north-central texas | Yes | Yes | Yes | Yes | Yes | Yes | Yes | Yes | Yes | Yes | Yes | Yes | Yes | Yes | Yes | No |
| 131 | comprehensive evaluation of bacteroidales for identification of fecal contamination sources in freshwater | Yes | Yes | Yes | Yes | Yes | Yes | Yes | No | Yes | Yes | Yes | Yes | Yes | Yes | Yes | Yes |
| 132 | confirming the source of high-sulfate concentrations in dead horse creek, winkler, manitoba, canada, using a dual-isotope bayesian probability mixing model | No | No | No | No | No | No | No | No | No | No | No | No | No | No | No | No |
| 133 | connecting microbial, nutrient, physiochemical, and land use variables for the evaluation of water quality within mixed use watersheds | Yes | Yes | Yes | Yes | Yes | Yes | Yes | Yes | Yes | Yes | Yes | Yes | Yes | Yes | Yes | Yes |
| 134 | conservation biology of the cross river gorilla (gorilla gorilla diehli) | No | No | No | No | No | No | No | No | No | No | No | No | No | No | No | No |
| 135 | conservation genetics of neotropical otters (lontra longicaudis) in mexico | No | No | No | No | No | No | No | No | No | No | No | No | No | No | No | No |
| 136 | contamination with bacterial zoonotic pathogen genes in u.s. streams influenced by varying types of animal agriculture | Yes | Yes | Yes | Yes | Yes | Yes | Yes | Yes | Yes | Yes | Yes | Yes | Yes | No | Yes | No |
| 137 | controls on the chemical and isotopic compositions of urban stormwater in a semiarid zone | No | No | No | No | Yes | No | No | No | Yes | No | No | No | No | No | No | No |
| 138 | converting treatment wetlands into treatment gardens: use of ornamental plants for greywater treatment | No | No | No | No | No | Yes | No | Yes | No | No | No | No | No | No | No | No |
| 139 | coupled dynamics of fecal indicator bacteria in sandy sediments and the water column: a 3-year high-frequency study at a pennsylvania creek | Yes | Yes | Yes | Yes | Yes | Yes | Yes | Yes | Yes | Yes | Yes | Yes | Yes | Yes | Yes | Yes |
| 140 | covid-19 lockdown pandemic period effects in highly impacted aquatic ecosystems | No | No | No | No | No | No | No | No | No | No | No | No | No | No | No | No |
| 148 | detection and monitoring of microbes of concern in animal production environment | No | No | No | No | No | No | No | No | No | No | No | No | No | No | No | No |
| 149 | detection of helicobacter pylori and fecal indicator bacteria in five north american rivers | No | No | No | No | No | No | No | No | No | No | No | No | No | No | No | No |
| 150 | detection of human enteric viruses in stream water with rt-pcr and cell culture | No | No | No | No | No | No | No | No | No | No | No | No | No | No | No | No |
| 151 | detection of human-associated bacteria in water from akiyoshi-do cave, japan | No | No | No | No | No | No | No | No | No | No | No | No | No | No | No | No |
| 152 | detection of sars-cov-2 in urban stormwater: an environmental reservoir and potential interface between human and animal sources | No | No | No | No | No | No | No | No | No | No | No | No | No | No | No | No |
| 153 | determinants of spatio-temporal variability of water quality in the barotse floodplain, western zambia | Yes | Yes | Yes | No | Yes | Yes | Yes | Yes | Yes | Yes | Yes | Yes | Yes | Yes | Yes | Yes |
| 154 | determination effects of impervious areas on urban watershed | No | No | No | No | No | No | No | No | No | No | No | No | No | No | No | No |
| 155 | determination of urban groundwater pollution in alluvial aquifer using linked process models considering urban water cycle | No | No | No | No | No | No | No | No | No | No | No | No | No | No | No | No |
| 156 | determination ofwater quality of rivers under various land use activities using physico-chemical parameters and bacterial populations in northern peninsular malaysia | Yes | Yes | Yes | Yes | Yes | Yes | Yes | Yes | Yes | Yes | Yes | Yes | Yes | Yes | Yes | Yes |
| 157 | determining hot spots of fecal contamination in a tropical watershed by combining land-use information and meteorological data with source-specific assays | Yes | Yes | Yes | Yes | Yes | Yes | Yes | Yes | Yes | Yes | Yes | Yes | Yes | Yes | Yes | Yes |
| 158 | determining overall water quality related to anthropogenic influences across freshwater systems of thailand | Yes | No | Yes | Yes | Yes | No | No | Yes | Yes | Yes | Yes | Yes | Yes | No | Yes | No |
| 159 | determining the degree of fecal pollution in natural waterways feeding the mississippi river in hancock county, illinois | Yes | Yes | Yes | Yes | Yes | Yes | Yes | Yes | Yes | Yes | Yes | Yes | Yes | Yes | Yes | Yes |
| 160 | determining the primary sources of fecal pollution using microbial source tracking assays combined with land-use information in the edwards aquifer | Yes | Yes | Yes | Yes | Yes | Yes | Yes | Yes | Yes | Yes | Yes | Yes | Yes | Yes | Yes | Yes |
| 161 | developing alternative regression models for describing water quality using a self-organizing map | No | No | No | No | No | No | No | No | No | No | No | No | No | No | No | No |
| 162 | development and application of ecosystem health indicators in the north american great lakes basin | No | No | No | No | No | No | No | No | No | No | No | No | No | No | No | No |
| 163 | development and application of exceedance model for surface water quality parameters | Yes | Yes | Yes | Yes | Yes | Yes | Yes | Yes | Yes | Yes | Yes | No | Yes | Yes | Yes | Yes |
| 164 | development and evaluation of the bacterial fate and transport module for the agricultural policy/environmental extender (apex) model | Yes | Yes | Yes | Yes | Yes | Yes | Yes | Yes | Yes | Yes | Yes | Yes | Yes | Yes | Yes | Yes |
| 165 | development of a national-scale framework to characterise transfers of n, p and escherichia coli from land to water | No | No | No | No | No | No | No | No | No | No | No | No | No | No | No | No |
| 166 | development of a pathogen transport model for irish catchments using swat | No | Yes | No | No | No | Yes | No | No | No | Yes | No | No | Yes | No | No | No |
| 167 | development of a process-based model to predict pathogen budgets for the sydney drinking water catchment | Yes | Yes | Yes | Yes | Yes | No | Yes | Yes | Yes | Yes | Yes | Yes | Yes | Yes | Yes | Yes |
| 168 | development of a risk-based index for source water protection planning, which supports the reduction of pathogens from agricultural activity entering water resources | Yes | Yes | Yes | No | Yes | Yes | Yes | Yes | Yes | Yes | Yes | Yes | No | Yes | Yes | Yes |
| 169 | development of multiple regression models to predict sources of fecal pollution | No | No | No | No | No | No | No | No | No | No | No | No | Yes | No | No | Yes |
| 170 | development of regression-based models to predict fecal bacteria at the illinois river basin, arkansas and oklahoma | No | No | No | No | No | No | No | No | No | No | No | No | No | No | No | No |
| 171 | diffuse and point pollution impacts on the pathogen indicator organism level in the geum river, korea | Yes | Yes | Yes | Yes | Yes | Yes | Yes | Yes | Yes | Yes | Yes | Yes | Yes | Yes | Yes | Yes |
| 172 | dissolved inorganic nitrogen, soluble reactive phosphorous, and microbial pollutant loading from tropical rural watersheds in hawai'i to the coastal ocean during non-storm conditions | Yes | No | Yes | Yes | Yes | Yes | No | Yes | No | Yes | Yes | No | No | No | No | Yes |
| 173 | distribution and diversity of escherichia coli populations in the south nation river drainage basin, eastern ontario, canada | Yes | Yes | Yes | Yes | Yes | Yes | Yes | Yes | Yes | Yes | Yes | Yes | Yes | Yes | Yes | Yes |
| 178 | earlier detection of rumors in online social networks using certainty-factor-based convolutional neural networks | No | No | No | No | No | No | No | No | No | No | No | No | No | No | No | No |
| 179 | ecological status of aquatic communities in selected streams in the milwaukee metropolitan sewerage district planning area of wisconsin, 2004-13. | Yes | Yes | Yes | Yes | Yes | Yes | Yes | Yes | Yes | Yes | Yes | Yes | Yes | Yes | Yes | Yes |
| 181 | ecology of tigers in churia habitat and a non-invasive genetic approach to tiger conservation in terai arc, nepal | No | No | No | No | No | No | No | No | No | No | No | No | No | No | No | No |
| 185 | effect of development on water quality for seven streams in north carolina | Yes | Yes | Yes | Yes | Yes | Yes | Yes | Yes | Yes | Yes | Yes | Yes | Yes | Yes | Yes | Yes |
| 186 | effect of human development on bacteriological water quality in coastal watersheds | Yes | Yes | Yes | Yes | Yes | Yes | Yes | Yes | Yes | Yes | Yes | Yes | Yes | Yes | Yes | Yes |
| 187 | effect of land use and hydrological processes on escherichia coli concentrations in streams of tropical, humid headwater catchments | Yes | Yes | Yes | Yes | Yes | Yes | Yes | Yes | Yes | Yes | Yes | Yes | Yes | Yes | Yes | Yes |
| 188 | effect of land use and land cover changes on water quality in the nawuni catchment of the white volta basin, northern region, ghana | Yes | Yes | Yes | No | Yes | Yes | Yes | Yes | Yes | Yes | No | Yes | Yes | Yes | Yes | Yes |
| 189 | effect of streambed bacteria release on e. coli concentrations: monitoring and modeling with the modified swat | Yes | Yes | Yes | Yes | Yes | Yes | Yes | Yes | Yes | Yes | Yes | Yes | Yes | Yes | Yes | Yes |
| 190 | effects of agricultural and urban land cover on new zealand's estuarine water quality | Yes | Yes | Yes | Yes | Yes | Yes | Yes | Yes | Yes | Yes | Yes | Yes | Yes | Yes | Yes | Yes |
| 191 | effects of agricultural land cover on water quality at the watershed scale in the lower kaskaskia river watershed | Yes | Yes | Yes | Yes | Yes | Yes | Yes | Yes | Yes | Yes | Yes | Yes | Yes | Yes | Yes | Yes |
| 192 | effects of agricultural management, land use, and watershed scale on e-coli concentrations in runoff and streamflow | Yes | Yes | Yes | Yes | Yes | Yes | Yes | Yes | Yes | Yes | Yes | Yes | Yes | Yes | Yes | Yes |
| 193 | effects of agricultural management, land use, and watershed scale on e. coli concentrations in runoff and streamflow | Yes | Yes | Yes | Yes | Yes | Yes | Yes | Yes | Yes | Yes | Yes | Yes | Yes | Yes | Yes | Yes |
| 194 | effects of anthropic actions and forest areas on a neotropical aquatic ecosystem | Yes | Yes | Yes | Yes | Yes | Yes | Yes | Yes | Yes | Yes | Yes | Yes | Yes | Yes | Yes | Yes |
| 195 | effects of bathing intensity, rainfall events, and location on the recreational water quality of stream pools in southern ecuador | No | No | No | No | No | Yes | No | No | No | No | No | No | No | No | No | No |
| 196 | effects of changing land use on the microbial water quality of tidal creeks | Yes | Yes | Yes | Yes | Yes | Yes | Yes | Yes | Yes | Yes | Yes | Yes | Yes | Yes | Yes | Yes |
| 197 | effects of future climate and land use scenarios on riverine source water quality | Yes | Yes | Yes | Yes | Yes | Yes | Yes | Yes | Yes | Yes | Yes | Yes | Yes | Yes | Yes | Yes |
| 198 | effects of human activities on rivers located in protected areas of the atlantic forest | Yes | Yes | Yes | Yes | Yes | Yes | Yes | No | Yes | Yes | Yes | Yes | Yes | Yes | Yes | Yes |
| 199 | effects of hurricanes, land use, and water management on nutrient and microbial pollution: st. lucie estuary, southeast florida | Yes | Yes | Yes | Yes | Yes | Yes | Yes | Yes | Yes | Yes | Yes | Yes | Yes | Yes | Yes | Yes |
| 200 | effects of hydrological regime and land use on in-stream escherichia coli concentration in the mekong basin, lao pdr | Yes | Yes | Yes | Yes | Yes | Yes | Yes | Yes | Yes | Yes | Yes | Yes | Yes | Yes | Yes | Yes |
| 201 | effects of land use and land cover changes on water quality in the umngeni river catchment, south africa | Yes | Yes | Yes | Yes | Yes | Yes | Yes | Yes | Yes | Yes | Yes | Yes | Yes | Yes | Yes | Yes |
| 202 | effects of land use and land cover on water quality of low-order streams in southeastern brazil: watershed versus riparian zone | Yes | Yes | Yes | Yes | Yes | Yes | Yes | Yes | Yes | Yes | Yes | Yes | Yes | Yes | Yes | Yes |
| 203 | effects of land use changes on water quality and flooding in upper nan river, thailand | Yes | Yes | Yes | Yes | Yes | Yes | Yes | Yes | Yes | Yes | Yes | No | Yes | No | Yes | Yes |
| 204 | effects of land use in the ohio river basin on the distribution of coliform and antibiotic resistant bacteria in the ohio river | Yes | Yes | Yes | Yes | Yes | Yes | Yes | Yes | Yes | Yes | Yes | Yes | Yes | Yes | Yes | Yes |
| 205 | effects of land use on the number of coliform bacteria in boyong river, sleman | Yes | Yes | Yes | Yes | Yes | Yes | Yes | Yes | Yes | Yes | Yes | Yes | Yes | Yes | Yes | Yes |
| 206 | effects of land uses on fecal indicator bacteria in the water and soil of a tropical watershed | Yes | Yes | Yes | Yes | Yes | Yes | Yes | Yes | Yes | Yes | Yes | Yes | Yes | Yes | Yes | Yes |
| 207 | effects of land-use change on benthic macroinvertebrates in the upper reaches of the apies-pienaar catchment | No | No | No | No | No | No | No | No | No | No | No | No | No | No | No | No |
| 208 | effects of low-impact-development (lid) practices on streamflow, runoff quantity, and runoff quality in the ipswich river basin, massachusetts: a summary of field and modeling studies | No | No | No | Yes | No | No | No | No | No | No | No | No | No | No | No | Yes |
| 209 | effects of shelter belts on fence-line pacing of deer and associated impacts on water and soil quality | No | Yes | No | No | No | Yes | Yes | No | Yes | No | No | No | No | Yes | No | No |
| 210 | effects of urbanisation on the quality of the urban runoff for delhi watershed | Yes | Yes | Yes | Yes | Yes | Yes | Yes | Yes | Yes | Yes | Yes | Yes | Yes | Yes | Yes | Yes |
| 211 | effects of urbanization on water quality and hydrology in the lower kaskaskia river watershed in southern illinois, united states | Yes | Yes | Yes | Yes | Yes | Yes | Yes | Yes | Yes | Yes | Yes | Yes | Yes | Yes | Yes | Yes |
| 212 | efficacy of bacteroides measurements for reducing the statistical uncertainty associated with hydrologic flow and fecal loads in a mixed use watershed | Yes | Yes | No | No | Yes | Yes | Yes | Yes | No | No | Yes | Yes | No | Yes | Yes | Yes |
| 213 | elucidating the effects of land cover and usage on background escherichia coli sources in edge-of-field runoff | Yes | Yes | Yes | Yes | Yes | Yes | Yes | Yes | Yes | Yes | Yes | Yes | Yes | Yes | Yes | Yes |
| 214 | enhancing britain's rivers: an interdisciplinary analysis of selected issues arising from implementation of the water framework directive | Yes | Yes | Yes | Yes | Yes | Yes | Yes | Yes | Yes | Yes | Yes | Yes | Yes | Yes | Yes | Yes |
| 215 | entire catchment and buffer zone approaches to modeling linkage between river water quality and land cover - a case study of yamaguchi prefecture, japan | No | No | No | No | Yes | Yes | No | Yes | Yes | No | No | No | Yes | Yes | No | No |
| 216 | environmental analysis of groundwater in mecosta county, michigan | No | No | No | No | No | No | No | No | No | No | No | No | No | No | No | No |
| 217 | environmental dna clarifies impacts of combined sewer overflows on the bacteriology of an urban river and resulting risks to public health | Yes | Yes | Yes | Yes | Yes | Yes | Yes | Yes | Yes | Yes | Yes | Yes | Yes | Yes | Yes | Yes |
| 218 | environmental factors controlling contamination of alternative water supply points in the lefock semi-urban watershed, cameroon western highlands | Yes | Yes | Yes | No | No | No | Yes | Yes | Yes | Yes | Yes | No | Yes | Yes | Yes | Yes |
| 219 | environmental fragility as an indicator of the risk of contamination by human action in watersheds used for public supply in western parana, brazil | Yes | Yes | Yes | Yes | Yes | Yes | Yes | Yes | Yes | Yes | Yes | Yes | Yes | Yes | Yes | Yes |
| 220 | environmental impacts on enterococcus in shem creek, south carolina, and characterization of changing land uses | Yes | Yes | Yes | Yes | Yes | Yes | Yes | Yes | Yes | Yes | Yes | Yes | Yes | Yes | Yes | Yes |
| 221 | environmental risk factors in the incidence of johnes disease | No | No | No | No | No | No | No | No | No | No | No | No | No | No | No | No |
| 222 | environmental variables likely influence the periphytic diatom community in a subtropical lotic environment | No | No | No | No | No | No | No | No | No | No | No | No | No | No | No | No |
| 223 | escherichia coli loading at or near base flow in a mixed-use watershed | No | No | No | No | No | No | No | No | No | No | No | No | No | No | No | No |
| 224 | escherichia coli reduction by bivalves in an impaired river impacted by agricultural land use | Yes | Yes | Yes | Yes | Yes | No | Yes | Yes | Yes | Yes | Yes | Yes | Yes | Yes | Yes | Yes |
| 225 | estimating daily potential e. coli loads in rural texas watersheds using spatially explicit load enrichment calculation tool (select) | Yes | Yes | Yes | Yes | Yes | Yes | Yes | Yes | Yes | Yes | Yes | Yes | Yes | Yes | Yes | Yes |
| 226 | estimating potential e. coli sources in a watershed using spatially explicit modeling techniques | Yes | Yes | Yes | Yes | Yes | Yes | Yes | Yes | Yes | Yes | Yes | Yes | Yes | Yes | Yes | Yes |
| 227 | estrogenic activity in the environment: municipal wastewater effluent, river, ponds, and wetlands | No | No | No | No | No | No | No | No | No | No | No | No | No | No | No | No |
| 228 | estuarine habitat quality reflects urbanization at large spatial scales in south carolina's coastal zone | Yes | Yes | Yes | Yes | Yes | Yes | Yes | Yes | Yes | Yes | Yes | Yes | Yes | Yes | Yes | Yes |
| 229 | evaluating land use impacts on water quality: perspectives for watershed management | Yes | Yes | Yes | Yes | Yes | Yes | Yes | Yes | Yes | Yes | Yes | Yes | Yes | Yes | Yes | Yes |
| 230 | evaluating the impacts of coastal development on the sinuosity and water quality of tidal creek headwaters in the southeast | Yes | Yes | Yes | Yes | Yes | Yes | Yes | Yes | Yes | Yes | Yes | Yes | No | Yes | Yes | Yes |
| 231 | evaluating the impacts of environmental and anthropogenic factors on water quality in the bumbu river watershed, papua new guinea | No | No | No | No | No | No | No | No | No | No | No | No | No | No | No | No |
| 232 | evaluating the influence of septic systems and watershed characteristics on stream faecal pollution in suburban watersheds in georgia, usa | Yes | Yes | Yes | No | Yes | Yes | Yes | Yes | Yes | Yes | Yes | Yes | Yes | Yes | Yes | Yes |
| 233 | evaluation and assessment of water quality in likangala river and its catchment area | No | No | No | No | No | No | No | No | No | No | No | No | No | No | No | No |
| 234 | evaluation of a multivariate analysis modeling approach identifying sources and patterns of nonpoint fecal pollution in a mixed use watershed | Yes | Yes | Yes | Yes | Yes | Yes | Yes | Yes | Yes | Yes | Yes | Yes | Yes | Yes | Yes | Yes |
| 235 | evaluation of land use and water quality in an agricultural watershed in the usa indicates multiple sources of bacterial impairment | Yes | Yes | Yes | Yes | Yes | Yes | Yes | Yes | Yes | Yes | Yes | Yes | Yes | Yes | Yes | Yes |
| 236 | evaluation of recirculating sand filter in a cold climate | Yes | Yes | Yes | Yes | Yes | Yes | No | No | No | No | No | No | Yes | No | Yes | Yes |
| 237 | evaluation of statistical models for predicting escherichia coli particle attachment in fluvial systems | No | Yes | No | No | Yes | No | No | No | No | No | Yes | No | No | No | No | No |
| 238 | evaluation of the distribution of fecal indicator bacteria in a river system depending on different types of land use in the southern watershed of the baltic sea | Yes | Yes | Yes | Yes | Yes | Yes | Yes | Yes | Yes | Yes | Yes | Yes | Yes | Yes | Yes | Yes |
| 243 | exploitation and management of natural resources by rural communities in the caete river basin in northern brazil | No | No | No | No | Yes | Yes | Yes | No | No | No | Yes | Yes | No | No | No | No |
| 245 | factors affecting surface-water and ground-water quality within tribal lands of eastern nebraska | Yes | Yes | Yes | Yes | Yes | Yes | Yes | Yes | Yes | Yes | Yes | Yes | Yes | Yes | Yes | Yes |
| 249 | factors influencing the bacteriological water quality in mountainous surface and groundwaters | No | No | No | No | No | No | No | No | No | No | No | No | No | No | No | No |
| 250 | factors related to occurrence and distribution of selected bacterial and protozoan pathogens in pennsylvania streams | Yes | Yes | Yes | Yes | Yes | Yes | Yes | Yes | Yes | Yes | Yes | Yes | Yes | Yes | Yes | Yes |
| 251 | faecal bacterial loads during flood events in northwestern mediterranean coastal rivers | No | Yes | No | No | No | No | No | No | No | No | No | No | No | No | No | No |
| 252 | faecal contamination of water and sediment in the rivers of the scheldt drainage network | Yes | Yes | Yes | Yes | Yes | Yes | Yes | Yes | Yes | Yes | Yes | Yes | Yes | Yes | Yes | Yes |
| 253 | faecal indicator organism concentrations and catchment export coefficients in the uk | Yes | Yes | Yes | Yes | Yes | Yes | Yes | Yes | Yes | Yes | Yes | Yes | Yes | Yes | Yes | Yes |
| 254 | faecal pollution source identification in an urbanising catchment using antibiotic resistance profiling, discriminant analysis and partial least squares regression | No | Yes | No | No | Yes | Yes | No | No | No | No | No | Yes | No | No | Yes | No |
| 255 | faecal-indicator concentrations in waters draining lowland pastoral catchments in the uk: relationships with land use and farming practices | Yes | Yes | Yes | Yes | Yes | Yes | Yes | Yes | Yes | Yes | Yes | Yes | Yes | Yes | Yes | Yes |
| 256 | fecal bacteria in the waters of an upland area in derbyshire, england: the influence of agricultural land use | Yes | Yes | Yes | Yes | Yes | Yes | Yes | Yes | Yes | Yes | Yes | Yes | Yes | Yes | Yes | Yes |
| 257 | fecal coliform concentrations in the upper cohansey river watershed predicted by air temperature, discharge, and land use | Yes | Yes | Yes | Yes | Yes | Yes | No | Yes | Yes | Yes | Yes | Yes | Yes | Yes | Yes | Yes |
| 258 | fecal coliform export from four coastal north carolina areas | Yes | Yes | Yes | Yes | Yes | Yes | Yes | Yes | Yes | Yes | Yes | Yes | Yes | Yes | Yes | Yes |
| 259 | fecal coliform predictive model using genetic algorithm-based radial basis function neural networks (ga-rbfnns) | Yes | Yes | Yes | Yes | Yes | Yes | Yes | Yes | Yes | Yes | Yes | Yes | Yes | Yes | Yes | Yes |
| 260 | fecal coliform source assessment in a small, mixed land use watershed | Yes | Yes | Yes | Yes | Yes | Yes | Yes | Yes | Yes | Yes | Yes | Yes | Yes | Yes | Yes | Yes |
| 261 | fecal contamination and high nutrient levels pollute the watersheds of wujiang, china | Yes | Yes | Yes | Yes | Yes | Yes | Yes | Yes | Yes | Yes | Yes | Yes | Yes | Yes | Yes | Yes |
| 262 | fecal contamination of water from a dog park and water potential changes affecting bacterial survival | Yes | Yes | No | Yes | Yes | No | Yes | Yes | Yes | Yes | Yes | Yes | Yes | Yes | No | No |
| 263 | fecal indicator bacteria removal by river networks | Yes | No | No | Yes | Yes | Yes | Yes | No | Yes | Yes | Yes | Yes | No | Yes | No | Yes |
| 264 | fecal indicator concentrations of surface runoff in rural watersheds, korea | Yes | Yes | Yes | Yes | Yes | Yes | Yes | Yes | Yes | Yes | Yes | Yes | Yes | No | Yes | Yes |
| 265 | fecal indicators and antibiotic resistance genes exhibit diurnal trends in the chattahoochee river: implications for water quality monitoring | Yes | Yes | Yes | Yes | Yes | Yes | Yes | Yes | Yes | Yes | Yes | Yes | Yes | Yes | Yes | Yes |
| 266 | fecal pollution source characterization in the surface waters of recharge and contributing zones of a karst aquifer using general and host-associated fecal genetic markers | Yes | Yes | Yes | Yes | Yes | Yes | Yes | Yes | Yes | Yes | Yes | Yes | Yes | Yes | Yes | Yes |
| 267 | fecal pollution source tracking toolbox for identification, evaluation and characterization of fecal contamination in receiving urban surface waters and groundwater | No | No | No | No | No | No | No | No | No | No | No | No | No | No | No | No |
| 268 | fecal sterol and runoff analysis for nonpoint source tracking | No | No | No | No | No | No | No | No | No | No | No | No | No | No | No | No |
| 269 | field and modelling studies of escherichia coli loads in tropical streams of montane agro-ecosystems | Yes | Yes | Yes | Yes | Yes | Yes | Yes | Yes | Yes | Yes | Yes | Yes | Yes | Yes | Yes | Yes |
| 270 | find, inform, and test (fit): a spatial modeling framework to estimate contributions of spatially distributed sources to microbial contaminants in the environment | Yes | No | No | No | Yes | Yes | Yes | Yes | No | Yes | No | Yes | No | Yes | Yes | Yes |
| 271 | fine-scale genetic structure in an eastern alpine black grouse tetrao tetrix metapopulation | No | No | No | No | No | No | No | No | No | No | No | No | No | No | No | No |
| 272 | fish assemblages and water quality in pampean streams (argentina) along an urbanization gradient | Yes | Yes | Yes | Yes | No | Yes | Yes | Yes | Yes | Yes | Yes | Yes | Yes | Yes | Yes | Yes |
| 273 | flow regulation by dams impacts more than land use on water quality and benthic communities in high-gradient streams in a semi-arid region | No | No | No | No | No | No | No | No | No | No | No | No | No | No | No | No |
| 281 | geochemical characterization and health risk assessment in two diversified environmental settings (southern italy) | Yes | Yes | Yes | Yes | Yes | Yes | Yes | Yes | Yes | Yes | Yes | Yes | Yes | Yes | Yes | Yes |
| 282 | geographic information of helminthiasis in thailand | No | No | No | No | No | No | No | No | No | No | No | No | No | No | No | No |
| 283 | geographic setting influences great lakes beach microbiological water quality | No | No | No | No | No | No | No | No | No | No | No | No | No | No | No | No |
| 284 | geospatial tools to inform land use change decisions: from local installation to watershed scale | No | No | No | No | Yes | No | No | No | No | No | No | Yes | No | No | No | No |
| 285 | geostatistical prediction of microbial water quality throughout a stream network using meteorology, land cover, and spatiotemporal autocorrelation | No | Yes | No | No | No | Yes | No | No | No | No | Yes | No | No | No | No | No |
| 286 | gis and artificial neural network-based water quality model for a stream network in the upper green river basin, kentucky, usa | No | No | No | No | No | No | No | No | No | No | No | No | No | No | No | No |
| 287 | grazing intensity is a poor indicator of waterborne escherichia coli o157 activity | Yes | Yes | Yes | Yes | Yes | Yes | Yes | Yes | Yes | Yes | Yes | Yes | Yes | Yes | Yes | Yes |
| 295 | high spatial resolution landscape indicators show promise in explaining water quality in urban streams | Yes | Yes | Yes | Yes | Yes | Yes | Yes | Yes | Yes | Yes | Yes | Yes | Yes | Yes | Yes | Yes |
| 296 | how can we improve understanding of faecal indicator dynamics in karst systems under changing climatic, population, and land use stressors? - research opportunities in sw china | No | No | No | No | Yes | Yes | No | Yes | No | No | Yes | No | Yes | No | No | No |
| 297 | how do different modalities of land use practices impact the environmental features and macroinvertebrates? an assessment of mountain streams from patagonia, argentina | Yes | Yes | Yes | Yes | Yes | Yes | Yes | Yes | Yes | Yes | Yes | Yes | Yes | Yes | Yes | Yes |
| 298 | hspf modeling of nonpoint sources in tickfaw river watershed | Yes | Yes | Yes | Yes | Yes | Yes | Yes | Yes | Yes | Yes | Yes | No | Yes | Yes | Yes | No |
| 299 | hubac and nifh source tracking markers display a relationship to land use but not rainfall | Yes | Yes | Yes | Yes | Yes | Yes | Yes | Yes | Yes | Yes | Yes | Yes | Yes | Yes | Yes | Yes |
| 300 | human and animal microbial source tracking in a tropical river with multiple land use activities | Yes | Yes | No | Yes | Yes | Yes | Yes | Yes | Yes | Yes | Yes | Yes | Yes | Yes | Yes | Yes |
| 301 | human development is linked to multiple water body impairments along the california coast | Yes | Yes | Yes | Yes | Yes | Yes | Yes | Yes | Yes | Yes | Yes | Yes | Yes | Yes | Yes | Yes |
| 302 | human fecal contamination corresponds to changes in the freshwater bacterial communities of a large river basin | No | No | No | No | No | No | No | No | No | No | No | No | No | No | No | No |
| 303 | human fecal pollution monitoring and microbial risk assessment for water reuse potential in a coastal industrial-residential mixed-use watershed | Yes | Yes | Yes | Yes | Yes | Yes | Yes | Yes | Yes | Yes | Yes | Yes | Yes | Yes | Yes | Yes |
| 304 | human health risk assessment and environmental distribution of trace elements, glyphosate, fecal coliform and total coliform in atlantic rainforest mountain rivers (south america) | Yes | No | No | Yes | Yes | Yes | Yes | No | No | No | Yes | Yes | Yes | Yes | No | No |
| 305 | human source identification by using a human-associated escherichia coli genetic marker in the mae klong river, thailand | No | No | No | No | No | No | No | No | No | No | No | No | No | No | No | No |
| 306 | human-dominated land use change in a phosphate mining area and its impact on the water environment | No | No | Yes | No | Yes | No | No | Yes | No | No | Yes | No | Yes | No | No | No |
| 307 | humans and hoofed livestock are the main sources of fecal contamination of rivers used for crop irrigation: a microbial source tracking approach | Yes | Yes | Yes | Yes | Yes | Yes | Yes | Yes | Yes | Yes | Yes | Yes | Yes | Yes | Yes | Yes |
| 308 | hydrochemical evidence of the depth of penetration of anthropogenic recharge in sandstone aquifers underlying two mature cities in the uk | Yes | Yes | Yes | Yes | Yes | Yes | Yes | Yes | Yes | No | Yes | Yes | Yes | Yes | Yes | Yes |
| 309 | hydrochemical indices as a proxy for assessing land-use impacts on water resources: a sustainable management perspective and case study of can tho city, vietnam | No | No | No | No | No | No | No | No | No | No | Yes | No | No | No | No | No |
| 310 | hydrochemistry of shallow groundwater and springs used for potable supply in southern brazil | Yes | Yes | Yes | Yes | Yes | Yes | Yes | Yes | Yes | Yes | Yes | Yes | Yes | Yes | Yes | Yes |
| 313 | hydrology, water quality, and channel morphology across an urban-rural land use gradient in western georgia, united states of america | Yes | Yes | Yes | Yes | Yes | Yes | Yes | Yes | Yes | Yes | Yes | Yes | Yes | Yes | Yes | Yes |
| 315 | identification and remediation of microbial contaminants in the headwaters of an agricultural watershed | No | No | No | No | No | Yes | No | Yes | No | No | No | Yes | Yes | No | No | Yes |
| 319 | impact of construction and functioning of a newly built ski slope on the quality of nearby stream water | Yes | Yes | Yes | Yes | Yes | Yes | Yes | No | Yes | Yes | Yes | No | Yes | Yes | Yes | Yes |
| 320 | impact of covid-19 lockdown on availability of drinking water in the arsenic-affected ganges river basin | No | No | No | No | No | No | No | No | No | No | No | No | No | No | No | No |
| 321 | impact of home industries on water quality in a tributary of the marimba river, harare: implications for urban water management | Yes | Yes | Yes | Yes | Yes | Yes | Yes | Yes | Yes | Yes | Yes | Yes | Yes | Yes | Yes | Yes |
| 322 | impact of land use and urban runoff on the contamination of the sarno river basin in southwestern italy | Yes | Yes | Yes | Yes | Yes | No | Yes | Yes | No | Yes | No | Yes | No | Yes | Yes | Yes |
| 323 | impact of land use on fecal coliform levels in surface waters of fairfax county, virginia | Yes | Yes | Yes | Yes | Yes | Yes | Yes | Yes | Yes | Yes | Yes | Yes | Yes | Yes | Yes | No |
| 324 | impact of land use on the faecal microbial quality of hill-country streams | Yes | Yes | Yes | Yes | Yes | Yes | Yes | Yes | Yes | Yes | Yes | Yes | Yes | Yes | Yes | Yes |
| 325 | impact of land use on water quality in the likangala catchment, southern malawi | Yes | Yes | Yes | Yes | Yes | Yes | Yes | Yes | Yes | Yes | Yes | Yes | Yes | Yes | Yes | Yes |
| 326 | impact of land uses, drought, flood, wildfire, and cascading events on water quality and microbial communities: a review and analysis | Yes | Yes | No | No | Yes | No | Yes | Yes | No | Yes | Yes | No | Yes | Yes | No | Yes |
| 327 | impact of land-use/land-cover dynamics on water quality in the upper lilongwe river basin, malawi | Yes | Yes | Yes | Yes | Yes | Yes | Yes | Yes | Yes | Yes | Yes | Yes | Yes | Yes | Yes | Yes |
| 328 | spatio-seasonal variation of water quality influenced by land use and land cover in lake muhazi | NA | NA | NA | NA | NA | NA | NA | NA | NA | NA | NA | NA | NA | NA | NA | NA |
| 329 | impact of urbanization and agriculture on the occurrence of bacterial pathogens and stx genes in coastal waterbodies of central california | Yes | Yes | Yes | Yes | Yes | Yes | Yes | Yes | Yes | Yes | Yes | Yes | Yes | Yes | Yes | Yes |
| 330 | impact of urbanization on the water quality of the uberaba river and tributaries. | Yes | Yes | Yes | Yes | Yes | Yes | Yes | Yes | Yes | Yes | Yes | Yes | Yes | Yes | Yes | Yes |
| 331 | impact on water quality of land uses along thamalakane-boteti river: an outlet of the okavango delta | Yes | Yes | Yes | Yes | Yes | No | Yes | Yes | Yes | Yes | Yes | Yes | Yes | Yes | Yes | Yes |
| 332 | impacts of droughts and heatwaves on river water quality worldwide | Yes | Yes | Yes | Yes | Yes | Yes | Yes | Yes | Yes | Yes | Yes | Yes | Yes | Yes | Yes | No |
| 333 | impacts of global change on the concentrations and dilution of combined sewer overflows in a drinking water source | No | No | No | No | No | No | No | No | No | No | No | No | No | No | No | No |
| 334 | impacts of land-based recreation on water quality | No | No | No | No | No | No | No | No | No | No | No | No | No | No | No | No |
| 335 | impacts of manure management practices on stream microbial loading into conesus lake, ny | Yes | Yes | Yes | Yes | Yes | Yes | Yes | Yes | Yes | Yes | Yes | Yes | Yes | Yes | Yes | Yes |
| 336 | impacts of tropical forest cover on water quality in agricultural watersheds in southeastern brazil | Yes | Yes | Yes | Yes | Yes | Yes | Yes | Yes | Yes | Yes | Yes | Yes | Yes | Yes | Yes | Yes |
| 337 | impacts of urbanization on the prevalence of antibiotic-resistant escherichia coli in the chaophraya river and its tributaries | Yes | Yes | Yes | Yes | Yes | Yes | Yes | Yes | Yes | Yes | Yes | Yes | Yes | Yes | Yes | Yes |
| 338 | implementation and integration of microbial source tracking in a river watershed monitoring plan | Yes | No | Yes | Yes | Yes | No | Yes | No | No | Yes | Yes | No | No | Yes | Yes | Yes |
| 339 | implementing landscape indices to predict stream water quality in an agricultural setting: an assessment of the lake and river enhancement (lare) protocol in the mississinewa river watershed, east-central indiana | No | No | No | No | No | No | No | No | No | No | No | No | No | No | No | No |
| 340 | implications of demographic changes and land transformations on surface water quality of rural and urban subbasins of upper bhima river basin, maharashtra, india | Yes | Yes | No | Yes | Yes | Yes | Yes | Yes | Yes | No | Yes | No | No | Yes | Yes | Yes |
| 341 | importance of land use factors in the prediction of water quality of the upper green river watershed, kentucky, usa, using random forest | Yes | Yes | Yes | Yes | Yes | Yes | Yes | Yes | Yes | Yes | Yes | Yes | Yes | Yes | Yes | Yes |
| 342 | improved management of farm dams increases vegetation cover, water quality, and macroinvertebrate biodiversity | Yes | Yes | Yes | No | Yes | Yes | No | No | No | No | Yes | No | Yes | Yes | Yes | Yes |
| 343 | improving the economic and environmental performance of a new zealand hill country farm catchment: 3. short-term outcomes of land-use change | Yes | Yes | Yes | Yes | Yes | Yes | Yes | Yes | Yes | Yes | Yes | Yes | Yes | Yes | Yes | Yes |
| 344 | in-stream escherichia coli modeling using high-temporal-resolution data with deep learning and process-based models | Yes | Yes | Yes | Yes | Yes | Yes | Yes | Yes | Yes | Yes | Yes | Yes | Yes | Yes | Yes | Yes |
| 345 | inadequate riparian zone use directly decreases water quality of a low-order urban stream in southern brazil | Yes | Yes | Yes | Yes | Yes | Yes | Yes | Yes | Yes | Yes | Yes | Yes | Yes | Yes | Yes | Yes |
| 346 | incorporating water quality into land use scenario analysis with random forest models | Yes | Yes | Yes | Yes | Yes | Yes | Yes | Yes | Yes | Yes | Yes | Yes | Yes | Yes | Yes | Yes |
| 347 | index of state trophic river basins ribeirao the big tank, guarulhos (sp): comparative analysis of rural areas and urban | Yes | Yes | Yes | Yes | Yes | No | Yes | Yes | Yes | Yes | No | No | No | No | Yes | Yes |
| 348 | indicator bacteria and associated water quality constituents in stormwater and snowmelt from four urban catchments | No | No | No | Yes | Yes | No | No | No | No | No | No | No | No | No | No | No |
| 349 | influence of climate change, tidal mixing, and watershed urbanization on historical water quality in newport bay, a saltwater wetland and tidal embayment in southern california | Yes | No | Yes | Yes | No | Yes | No | Yes | No | No | No | Yes | Yes | Yes | Yes | Yes |
| 350 | influence of climate variables on the concentration of escherichia coli in the rhine, meuse, and drentse aa during 1985-2010 | No | No | No | No | No | No | No | No | No | No | No | No | No | No | No | No |
| 351 | influence of intensive agriculture on benthic macroinvertebrate assemblages and water quality in the aconcagua river basin (central chile) | No | Yes | No | No | No | No | No | No | No | Yes | Yes | Yes | No | No | No | No |
| 352 | influence of land use and nutrient flux on metabolic activity of e. coli o157 in river water | Yes | Yes | Yes | Yes | Yes | Yes | Yes | Yes | Yes | Yes | Yes | Yes | Yes | Yes | Yes | Yes |
| 353 | influence of land use land cover on river water quality in rural north wales, uk | Yes | Yes | Yes | Yes | Yes | Yes | Yes | Yes | Yes | Yes | Yes | Yes | Yes | Yes | Yes | Yes |
| 354 | influence of land use on the chemistry and microbial abundance in groundwater | Yes | Yes | Yes | No | No | Yes | No | Yes | Yes | Yes | Yes | Yes | Yes | Yes | Yes | No |
| 355 | influence of land use on water quality in a tropical landscape: a multi-scale analysis | No | Yes | No | Yes | No | No | No | Yes | No | Yes | Yes | No | No | Yes | No | Yes |
| 356 | influence of physicochemical factors on bacterial communities along the lower mekong river assessed by illumina next-generation sequencing | No | Yes | No | No | Yes | Yes | No | No | No | No | No | No | No | Yes | No | No |
| 357 | influence of rainy season and land use on drinking water quality in a karst landscape, state of yucatan, mexico | Yes | Yes | Yes | No | No | Yes | Yes | No | Yes | No | Yes | No | Yes | Yes | No | No |
| 358 | influence of watershed land use on water quality in the state of santa catarina, brazil | No | No | Yes | No | Yes | No | No | Yes | No | Yes | No | Yes | No | Yes | Yes | No |
| 359 | influences of external factors on escherichia coli distribution, concentration, sources, and fate in secondary environments | Yes | Yes | Yes | Yes | Yes | Yes | Yes | Yes | Yes | Yes | Yes | Yes | Yes | Yes | Yes | Yes |
| 360 | influential factors in surface water quality in catchments within the pampa biome with different land use | Yes | Yes | Yes | Yes | Yes | Yes | Yes | Yes | Yes | Yes | No | Yes | Yes | Yes | Yes | Yes |
| 361 | inland lake indicator bacteria: long-term impervious surface and weather influences and a predictive bayesian model | No | No | No | No | No | No | Yes | No | No | No | No | No | No | Yes | No | No |
| 362 | innate immunity and stress physiology of eastern hellbenders (cryptobranchus alleganiensis) from two stream reaches with differing habitat quality | No | No | No | No | No | No | No | No | No | No | No | No | No | No | No | No |
| 363 | inputs of nutrients and fecal bacteria to freshwaters from irrigated agriculture: case studies in australia and new zealand | No | No | No | No | No | No | No | No | No | No | No | No | No | No | No | No |
| 364 | inside or outside: quantifying extrapolation across river networks | No | No | No | No | No | No | No | No | No | No | No | No | No | No | No | No |
| 365 | instream coliform gradients in the holtemme, a small headwater stream in the elbe river basin, northern germany | Yes | No | Yes | Yes | No | Yes | Yes | Yes | Yes | No | No | Yes | Yes | No | Yes | Yes |
| 366 | integral assessment of pollution in the suquia river (cordoba, argentina) as a contribution to lotic ecosystem restoration programs | Yes | Yes | Yes | Yes | Yes | Yes | Yes | Yes | Yes | Yes | Yes | Yes | Yes | Yes | Yes | Yes |
| 367 | integrated approach for quantitative estimation of particulate organic carbon sources in a complex river system | Yes | Yes | No | No | Yes | Yes | Yes | No | Yes | Yes | Yes | No | Yes | Yes | Yes | Yes |
| 368 | integrating environmental and socio-economic indicators of a linked catchment-coastal system using variable environmental intensity | Yes | No | Yes | Yes | Yes | Yes | Yes | Yes | Yes | Yes | Yes | Yes | Yes | No | Yes | Yes |
| 369 | integration of remote sensing data and in situ measurements to monitor the water quality of the ismailia canal, nile delta, egypt | Yes | Yes | Yes | Yes | Yes | Yes | Yes | Yes | Yes | Yes | Yes | Yes | Yes | Yes | Yes | Yes |
| 370 | integrative survey of 68 non-overlapping upstate new york watersheds reveals stream features associated with aquatic fecal contamination | Yes | Yes | Yes | Yes | Yes | Yes | Yes | Yes | Yes | Yes | Yes | Yes | Yes | Yes | Yes | Yes |
| 371 | intra-event variability of bacterial composition in stormwater runoff from mixed land use and land cover catchment | Yes | Yes | Yes | Yes | Yes | No | Yes | No | Yes | Yes | Yes | Yes | Yes | Yes | Yes | Yes |
| 372 | investigating escherichia coli in a mixed land-use watershed in west virginia, usa | Yes | Yes | Yes | Yes | Yes | Yes | Yes | Yes | Yes | Yes | Yes | Yes | Yes | Yes | Yes | Yes |
| 373 | investigating landscape-stream water quality relationships and stream water quality preservation strategies in the texas gulf region using a hybrid of machine learning and hydrological modeling approach | Yes | Yes | Yes | Yes | No | Yes | Yes | Yes | Yes | Yes | Yes | No | Yes | Yes | Yes | Yes |
| 374 | investigating the relationship between surface water pollution and onsite wastewater treatment systems | No | No | No | Yes | Yes | No | No | No | No | No | Yes | Yes | Yes | No | Yes | No |
| 379 | lake tuscaloosa and the north river: an analysis of, and plans to improve, water quality | No | Yes | No | No | Yes | No | Yes | No | No | Yes | No | Yes | Yes | No | No | Yes |
| 384 | land use and monitoring of water resources of ipe stream, ilha solteira, sp | Yes | Yes | Yes | Yes | Yes | Yes | Yes | Yes | Yes | Yes | Yes | Yes | Yes | Yes | Yes | Yes |
| 385 | land use and water quality in a rural cloud forest region (intag, ecuador) | Yes | Yes | Yes | Yes | Yes | Yes | Yes | Yes | Yes | Yes | Yes | Yes | Yes | Yes | Yes | Yes |
| 386 | land use and water quality in guangzhou, china: a survey of ecological and social vulnerability in four urban units of the rapidly developing megacity | Yes | Yes | Yes | Yes | Yes | Yes | Yes | Yes | Yes | Yes | Yes | Yes | Yes | No | Yes | Yes |
| 387 | land use and water quality relationships in the lower little bow river watershed, alberta, canada | No | No | No | No | No | No | No | No | No | No | No | No | No | No | No | No |
| 388 | land use as a critical determinant of faecal and antimicrobial resistance gene pollution in riverine systems | Yes | Yes | Yes | Yes | Yes | Yes | Yes | Yes | Yes | Yes | Yes | Yes | Yes | Yes | Yes | Yes |
| 389 | land use effects on water quality in the urban agglomeration of cuiaba and varzea grande, mato grosso state, central brazil | Yes | Yes | Yes | Yes | Yes | Yes | Yes | Yes | Yes | No | Yes | Yes | Yes | Yes | Yes | Yes |
| 390 | land use impact on the water quality of large tropical river: mun river basin, thailand | Yes | Yes | Yes | Yes | Yes | Yes | Yes | Yes | Yes | Yes | Yes | Yes | Yes | Yes | Yes | Yes |
| 391 | land use impacts on parasitic infection: a cross-sectional epidemiological study on the role of irrigated agriculture in schistosome infection in a dammed landscape | No | No | No | No | No | No | No | No | No | No | No | No | No | No | No | No |
| 392 | land use land cover changes in detection of water quality: a study based on remote sensing and multivariate statistics | Yes | Yes | Yes | Yes | Yes | Yes | Yes | Yes | Yes | Yes | Yes | Yes | Yes | Yes | Yes | Yes |
| 393 | land use practices and elevated levels of escherichia coli in the coosawattee river, georgia | Yes | Yes | Yes | Yes | Yes | Yes | Yes | Yes | Yes | Yes | Yes | Yes | Yes | Yes | Yes | Yes |
| 394 | land use, weather, and water quality factors associated with fecal contamination of northeastern streams that span an urban-rural gradient | Yes | Yes | Yes | Yes | Yes | Yes | Yes | Yes | Yes | Yes | Yes | Yes | Yes | Yes | Yes | Yes |
| 395 | land-use & water quality in the headwaters of the alafia river watershed | Yes | Yes | Yes | Yes | Yes | Yes | Yes | Yes | Yes | Yes | Yes | Yes | Yes | Yes | Yes | Yes |
| 396 | land-use change caused microbial pollution in a karst underground river, chongqing, china | No | No | Yes | No | Yes | No | Yes | Yes | No | No | No | No | Yes | Yes | No | No |
| 397 | land-use impact on water quality of the opak sub-watershed, yogyakarta, indonesia | Yes | Yes | Yes | Yes | Yes | Yes | Yes | Yes | Yes | Yes | Yes | Yes | Yes | Yes | Yes | Yes |
| 398 | land-use impacts and water quality targets in the intensive dairying catchment of the toenepi stream, new zealand | No | Yes | Yes | No | No | Yes | No | No | Yes | Yes | Yes | No | No | Yes | No | No |
| 399 | land-use related changes to sedimentary organic matter in tidal creeks of the northern gulf of mexico | No | Yes | No | No | No | No | No | Yes | No | Yes | Yes | No | Yes | Yes | No | No |
| 400 | land-use-mediated escherichia coli concentrations in a contemporary appalachian watershed | Yes | Yes | Yes | Yes | Yes | Yes | Yes | Yes | Yes | Yes | Yes | Yes | Yes | Yes | Yes | Yes |
| 401 | land-use/land-cover and water qualty in the cape fear river basin, north carolina: spatial-temporal relationships | Yes | Yes | Yes | Yes | Yes | Yes | Yes | Yes | Yes | Yes | Yes | Yes | Yes | Yes | Yes | Yes |
| 402 | landscape and seasonal factors influence salmonella and campylobacter prevalence in a rural mixed use watershed | Yes | Yes | No | Yes | Yes | Yes | No | Yes | Yes | Yes | Yes | No | Yes | No | Yes | Yes |
| 403 | landscape drivers and social dynamics shaping microbial contamination risk in three maya communities in southern belize, central america | Yes | Yes | Yes | Yes | Yes | Yes | Yes | Yes | Yes | Yes | Yes | Yes | Yes | Yes | Yes | Yes |
| 404 | landscape genetics of northern bobwhite and swamp rabbits in illinois | No | No | No | No | No | No | No | No | No | No | No | No | No | No | No | No |
| 405 | landscape-scale factors affecting the prevalence of escherichia coli in surface soil include land cover type, edge interactions, and soil ph | Yes | Yes | Yes | Yes | Yes | Yes | Yes | Yes | Yes | Yes | Yes | Yes | Yes | Yes | Yes | Yes |
| 406 | large-scale implementation of standardized quantitative real-time pcr fecal source identification procedures in the tillamook bay watershed | Yes | Yes | Yes | Yes | Yes | Yes | Yes | Yes | Yes | Yes | Yes | Yes | Yes | Yes | Yes | Yes |
| 407 | learning hierarchical bayesian networks to assess the interaction effects of controlling factors on spatiotemporal patterns of fecal pollution in streams | Yes | Yes | Yes | Yes | Yes | Yes | Yes | Yes | Yes | Yes | Yes | Yes | Yes | Yes | Yes | Yes |
| 408 | level and transport pattern of faecal coliform bacteria from tropical urban catchments | Yes | Yes | Yes | No | Yes | Yes | Yes | Yes | Yes | Yes | Yes | Yes | Yes | Yes | Yes | Yes |
| 409 | levels and patterns of fecal indicator bacteria in stormwater runoff from homogenous land use sites and urban watersheds | Yes | Yes | Yes | Yes | Yes | Yes | Yes | Yes | Yes | Yes | Yes | Yes | Yes | Yes | Yes | Yes |
| 410 | linkages between tidal creek ecosystems and the landscape and demographic attributes of their watersheds | Yes | Yes | Yes | Yes | Yes | Yes | Yes | Yes | Yes | Yes | Yes | Yes | Yes | Yes | Yes | Yes |
| 411 | linking fecal bacteria in rivers to landscape, geochemical, and hydrologic factors and sources at the basin scale | No | No | No | Yes | No | No | No | Yes | No | No | Yes | Yes | Yes | No | Yes | Yes |
| 412 | linking land-use type and stream water quality using spatial data of fecal indicator bacteria and heavy metals in the yeongsan river basin | Yes | Yes | Yes | Yes | Yes | Yes | Yes | Yes | Yes | Yes | Yes | Yes | Yes | Yes | Yes | Yes |
| 418 | mapping the potential risk of escherichia coli leaching through soils of the waikato river catchment, new zealand | Yes | Yes | Yes | Yes | Yes | Yes | Yes | Yes | Yes | Yes | Yes | Yes | Yes | Yes | Yes | Yes |
| 419 | mapping the spatial distribution of the rumen fluke calicophoron daubneyi in a mediterranean area | No | No | No | No | No | No | No | No | No | No | No | No | No | No | No | No |
| 420 | mapping the spatial distribution of water quality as a function of land use and occupation and rainfall in the para river basin, mg | No | Yes | Yes | Yes | No | Yes | No | No | No | No | Yes | No | No | Yes | Yes | No |
| 421 | measurement and modeling of denitrification in sand-bed streams under various land uses | No | No | No | No | No | No | No | No | No | No | No | No | No | No | No | No |
| 422 | microarray assessment of virulence, antibiotic, and heavy metal resistance in an agricultural watershed creek | No | No | No | No | No | No | No | No | No | No | No | No | No | No | No | No |
| 423 | microbial find, inform, and test model for identifying spatially distributed contamination sources: framework foundation and demonstration of ruminant bacteroides abundance in river sediments | Yes | Yes | No | Yes | Yes | Yes | Yes | Yes | No | Yes | Yes | Yes | Yes | Yes | No | No |
| 424 | microbial pollution source identification in rural / urban mixed watersheds | No | No | No | No | No | No | No | No | No | No | No | No | No | No | No | No |
| 425 | microbial source tracking (mst) in chattahoochee river national recreasion area: seasonal and precipitation in mst marker concentrations, and associations with e. coli levels, pathogenic marker presence, and land use | Yes | Yes | Yes | No | Yes | Yes | Yes | Yes | Yes | Yes | Yes | Yes | Yes | Yes | Yes | Yes |
| 426 | microbial source tracking to elucidate the impact of land-use and physiochemical water quality on fecal contamination in a mixed land-use watershed | Yes | Yes | Yes | Yes | Yes | Yes | Yes | Yes | Yes | Yes | Yes | Yes | Yes | Yes | Yes | Yes |
| 427 | microbial source tracking: a forensic technique for microbial source identification? | No | No | No | No | Yes | No | No | No | Yes | No | Yes | No | No | Yes | No | No |
| 428 | microbial source-tracking reveals origins of fecal contamination in a recoveringwatershed | Yes | Yes | Yes | Yes | Yes | Yes | Yes | Yes | Yes | Yes | Yes | Yes | Yes | No | No | Yes |
| 429 | microbial water pollution: a screening tool for initial catchment-scale assessment and source apportionment | Yes | Yes | Yes | Yes | Yes | Yes | Yes | Yes | Yes | Yes | Yes | Yes | Yes | Yes | Yes | Yes |
| 430 | microbial water quality and influences of fecal accumulation from a dog exercise area | Yes | Yes | Yes | Yes | Yes | Yes | Yes | Yes | Yes | Yes | Yes | Yes | Yes | Yes | Yes | Yes |
| 431 | microbiological quality assessment of watershed associated with animal-based agriculture in santa catarina, brazil | Yes | Yes | Yes | Yes | Yes | Yes | Yes | Yes | Yes | Yes | Yes | Yes | Yes | Yes | Yes | Yes |
| 432 | mitochondrial activity in fern spores of cyathea costaricensis as an indicator of the impact of land use and water quality in rivers running through cloud forests | No | No | No | No | No | No | No | No | No | No | No | No | No | No | No | No |
| 433 | model-based analysis of the potential of macroinvertebrates as indicators for microbial pathogens in rivers | No | No | No | No | No | No | No | No | No | No | No | No | No | No | No | No |
| 434 | modeling bacteria fate and transport in watersheds to support tmdls | No | No | No | No | No | No | No | No | No | No | No | No | No | No | No | No |
| 435 | modeling spatiotemporal bacterial variability with meteorological and watershed land-use characteristics | Yes | Yes | Yes | Yes | Yes | Yes | Yes | Yes | Yes | Yes | Yes | Yes | Yes | Yes | Yes | Yes |
| 436 | modeling the dispersion of e. coli in waterbodies due to urban sources: a spatial approach | Yes | Yes | Yes | Yes | Yes | Yes | Yes | Yes | Yes | Yes | Yes | Yes | Yes | Yes | Yes | Yes |
| 437 | modeling the impact of land use change on basin-scale transfer of fecal indicator bacteria: swat model performance | Yes | Yes | Yes | Yes | Yes | Yes | Yes | Yes | Yes | Yes | Yes | Yes | Yes | Yes | Yes | Yes |
| 438 | modeling the relationship between land use and surface water quality | Yes | Yes | Yes | Yes | Yes | Yes | Yes | Yes | Yes | Yes | Yes | Yes | Yes | Yes | Yes | Yes |
| 439 | modelling faecal bacteria pathways in receiving waters | Yes | Yes | Yes | Yes | Yes | No | Yes | Yes | Yes | Yes | Yes | Yes | No | Yes | No | Yes |
| 440 | modelling faecal indicator concentrations in large rural catchments using land use and topographic data | Yes | Yes | Yes | Yes | Yes | Yes | Yes | Yes | Yes | Yes | Yes | Yes | Yes | Yes | Yes | Yes |
| 441 | modelling microbiological water quality in the seine river drainage network: past, present and future situations | Yes | Yes | Yes | Yes | Yes | Yes | Yes | Yes | Yes | Yes | Yes | Yes | Yes | Yes | Yes | Yes |
| 442 | modelling of faecal indicator bacteria (fib) in the red river basin (vietnam) | Yes | Yes | Yes | No | Yes | Yes | Yes | Yes | Yes | Yes | Yes | Yes | Yes | Yes | Yes | Yes |
| 443 | modelling the hydrologic effects of land-use and climate changes | Yes | Yes | Yes | Yes | No | Yes | Yes | Yes | Yes | Yes | Yes | Yes | Yes | Yes | Yes | Yes |
| 444 | modelling the impact of future socio-economic and climate change scenarios on river microbial water quality | Yes | No | Yes | Yes | Yes | Yes | Yes | Yes | Yes | Yes | Yes | Yes | Yes | Yes | Yes | Yes |
| 445 | modelling the seasonal impacts of a wastewater treatment plant on water quality in a mediterranean stream using microbial indicators | No | No | No | No | No | No | No | No | No | No | No | No | No | No | No | No |
| 446 | models of total and presumed wildlife sources of fecal coliform bacteria in coastal ponds | No | No | No | No | No | No | Yes | No | No | No | No | No | No | No | No | No |
| 447 | molecular tracers of soot and sewage contamination in streams supplying new york city drinking water | Yes | Yes | Yes | Yes | No | Yes | Yes | Yes | Yes | Yes | Yes | Yes | Yes | Yes | No | Yes |
| 448 | monitoring and evaluation of the water quality of the lower neches river, texas, usa | Yes | Yes | Yes | Yes | No | Yes | Yes | Yes | No | Yes | Yes | Yes | Yes | Yes | Yes | Yes |
| 449 | monitoring and predicting the fecal indicator bacteria concentrations from agricultural, mixed land use and urban stormwater runoff | Yes | Yes | Yes | Yes | Yes | Yes | Yes | Yes | Yes | Yes | Yes | Yes | Yes | Yes | Yes | Yes |
| 450 | monitoring bacterial indicators of water quality in a tidally influenced delta: a sisyphean pursuit | No | No | No | Yes | No | No | No | No | Yes | No | No | No | Yes | No | Yes | No |
| 451 | monitoring of spunky bottoms restored wetland in southern illinois for biotic and abiotic pollution indicators | Yes | Yes | Yes | Yes | Yes | Yes | Yes | Yes | Yes | Yes | Yes | Yes | Yes | Yes | Yes | Yes |
| 452 | motueka river plume facilitates transport of ruminant faecal contaminants into shellfish growing waters, tasman bay, new zealand | Yes | No | Yes | No | Yes | Yes | Yes | Yes | Yes | Yes | Yes | Yes | Yes | Yes | Yes | No |
| 453 | multi-scale landscape factors influencing stream water quality in the state of oregon | Yes | Yes | Yes | Yes | Yes | Yes | Yes | Yes | Yes | Yes | Yes | Yes | Yes | Yes | Yes | Yes |
| 454 | multi-year microbial source tracking study characterizing fecal contamination in an urban watershed | No | No | No | No | No | No | No | No | No | No | No | No | No | No | No | No |
| 455 | multiple modes of water quality impairment by fecal contamination in a rapidly developing coastal area: southwest brunswick county, north carolina | Yes | Yes | Yes | Yes | Yes | Yes | Yes | Yes | No | Yes | Yes | Yes | Yes | Yes | Yes | Yes |
| 461 | occurrence and distribution of fecal indicator bacteria with respect to urban and rural land uses | Yes | Yes | Yes | Yes | Yes | Yes | Yes | Yes | Yes | Yes | Yes | Yes | Yes | Yes | Yes | Yes |
| 466 | optimization of the water quality monitoring network in a basin with intensive agriculture using artificial intelligence algorithms | No | No | No | No | No | No | No | No | No | No | Yes | No | No | No | No | No |
| 471 | patterns in water quality on canadian shores of lake ontario: correspondence with proximity to land and level of urbanization | Yes | No | Yes | Yes | Yes | Yes | Yes | Yes | No | No | Yes | Yes | Yes | Yes | Yes | Yes |
| 472 | patterns of genetic diversity in african forest elephants living in a human-modified landscape in southwest gabon | No | No | No | No | No | No | No | No | No | No | No | No | No | No | No | No |
| 473 | patterns of host-associated fecal indicators driven by hydrology, precipitation, and land use attributes in great lakes watersheds | Yes | Yes | Yes | Yes | Yes | Yes | Yes | Yes | Yes | Yes | Yes | Yes | Yes | No | Yes | Yes |
| 474 | phosphorus and thermotolerant coliforms's loads in brazilian watersheds with limited data: considerations on the integrated analysis of water quality and quantity | No | No | No | No | No | No | No | No | No | No | No | No | No | No | No | No |
| 475 | physical, chemical, and microbial quality of floodwaters in houston following hurricane harvey | No | No | No | No | No | No | No | No | No | No | No | No | No | No | No | No |
| 476 | physico-chemical and biological characteristics of mountainous streams under different land uses of mid hills of himachal pradesh | Yes | Yes | Yes | Yes | Yes | Yes | Yes | Yes | Yes | Yes | Yes | Yes | Yes | Yes | Yes | Yes |
| 477 | physiographic environment classification: a controlling factor classification of landscape susceptibility to waterborne contaminant loss | No | No | Yes | Yes | No | No | No | Yes | No | No | No | No | No | No | No | No |
| 478 | pine afforestation and stream health: a comparison of land-use in two soft rock catchments, east cape, new zealand | Yes | Yes | Yes | No | Yes | Yes | Yes | Yes | Yes | Yes | Yes | Yes | Yes | Yes | Yes | No |
| 479 | point and non-point microbial source pollution: a case study of delhi | Yes | Yes | Yes | Yes | Yes | Yes | Yes | Yes | Yes | Yes | Yes | Yes | Yes | Yes | Yes | Yes |
| 480 | pollution in qaraaoun lake, central lebanon | Yes | Yes | Yes | Yes | Yes | Yes | Yes | Yes | Yes | Yes | Yes | Yes | Yes | No | Yes | Yes |
| 481 | population dynamics and genetic variability of escherichia coli in a mixed land-use watershed | No | No | Yes | No | Yes | No | No | No | No | No | No | No | No | No | Yes | No |
| 482 | population genetics and structure of the sumatran tiger | No | No | No | No | No | No | No | No | No | No | No | No | No | No | No | No |
| 483 | population growth, land use and land cover transformations, and water quality nexus in the upper ganga river basin | No | No | No | No | No | No | Yes | No | No | No | No | No | No | No | No | No |
| 484 | potential impacts of climate and land use change on the water quality of ganga river around the industrialized kanpur region | Yes | Yes | Yes | Yes | Yes | Yes | Yes | Yes | Yes | Yes | Yes | Yes | Yes | Yes | Yes | Yes |
| 485 | potential pollutant sources in a choptank river (usa) subwatershed and the influence of land use and watershed characteristics | Yes | Yes | Yes | Yes | Yes | Yes | Yes | Yes | Yes | Yes | Yes | Yes | Yes | No | Yes | Yes |
| 486 | potential sources of ammonium-nitrogen in the coastal groundwater determined from a combined analysis of nitrogen isotope, biological and geological parameters, and land use | No | No | No | No | No | No | No | Yes | Yes | Yes | No | No | No | No | No | No |
| 487 | precipitation-driven anthropogenic pollutant fluctuations within standing water sources of the edwards aquifer region, texas | Yes | Yes | Yes | Yes | Yes | Yes | Yes | Yes | Yes | Yes | Yes | Yes | No | No | Yes | Yes |
| 488 | predicting diffuse microbial pollution risk across catchments: the performance of scimap and recommendations for future development | Yes | Yes | Yes | Yes | Yes | Yes | Yes | Yes | Yes | Yes | Yes | Yes | Yes | Yes | Yes | Yes |
| 489 | predicting faecal indicator fluxes using digital land use data in the uk's sentinel water framework directive catchment: the ribble study | Yes | Yes | Yes | Yes | Yes | Yes | Yes | Yes | Yes | Yes | Yes | Yes | Yes | Yes | Yes | Yes |
| 490 | predicting fecal indicator bacteria using spatial stream network models in a mixed-land-use suburban watershed in new jersey, usa | Yes | Yes | Yes | Yes | Yes | Yes | Yes | Yes | Yes | Yes | Yes | Yes | Yes | Yes | Yes | Yes |
| 491 | predicting fecal indicator organism contamination in oregon coastal streams | Yes | Yes | Yes | Yes | Yes | Yes | Yes | Yes | Yes | Yes | Yes | Yes | Yes | Yes | Yes | Yes |
| 492 | predicting gene flow corridors and wildlife health using landscape genomics and non-invasive metagenomic monitoring: investigation of elk (cervus canadensis) in the greater yellowstone ecosystem | No | No | No | No | No | No | No | No | No | No | No | No | No | No | No | No |
| 493 | predicting in-stream water quality constituents at the watershed scale using machine learning | No | No | No | No | No | No | No | No | No | Yes | No | No | No | No | No | No |
| 494 | predicting microbial pollution concentrations in uk rivers in response to land use change | Yes | Yes | Yes | Yes | Yes | Yes | Yes | Yes | Yes | Yes | Yes | Yes | Yes | Yes | Yes | Yes |
| 495 | predicting stream water quality under different urban development pattern scenarios with an interpretable machine learning approach | Yes | Yes | No | Yes | No | Yes | No | Yes | Yes | Yes | Yes | Yes | No | Yes | Yes | No |
| 496 | predicting the fate and transport of e. coli in two texas river basins using a spatially referenced regression model | Yes | Yes | Yes | Yes | Yes | Yes | Yes | Yes | Yes | Yes | Yes | Yes | Yes | Yes | Yes | Yes |
| 497 | predictive models may complement or provide an alternative to existing strategies for assessing the enteric pathogen contamination status of northeastern streams used to provide water for produce production | No | No | No | No | No | No | No | No | No | No | No | No | No | No | No | No |
| 498 | prevalence and seasonal dynamics of blactx-m antibiotic resistance genes and fecal indicator organisms in the lower lahn river, germany | No | No | No | No | No | No | No | No | No | Yes | Yes | No | No | No | No | No |
| 500 | quality of surface water in missouri, water year 2020 | Yes | Yes | No | No | Yes | No | Yes | Yes | Yes | Yes | No | No | No | Yes | Yes | No |
| 501 | quality of surface water in missouri, water year 2021 | No | No | No | No | No | No | No | No | No | No | No | No | Yes | No | No | No |
| 505 | quantification of poultry and human fecal contamination in the tidal creeks of the virginia eastern shore using a multifaceted edna method | Yes | Yes | Yes | Yes | Yes | Yes | Yes | Yes | Yes | Yes | Yes | Yes | Yes | Yes | Yes | Yes |
| 507 | quantifying the contribution of riparian soils to the provision of ecosystem services | No | No | No | No | No | No | No | No | No | No | No | No | No | No | No | No |
| 508 | quantifying the effect of overland flow on escherichia coli pulses during floods: use of a tracer-based approach in an erosion-prone tropical catchment | No | No | No | No | No | No | No | No | No | No | No | No | No | No | No | No |
| 509 | quantifying the variability in escherichia coli (e. coli) throughout storm events at a karst spring in northwestern arkansas, united states | Yes | Yes | Yes | Yes | Yes | Yes | Yes | Yes | Yes | Yes | Yes | Yes | Yes | Yes | Yes | Yes |
| 510 | quantitative multi-year elucidation of fecal sources of waterborne pathogen contamination in the south nation river basin using bacteroidales microbial source tracking markers | No | No | No | No | No | No | No | No | No | No | No | No | No | No | No | No |
| 514 | real time characterization and modeling of escherichia coli contamination in urban streams | Yes | Yes | Yes | Yes | Yes | No | Yes | Yes | Yes | Yes | Yes | Yes | Yes | Yes | Yes | Yes |
| 517 | regional assessment of concentrations and sources of pharmaceutically active compounds, pesticides, nitrate, and e. coli in post-glacial aquifer environments (canada) | No | No | No | No | No | No | No | Yes | No | No | No | No | No | No | No | No |
| 518 | regional variations of bovine and porcine fecal pollution as a function of landscape, nutrient, and hydrological factors | Yes | Yes | Yes | Yes | Yes | Yes | Yes | Yes | Yes | Yes | Yes | Yes | Yes | Yes | Yes | Yes |
| 519 | relating watershed characteristics to elevated stream escherichia coli levels in agriculturally dominated landscapes: an iowa case study | Yes | Yes | Yes | Yes | Yes | Yes | Yes | Yes | Yes | Yes | Yes | Yes | Yes | Yes | Yes | Yes |
| 520 | relations of the groundwater quality and disorderly occupation in an amazon low-income neighborhood developed over a former dump area, santarem/pa, brazil | Yes | No | Yes | Yes | No | Yes | Yes | Yes | No | No | Yes | Yes | Yes | Yes | No | Yes |
| 521 | relationship between land use and surface water quality in a rapidly developing watershed in southeast louisiana | Yes | Yes | Yes | Yes | Yes | Yes | Yes | Yes | Yes | Yes | Yes | Yes | Yes | Yes | Yes | Yes |
| 522 | relationship between land use and water quality in a watershed impacted by iron ore tailings and domestic sewage | Yes | No | No | Yes | Yes | No | Yes | Yes | No | Yes | Yes | Yes | Yes | Yes | No | No |
| 523 | relationships between intra-aggregate pore structures and distributions of escherichia coli within soil macro-aggregates | Yes | Yes | Yes | Yes | Yes | Yes | Yes | Yes | Yes | Yes | Yes | Yes | Yes | Yes | Yes | Yes |
| 524 | relationships between land use and mercury contamination in twelve tributaries of the lake st. francis region of the st. lawrence river near cornwall, ontario | No | No | No | No | No | No | No | No | No | No | No | No | No | No | No | No |
| 525 | relationships between land use patterns and water quality in the pong river basin, northeast thailand | Yes | Yes | Yes | Yes | Yes | Yes | Yes | Yes | Yes | Yes | Yes | Yes | Yes | Yes | Yes | Yes |
| 526 | respective contributions of point and non-point sources of e. coli and enterococci in a large urbanized watershed (the seine river, france) | Yes | Yes | Yes | Yes | Yes | Yes | Yes | Yes | Yes | Yes | Yes | Yes | Yes | Yes | Yes | Yes |
| 527 | responses of stream macroinvertebrate communities and water quality of five dairy farming streams following adoption of mitigation practices | Yes | Yes | Yes | Yes | Yes | Yes | Yes | Yes | Yes | Yes | Yes | Yes | Yes | Yes | Yes | Yes |
| 528 | riparian buffers: disrupting the transport of e. coli from rural catchments to streams | Yes | Yes | Yes | Yes | Yes | Yes | Yes | Yes | Yes | Yes | Yes | Yes | Yes | Yes | Yes | Yes |
| 529 | riparian protection and on-farm best management practices for restoration of a lowland stream in an intensive dairy farming catchment: a case study | Yes | Yes | Yes | Yes | Yes | Yes | Yes | Yes | Yes | Yes | Yes | Yes | Yes | Yes | Yes | Yes |
| 530 | river water quality in new zealand: an introduction and overview | No | No | No | No | No | No | No | No | No | No | No | No | No | No | No | No |
| 531 | river water quality modelling for river basin and water resources management | Yes | Yes | Yes | Yes | Yes | Yes | Yes | Yes | Yes | Yes | Yes | Yes | Yes | Yes | Yes | Yes |
| 532 | roadside ditches as conduits of fecal indicator organisms and sediment: implications for water quality management | Yes | Yes | Yes | Yes | Yes | Yes | Yes | Yes | Yes | Yes | Yes | Yes | Yes | Yes | Yes | Yes |
| 538 | searching for balance between hill country pastoral farming and nature | Yes | Yes | Yes | Yes | Yes | Yes | Yes | Yes | Yes | Yes | Yes | Yes | Yes | Yes | Yes | Yes |
| 539 | seasonal variation of surface water quality and streamflow in rispana: a tributary of ganges river, india | Yes | Yes | Yes | Yes | Yes | Yes | Yes | Yes | Yes | Yes | Yes | Yes | Yes | Yes | Yes | Yes |
| 540 | seasonality, richness and prevalence of intestinal parasites of three neotropical primates (alouatta seniculus, ateles hybridus and cebus versicolor) in a fragmented forest in colombia | No | No | No | No | No | No | No | No | No | No | No | No | No | No | No | No |
| 541 | sediment and fecal indicator bacteria loading in a mixed land use watershed: contributions from suspended sediment and bedload transport | No | No | No | No | No | No | No | No | No | No | No | No | No | No | No | No |
| 542 | semi-quantitative evaluation of fecal contamination potential by human and ruminant sources using multiple lines of evidence | No | No | Yes | No | No | No | No | No | Yes | No | Yes | No | No | Yes | No | Yes |
| 543 | sensitivity of streamflow and microbial water quality to future climate and land use change in the west of ireland | Yes | Yes | Yes | Yes | Yes | Yes | Yes | Yes | Yes | Yes | Yes | Yes | Yes | Yes | Yes | Yes |
| 544 | sewage and faecal sludge management; revisiting discharge standards in india | No | No | No | No | No | No | No | No | No | No | No | No | No | No | No | No |
| 545 | simulating fecal coliform bacteria loading from an urbanizing watershed | Yes | No | No | No | Yes | Yes | Yes | No | No | Yes | Yes | Yes | No | Yes | Yes | No |
| 546 | soil and water quality linked to landuse pattern -a case study of karuvannur river basin, thrissur district, kerala | No | No | No | Yes | No | Yes | Yes | No | No | No | No | No | No | No | No | No |
| 547 | sources and fate of salmonella and fecal indicator bacteria in an urban creek | Yes | Yes | Yes | Yes | Yes | Yes | No | Yes | Yes | Yes | Yes | Yes | Yes | Yes | Yes | Yes |
| 548 | sources and management of urban stormwater pollution in rural catchments, australia | Yes | No | No | Yes | Yes | Yes | No | No | Yes | No | No | Yes | No | Yes | Yes | Yes |
| 549 | sources and persistence of fecal coliform bacteria in a rural watershed | Yes | Yes | Yes | Yes | Yes | Yes | Yes | Yes | Yes | Yes | No | Yes | Yes | Yes | Yes | No |
| 550 | sources of nutrients and fecal indicator bacteria to nearshore waters on the north shore of kaua'i (hawai'i, usa) | Yes | Yes | Yes | Yes | Yes | Yes | Yes | Yes | Yes | Yes | Yes | Yes | Yes | Yes | Yes | Yes |
| 551 | spatial and hydrologic variation of bacteroidales, adenovirus and enterovirus in a semi-arid, wastewater effluent-impacted watershed | No | No | No | No | No | No | No | No | No | No | No | No | No | No | No | No |
| 552 | spatial and seasonal variability of the water quality characteristics of a river in northeast brazil | No | No | No | No | No | No | No | No | No | No | No | Yes | No | No | No | No |
| 553 | spatial and temporal analysis of land cover change, sedimentation and water quality in the lake issaqueena watershed, south carolina | Yes | Yes | Yes | Yes | Yes | Yes | Yes | Yes | Yes | Yes | Yes | Yes | Yes | Yes | Yes | Yes |
| 554 | spatial and temporal bacterial quality of a lowland agricultural stream in northeast scotland | Yes | Yes | Yes | Yes | Yes | Yes | Yes | Yes | Yes | Yes | Yes | Yes | Yes | Yes | Yes | Yes |
| 555 | spatial and temporal characterization of escherichia coli, suspended particulate matter and land use practice relationships in a mixed-land use contemporary watershed | Yes | Yes | Yes | Yes | Yes | Yes | Yes | Yes | Yes | Yes | Yes | Yes | Yes | Yes | Yes | Yes |
| 556 | spatial and temporal distribution of cryptosporidium and giardia in a drinking water resource: implications for monitoring and risk assessment | No | No | No | No | No | No | No | No | No | No | No | No | No | No | No | No |
| 557 | spatial and temporal drivers of zoonotic pathogen contamination of an agricultural watershed | No | No | No | No | No | No | No | No | No | No | No | No | No | No | No | No |
| 558 | spatial and temporal dynamics of suspended particles and e. coli in a complex surface-water and karst groundwater system as a basis for an adapted water protection scheme, northern vietnam | Yes | Yes | Yes | Yes | Yes | Yes | Yes | Yes | Yes | Yes | Yes | Yes | Yes | Yes | Yes | Yes |
| 559 | spatial and temporal evaluation of water streams using quality indexes: a case study | No | No | No | No | No | No | No | No | No | No | No | No | No | No | No | No |
| 560 | spatial and temporal variability of surface water pollution in the mekong delta, vietnam | No | No | No | No | No | No | No | No | No | No | No | No | No | No | No | No |
| 561 | spatial and temporal variation of fecal indicator organisms in two creeks in beltsville, maryland | Yes | Yes | Yes | Yes | Yes | Yes | Yes | Yes | Yes | Yes | Yes | Yes | Yes | Yes | Yes | Yes |
| 562 | spatial and temporal variations in microbiological water quality of the river wiwi in kumasi, ghana | Yes | Yes | Yes | No | Yes | Yes | Yes | Yes | Yes | Yes | Yes | Yes | No | No | No | No |
| 563 | spatial and temporal variations in pollution indicator bacteria in the lower vaal river, south africa | Yes | Yes | Yes | Yes | No | No | No | Yes | Yes | Yes | No | No | No | Yes | No | Yes |
| 564 | spatial aspects of surface water quality in the jakara basin, nigeria using chemometric analysis | No | No | No | No | No | No | No | No | No | No | No | No | No | No | No | No |
| 565 | spatial assessment and analysis of pollution sources and water quality in the bogue falaya river and abita river watersheds, st. tammany parish, la | Yes | Yes | Yes | Yes | Yes | Yes | Yes | Yes | Yes | Yes | Yes | Yes | Yes | Yes | Yes | Yes |
| 566 | spatial assessment of water quality in the vicinity of lake alice national wildlife refuge, upper devils lake basin, north dakota | Yes | Yes | Yes | Yes | Yes | Yes | Yes | Yes | Yes | Yes | Yes | Yes | Yes | Yes | Yes | Yes |
| 567 | spatial characterization of pollution sources: an analysis of in-stream water quality data from the potomac headwaters of west virginia | Yes | Yes | Yes | Yes | Yes | Yes | Yes | Yes | Yes | Yes | Yes | Yes | Yes | Yes | Yes | Yes |
| 568 | spatial distribution of coliform bacteria in batang arau river, padang, west sumatera, indonesia | Yes | Yes | Yes | Yes | No | Yes | No | Yes | Yes | Yes | Yes | Yes | Yes | No | Yes | No |
| 569 | spatial pattern assessment of lake kivu basin rivers water quality using national sanitation foundation water quality and rivers pollution indices | Yes | Yes | Yes | Yes | Yes | Yes | Yes | Yes | Yes | Yes | Yes | Yes | Yes | Yes | Yes | Yes |
| 570 | spatial patterns of enzymatic activity in large water bodies: ship-borne measurements of beta-d-glucuronidase activity as a rapid indicator of microbial water quality | Yes | Yes | Yes | Yes | Yes | Yes | Yes | Yes | Yes | Yes | Yes | Yes | Yes | Yes | Yes | Yes |
| 571 | spatial patterns of water quality in the cuiaba river basin, central brazil | No | No | No | No | No | No | No | No | Yes | Yes | No | No | No | No | No | No |
| 572 | spatial scale of land-use impacts on riverine drinking source water quality | Yes | Yes | Yes | Yes | Yes | Yes | No | Yes | Yes | Yes | Yes | Yes | Yes | Yes | Yes | Yes |
| 573 | spatial variation of physico-chemical and hydrological parameters with land-use in venkatapura catchment, karnataka | No | No | Yes | No | No | Yes | Yes | Yes | No | Yes | Yes | No | No | No | No | No |
| 574 | spatial-temporal assessment of pollutions in ekbatan lake using qualitative indices and statistical methods | No | No | No | No | No | No | No | No | No | Yes | No | No | No | No | No | No |
| 575 | spatially explicit pollutant load integrated in stream e. coli concentration modeling in a mixed land use catchment | Yes | Yes | Yes | Yes | Yes | Yes | Yes | Yes | Yes | Yes | Yes | Yes | Yes | Yes | Yes | Yes |
| 576 | spatio-temporal analysis of urban changes and surface water quality | Yes | Yes | Yes | Yes | Yes | Yes | Yes | Yes | Yes | Yes | Yes | Yes | Yes | Yes | Yes | Yes |
| 577 | spatio-temporal distribution of fecal indicators in three rivers of the haihe river basin, china | Yes | Yes | Yes | Yes | Yes | Yes | Yes | Yes | Yes | Yes | Yes | Yes | Yes | Yes | Yes | Yes |
| 578 | spatio-temporal variation of water quality in the yan oya river basin, sri lanka | Yes | Yes | Yes | Yes | Yes | Yes | No | Yes | Yes | Yes | Yes | Yes | Yes | Yes | Yes | Yes |
| 579 | spatiotemporal analysis of cryptosporidium species/genotypes and relationships with other zoonotic pathogens in surface water from mixed-use watersheds | Yes | Yes | Yes | Yes | Yes | Yes | Yes | No | Yes | Yes | No | Yes | Yes | Yes | No | Yes |
| 580 | spatiotemporal characteristics of the water quality in the jinsha river basin (panzhihua, china) | Yes | Yes | Yes | Yes | Yes | Yes | Yes | Yes | Yes | Yes | Yes | Yes | Yes | Yes | Yes | Yes |
| 581 | spatiotemporal characterization of water chemistry and pollution sources of the umhlatuzana, umbilo and amanzimnyama river catchments of durban, kwazulu-natal, south africa | Yes | Yes | Yes | Yes | Yes | Yes | Yes | Yes | Yes | Yes | Yes | Yes | Yes | Yes | Yes | Yes |
| 582 | spatiotemporal variability and key influencing factors of river fecal coliform within a typical complex watershed | Yes | Yes | Yes | Yes | Yes | Yes | Yes | Yes | Yes | Yes | Yes | Yes | Yes | Yes | Yes | Yes |
| 583 | spatiotemporal variation and the role of wildlife in seasonal water quality declines in the chobe river, botswana | Yes | Yes | Yes | Yes | Yes | Yes | Yes | Yes | Yes | Yes | Yes | Yes | Yes | Yes | No | Yes |
| 584 | spatiotemporal variation of bacterial water quality and the relationship with pasture land cover | Yes | Yes | Yes | Yes | Yes | Yes | Yes | Yes | Yes | Yes | Yes | Yes | Yes | Yes | Yes | Yes |
| 585 | state and potential management to improve water quality in an agricultural catchment relative to a natural baseline | Yes | Yes | Yes | Yes | Yes | Yes | Yes | Yes | Yes | Yes | Yes | Yes | Yes | Yes | Yes | Yes |
| 586 | statewide empirical modeling of bacterial contamination of surface waters | Yes | Yes | Yes | Yes | Yes | Yes | Yes | Yes | Yes | Yes | Yes | Yes | Yes | Yes | Yes | Yes |
| 587 | statistical assessment and neural network modeling of stream water quality observations of green river watershed, ky, usa | No | No | No | No | No | No | No | No | No | No | No | No | No | No | No | No |
| 588 | statistical assessment of nonpoint source pollution in agricultural watersheds in the lower grand river watershed, mo, usa | Yes | Yes | Yes | Yes | Yes | Yes | No | Yes | Yes | Yes | Yes | Yes | Yes | No | Yes | Yes |
| 589 | statistical investigations into indicator bacteria concentrations in houston metropolitan watersheds | Yes | Yes | Yes | Yes | Yes | Yes | Yes | Yes | Yes | Yes | Yes | Yes | Yes | Yes | Yes | Yes |
| 590 | status and trends of fecal indicator bacteria in two urban watersheds | Yes | Yes | Yes | Yes | Yes | Yes | Yes | Yes | Yes | Yes | Yes | Yes | Yes | No | Yes | Yes |
| 591 | storm water events in a small agricultural watershed: characterization and evaluation of improvements in stream water microbiology following implementation of best management practices | Yes | Yes | Yes | Yes | Yes | Yes | Yes | Yes | Yes | Yes | Yes | Yes | Yes | Yes | Yes | Yes |
| 592 | stormflow dynamics and loads of escherichia coli in a large mixed land use catchment | Yes | Yes | Yes | Yes | Yes | Yes | Yes | Yes | Yes | Yes | Yes | Yes | Yes | Yes | Yes | Yes |
| 593 | straight pipes and household wastewater discharges into the rural alabama and impact on watershed water quality with wetland land-uses | Yes | Yes | Yes | Yes | Yes | Yes | Yes | Yes | Yes | Yes | Yes | Yes | Yes | Yes | Yes | Yes |
| 594 | stream water quality changes following timber harvest in a coastal plain swamp forest | Yes | Yes | Yes | Yes | Yes | Yes | Yes | Yes | Yes | Yes | Yes | Yes | Yes | Yes | Yes | Yes |
| 595 | studies show buff ers can reduce bacteria and protect waterways | No | No | No | No | No | No | No | No | No | Yes | No | No | No | No | No | Yes |
| 596 | study of spatial distribution of water quality and landscape types impact on stream water quality in butler county, oh | Yes | Yes | Yes | Yes | Yes | Yes | Yes | Yes | Yes | Yes | Yes | Yes | Yes | Yes | Yes | Yes |
| 597 | study of the impact of land use and hydrogeological settings on the shallow groundwater quality in a peri-urban area of kampala, uganda | Yes | Yes | Yes | Yes | Yes | Yes | Yes | Yes | Yes | Yes | Yes | Yes | Yes | Yes | Yes | Yes |
| 598 | study of water quality in bengalon river on oil palm estate | No | No | No | No | No | Yes | No | No | No | No | No | No | No | Yes | No | Yes |
| 599 | study on water quality of mahabad dam river and lake for drinking and tourism purposes | No | No | No | No | No | No | No | No | No | No | No | No | No | No | No | No |
| 600 | surface water and contamination sources in urban river watersheds (northern portugal) | No | No | No | No | No | No | No | No | No | No | No | No | No | No | No | No |
| 601 | surface water contamination risk assessment modeled by fuzzy-wrastic | No | No | No | No | No | No | No | No | No | No | No | No | No | No | No | No |
| 602 | surface water quality after the woolsey fire in southern california | No | No | Yes | No | No | No | No | No | Yes | No | No | Yes | Yes | No | Yes | Yes |
| 603 | surface water quality along the central john muir trail in the sierra nevada mountains: coliforms and algae | Yes | Yes | Yes | Yes | Yes | Yes | Yes | Yes | Yes | Yes | Yes | Yes | Yes | Yes | Yes | Yes |
| 604 | surface water quality and landscape gradients in the north carolina cape fear river basin: the key role of fecal coliform | Yes | Yes | Yes | Yes | Yes | Yes | Yes | Yes | Yes | Yes | Yes | Yes | Yes | Yes | Yes | Yes |
| 605 | surface water quality in rural communities in the state of goi?s during the dry season and its relationship with land use and occupation | Yes | Yes | Yes | Yes | Yes | Yes | Yes | Yes | Yes | Yes | Yes | Yes | Yes | Yes | Yes | Yes |
| 606 | surface water quality in rural communities in the state of goias during the dry season and its relationship with land use and occupation | Yes | Yes | Yes | Yes | Yes | Yes | Yes | Yes | Yes | Yes | Yes | Yes | Yes | Yes | Yes | Yes |
| 608 | temporal and spatial monitoring of escherichia coli and pathogen indicators in the shallow groundwater and vadose zone, abbotsford, british columbia, canada | Yes | No | Yes | Yes | Yes | Yes | Yes | Yes | Yes | Yes | Yes | Yes | Yes | Yes | Yes | Yes |
| 613 | the effect of anthropogenic pressure shown by microbiological and chemical water quality indicators on the main rivers of podhale, southern poland | Yes | Yes | Yes | Yes | Yes | Yes | Yes | Yes | Yes | Yes | Yes | Yes | Yes | Yes | Yes | Yes |
| 614 | the effects of precipitation, river discharge, land use and coastal circulation on water quality in coastal maine | No | No | No | No | No | No | No | No | No | No | No | No | No | No | No | No |
| 615 | the effects of spatial variability of land use on stream water quality in a costal watershed | Yes | Yes | Yes | No | Yes | No | No | Yes | Yes | No | Yes | Yes | Yes | Yes | Yes | Yes |
| 616 | the effects on lowland habitat in the banks island bird sanctuary number 1, northwest territories, by the growing colony of lesser snow geese (chen caerulescens caerulescens) | No | No | No | No | No | No | No | No | No | No | No | No | No | No | No | No |
| 617 | the hydrology and geochemistry of urban and rural watersheds in east-central missouri | No | Yes | No | No | No | No | No | No | No | No | No | Yes | No | Yes | No | No |
| 618 | the impact of cattle farming best management practices on surface water nutrient concentrations, faecal bacteria and algal dominance in the lake oconee watershed | Yes | Yes | No | Yes | Yes | Yes | No | Yes | Yes | Yes | Yes | Yes | Yes | Yes | Yes | Yes |
| 619 | the impact of various land uses on the microbial and physicochemical quality of surface water bodies in developing countries: prioritisation of water resources management areas | Yes | Yes | Yes | Yes | Yes | Yes | Yes | Yes | Yes | Yes | Yes | Yes | Yes | Yes | Yes | Yes |
| 620 | the inca-pathogens model: an application to the loimijoki river basin in finland | Yes | Yes | Yes | Yes | Yes | Yes | Yes | Yes | Yes | Yes | Yes | Yes | Yes | Yes | Yes | Yes |
| 621 | the influence of land-use composition on fecal contamination of riverine source water in southern british columbia | Yes | Yes | Yes | Yes | Yes | Yes | Yes | Yes | Yes | Yes | Yes | Yes | Yes | Yes | Yes | Yes |
| 622 | the influence of rainfall on the incidence of microbial faecal indicators and the dominant sources of faecal pollution in a florida river | Yes | Yes | No | Yes | No | No | No | Yes | No | Yes | Yes | Yes | Yes | Yes | Yes | Yes |
| 623 | the interactions of indicator bacteria and sediments in fresh water streams | Yes | Yes | Yes | Yes | Yes | Yes | Yes | Yes | Yes | Yes | Yes | Yes | Yes | Yes | Yes | Yes |
| 624 | the modelling approach for predicting coastal pollutions using rainfall distributions over different land use/land cover | No | No | Yes | No | No | No | Yes | Yes | Yes | Yes | No | No | No | No | No | No |
| 625 | the modified swat model for predicting fecal coliforms in the wachusett reservoir watershed, usa | No | No | No | No | No | No | No | No | No | No | No | No | No | No | No | No |
| 626 | the multiscale tropical catchments critical zone observatory m-tropics dataset ii: land use, hydrology and sediment production monitoring in houay pano, northern lao pdr | No | No | No | No | No | No | No | No | No | No | No | No | No | No | No | No |
| 627 | the need for proper management leading to the sustainability of the kelani river and its lower basin | Yes | No | Yes | Yes | Yes | No | No | Yes | Yes | Yes | No | Yes | Yes | Yes | No | Yes |
| 628 | the relationship of land use practices to surface water quality in the upper oconee watershed of georgia | Yes | Yes | Yes | Yes | Yes | Yes | Yes | Yes | Yes | Yes | Yes | Yes | Yes | Yes | Yes | Yes |
| 629 | the role of land use and environmental factors on microbial pollution of mountainous limestone aquifers | Yes | Yes | Yes | Yes | Yes | Yes | Yes | Yes | Yes | Yes | Yes | No | Yes | Yes | Yes | Yes |
| 630 | the role of riparian buffer management in reducing off-site impacts from grazed dairy systems | No | No | No | No | No | No | No | No | No | No | No | No | No | No | No | No |
| 631 | the settling of resource water quality objectives for the modder-riet river catchment | No | No | No | No | No | Yes | No | No | No | No | Yes | No | No | No | No | No |
| 632 | the use of selected water quality parameters to identify fecal coliform sources in support of the sinking creek total maximum daily load | Yes | Yes | Yes | Yes | Yes | Yes | Yes | Yes | Yes | Yes | Yes | Yes | Yes | Yes | Yes | Yes |
| 633 | tracing stream nitrate in a central pennsylvania mixed land-use basin using stable isotopes, bacteria, and inorganic chemicals | No | No | Yes | Yes | No | Yes | Yes | No | No | No | No | No | No | No | No | No |
| 634 | tracing water perturbation using no(3)(-), doc, particles size determination, and bacteria: a method development for karst aquifer water quality hazard assessment | No | No | No | No | Yes | No | Yes | No | No | No | Yes | No | Yes | No | No | Yes |
| 635 | tracking host sources of cryptosporidium spp. in raw water for improved health risk assessment | No | No | No | No | No | No | No | No | No | No | No | No | No | No | No | No |
| 636 | tracking sources and dissemination of indicator antibiotic resistance genes at a watershed scale | Yes | Yes | Yes | Yes | Yes | Yes | Yes | Yes | Yes | Yes | Yes | Yes | Yes | Yes | Yes | Yes |
| 637 | tracking sources of bacterial contamination in stormwater discharges to mission bay, california | No | No | No | No | No | Yes | Yes | No | Yes | Yes | No | Yes | Yes | No | No | Yes |
| 638 | tracking the sources of allochthonous organic matter along a subtropical fluvial-estuarine gradient using molecular proxies in view of land uses | No | No | No | No | No | No | No | No | No | No | No | No | No | No | No | No |
| 639 | transport and variability of fecal bacteria in carbonate conglomerate aquifers | Yes | No | No | Yes | Yes | Yes | Yes | Yes | Yes | No | No | No | Yes | No | Yes | Yes |
| 640 | trend analysis of water quality in some rivers with different degress of development within the sao paulo state, brazil | Yes | Yes | No | No | No | No | Yes | Yes | Yes | No | Yes | Yes | No | Yes | Yes | Yes |
| 641 | trends in water quality of five dairy farming streams in response to adoption of best practice and benefits of long-term monitoring at the catchment scale | Yes | Yes | Yes | Yes | Yes | Yes | Yes | Yes | Yes | Yes | Yes | Yes | Yes | Yes | Yes | Yes |
| 643 | twenty years of land use and the impact of nitrate, e. coli and chlorophyll for two lakes in north central texas | No | No | No | No | No | No | No | No | No | No | No | No | No | No | No | No |
| 644 | understanding the effects of cattle grazing in english chalk streams | No | No | No | No | No | No | No | No | No | No | No | No | No | No | No | No |
| 645 | understanding the impact of land use on microbial water quality to support decisions for a future land use plan | Yes | Yes | Yes | Yes | Yes | Yes | Yes | Yes | Yes | Yes | Yes | Yes | Yes | Yes | Yes | Yes |
| 649 | urban drool water quality in denver, colorado: pollutant occurrences and sources in dry-weather flows | No | Yes | No | Yes | No | No | Yes | No | No | Yes | No | No | No | No | Yes | No |
| 651 | urban influences on stream chemistry and biology in the big brushy creek watershed, south carolina | Yes | Yes | Yes | Yes | Yes | No | Yes | Yes | Yes | Yes | Yes | Yes | Yes | No | Yes | No |
| 652 | urban landscapes increase dispersal, gene flow, and pathogen transmission potential in banded mongoose (mungos mungo) in northern botswana | No | No | No | No | No | No | No | No | No | No | No | No | No | No | No | No |
| 653 | urban pollution of bagmati river corridor within the densely populated kathmandu valley in nepal | Yes | Yes | Yes | Yes | Yes | Yes | Yes | Yes | Yes | Yes | Yes | Yes | Yes | Yes | Yes | Yes |
| 654 | use of fallout radionuclides ((7)be, (210)pb) to estimate resuspension of escherichia coli from streambed sediments during floods in a tropical montane catchment | No | No | No | No | No | No | No | No | No | No | No | No | No | No | No | No |
| 655 | using a weight-of-evidence approach for management of watersheds | Yes | Yes | Yes | Yes | Yes | Yes | Yes | Yes | Yes | Yes | Yes | Yes | Yes | Yes | Yes | Yes |
| 656 | using generalized additive mixed models to assess spatial, temporal, and hydrologic controls on bacteria and nitrate in a vulnerable agricultural aquifer | No | No | No | No | No | No | No | No | No | No | No | No | Yes | Yes | Yes | No |
| 657 | using microbial source tracking to identify contamination sources in port jefferson harbor, setauket harbor, and conscience bay on long island, new york | No | Yes | No | No | No | No | Yes | No | Yes | No | Yes | No | No | No | Yes | Yes |
| 658 | using microbiological tracers to assess the impact of winter land use restrictions on the quality of stream headwaters in a small catchment | Yes | Yes | Yes | Yes | Yes | Yes | Yes | Yes | Yes | Yes | Yes | Yes | Yes | Yes | Yes | Yes |
| 659 | using multi-threshold regression techniques to assess river fecal pollution in the highly urbanized tamsui river watershed | Yes | Yes | Yes | Yes | Yes | Yes | Yes | Yes | Yes | Yes | Yes | Yes | Yes | Yes | Yes | Yes |
| 660 | using radical terraces for erosion control and water quality improvement in rwanda: a case study in sebeya catchment | Yes | Yes | Yes | Yes | Yes | Yes | Yes | Yes | Yes | Yes | Yes | Yes | Yes | Yes | Yes | Yes |
| 661 | using remote sensing to identify changes in land use and sources of fecal bacteria to support a watershed transport model | Yes | Yes | Yes | Yes | Yes | Yes | Yes | Yes | Yes | Yes | Yes | Yes | Yes | Yes | Yes | Yes |
| 662 | using spatial-stream-network models and long-term data to understand and predict dynamics of faecal contamination in a mixed land-use catchment | Yes | Yes | Yes | Yes | Yes | Yes | Yes | Yes | Yes | Yes | Yes | Yes | Yes | Yes | Yes | Yes |
| 663 | using watershed characteristics to enhance fecal source identification | Yes | Yes | Yes | Yes | Yes | Yes | Yes | Yes | Yes | Yes | Yes | Yes | Yes | Yes | Yes | Yes |
| 669 | verifying the applicability of swat to simulate fecal contamination for watershed management of selangor river, malaysia | Yes | Yes | Yes | No | Yes | Yes | Yes | No | No | No | Yes | No | Yes | Yes | Yes | No |
| 670 | vertical flow constructed wetlands as green facades and gardens for on-site greywater treatment in buildings: two-year mesocosm study on removal performance | Yes | No | No | No | Yes | Yes | No | Yes | Yes | Yes | Yes | Yes | No | No | Yes | No |
| 676 | water quality and the effects of different pastoral animals | No | No | No | No | No | No | No | No | No | No | No | No | No | No | No | No |
| 677 | water quality and thermal regime of the motueka river: influences of land cover, geology and position in the catchment | Yes | Yes | Yes | Yes | Yes | Yes | Yes | Yes | No | Yes | Yes | Yes | Yes | Yes | Yes | Yes |
| 678 | water quality assessment and evaluation of human health risk in mutangwi river, limpopo province, south africa | No | No | No | No | No | No | No | No | No | No | No | No | No | No | No | No |
| 679 | water quality assessment and meta model development in melen watershed - turkey | No | No | No | No | No | No | No | No | No | No | No | No | No | No | No | No |
| 680 | water quality assessment and the influence of landscape metrics at multiple scales in poyang lake basin | Yes | Yes | Yes | Yes | Yes | Yes | Yes | Yes | Yes | Yes | Yes | Yes | Yes | Yes | Yes | Yes |
| 681 | water quality assessment of six rivers of the pacific side of guatemala | No | No | No | No | No | No | Yes | Yes | Yes | No | No | No | No | No | Yes | No |
| 682 | water quality assessment with emphasis in parameter optimisation using pattern recognition methods and genetic algorithm | Yes | Yes | Yes | Yes | Yes | Yes | Yes | Yes | Yes | Yes | Yes | Yes | Yes | Yes | Yes | Yes |
| 683 | water quality changes in a polluted stream over a twenty-five-year period | Yes | Yes | Yes | Yes | No | Yes | No | Yes | No | No | No | Yes | Yes | Yes | Yes | No |
| 684 | water quality evaluation of two urban streams in northwest uruguay: are national regulations for urban stream quality sufficient? | Yes | No | Yes | Yes | No | Yes | No | Yes | Yes | Yes | No | No | No | Yes | Yes | Yes |
| 685 | water quality in aguadas within a protected karstic rain forest: the role of the vegetation-soil-water interactions | No | No | No | No | Yes | Yes | No | No | Yes | No | No | No | Yes | Yes | No | Yes |
| 686 | water quality in an urban environmental protection area in the cerrado biome, brazil | No | No | No | No | Yes | No | No | No | No | No | No | No | No | Yes | No | No |
| 687 | water quality in low-elevation streams and rivers of new zealand: recent state and trends in contrasting land-cover classes | Yes | Yes | Yes | Yes | Yes | Yes | Yes | Yes | Yes | Yes | Yes | Yes | Yes | Yes | Yes | Yes |
| 688 | water quality in microbasins and springs of the mineral water region of minas gerais state, brazil | Yes | No | Yes | Yes | Yes | Yes | Yes | Yes | No | Yes | No | No | Yes | Yes | No | Yes |
| 689 | water quality in new zealand rivers: current state and trends | Yes | Yes | Yes | Yes | Yes | Yes | Yes | Yes | Yes | Yes | Yes | Yes | Yes | Yes | Yes | Yes |
| 690 | water quality in part of the submiddle san francisco region due to seasonality and land use and occupation | Yes | Yes | Yes | Yes | Yes | Yes | Yes | Yes | Yes | Yes | No | Yes | Yes | Yes | Yes | Yes |
| 691 | water quality in relation to watershed management in the lower san gabriel river, southern california | No | No | No | No | Yes | No | No | No | No | No | No | No | No | No | No | No |
| 692 | water quality in shallow alluvial aquifers, upper colorado river basin, colorado, 1997 | No | No | No | No | No | No | No | No | No | No | No | No | No | No | No | No |
| 693 | water quality in various land cover type in nanggala sub watershed | Yes | Yes | No | Yes | Yes | Yes | No | Yes | No | Yes | Yes | No | Yes | No | Yes | No |
| 694 | water quality in watershed of the jaboatao river (pernambuco, brazil): a case study | Yes | Yes | No | Yes | Yes | Yes | No | Yes | Yes | Yes | Yes | Yes | Yes | Yes | Yes | Yes |
| 695 | water quality index in an urban watershed | No | No | No | No | Yes | Yes | No | No | No | No | No | No | No | No | No | No |
| 696 | water quality index in two land use situations in the mantiqueira range | Yes | Yes | Yes | Yes | Yes | Yes | Yes | Yes | Yes | Yes | Yes | Yes | Yes | Yes | Yes | Yes |
| 697 | water quality index using modified random forest technique: assessing novel input features | No | No | No | No | No | No | No | No | No | No | No | No | No | No | No | No |
| 698 | water quality indicators in the mantiqueira range region, minas gerais state | Yes | Yes | Yes | Yes | Yes | Yes | Yes | Yes | Yes | Yes | Yes | Yes | Yes | Yes | Yes | Yes |
| 699 | water quality modification by land use types in watershed ecosystems of southwestern nigeria | Yes | Yes | Yes | Yes | Yes | Yes | Yes | No | Yes | No | No | Yes | No | Yes | No | Yes |
| 700 | water quality studies in kranji catchment, singapore: use of organic tracers and polyethylene devices for identifying potential sewage sources | No | No | No | No | No | No | No | No | No | No | No | No | No | No | No | No |
| 701 | water quality variation in tributaries of the three gorges reservoir from 2000 to 2015 | No | No | No | No | No | No | No | No | No | No | No | No | No | No | No | No |
| 702 | water transport, retention, and survival of escherichia coli in unsaturated porous media: a comprehensive review of processes, models, and factors | No | No | No | No | No | No | No | No | No | No | No | No | No | No | No | No |
| 703 | watershed land use, surface water vulnerability and public health risks of two urban rivers, ado-ekiti, south-west nigeria | Yes | Yes | Yes | Yes | Yes | Yes | Yes | Yes | Yes | Yes | Yes | Yes | Yes | Yes | Yes | Yes |
| 704 | watershed planning in central iowa: an integrated assessment of the squaw creek watershed for prioritization of conservation practice establishment | No | No | No | Yes | No | Yes | No | No | No | No | No | No | No | No | No | No |
| 705 | watershed water quality modeling using integrated fuzzy modeling approach with hspf model and radar rainfall data | No | No | No | No | No | No | No | No | No | No | No | No | No | No | No | No |
| 706 | what matters most? stakeholders' perceptions of river water quality | No | No | No | No | No | No | No | No | No | No | No | No | No | No | No | No |
| 707 | whole catchment land cover effects on water quality in the lower kaskaskia river watershed | Yes | Yes | Yes | Yes | Yes | Yes | Yes | Yes | Yes | Yes | Yes | Yes | Yes | Yes | Yes | Yes |
| 708 | widespread detection of human- and ruminant-origin bacteroidales markers in subtidal waters of the salish sea in washington state | No | No | No | No | No | No | No | No | No | No | No | No | No | No | No | No |
| 709 | wildlife identified as major source of escherichia coli in agriculturally dominated watersheds by box a1r-derived genetic fingerprints | Yes | Yes | Yes | Yes | Yes | Yes | Yes | Yes | Yes | Yes | Yes | Yes | Yes | Yes | Yes | Yes |
| 711 | fluctuating silicate:nitrate ratios and coastal plankton food webs | No | No | No | No | No | No | No | No | No | No | No | No | No | No | No | No |
