# Supplementary material for: AI-assisted evidence screening method for systematic reviews in environmental research: integrating ChatGPT with domain knowledge
Source: Environ Evid. 2025 Apr 15;14:5. doi: 10.1186/s13750-025-00358-5 (PMC11998256; doi:10.1186/s13750-025-00358-5)
Supplement: Supplementary file 7 — Supplementary Material 7 [file 13750_2025_358_MOESM7_ESM.docx]

**Table A8.** The reviewers’ results of 45 articles

| **Unique ID** | **Title** | **Consensus Decision** | **R1** | **R2** | **R3** | **Rounds** | **Dataset** |
| --- | --- | --- | --- | --- | --- | --- | --- |
| 3 | a 22-site comparison of land-use practices, e-coli and enterococci concentrations | Yes | Yes | Yes | Yes | 1 | Test set |
| 4 | a basis water quality monitoring plan for rehabilitation and protection | No | No | No | No | 3 | Test set |
| 10 | a geospatial analysis of land use and stormwater management on fecal coliform contamination in north carolina streams | Yes | Yes | Yes | No | 2 | Test set |
| 77 | bacteria modeling with swat for assessment and remediation studies: a review | No | No | No | No | 3 | Test set |
| 83 | bacterial pathogens in hawaiian coastal streams-associations with fecal indicators, land cover, and water quality | Yes | Yes | Yes | Yes | 2 | Test set |
| 99 | causal connections between water quality and land use in a rural tropical island watershed: rural tropical island watershed analysis | Yes | No | Yes | Yes | 2 | Test set |
| 100 | changes in chemical and physical propertiesof stream water across an urban-rural gradient in western georgia | Yes | Yes | Yes | Yes | 1 | Test set |
| 102 | changes in land use/management and water quality in the long creek watershed | No | No | No | No | 1 | Test set |
| 144 | decadal and seasonal water quality trends downstream of urban and rural areas in southern alberta rivers | No | No | No | No | 2 | Test set |
| 175 | dna fingerprinting using box-a1r and (gtg)(5) primers identify spatial variations of fecal contamination along pasig river, philippines | No | Yes | Yes | No | 1 | Test set |
| 180 | ecological water health assessment using benthic macroinvertebrate communities (case study: the ghezel ozan river in zanjan province, iran) | No | No | No | No | 2 | Test set |
| 240 | evaluation of the water quality status and pollution load carrying capacity of way umpu river, way kanan district, lampung province, indonesia, based on land use | No | No | No | No | 3 | Test set |
| 246 | factors and mechanisms affecting seasonal changes in the prevalence of microbiological indicators of water quality and nutrient concentrations in waters of the biaka river catchment, southern poland | No | No | No | No | 2 | Test set |
| 247 | factors associated with e. coli levels in and salmonella contamination of agricultural water differed between north and south florida waterways | Yes | No | Yes | No | 2 | Test set |
| 275 | fourier landscape pattern indices for predicting south carolina watershed fecal coliform | No | Yes | No | Yes | 1 | Test set |
| 277 | generic modelling of faecal indicator organism concentrations in the uk | Yes | Yes | Yes | Yes | 1 | Test set |
| 278 | genetic fecal source identification in urban streams impacted by municipal separate storm sewer system discharges | Yes | No | No | Yes | 2 | Test set |
| 289 | groupwise modeling study of bacterially impaired watersheds in texas: clustering analysis | No | Yes | No | Yes | 2 | Test set |
| 290 | growing season surface water loading of fecal indicator organisms within a rural watershed | No | No | No | No | 1 | Test set |
| 317 | identifying sources of fecal pollution in the colville river using library-independent genetic markers | No | No | No | No | 2 | Test set |
| 318 | impact of changes of land use on water quality, from tropical forest to anthropogenic occupation: a multivariate approach | Yes | Yes | No | Yes | 1 | Test set |
| 380 | land cover impacts on stream nutrients and fecal coliform in the lower piedmont of west georgia | Yes | Yes | Yes | Yes | 3 | Test set |
| 381 | land use and environmental variables influence tetracycline-resistant bacteria occurrence in southeastern coastal plain streams | Yes | Yes | Yes | Yes | 3 | Test set |
| 382 | land use and hydroclimatic influences on faecal indicator organisms in two large scottish catchments: towards land use-based models as screening tools | Yes | No | Yes | Yes | 2 | Test set |
| 414 | macroinvertebrate indices versus microbial fecal pollution characteristics for water quality monitoring reveals contrasting results for an ethiopian river | No | No | No | Yes | 2 | Test set |
| 456 | multiscale spatiotemporal variability of fecal indicator bacteria and associated particle size distributions in the sandy bottom sediments of a pennsylvania creek | No | No | No | No | 3 | Test set |
| 457 | native forest cover safeguards stream water quality under a changing climate | Yes | Yes | Yes | Yes | 1 | Test set |
| 462 | occurrence and distribution of microbiological indicators in groundwater and stream water | Yes | Yes | Yes | Yes | 3 | Test set |
| 465 | occurrence of traditional and alternative fecal indicators in tropical urban environments under different land use patterns | Yes | Yes | Yes | Yes | 2 | Test set |
| 467 | participatory approach for more robust water resource management: case study of the santa rosa sub-watershed of the philippines | No | Yes | No | No | 3 | Test set |
| 502 | quantification of fecal coliform inputs to aquatic systems through soil leaching | Yes | Yes | No | Yes | 2 | Test set |
| 504 | quantification of microbial source tracking and pathogenic bacterial markers in water and sediments of tiaoxi river (taihu watershed) | No | No | No | No | 1 | Test set |
| 512 | rainfall driven e. coli transfer to the stream conduit network observed through increasing spatial scales in mixed land use paddy farming karst terrain | No | No | No | No | 3 | Test set |
| 513 | reach specificity in sediment e. coli population turnover and interaction with waterborne populations | No | No | No | No | 3 | Test set |
| 534 | sanitary analyses of runoff water a river | No | No | No | No | 3 | Test set |
| 609 | temporal stability of e. coli and enterococci concentrations in a pennsylvania creek | No | No | No | No | 3 | Test set |
| 610 | the 'black waters' of malaysia: tracking water quality from the peat swamp forest to the sea | No | No | No | No | 3 | Test set |
| 611 | the changing face of water: a dynamic reflection of antibiotic resistance across landscapes | No | No | No | No | 1 | Test set |
| 642 | turbidity as an indicator of water quality in diverse watersheds of the upper pecos river basin | No | No | No | No | 1 | Test set |
| 646 | understanding the spatiotemporal pollution dynamics of highly fragile montane watersheds of kashmir himalaya, india | No | No | Yes | Yes | 1 | Test set |
| 665 | validating microbial source tracking markers and assessing the efficacy of culturable e. coli and enterococcus assays in ozark streams, usa | Yes | Yes | Yes | Yes | 3 | Test set |
| 666 | variability of e. coli density and sources in an urban watershed | No | No | No | No | 1 | Test set |
| 673 | water and sediment microbial quality of mountain and agricultural streams | Yes | No | Yes | Yes | 1 | Test set |
| 674 | water pollution and water quality assessment of the way kuripan river in bandar lampung city (sumatera, indonesia) | No | No | No | No | 2 | Test set |
| 710 | year-long metagenomic study of river microbiomes across land use and water quality | No | No | Yes | Yes | 3 | Test set |
