# Supplementary material for: AI-assisted evidence screening method for systematic reviews in environmental research: integrating ChatGPT with domain knowledge
Source: Environ Evid. 2025 Apr 15;14:5. doi: 10.1186/s13750-025-00358-5 (PMC11998256; doi:10.1186/s13750-025-00358-5)
Supplement: Supplementary file 8 — Supplementary Material 8 [file 13750_2025_358_MOESM8_ESM.docx]

**Table A9.** The ChatGPT screening results of 339 articles in Step 2

| **Unique ID** | **Title** | **Consensus Decision** | **ChatGPT Majority Answer** | **1** | **2** | **3** | **4** | **5** | **6** | **7** | **8** | **9** | **10** | **11** | **12** | **13** | **14** | **15** |
| --- | --- | --- | --- | --- | --- | --- | --- | --- | --- | --- | --- | --- | --- | --- | --- | --- | --- | --- |
| 1 | !cuba! river water chemistry reveals rapid chemical weathering, the echo of uplift, and the promise of more sustainable agriculture | NA | No | No | No | No | No | No | No | No | No | No | No | No | No | No | No | No |
| 2 | escherichia coli concentration, multiscale monitoring over the decade 2011-2021 in the mekong river basin, lao pdr | NA | No | Yes | Yes | Yes | No | No | No | No | No | No | No | No | No | No | No | No |
| 3 | a 22-site comparison of land-use practices, e-coli and enterococci concentrations | Yes | Yes | Yes | Yes | Yes | Yes | Yes | Yes | Yes | Yes | Yes | Yes | Yes | Yes | Yes | Yes | Yes |
| 4 | a basis water quality monitoring plan for rehabilitation and protection | No | No | No | No | No | No | No | No | No | No | No | No | No | No | No | No | No |
| 7 | a comparison of soil staphylococcus aureus and fecal indicator bacteria concentrations across land uses in a hawaiian watershed | NA | No | No | No | No | No | No | No | No | No | No | No | No | No | No | No | No |
| 9 | a geographical approach to tracking escherichia coli and other water quality constituents in a texas coastal plains watershed | NA | No | No | No | No | No | No | No | No | No | No | No | No | No | No | No | No |
| 10 | a geospatial analysis of land use and stormwater management on fecal coliform contamination in north carolina streams | Yes | No | No | No | No | No | No | No | No | No | No | No | No | No | Yes | Yes | No |
| 11 | a hydrochemically guided landscape classification system for modelling spatial variation in multiple water quality indices: process-attribute mapping | NA | No | No | No | No | No | No | No | No | No | No | No | No | No | No | No | No |
| 13 | a multivariate and spatiotemporal analysis of water quality in code river, indonesia | NA | No | No | No | No | No | No | No | Yes | Yes | No | No | Yes | Yes | Yes | No | No |
| 14 | a multivariate statistical approach to the integration of different land-uses, seasons, and water quality as water resources management tool | NA | Yes | Yes | No | Yes | No | Yes | Yes | No | Yes | No | No | Yes | Yes | Yes | Yes | No |
| 15 | a novel approach for assessing watershed susceptibility using weighted overlay and analytical hierarchy process (ahp) methodology: a case study in eagle creek watershed, usa | NA | Yes | No | Yes | Yes | Yes | Yes | Yes | Yes | No | Yes | No | No | No | Yes | Yes | Yes |
| 17 | a review of on-farm roadway runoff characterisation and potential management options for ireland | NA | No | No | No | No | No | No | No | No | No | No | No | No | No | No | No | No |
| 20 | a spatial assessment of baseline nutrient and water quality values in the ashepoo-combahee-edisto (ace) basin, south carolina, usa | NA | No | No | No | No | No | No | No | No | No | No | No | No | No | No | No | No |
| 21 | a spatial-statistical approach for modeling the effect of non-point source pollution on different water quality parameters in the velhas river watershed--brazil | NA | Yes | Yes | Yes | Yes | Yes | Yes | Yes | Yes | Yes | Yes | Yes | Yes | Yes | Yes | Yes | Yes |
| 22 | a spatiotemporal analysis of water quality and land use in tambayakbayan river, yogyakarta | NA | Yes | Yes | Yes | Yes | No | Yes | Yes | Yes | Yes | Yes | Yes | No | Yes | No | No | Yes |
| 24 | a systematic assessment of watershed-scale nonpoint source pollution during rainfall-runoff events in the miyun reservoir watershed | NA | No | Yes | No | No | Yes | No | Yes | Yes | No | No | No | No | No | No | No | No |
| 25 | a watershed study assessing effects of commercial hog operations on microbial water quality in north carolina, usa | NA | No | No | No | No | No | No | No | No | No | No | No | No | No | No | No | No |
| 26 | abiotic and biotic changes at the basin scale in a tropical dry forest landscape after hurricanes jova and patricia in jalisco, mexico | NA | No | No | No | No | No | No | No | No | No | No | No | No | No | No | No | No |
| 28 | achieving sustainable water and land use systems in highly developed tropical landscapes | NA | No | No | No | No | No | No | No | No | No | No | No | No | No | No | No | No |
| 33 | advancing understanding of land use and physicochemical impacts on fecal contamination in mixed-land-use watersheds | NA | Yes | Yes | Yes | No | No | Yes | Yes | No | No | No | Yes | Yes | Yes | Yes | Yes | Yes |
| 40 | an inca model for pathogens in rivers and catchments: model structure, sensitivity analysis and application to the river thames catchment, uk | NA | No | No | No | No | No | No | No | No | No | No | No | No | No | No | No | No |
| 42 | analysis of escherichia coli and enterococci concentrations patterns in a pennsylvania creek using empirical orthogonal functions | NA | Yes | Yes | Yes | Yes | Yes | Yes | Yes | Yes | Yes | Yes | Yes | Yes | Yes | Yes | Yes | Yes |
| 43 | analysis of the hspf water quality parameter uncertainty in predicting peak in-stream fecal coliform concentrations | NA | No | No | No | No | No | No | No | No | No | No | No | No | No | No | No | No |
| 44 | analytical and detection sources of pollution based environmetric techniques in malacca river, malaysia | NA | No | No | No | No | No | No | No | No | No | No | No | No | No | No | No | No |
| 45 | anthropogenic influence on surface water quality of the nhue and day sub-river systems in vietnam | NA | No | No | No | No | No | No | No | No | No | No | No | No | No | No | No | No |
| 48 | application of host-specific genetic markers for microbial source tracking of faecal water contamination in an agricultural catchment | NA | No | No | No | No | No | No | No | No | No | No | No | No | No | No | No | No |
| 49 | application of indexes to assess the water quality of coastal basin of the sapucaia in sergipe | NA | No | No | No | No | No | No | No | No | No | No | No | No | No | No | No | No |
| 54 | applying the manning equation to determine the critical distance in non-point source pollution using remotely sensed data and cartographic modelling | NA | Yes | Yes | Yes | No | No | Yes | No | Yes | Yes | No | Yes | Yes | Yes | Yes | Yes | Yes |
| 55 | assessing environmental contamination of river ganga using correlation and multivariate analysis | NA | No | No | No | No | No | No | No | No | No | No | No | No | No | No | No | No |
| 56 | assessing land-cover effects on stream water quality in metropolitan areas using the water quality index | NA | Yes | Yes | Yes | Yes | Yes | Yes | Yes | Yes | Yes | No | Yes | Yes | Yes | Yes | Yes | Yes |
| 60 | assessing the impacts of watershed indexes and precipitation on spatial in-stream e. coli concentrations | NA | Yes | Yes | No | Yes | No | Yes | Yes | Yes | Yes | No | Yes | Yes | Yes | No | Yes | Yes |
| 61 | assessing the service of water quality regulation by quantifying the effects of land use on water quality and public health in central veracruz, mexico | NA | No | No | No | No | No | No | No | No | No | No | No | No | No | No | No | No |
| 62 | assessing the yield and load of contaminants with stream order: would policy requiring livestock to be fenced out of high-order streams decrease catchment contaminant loads? | NA | No | No | No | No | No | No | No | No | No | No | No | No | No | No | No | No |
| 64 | assessing water use and quality through youth participatory research in a rural andean watershed | NA | No | No | No | No | No | No | No | No | No | No | No | No | No | No | No | No |
| 65 | assessment of characteristics, water quality and groundwater vulnerability in pakis district, east java province, indonesia | NA | No | No | No | No | No | No | No | No | No | No | No | No | No | No | No | No |
| 66 | assessment of emerging hydrological, water quality issues and policy discussion on water sharing of transboundary kabul river | NA | No | No | No | No | No | No | No | No | No | No | No | No | No | No | No | No |
| 67 | assessment of fecal coliform and escherichia coli across a land cover gradient in west georgia streams | NA | Yes | Yes | Yes | Yes | Yes | Yes | Yes | Yes | Yes | Yes | Yes | Yes | Yes | Yes | Yes | Yes |
| 68 | assessment of physico-chemical and microbiological parameters of mthatha river in eastern cape, south africa | NA | No | No | No | No | No | No | No | No | No | No | No | No | No | No | No | No |
| 70 | assessment of water quality and identification of pollution risk locations in tiaoxi river (taihu watershed), china | NA | No | No | No | No | No | No | No | No | No | No | No | No | No | No | No | No |
| 76 | associations among pathogenic bacteria, parasites, and environmental and land use factors in multiple mixed-use watersheds | NA | No | No | No | No | No | No | No | No | No | No | No | No | No | No | No | No |
| 77 | bacteria modeling with swat for assessment and remediation studies: a review | No | No | No | No | No | No | No | No | No | No | No | No | No | No | No | No | No |
| 81 | bacterial indicators of faecal pollution in the waters of the el-kabir river and akkar watershed in syria and lebanon | NA | No | No | No | No | No | No | No | No | No | No | No | No | No | No | No | No |
| 83 | bacterial pathogens in hawaiian coastal streams-associations with fecal indicators, land cover, and water quality | Yes | No | No | No | No | No | No | No | No | No | No | No | Yes | Yes | Yes | No | Yes |
| 97 | bslc: a tool for bacteria source characterization for watershed management | NA | No | No | No | No | No | No | No | No | No | No | No | No | No | No | No | No |
| 99 | causal connections between water quality and land use in a rural tropical island watershed: rural tropical island watershed analysis | Yes | No | No | No | No | No | No | No | No | No | No | No | No | No | No | No | No |
| 100 | changes in chemical and physical propertiesof stream water across an urban-rural gradient in western georgia | Yes | Yes | Yes | Yes | Yes | Yes | Yes | Yes | Yes | Yes | Yes | Yes | Yes | Yes | Yes | No | Yes |
| 102 | changes in land use/management and water quality in the long creek watershed | No | No | No | No | No | No | No | No | No | No | No | No | No | No | No | No | No |
| 105 | characterization and prediction of stormwater runoff quality in sub-tropical rural catchments | NA | No | No | No | No | No | No | No | No | No | No | No | No | No | No | No | No |
| 109 | characterization of sources and loadings of fecal pollutants using microbial source tracking assays in urban and rural areas of the grand river watershed, southwestern ontario | NA | No | No | No | No | No | No | No | Yes | No | No | No | No | No | No | No | No |
| 111 | characterizing differences in sources of and contributions to fecal contamination of sediment and surface water with the microbial fit framework | NA | No | No | No | No | No | No | No | No | No | No | No | No | No | No | No | No |
| 112 | characterizing relationships among fecal indicator bacteria, microbial source tracking markers, and associated waterborne pathogen occurrence in stream water and sediments in a mixed land use watershed | NA | No | Yes | Yes | No | No | No | Yes | Yes | No | No | No | No | Yes | No | No | Yes |
| 113 | chemical and microbiological indicators to assess the impact of agricultural activities on groundwater in the pampean agro-ecosystem | NA | Yes | No | Yes | Yes | Yes | Yes | Yes | Yes | No | Yes | Yes | Yes | Yes | Yes | Yes | Yes |
| 114 | chronic urban hotspots and agricultural drainage drive microbial pollution of karst water resources in rural developing regions | NA | No | Yes | No | Yes | Yes | No | No | No | No | No | Yes | No | No | No | No | Yes |
| 116 | climate and land-use change impact on faecal indicator bacteria in a temperate maritime catchment (the river conwy, wales) | NA | No | No | No | No | No | No | No | No | No | No | No | No | No | No | No | No |
| 117 | climate change and land use drivers of fecal bacteria in tropical hawaiian rivers | NA | Yes | Yes | Yes | Yes | Yes | Yes | Yes | Yes | Yes | Yes | Yes | Yes | Yes | Yes | Yes | Yes |
| 122 | coherence among different microbial source tracking markers in a small agricultural stream with or without livestock exclusion practices | NA | No | No | No | No | No | No | No | No | No | No | No | No | No | No | No | No |
| 124 | combining land use information and small stream sampling with pcr-based methods for better characterization of diffuse sources of human fecal pollution | NA | Yes | Yes | No | No | No | Yes | Yes | Yes | Yes | Yes | Yes | Yes | Yes | Yes | No | Yes |
| 127 | comparison of qpcr and amplicon sequencing based methods for fecal source tracking in a mixed land use estuarine watershed | NA | No | No | No | No | No | No | No | No | No | No | No | No | No | No | No | No |
| 130 | compositions of first flush and composite storm water runoff in small urban and rural watersheds, north-central texas | NA | No | No | No | No | No | No | No | No | No | No | No | No | No | No | No | No |
| 133 | connecting microbial, nutrient, physiochemical, and land use variables for the evaluation of water quality within mixed use watersheds | NA | No | No | No | No | No | No | No | Yes | No | No | No | No | No | Yes | No | No |
| 136 | contamination with bacterial zoonotic pathogen genes in u.s. streams influenced by varying types of animal agriculture | NA | No | No | No | No | No | No | No | No | No | No | No | No | No | No | No | No |
| 139 | coupled dynamics of fecal indicator bacteria in sandy sediments and the water column: a 3-year high-frequency study at a pennsylvania creek | NA | No | No | No | No | No | No | No | No | No | No | No | No | No | No | No | No |
| 141 | cryptosporidium genotyping and land use mapping for hazard identification and source tracking in a small mixed rural-urban watershed in southeastern brazil | NA | No | No | No | No | No | No | No | No | No | No | No | No | No | No | No | No |
| 143 | current state of water quality indicators in urban streams in new zealand | NA | No | No | No | No | No | No | No | No | No | No | No | No | No | No | No | No |
| 144 | decadal and seasonal water quality trends downstream of urban and rural areas in southern alberta rivers | No | No | No | No | No | No | No | No | No | No | No | No | No | No | No | No | No |
| 147 | detangling seasonal relationships of fecal contamination sources and correlates with indicators in michigan watersheds | NA | No | No | No | No | No | No | No | No | No | No | No | No | No | No | No | No |
| 153 | determinants of spatio-temporal variability of water quality in the barotse floodplain, western zambia | NA | No | No | No | No | No | No | No | No | No | No | No | No | No | No | No | No |
| 156 | determination ofwater quality of rivers under various land use activities using physico-chemical parameters and bacterial populations in northern peninsular malaysia | NA | No | No | No | No | No | No | No | No | No | No | No | No | No | No | No | No |
| 157 | determining hot spots of fecal contamination in a tropical watershed by combining land-use information and meteorological data with source-specific assays | NA | No | No | No | No | No | No | No | No | No | No | No | No | No | Yes | Yes | No |
| 158 | determining overall water quality related to anthropogenic influences across freshwater systems of thailand | NA | Yes | Yes | Yes | Yes | Yes | Yes | Yes | Yes | Yes | Yes | Yes | Yes | Yes | Yes | Yes | Yes |
| 160 | determining the primary sources of fecal pollution using microbial source tracking assays combined with land-use information in the edwards aquifer | NA | No | No | No | No | No | No | No | No | No | No | No | No | No | No | No | No |
| 163 | development and application of exceedance model for surface water quality parameters | NA | No | No | No | No | No | No | No | No | No | No | No | No | No | No | No | No |
| 164 | development and evaluation of the bacterial fate and transport module for the agricultural policy/environmental extender (apex) model | NA | No | No | No | No | No | No | No | No | No | No | No | No | No | No | No | No |
| 167 | development of a process-based model to predict pathogen budgets for the sydney drinking water catchment | NA | No | No | No | No | No | No | No | No | No | No | Yes | No | No | No | No | No |
| 168 | development of a risk-based index for source water protection planning, which supports the reduction of pathogens from agricultural activity entering water resources | NA | No | No | No | No | No | No | No | No | No | No | No | No | No | No | No | No |
| 171 | diffuse and point pollution impacts on the pathogen indicator organism level in the geum river, korea | NA | Yes | Yes | Yes | Yes | Yes | Yes | Yes | Yes | Yes | Yes | Yes | Yes | Yes | Yes | Yes | Yes |
| 172 | dissolved inorganic nitrogen, soluble reactive phosphorous, and microbial pollutant loading from tropical rural watersheds in hawai'i to the coastal ocean during non-storm conditions | NA | No | No | No | No | No | No | No | No | No | No | No | No | No | No | No | No |
| 173 | distribution and diversity of escherichia coli populations in the south nation river drainage basin, eastern ontario, canada | NA | Yes | Yes | Yes | Yes | Yes | Yes | No | No | No | Yes | Yes | Yes | Yes | No | No | No |
| 175 | dna fingerprinting using box-a1r and (gtg)(5) primers identify spatial variations of fecal contamination along pasig river, philippines | No | No | No | No | No | No | No | No | No | No | No | No | No | No | No | No | No |
| 177 | does land use affect pathogen presence in new zealand drinking water supplies? | NA | Yes | Yes | No | Yes | No | No | No | Yes | Yes | Yes | Yes | Yes | No | Yes | Yes | Yes |
| 180 | ecological water health assessment using benthic macroinvertebrate communities (case study: the ghezel ozan river in zanjan province, iran) | No | No | No | No | No | No | No | No | No | No | No | No | No | No | No | No | No |
| 183 | ecosystemic assessment of surface water quality in the virilla river: towards sanitation processes in costa rica | NA | No | No | No | No | No | No | No | No | No | No | No | No | No | No | No | No |
| 184 | effect of agricultural activities on surface water quality from paramo ecosystems | NA | No | No | No | No | No | No | No | No | No | No | No | No | No | No | No | No |
| 185 | effect of development on water quality for seven streams in north carolina | NA | Yes | No | No | No | Yes | Yes | Yes | Yes | No | Yes | Yes | Yes | No | Yes | Yes | Yes |
| 186 | effect of human development on bacteriological water quality in coastal watersheds | NA | Yes | Yes | Yes | Yes | Yes | Yes | Yes | Yes | Yes | Yes | Yes | Yes | Yes | Yes | Yes | Yes |
| 187 | effect of land use and hydrological processes on escherichia coli concentrations in streams of tropical, humid headwater catchments | NA | No | No | No | No | No | No | No | No | No | No | No | No | No | No | No | No |
| 188 | effect of land use and land cover changes on water quality in the nawuni catchment of the white volta basin, northern region, ghana | NA | No | No | No | No | No | No | No | No | No | No | No | No | No | No | No | No |
| 189 | effect of streambed bacteria release on e. coli concentrations: monitoring and modeling with the modified swat | NA | No | No | No | No | No | No | No | No | No | No | No | No | No | No | No | No |
| 190 | effects of agricultural and urban land cover on new zealand's estuarine water quality | NA | No | No | No | No | No | No | No | No | No | No | No | No | No | No | No | No |
| 191 | effects of agricultural land cover on water quality at the watershed scale in the lower kaskaskia river watershed | NA | No | No | No | No | No | No | No | No | No | No | No | Yes | No | No | No | No |
| 192 | effects of agricultural management, land use, and watershed scale on e-coli concentrations in runoff and streamflow | NA | No | No | No | No | No | No | No | No | No | No | No | No | No | No | No | No |
| 193 | effects of agricultural management, land use, and watershed scale on e. coli concentrations in runoff and streamflow | NA | No | No | No | No | No | No | No | No | No | No | No | No | No | No | No | No |
| 194 | effects of anthropic actions and forest areas on a neotropical aquatic ecosystem | NA | Yes | No | No | Yes | Yes | Yes | Yes | No | Yes | Yes | Yes | Yes | No | No | Yes | No |
| 196 | effects of changing land use on the microbial water quality of tidal creeks | NA | Yes | Yes | Yes | Yes | Yes | Yes | Yes | Yes | Yes | Yes | Yes | Yes | Yes | Yes | Yes | Yes |
| 197 | effects of future climate and land use scenarios on riverine source water quality | NA | Yes | Yes | Yes | Yes | Yes | Yes | Yes | Yes | Yes | Yes | Yes | Yes | Yes | Yes | Yes | Yes |
| 198 | effects of human activities on rivers located in protected areas of the atlantic forest | NA | No | No | No | No | No | No | No | No | No | No | No | No | No | No | No | No |
| 199 | effects of hurricanes, land use, and water management on nutrient and microbial pollution: st. lucie estuary, southeast florida | NA | No | No | No | No | No | No | No | No | No | No | No | No | No | No | No | No |
| 200 | effects of hydrological regime and land use on in-stream escherichia coli concentration in the mekong basin, lao pdr | NA | No | No | No | No | No | No | No | No | No | No | No | No | No | No | No | No |
| 201 | effects of land use and land cover changes on water quality in the umngeni river catchment, south africa | NA | No | No | No | No | No | No | No | No | No | No | No | No | No | No | No | No |
| 202 | effects of land use and land cover on water quality of low-order streams in southeastern brazil: watershed versus riparian zone | NA | Yes | Yes | Yes | No | Yes | Yes | Yes | Yes | Yes | Yes | Yes | Yes | Yes | Yes | Yes | Yes |
| 203 | effects of land use changes on water quality and flooding in upper nan river, thailand | NA | No | No | No | No | No | No | Yes | No | No | No | No | No | No | No | Yes | Yes |
| 205 | effects of land use on the number of coliform bacteria in boyong river, sleman | NA | Yes | Yes | Yes | Yes | Yes | Yes | Yes | Yes | Yes | Yes | Yes | Yes | Yes | Yes | Yes | Yes |
| 206 | effects of land uses on fecal indicator bacteria in the water and soil of a tropical watershed | NA | Yes | No | Yes | Yes | Yes | Yes | Yes | Yes | No | Yes | Yes | No | Yes | Yes | Yes | Yes |
| 210 | effects of urbanisation on the quality of the urban runoff for delhi watershed | NA | No | No | No | No | No | No | No | No | Yes | No | Yes | No | No | No | No | No |
| 212 | efficacy of bacteroides measurements for reducing the statistical uncertainty associated with hydrologic flow and fecal loads in a mixed use watershed | NA | No | No | No | No | No | No | No | No | No | No | No | No | No | No | No | No |
| 213 | elucidating the effects of land cover and usage on background escherichia coli sources in edge-of-field runoff | NA | Yes | Yes | Yes | Yes | Yes | Yes | Yes | Yes | Yes | Yes | Yes | Yes | Yes | Yes | Yes | No |
| 217 | environmental dna clarifies impacts of combined sewer overflows on the bacteriology of an urban river and resulting risks to public health | NA | No | No | No | No | No | No | No | No | No | No | No | No | No | No | No | No |
| 218 | environmental factors controlling contamination of alternative water supply points in the lefock semi-urban watershed, cameroon western highlands | NA | No | No | No | No | No | No | No | No | No | No | No | No | No | No | No | No |
| 219 | environmental fragility as an indicator of the risk of contamination by human action in watersheds used for public supply in western parana, brazil | NA | No | No | No | No | No | No | No | No | No | No | No | No | No | No | No | No |
| 224 | escherichia coli reduction by bivalves in an impaired river impacted by agricultural land use | NA | No | No | No | No | No | No | No | No | No | No | No | No | No | No | No | No |
| 225 | estimating daily potential e. coli loads in rural texas watersheds using spatially explicit load enrichment calculation tool (select) | NA | Yes | No | Yes | Yes | Yes | Yes | Yes | Yes | Yes | Yes | Yes | Yes | No | Yes | Yes | Yes |
| 226 | estimating potential e. coli sources in a watershed using spatially explicit modeling techniques | NA | No | No | No | No | No | No | No | No | No | No | No | No | No | No | No | No |
| 228 | estuarine habitat quality reflects urbanization at large spatial scales in south carolina's coastal zone | NA | Yes | Yes | Yes | Yes | Yes | Yes | No | Yes | Yes | Yes | Yes | Yes | Yes | Yes | Yes | Yes |
| 229 | evaluating land use impacts on water quality: perspectives for watershed management | NA | No | No | No | No | No | No | No | No | No | No | No | No | No | No | No | No |
| 232 | evaluating the influence of septic systems and watershed characteristics on stream faecal pollution in suburban watersheds in georgia, usa | NA | No | No | No | No | No | No | No | No | No | No | No | No | No | No | No | No |
| 234 | evaluation of a multivariate analysis modeling approach identifying sources and patterns of nonpoint fecal pollution in a mixed use watershed | NA | Yes | Yes | Yes | Yes | Yes | Yes | Yes | Yes | Yes | Yes | Yes | Yes | Yes | Yes | Yes | Yes |
| 235 | evaluation of land use and water quality in an agricultural watershed in the usa indicates multiple sources of bacterial impairment | NA | No | No | No | No | No | No | No | No | No | No | No | No | No | No | No | No |
| 236 | evaluation of recirculating sand filter in a cold climate | NA | No | No | No | No | No | No | No | No | No | No | No | No | No | No | No | No |
| 238 | evaluation of the distribution of fecal indicator bacteria in a river system depending on different types of land use in the southern watershed of the baltic sea | NA | Yes | Yes | Yes | Yes | Yes | Yes | Yes | Yes | Yes | Yes | Yes | Yes | Yes | Yes | Yes | Yes |
| 239 | evaluation of the impacts of land use in water quality and the role of nature-based solutions: a citizen science-based study | NA | No | No | No | No | Yes | No | Yes | Yes | Yes | No | Yes | No | Yes | No | Yes | No |
| 240 | evaluation of the water quality status and pollution load carrying capacity of way umpu river, way kanan district, lampung province, indonesia, based on land use | No | No | No | No | No | No | No | No | No | No | No | No | No | No | No | No | No |
| 246 | factors and mechanisms affecting seasonal changes in the prevalence of microbiological indicators of water quality and nutrient concentrations in waters of the biaka river catchment, southern poland | No | No | No | No | No | No | No | No | No | No | No | No | No | No | No | No | No |
| 247 | factors associated with e. coli levels in and salmonella contamination of agricultural water differed between north and south florida waterways | Yes | No | No | No | Yes | No | No | No | Yes | No | Yes | No | No | No | No | No | No |
| 250 | factors related to occurrence and distribution of selected bacterial and protozoan pathogens in pennsylvania streams | NA | No | No | No | No | No | No | No | No | No | No | No | No | No | No | No | No |
| 252 | faecal contamination of water and sediment in the rivers of the scheldt drainage network | NA | No | No | No | No | No | No | No | No | No | No | No | No | No | No | No | No |
| 253 | faecal indicator organism concentrations and catchment export coefficients in the uk | NA | No | No | No | No | No | No | Yes | No | No | No | No | No | No | No | No | Yes |
| 255 | faecal-indicator concentrations in waters draining lowland pastoral catchments in the uk: relationships with land use and farming practices | NA | Yes | No | Yes | Yes | Yes | Yes | Yes | Yes | Yes | Yes | Yes | Yes | Yes | No | Yes | Yes |
| 256 | fecal bacteria in the waters of an upland area in derbyshire, england: the influence of agricultural land use | NA | No | No | No | No | No | No | No | No | No | No | No | No | No | No | No | No |
| 257 | fecal coliform concentrations in the upper cohansey river watershed predicted by air temperature, discharge, and land use | NA | No | No | Yes | No | No | No | Yes | No | No | No | No | Yes | No | Yes | No | Yes |
| 258 | fecal coliform export from four coastal north carolina areas | NA | No | No | No | No | No | No | No | No | No | No | No | No | No | No | No | No |
| 259 | fecal coliform predictive model using genetic algorithm-based radial basis function neural networks (ga-rbfnns) | NA | No | No | No | No | No | No | No | No | No | No | No | No | No | No | No | No |
| 260 | fecal coliform source assessment in a small, mixed land use watershed | NA | No | No | No | No | No | No | No | No | No | No | No | No | No | Yes | No | No |
| 261 | fecal contamination and high nutrient levels pollute the watersheds of wujiang, china | NA | No | No | No | No | No | No | No | No | No | No | No | No | No | No | No | No |
| 264 | fecal indicator concentrations of surface runoff in rural watersheds, korea | NA | No | No | No | No | No | No | No | No | No | No | No | No | No | No | No | No |
| 265 | fecal indicators and antibiotic resistance genes exhibit diurnal trends in the chattahoochee river: implications for water quality monitoring | NA | No | No | No | No | No | No | No | No | No | No | No | No | No | No | No | No |
| 266 | fecal pollution source characterization in the surface waters of recharge and contributing zones of a karst aquifer using general and host-associated fecal genetic markers | NA | No | No | No | No | No | No | No | No | No | No | No | No | No | No | No | No |
| 269 | field and modelling studies of escherichia coli loads in tropical streams of montane agro-ecosystems | NA | Yes | Yes | Yes | Yes | Yes | Yes | Yes | Yes | Yes | Yes | Yes | Yes | Yes | Yes | Yes | Yes |
| 272 | fish assemblages and water quality in pampean streams (argentina) along an urbanization gradient | NA | No | No | No | No | No | No | No | No | No | No | No | No | No | No | No | No |
| 275 | fourier landscape pattern indices for predicting south carolina watershed fecal coliform | No | No | No | No | No | No | No | No | No | No | No | No | No | No | No | No | No |
| 277 | generic modelling of faecal indicator organism concentrations in the uk | Yes | Yes | Yes | Yes | Yes | Yes | Yes | Yes | Yes | Yes | No | Yes | Yes | Yes | Yes | Yes | Yes |
| 278 | genetic fecal source identification in urban streams impacted by municipal separate storm sewer system discharges | Yes | No | No | No | Yes | No | No | No | No | No | No | No | No | No | No | No | No |
| 280 | geo-environmental impacts of hydrogeological setting and anthropogenic activities on water quality in the quaternary aquifer southeast of the nile delta, egypt | NA | No | No | No | No | No | No | No | No | No | No | No | No | No | No | No | No |
| 281 | geochemical characterization and health risk assessment in two diversified environmental settings (southern italy) | NA | No | No | No | No | No | No | No | No | No | No | No | No | No | No | No | No |
| 287 | grazing intensity is a poor indicator of waterborne escherichia coli o157 activity | NA | Yes | Yes | Yes | Yes | Yes | Yes | Yes | No | No | Yes | Yes | Yes | Yes | Yes | Yes | Yes |
| 289 | groupwise modeling study of bacterially impaired watersheds in texas: clustering analysis | No | No | No | No | No | No | No | No | No | No | No | No | No | No | No | No | No |
| 290 | growing season surface water loading of fecal indicator organisms within a rural watershed | No | No | No | No | No | No | No | No | No | No | No | No | No | Yes | No | No | No |
| 293 | health risk assessment related to waterborne pathogens from the river to the tap | NA | No | No | No | No | No | No | No | No | No | No | No | No | No | No | No | No |
| 295 | high spatial resolution landscape indicators show promise in explaining water quality in urban streams | NA | Yes | Yes | Yes | Yes | Yes | Yes | Yes | Yes | Yes | Yes | Yes | Yes | Yes | Yes | Yes | Yes |
| 297 | how do different modalities of land use practices impact the environmental features and macroinvertebrates? an assessment of mountain streams from patagonia, argentina | NA | Yes | Yes | Yes | Yes | Yes | No | Yes | Yes | Yes | Yes | Yes | Yes | Yes | No | Yes | No |
| 299 | hubac and nifh source tracking markers display a relationship to land use but not rainfall | NA | Yes | Yes | Yes | Yes | Yes | Yes | Yes | Yes | Yes | Yes | Yes | Yes | Yes | Yes | Yes | Yes |
| 300 | human and animal microbial source tracking in a tropical river with multiple land use activities | NA | No | No | No | No | No | No | No | No | No | No | No | No | No | No | No | No |
| 301 | human development is linked to multiple water body impairments along the california coast | NA | No | No | No | No | No | No | No | No | No | No | No | No | No | No | Yes | No |
| 303 | human fecal pollution monitoring and microbial risk assessment for water reuse potential in a coastal industrial-residential mixed-use watershed | NA | No | No | No | No | No | No | No | No | No | No | No | No | No | No | No | No |
| 304 | human health risk assessment and environmental distribution of trace elements, glyphosate, fecal coliform and total coliform in atlantic rainforest mountain rivers (south america) | NA | No | No | No | No | Yes | No | No | No | No | Yes | No | No | No | No | No | No |
| 307 | humans and hoofed livestock are the main sources of fecal contamination of rivers used for crop irrigation: a microbial source tracking approach | NA | Yes | Yes | Yes | Yes | Yes | Yes | Yes | Yes | Yes | Yes | Yes | Yes | Yes | Yes | Yes | Yes |
| 308 | hydrochemical evidence of the depth of penetration of anthropogenic recharge in sandstone aquifers underlying two mature cities in the uk | NA | No | No | No | No | No | No | No | No | No | No | No | No | No | No | No | No |
| 310 | hydrochemistry of shallow groundwater and springs used for potable supply in southern brazil | NA | No | No | No | No | No | No | No | Yes | No | Yes | No | No | No | No | No | No |
| 317 | identifying sources of fecal pollution in the colville river using library-independent genetic markers | No | No | No | No | No | No | No | No | No | No | No | No | No | No | No | No | No |
| 318 | impact of changes of land use on water quality, from tropical forest to anthropogenic occupation: a multivariate approach | Yes | No | No | No | No | No | No | No | No | No | No | No | No | No | No | No | No |
| 319 | impact of construction and functioning of a newly built ski slope on the quality of nearby stream water | NA | No | No | No | No | No | No | Yes | No | No | No | No | No | No | Yes | Yes | No |
| 321 | impact of home industries on water quality in a tributary of the marimba river, harare: implications for urban water management | NA | No | No | No | No | No | No | No | No | No | No | No | No | No | No | No | No |
| 322 | impact of land use and urban runoff on the contamination of the sarno river basin in southwestern italy | NA | No | No | No | No | No | No | No | No | No | No | No | No | No | No | No | No |
| 324 | impact of land use on the faecal microbial quality of hill-country streams | NA | Yes | Yes | Yes | Yes | Yes | Yes | Yes | Yes | Yes | Yes | No | Yes | Yes | Yes | Yes | Yes |
| 325 | impact of land use on water quality in the likangala catchment, southern malawi | NA | No | No | No | No | No | No | No | No | No | No | No | No | No | No | No | No |
| 326 | impact of land uses, drought, flood, wildfire, and cascading events on water quality and microbial communities: a review and analysis | NA | No | No | No | No | No | No | No | No | No | No | No | No | No | No | No | No |
| 327 | impact of land-use/land-cover dynamics on water quality in the upper lilongwe river basin, malawi | NA | Yes | Yes | Yes | Yes | Yes | Yes | Yes | Yes | Yes | Yes | Yes | Yes | Yes | Yes | Yes | Yes |
| 329 | impact of urbanization and agriculture on the occurrence of bacterial pathogens and stx genes in coastal waterbodies of central california | NA | Yes | Yes | Yes | Yes | Yes | Yes | Yes | Yes | Yes | Yes | Yes | Yes | Yes | Yes | Yes | Yes |
| 330 | impact of urbanization on the water quality of the uberaba river and tributaries. | NA | Yes | Yes | Yes | Yes | Yes | Yes | Yes | Yes | Yes | Yes | Yes | Yes | Yes | Yes | Yes | Yes |
| 331 | impact on water quality of land uses along thamalakane-boteti river: an outlet of the okavango delta | NA | No | No | No | No | No | No | No | No | No | No | No | No | No | No | No | No |
| 332 | impacts of droughts and heatwaves on river water quality worldwide | NA | No | No | No | No | No | No | No | No | Yes | No | No | No | No | No | No | No |
| 335 | impacts of manure management practices on stream microbial loading into conesus lake, ny | NA | No | No | No | No | No | No | No | No | No | No | No | No | No | No | No | No |
| 336 | impacts of tropical forest cover on water quality in agricultural watersheds in southeastern brazil | NA | Yes | Yes | Yes | Yes | Yes | Yes | Yes | Yes | Yes | Yes | Yes | Yes | Yes | Yes | No | Yes |
| 337 | impacts of urbanization on the prevalence of antibiotic-resistant escherichia coli in the chaophraya river and its tributaries | NA | No | No | No | No | No | No | No | No | No | No | No | No | No | No | No | No |
| 338 | implementation and integration of microbial source tracking in a river watershed monitoring plan | NA | Yes | Yes | No | Yes | Yes | Yes | Yes | Yes | Yes | Yes | Yes | Yes | Yes | Yes | No | Yes |
| 340 | implications of demographic changes and land transformations on surface water quality of rural and urban subbasins of upper bhima river basin, maharashtra, india | NA | No | No | No | No | No | No | No | No | No | No | No | No | No | No | No | No |
| 341 | importance of land use factors in the prediction of water quality of the upper green river watershed, kentucky, usa, using random forest | NA | No | No | No | No | No | No | No | No | No | No | No | No | No | No | No | No |
| 342 | improved management of farm dams increases vegetation cover, water quality, and macroinvertebrate biodiversity | NA | No | No | No | No | No | No | No | No | No | No | No | No | No | No | No | No |
| 343 | improving the economic and environmental performance of a new zealand hill country farm catchment: 3. short-term outcomes of land-use change | NA | No | No | No | No | No | No | No | No | No | No | No | No | No | No | No | No |
| 344 | in-stream escherichia coli modeling using high-temporal-resolution data with deep learning and process-based models | NA | No | No | No | No | No | No | Yes | No | No | No | No | No | No | No | No | No |
| 345 | inadequate riparian zone use directly decreases water quality of a low-order urban stream in southern brazil | NA | Yes | Yes | Yes | Yes | Yes | Yes | Yes | Yes | Yes | Yes | Yes | Yes | Yes | Yes | Yes | Yes |
| 346 | incorporating water quality into land use scenario analysis with random forest models | NA | Yes | Yes | Yes | Yes | Yes | No | Yes | Yes | Yes | Yes | Yes | Yes | Yes | Yes | Yes | Yes |
| 349 | influence of climate change, tidal mixing, and watershed urbanization on historical water quality in newport bay, a saltwater wetland and tidal embayment in southern california | NA | No | No | No | No | No | No | No | No | No | No | No | No | No | No | No | No |
| 352 | influence of land use and nutrient flux on metabolic activity of e. coli o157 in river water | NA | Yes | Yes | Yes | Yes | Yes | Yes | Yes | Yes | Yes | Yes | Yes | Yes | Yes | Yes | Yes | Yes |
| 353 | influence of land use land cover on river water quality in rural north wales, uk | NA | Yes | Yes | Yes | No | No | Yes | Yes | No | Yes | Yes | Yes | No | Yes | No | Yes | Yes |
| 357 | influence of rainy season and land use on drinking water quality in a karst landscape, state of yucatan, mexico | NA | No | No | No | No | No | No | No | No | No | No | No | No | No | No | No | No |
| 360 | influential factors in surface water quality in catchments within the pampa biome with different land use | NA | No | No | No | No | No | No | No | No | No | No | No | No | No | No | No | No |
| 365 | instream coliform gradients in the holtemme, a small headwater stream in the elbe river basin, northern germany | NA | No | No | No | No | No | No | No | No | No | No | No | No | No | No | No | No |
| 366 | integral assessment of pollution in the suquia river (cordoba, argentina) as a contribution to lotic ecosystem restoration programs | NA | No | No | No | No | No | No | No | No | No | No | No | No | No | No | No | No |
| 367 | integrated approach for quantitative estimation of particulate organic carbon sources in a complex river system | NA | No | No | No | No | No | No | No | No | No | No | No | No | No | No | No | No |
| 368 | integrating environmental and socio-economic indicators of a linked catchment-coastal system using variable environmental intensity | NA | Yes | Yes | No | Yes | No | No | No | No | Yes | Yes | No | Yes | Yes | Yes | Yes | No |
| 369 | integration of remote sensing data and in situ measurements to monitor the water quality of the ismailia canal, nile delta, egypt | NA | Yes | Yes | No | No | Yes | No | No | No | No | Yes | Yes | Yes | Yes | Yes | Yes | No |
| 370 | integrative survey of 68 non-overlapping upstate new york watersheds reveals stream features associated with aquatic fecal contamination | NA | No | No | Yes | No | No | No | No | No | No | No | No | No | No | No | No | No |
| 371 | intra-event variability of bacterial composition in stormwater runoff from mixed land use and land cover catchment | NA | No | No | No | No | No | No | No | No | No | No | No | No | No | No | No | No |
| 380 | land cover impacts on stream nutrients and fecal coliform in the lower piedmont of west georgia | Yes | Yes | Yes | Yes | Yes | Yes | Yes | Yes | Yes | Yes | Yes | Yes | Yes | Yes | Yes | Yes | Yes |
| 381 | land use and environmental variables influence tetracycline-resistant bacteria occurrence in southeastern coastal plain streams | Yes | Yes | Yes | Yes | Yes | Yes | Yes | Yes | Yes | Yes | Yes | Yes | Yes | Yes | Yes | Yes | Yes |
| 382 | land use and hydroclimatic influences on faecal indicator organisms in two large scottish catchments: towards land use-based models as screening tools | Yes | Yes | Yes | Yes | No | Yes | Yes | Yes | No | No | No | Yes | No | Yes | Yes | Yes | Yes |
| 383 | land use and land cover changes in zezere watershed (portugal)--water quality implications | NA | No | No | No | No | No | No | No | No | No | No | No | No | No | No | Yes | No |
| 385 | land use and water quality in a rural cloud forest region (intag, ecuador) | NA | No | No | No | No | No | No | No | No | No | No | No | No | No | No | No | No |
| 386 | land use and water quality in guangzhou, china: a survey of ecological and social vulnerability in four urban units of the rapidly developing megacity | NA | No | No | No | No | No | No | No | No | No | No | No | No | No | No | No | No |
| 388 | land use as a critical determinant of faecal and antimicrobial resistance gene pollution in riverine systems | NA | Yes | Yes | Yes | No | Yes | Yes | Yes | Yes | No | Yes | Yes | Yes | Yes | Yes | Yes | Yes |
| 389 | land use effects on water quality in the urban agglomeration of cuiaba and varzea grande, mato grosso state, central brazil | NA | No | No | No | Yes | Yes | Yes | No | No | No | Yes | No | No | No | No | Yes | Yes |
| 390 | land use impact on the water quality of large tropical river: mun river basin, thailand | NA | Yes | Yes | Yes | Yes | Yes | Yes | Yes | Yes | No | Yes | No | Yes | Yes | Yes | Yes | No |
| 392 | land use land cover changes in detection of water quality: a study based on remote sensing and multivariate statistics | NA | No | No | No | No | No | No | No | No | No | No | No | No | No | No | No | No |
| 393 | land use practices and elevated levels of escherichia coli in the coosawattee river, georgia | NA | Yes | Yes | Yes | Yes | Yes | Yes | Yes | Yes | Yes | Yes | Yes | Yes | Yes | Yes | Yes | Yes |
| 394 | land use, weather, and water quality factors associated with fecal contamination of northeastern streams that span an urban-rural gradient | NA | Yes | Yes | Yes | Yes | Yes | Yes | Yes | Yes | No | Yes | Yes | Yes | Yes | Yes | Yes | Yes |
| 397 | land-use impact on water quality of the opak sub-watershed, yogyakarta, indonesia | NA | No | No | No | No | No | No | No | No | No | No | No | No | No | No | No | No |
| 400 | land-use-mediated escherichia coli concentrations in a contemporary appalachian watershed | NA | Yes | Yes | Yes | Yes | Yes | Yes | Yes | Yes | Yes | Yes | Yes | Yes | Yes | Yes | Yes | Yes |
| 402 | landscape and seasonal factors influence salmonella and campylobacter prevalence in a rural mixed use watershed | NA | No | No | No | No | No | No | No | No | No | No | No | No | No | No | No | No |
| 403 | landscape drivers and social dynamics shaping microbial contamination risk in three maya communities in southern belize, central america | NA | Yes | No | Yes | Yes | No | Yes | No | Yes | Yes | Yes | Yes | Yes | Yes | Yes | Yes | Yes |
| 405 | landscape-scale factors affecting the prevalence of escherichia coli in surface soil include land cover type, edge interactions, and soil ph | NA | No | No | No | Yes | Yes | No | Yes | No | Yes | No | No | No | Yes | No | No | Yes |
| 406 | large-scale implementation of standardized quantitative real-time pcr fecal source identification procedures in the tillamook bay watershed | NA | No | No | No | No | No | No | No | No | No | No | No | No | No | No | No | No |
| 407 | learning hierarchical bayesian networks to assess the interaction effects of controlling factors on spatiotemporal patterns of fecal pollution in streams | NA | No | No | No | No | No | No | No | No | No | No | No | No | No | No | No | No |
| 408 | level and transport pattern of faecal coliform bacteria from tropical urban catchments | NA | No | No | No | No | No | No | Yes | No | Yes | No | No | No | No | Yes | No | No |
| 409 | levels and patterns of fecal indicator bacteria in stormwater runoff from homogenous land use sites and urban watersheds | NA | Yes | Yes | Yes | Yes | Yes | Yes | Yes | Yes | Yes | Yes | Yes | Yes | Yes | Yes | Yes | Yes |
| 410 | linkages between tidal creek ecosystems and the landscape and demographic attributes of their watersheds | NA | No | No | No | No | No | No | No | No | No | No | No | No | No | No | No | No |
| 412 | linking land-use type and stream water quality using spatial data of fecal indicator bacteria and heavy metals in the yeongsan river basin | NA | Yes | Yes | Yes | Yes | Yes | Yes | Yes | Yes | Yes | Yes | Yes | Yes | Yes | Yes | Yes | Yes |
| 414 | macroinvertebrate indices versus microbial fecal pollution characteristics for water quality monitoring reveals contrasting results for an ethiopian river | No | Yes | Yes | No | Yes | No | Yes | Yes | No | Yes | Yes | Yes | Yes | No | Yes | Yes | Yes |
| 418 | mapping the potential risk of escherichia coli leaching through soils of the waikato river catchment, new zealand | NA | No | No | No | No | No | No | No | No | No | No | No | No | No | No | No | No |
| 423 | microbial find, inform, and test model for identifying spatially distributed contamination sources: framework foundation and demonstration of ruminant bacteroides abundance in river sediments | NA | No | No | No | No | No | No | No | No | No | No | No | No | No | No | No | No |
| 425 | microbial source tracking (mst) in chattahoochee river national recreasion area: seasonal and precipitation in mst marker concentrations, and associations with e. coli levels, pathogenic marker presence, and land use | NA | Yes | Yes | Yes | Yes | No | Yes | Yes | Yes | Yes | Yes | No | No | No | Yes | No | Yes |
| 426 | microbial source tracking to elucidate the impact of land-use and physiochemical water quality on fecal contamination in a mixed land-use watershed | NA | Yes | Yes | Yes | Yes | Yes | Yes | Yes | Yes | Yes | Yes | Yes | Yes | Yes | Yes | Yes | Yes |
| 428 | microbial source-tracking reveals origins of fecal contamination in a recoveringwatershed | NA | No | No | No | No | No | No | No | No | No | No | No | No | No | No | No | No |
| 429 | microbial water pollution: a screening tool for initial catchment-scale assessment and source apportionment | NA | No | No | Yes | No | Yes | Yes | No | No | No | No | No | No | No | No | No | Yes |
| 430 | microbial water quality and influences of fecal accumulation from a dog exercise area | NA | Yes | Yes | Yes | Yes | Yes | Yes | Yes | Yes | Yes | Yes | Yes | Yes | Yes | Yes | Yes | Yes |
| 431 | microbiological quality assessment of watershed associated with animal-based agriculture in santa catarina, brazil | NA | Yes | Yes | Yes | Yes | Yes | Yes | Yes | Yes | Yes | Yes | Yes | Yes | Yes | Yes | Yes | Yes |
| 435 | modeling spatiotemporal bacterial variability with meteorological and watershed land-use characteristics | NA | Yes | Yes | Yes | Yes | Yes | Yes | Yes | Yes | Yes | Yes | Yes | Yes | Yes | Yes | Yes | Yes |
| 436 | modeling the dispersion of e. coli in waterbodies due to urban sources: a spatial approach | NA | No | No | No | No | No | No | No | No | No | No | No | No | No | No | No | No |
| 437 | modeling the impact of land use change on basin-scale transfer of fecal indicator bacteria: swat model performance | NA | No | No | No | No | No | No | No | No | No | No | No | No | No | No | No | No |
| 438 | modeling the relationship between land use and surface water quality | NA | Yes | Yes | Yes | Yes | Yes | Yes | Yes | Yes | Yes | Yes | Yes | Yes | Yes | Yes | Yes | Yes |
| 439 | modelling faecal bacteria pathways in receiving waters | NA | No | No | No | No | No | No | No | No | No | No | No | No | No | No | No | No |
| 440 | modelling faecal indicator concentrations in large rural catchments using land use and topographic data | NA | Yes | Yes | Yes | No | No | Yes | Yes | Yes | Yes | Yes | Yes | Yes | Yes | Yes | Yes | Yes |
| 441 | modelling microbiological water quality in the seine river drainage network: past, present and future situations | NA | No | No | No | No | No | No | No | No | No | No | No | No | No | No | No | No |
| 442 | modelling of faecal indicator bacteria (fib) in the red river basin (vietnam) | NA | No | No | No | No | No | No | No | No | No | No | No | No | No | No | No | No |
| 443 | modelling the hydrologic effects of land-use and climate changes | NA | Yes | Yes | Yes | Yes | Yes | Yes | Yes | Yes | Yes | Yes | Yes | Yes | Yes | Yes | Yes | Yes |
| 444 | modelling the impact of future socio-economic and climate change scenarios on river microbial water quality | NA | Yes | Yes | Yes | Yes | Yes | Yes | Yes | Yes | Yes | Yes | No | Yes | Yes | Yes | Yes | No |
| 447 | molecular tracers of soot and sewage contamination in streams supplying new york city drinking water | NA | No | No | No | No | No | No | No | No | No | No | No | No | No | No | No | No |
| 448 | monitoring and evaluation of the water quality of the lower neches river, texas, usa | NA | No | No | No | No | No | Yes | No | No | Yes | No | No | No | No | No | No | No |
| 449 | monitoring and predicting the fecal indicator bacteria concentrations from agricultural, mixed land use and urban stormwater runoff | NA | Yes | Yes | Yes | Yes | Yes | No | Yes | Yes | Yes | Yes | Yes | No | Yes | Yes | Yes | Yes |
| 451 | monitoring of spunky bottoms restored wetland in southern illinois for biotic and abiotic pollution indicators | NA | No | No | No | No | No | No | No | No | No | No | No | No | No | No | No | No |
| 452 | motueka river plume facilitates transport of ruminant faecal contaminants into shellfish growing waters, tasman bay, new zealand | NA | No | No | No | No | No | No | No | No | No | No | No | No | No | No | No | No |
| 453 | multi-scale landscape factors influencing stream water quality in the state of oregon | NA | No | No | No | No | No | Yes | Yes | No | No | Yes | No | No | No | Yes | Yes | Yes |
| 455 | multiple modes of water quality impairment by fecal contamination in a rapidly developing coastal area: southwest brunswick county, north carolina | NA | No | No | No | No | No | No | No | No | No | No | No | No | No | No | No | No |
| 456 | multiscale spatiotemporal variability of fecal indicator bacteria and associated particle size distributions in the sandy bottom sediments of a pennsylvania creek | No | No | No | No | No | No | No | No | No | No | No | No | No | No | No | No | No |
| 457 | native forest cover safeguards stream water quality under a changing climate | Yes | Yes | Yes | Yes | Yes | Yes | Yes | Yes | Yes | Yes | Yes | Yes | Yes | Yes | Yes | Yes | Yes |
| 462 | occurrence and distribution of microbiological indicators in groundwater and stream water | Yes | Yes | No | Yes | Yes | No | No | Yes | Yes | No | Yes | Yes | Yes | Yes | Yes | Yes | Yes |
| 464 | occurrence of microbial indicators, pathogenic bacteria and viruses in tropical surface waters subject to contrasting land use | NA | No | No | No | No | No | No | No | No | No | No | No | No | No | No | No | No |
| 465 | occurrence of traditional and alternative fecal indicators in tropical urban environments under different land use patterns | Yes | Yes | Yes | Yes | Yes | Yes | Yes | Yes | Yes | Yes | Yes | Yes | Yes | Yes | Yes | Yes | Yes |
| 467 | participatory approach for more robust water resource management: case study of the santa rosa sub-watershed of the philippines | No | Yes | Yes | Yes | Yes | Yes | Yes | Yes | Yes | No | Yes | No | Yes | Yes | Yes | Yes | No |
| 470 | patterns and drivers of fecal coliform exports in a typhoon-affected watershed: insights from 10-year observations and swat model | NA | Yes | Yes | Yes | Yes | No | Yes | Yes | Yes | Yes | Yes | No | Yes | Yes | Yes | Yes | Yes |
| 471 | patterns in water quality on canadian shores of lake ontario: correspondence with proximity to land and level of urbanization | NA | No | No | No | No | No | No | No | No | No | No | No | No | No | No | No | No |
| 473 | patterns of host-associated fecal indicators driven by hydrology, precipitation, and land use attributes in great lakes watersheds | NA | Yes | Yes | Yes | Yes | Yes | Yes | Yes | Yes | Yes | Yes | Yes | Yes | Yes | Yes | Yes | Yes |
| 476 | physico-chemical and biological characteristics of mountainous streams under different land uses of mid hills of himachal pradesh | NA | No | No | No | No | No | No | No | No | No | No | No | No | No | No | No | No |
| 478 | pine afforestation and stream health: a comparison of land-use in two soft rock catchments, east cape, new zealand | NA | No | No | No | Yes | No | No | No | No | No | No | No | No | Yes | No | No | No |
| 479 | point and non-point microbial source pollution: a case study of delhi | NA | No | No | No | Yes | Yes | No | Yes | Yes | Yes | No | No | No | No | No | No | No |
| 480 | pollution in qaraaoun lake, central lebanon | NA | No | No | Yes | No | No | No | No | No | No | No | No | No | No | No | No | No |
| 484 | potential impacts of climate and land use change on the water quality of ganga river around the industrialized kanpur region | NA | No | No | No | Yes | Yes | No | Yes | No | No | No | No | No | No | No | No | Yes |
| 485 | potential pollutant sources in a choptank river (usa) subwatershed and the influence of land use and watershed characteristics | NA | No | No | No | No | No | No | No | No | No | No | No | No | No | No | No | No |
| 487 | precipitation-driven anthropogenic pollutant fluctuations within standing water sources of the edwards aquifer region, texas | NA | No | No | No | No | No | No | No | No | No | No | No | No | No | No | No | No |
| 488 | predicting diffuse microbial pollution risk across catchments: the performance of scimap and recommendations for future development | NA | No | No | Yes | Yes | No | Yes | No | No | Yes | No | Yes | No | No | No | Yes | Yes |
| 489 | predicting faecal indicator fluxes using digital land use data in the uk's sentinel water framework directive catchment: the ribble study | NA | Yes | Yes | Yes | No | Yes | Yes | Yes | No | Yes | Yes | Yes | Yes | Yes | Yes | Yes | Yes |
| 490 | predicting fecal indicator bacteria using spatial stream network models in a mixed-land-use suburban watershed in new jersey, usa | NA | Yes | Yes | Yes | Yes | Yes | Yes | Yes | Yes | Yes | Yes | Yes | Yes | Yes | Yes | Yes | Yes |
| 491 | predicting fecal indicator organism contamination in oregon coastal streams | NA | Yes | Yes | Yes | Yes | Yes | Yes | Yes | Yes | Yes | Yes | Yes | Yes | Yes | Yes | No | Yes |
| 494 | predicting microbial pollution concentrations in uk rivers in response to land use change | NA | No | No | No | No | No | No | No | No | No | No | No | No | No | No | No | No |
| 495 | predicting stream water quality under different urban development pattern scenarios with an interpretable machine learning approach | NA | Yes | Yes | Yes | Yes | Yes | Yes | Yes | Yes | Yes | Yes | Yes | Yes | Yes | Yes | Yes | Yes |
| 496 | predicting the fate and transport of e. coli in two texas river basins using a spatially referenced regression model | NA | Yes | Yes | Yes | Yes | Yes | Yes | Yes | Yes | Yes | Yes | Yes | Yes | Yes | Yes | Yes | Yes |
| 502 | quantification of fecal coliform inputs to aquatic systems through soil leaching | Yes | Yes | Yes | No | No | No | Yes | No | Yes | Yes | Yes | Yes | Yes | Yes | Yes | Yes | Yes |
| 504 | quantification of microbial source tracking and pathogenic bacterial markers in water and sediments of tiaoxi river (taihu watershed) | No | No | No | No | No | No | No | No | No | No | No | No | No | No | No | No | No |
| 506 | quantifying escherichia coli and suspended particulate matter concentrations in a mixed-land use appalachian watershed | NA | No | No | No | No | No | Yes | No | No | No | No | No | Yes | No | No | No | Yes |
| 509 | quantifying the variability in escherichia coli (e. coli) throughout storm events at a karst spring in northwestern arkansas, united states | NA | No | No | No | No | No | No | No | No | No | No | No | No | No | Yes | No | No |
| 512 | rainfall driven e. coli transfer to the stream conduit network observed through increasing spatial scales in mixed land use paddy farming karst terrain | No | No | No | No | No | No | No | No | No | No | No | No | No | No | No | No | No |
| 513 | reach specificity in sediment e. coli population turnover and interaction with waterborne populations | No | No | No | No | No | No | No | No | No | No | No | No | No | No | No | No | No |
| 516 | recreational disturbance of river sediments during base flow deteriorates microbial water quality | NA | No | Yes | Yes | No | No | Yes | Yes | No | Yes | Yes | No | No | No | No | Yes | No |
| 518 | regional variations of bovine and porcine fecal pollution as a function of landscape, nutrient, and hydrological factors | NA | No | No | No | No | No | No | No | No | No | No | No | No | No | No | No | No |
| 519 | relating watershed characteristics to elevated stream escherichia coli levels in agriculturally dominated landscapes: an iowa case study | NA | Yes | Yes | No | Yes | Yes | Yes | Yes | Yes | Yes | Yes | Yes | Yes | No | Yes | Yes | Yes |
| 520 | relations of the groundwater quality and disorderly occupation in an amazon low-income neighborhood developed over a former dump area, santarem/pa, brazil | NA | No | No | No | No | No | No | No | No | No | No | No | No | No | No | No | No |
| 522 | relationship between land use and water quality in a watershed impacted by iron ore tailings and domestic sewage | NA | No | No | No | No | No | No | No | No | No | No | No | No | No | No | No | No |
| 523 | relationships between intra-aggregate pore structures and distributions of escherichia coli within soil macro-aggregates | NA | No | No | No | No | No | No | No | No | No | No | No | No | No | No | No | No |
| 525 | relationships between land use patterns and water quality in the pong river basin, northeast thailand | NA | Yes | Yes | Yes | Yes | Yes | Yes | Yes | Yes | Yes | Yes | Yes | Yes | Yes | Yes | Yes | Yes |
| 526 | respective contributions of point and non-point sources of e. coli and enterococci in a large urbanized watershed (the seine river, france) | NA | Yes | Yes | Yes | Yes | Yes | Yes | Yes | Yes | Yes | Yes | Yes | Yes | Yes | Yes | Yes | Yes |
| 527 | responses of stream macroinvertebrate communities and water quality of five dairy farming streams following adoption of mitigation practices | NA | No | No | No | No | No | No | No | No | No | No | No | No | No | No | No | No |
| 528 | riparian buffers: disrupting the transport of e. coli from rural catchments to streams | NA | Yes | Yes | Yes | Yes | Yes | No | Yes | Yes | Yes | Yes | Yes | Yes | Yes | No | Yes | Yes |
| 529 | riparian protection and on-farm best management practices for restoration of a lowland stream in an intensive dairy farming catchment: a case study | NA | No | No | No | No | No | No | No | No | No | No | No | No | No | No | No | No |
| 532 | roadside ditches as conduits of fecal indicator organisms and sediment: implications for water quality management | NA | Yes | Yes | Yes | Yes | Yes | Yes | Yes | Yes | Yes | No | Yes | Yes | Yes | No | Yes | Yes |
| 534 | sanitary analyses of runoff water a river | No | No | No | No | No | No | No | No | No | No | No | No | No | No | No | No | No |
| 536 | scale-dependence of land use effects on water quality of streams in agricultural catchments | NA | No | No | No | No | No | No | No | No | No | No | No | No | No | No | No | No |
| 538 | searching for balance between hill country pastoral farming and nature | NA | No | No | No | Yes | No | No | No | No | No | No | Yes | No | No | No | No | No |
| 539 | seasonal variation of surface water quality and streamflow in rispana: a tributary of ganges river, india | NA | No | No | No | No | No | No | No | No | No | No | No | No | No | No | No | No |
| 543 | sensitivity of streamflow and microbial water quality to future climate and land use change in the west of ireland | NA | No | No | No | No | No | No | No | No | No | No | No | No | No | No | No | No |
| 545 | simulating fecal coliform bacteria loading from an urbanizing watershed | NA | No | No | No | No | No | No | No | No | No | No | No | No | No | No | No | No |
| 547 | sources and fate of salmonella and fecal indicator bacteria in an urban creek | NA | No | No | No | No | No | No | No | No | No | No | No | No | No | No | No | No |
| 548 | sources and management of urban stormwater pollution in rural catchments, australia | NA | No | No | No | No | No | No | No | No | No | No | No | No | No | No | No | No |
| 549 | sources and persistence of fecal coliform bacteria in a rural watershed | NA | No | No | No | No | No | No | No | No | No | No | No | No | No | No | No | No |
| 550 | sources of nutrients and fecal indicator bacteria to nearshore waters on the north shore of kaua'i (hawai'i, usa) | NA | No | No | No | No | No | No | No | No | No | No | No | No | No | No | No | No |
| 554 | spatial and temporal bacterial quality of a lowland agricultural stream in northeast scotland | NA | Yes | Yes | Yes | Yes | Yes | Yes | Yes | Yes | No | Yes | Yes | Yes | No | Yes | Yes | No |
| 555 | spatial and temporal characterization of escherichia coli, suspended particulate matter and land use practice relationships in a mixed-land use contemporary watershed | NA | Yes | Yes | Yes | Yes | Yes | Yes | Yes | Yes | Yes | Yes | Yes | Yes | Yes | Yes | Yes | Yes |
| 558 | spatial and temporal dynamics of suspended particles and e. coli in a complex surface-water and karst groundwater system as a basis for an adapted water protection scheme, northern vietnam | NA | No | No | No | No | No | No | No | No | No | No | No | No | No | No | No | No |
| 561 | spatial and temporal variation of fecal indicator organisms in two creeks in beltsville, maryland | NA | No | No | No | No | No | No | No | No | No | No | No | No | No | No | No | No |
| 562 | spatial and temporal variations in microbiological water quality of the river wiwi in kumasi, ghana | NA | No | Yes | No | Yes | No | No | No | No | No | No | No | No | No | No | Yes | No |
| 563 | spatial and temporal variations in pollution indicator bacteria in the lower vaal river, south africa | NA | No | No | No | No | No | No | No | No | No | No | No | No | No | Yes | No | No |
| 566 | spatial assessment of water quality in the vicinity of lake alice national wildlife refuge, upper devils lake basin, north dakota | NA | No | No | No | No | No | No | No | No | No | No | No | No | No | No | No | No |
| 568 | spatial distribution of coliform bacteria in batang arau river, padang, west sumatera, indonesia | NA | No | No | No | No | No | No | No | No | No | No | No | No | No | No | No | No |
| 569 | spatial pattern assessment of lake kivu basin rivers water quality using national sanitation foundation water quality and rivers pollution indices | NA | No | No | No | No | No | No | No | No | No | No | No | No | No | No | No | No |
| 570 | spatial patterns of enzymatic activity in large water bodies: ship-borne measurements of beta-d-glucuronidase activity as a rapid indicator of microbial water quality | NA | No | No | No | No | No | No | No | No | No | No | No | No | No | No | No | No |
| 572 | spatial scale of land-use impacts on riverine drinking source water quality | NA | Yes | Yes | No | Yes | Yes | Yes | Yes | Yes | Yes | Yes | No | Yes | Yes | Yes | Yes | No |
| 575 | spatially explicit pollutant load integrated in stream e. coli concentration modeling in a mixed land use catchment | NA | No | No | No | No | No | No | No | No | No | No | No | No | No | No | No | No |
| 576 | spatio-temporal analysis of urban changes and surface water quality | NA | Yes | Yes | Yes | Yes | Yes | Yes | Yes | Yes | Yes | Yes | Yes | Yes | No | Yes | Yes | Yes |
| 577 | spatio-temporal distribution of fecal indicators in three rivers of the haihe river basin, china | NA | No | No | No | No | No | No | No | No | No | No | No | No | No | No | Yes | No |
| 578 | spatio-temporal variation of water quality in the yan oya river basin, sri lanka | NA | No | No | No | No | No | No | No | No | No | No | No | No | No | No | No | No |
| 579 | spatiotemporal analysis of cryptosporidium species/genotypes and relationships with other zoonotic pathogens in surface water from mixed-use watersheds | NA | No | No | No | No | No | No | No | No | No | No | No | No | No | No | No | No |
| 580 | spatiotemporal characteristics of the water quality in the jinsha river basin (panzhihua, china) | NA | No | No | No | No | No | No | No | No | No | No | No | No | No | No | No | No |
| 581 | spatiotemporal characterization of water chemistry and pollution sources of the umhlatuzana, umbilo and amanzimnyama river catchments of durban, kwazulu-natal, south africa | NA | No | No | No | No | No | No | No | No | No | No | No | No | No | No | No | No |
| 582 | spatiotemporal variability and key influencing factors of river fecal coliform within a typical complex watershed | NA | No | Yes | No | No | Yes | No | No | Yes | Yes | No | No | No | Yes | No | No | Yes |
| 583 | spatiotemporal variation and the role of wildlife in seasonal water quality declines in the chobe river, botswana | NA | No | No | No | Yes | Yes | No | No | Yes | Yes | No | Yes | Yes | No | No | No | Yes |
| 584 | spatiotemporal variation of bacterial water quality and the relationship with pasture land cover | NA | Yes | Yes | Yes | Yes | Yes | Yes | Yes | Yes | Yes | Yes | Yes | Yes | Yes | Yes | Yes | Yes |
| 585 | state and potential management to improve water quality in an agricultural catchment relative to a natural baseline | NA | No | No | Yes | No | Yes | No | No | No | No | No | No | No | No | No | No | No |
| 586 | statewide empirical modeling of bacterial contamination of surface waters | NA | Yes | Yes | Yes | Yes | Yes | Yes | Yes | Yes | Yes | Yes | Yes | Yes | Yes | Yes | Yes | Yes |
| 588 | statistical assessment of nonpoint source pollution in agricultural watersheds in the lower grand river watershed, mo, usa | NA | Yes | No | Yes | Yes | Yes | Yes | Yes | No | Yes | Yes | Yes | Yes | Yes | No | Yes | Yes |
| 589 | statistical investigations into indicator bacteria concentrations in houston metropolitan watersheds | NA | No | No | No | No | No | No | No | No | No | No | No | No | No | No | No | No |
| 590 | status and trends of fecal indicator bacteria in two urban watersheds | NA | Yes | Yes | No | Yes | Yes | Yes | Yes | No | Yes | No | Yes | Yes | Yes | Yes | Yes | Yes |
| 591 | storm water events in a small agricultural watershed: characterization and evaluation of improvements in stream water microbiology following implementation of best management practices | NA | No | No | No | No | No | No | No | No | No | No | No | No | No | No | No | No |
| 592 | stormflow dynamics and loads of escherichia coli in a large mixed land use catchment | NA | No | No | No | No | No | No | No | No | No | No | No | No | No | No | No | No |
| 593 | straight pipes and household wastewater discharges into the rural alabama and impact on watershed water quality with wetland land-uses | NA | No | No | No | No | No | No | No | No | No | No | No | No | No | No | No | No |
| 594 | stream water quality changes following timber harvest in a coastal plain swamp forest | NA | No | No | No | No | No | No | No | No | No | No | No | No | No | No | No | No |
| 597 | study of the impact of land use and hydrogeological settings on the shallow groundwater quality in a peri-urban area of kampala, uganda | NA | No | No | No | No | No | No | Yes | No | No | No | Yes | No | No | No | No | Yes |
| 603 | surface water quality along the central john muir trail in the sierra nevada mountains: coliforms and algae | NA | Yes | Yes | Yes | Yes | Yes | Yes | Yes | Yes | Yes | Yes | Yes | Yes | No | Yes | Yes | Yes |
| 604 | surface water quality and landscape gradients in the north carolina cape fear river basin: the key role of fecal coliform | NA | Yes | Yes | Yes | Yes | Yes | Yes | Yes | Yes | Yes | Yes | Yes | Yes | Yes | Yes | Yes | Yes |
| 605 | surface water quality in rural communities in the state of goi?s during the dry season and its relationship with land use and occupation | NA | No | No | No | Yes | No | No | No | Yes | No | No | No | No | No | No | No | No |
| 606 | surface water quality in rural communities in the state of goias during the dry season and its relationship with land use and occupation | NA | No | No | No | No | No | No | No | No | No | No | No | No | No | No | No | No |
| 609 | temporal stability of e. coli and enterococci concentrations in a pennsylvania creek | No | No | No | No | No | No | No | No | No | No | No | No | No | No | No | No | No |
| 610 | the 'black waters' of malaysia: tracking water quality from the peat swamp forest to the sea | No | No | No | No | No | No | No | No | No | No | No | No | No | No | No | No | No |
| 611 | the changing face of water: a dynamic reflection of antibiotic resistance across landscapes | No | Yes | Yes | Yes | Yes | Yes | Yes | Yes | Yes | Yes | Yes | Yes | Yes | Yes | Yes | Yes | Yes |
| 612 | the chao phraya river basin: water quality and anthropogenic influences | NA | Yes | Yes | Yes | Yes | Yes | Yes | Yes | Yes | Yes | Yes | Yes | Yes | Yes | Yes | Yes | Yes |
| 613 | the effect of anthropogenic pressure shown by microbiological and chemical water quality indicators on the main rivers of podhale, southern poland | NA | Yes | Yes | Yes | Yes | Yes | Yes | Yes | Yes | Yes | Yes | Yes | Yes | Yes | Yes | Yes | Yes |
| 615 | the effects of spatial variability of land use on stream water quality in a costal watershed | NA | Yes | Yes | Yes | Yes | Yes | Yes | Yes | Yes | Yes | Yes | Yes | Yes | Yes | Yes | Yes | Yes |
| 618 | the impact of cattle farming best management practices on surface water nutrient concentrations, faecal bacteria and algal dominance in the lake oconee watershed | NA | No | No | No | No | No | No | No | No | No | No | No | No | No | No | No | No |
| 619 | the impact of various land uses on the microbial and physicochemical quality of surface water bodies in developing countries: prioritisation of water resources management areas | NA | Yes | Yes | Yes | Yes | Yes | No | Yes | Yes | Yes | No | Yes | Yes | Yes | Yes | Yes | Yes |
| 620 | the inca-pathogens model: an application to the loimijoki river basin in finland | NA | No | No | No | No | No | No | No | No | No | No | No | No | No | No | No | No |
| 621 | the influence of land-use composition on fecal contamination of riverine source water in southern british columbia | NA | Yes | Yes | Yes | Yes | Yes | Yes | Yes | Yes | Yes | Yes | Yes | Yes | Yes | Yes | Yes | Yes |
| 622 | the influence of rainfall on the incidence of microbial faecal indicators and the dominant sources of faecal pollution in a florida river | NA | No | No | No | No | No | No | No | No | No | No | No | No | No | No | No | No |
| 627 | the need for proper management leading to the sustainability of the kelani river and its lower basin | NA | No | No | No | No | No | No | No | No | No | No | No | No | No | No | No | No |
| 628 | the relationship of land use practices to surface water quality in the upper oconee watershed of georgia | NA | No | No | No | No | No | No | No | No | No | Yes | No | No | No | No | No | No |
| 629 | the role of land use and environmental factors on microbial pollution of mountainous limestone aquifers | NA | No | No | No | No | No | No | No | No | No | No | No | No | No | No | No | No |
| 636 | tracking sources and dissemination of indicator antibiotic resistance genes at a watershed scale | NA | No | No | No | Yes | No | No | No | No | No | No | No | No | No | No | No | No |
| 639 | transport and variability of fecal bacteria in carbonate conglomerate aquifers | NA | No | No | No | No | No | No | No | No | No | No | No | No | No | No | No | No |
| 640 | trend analysis of water quality in some rivers with different degress of development within the sao paulo state, brazil | NA | No | No | No | No | No | No | No | No | No | No | No | No | No | No | No | No |
| 641 | trends in water quality of five dairy farming streams in response to adoption of best practice and benefits of long-term monitoring at the catchment scale | NA | No | No | No | No | No | No | No | No | No | No | No | No | No | No | No | No |
| 642 | turbidity as an indicator of water quality in diverse watersheds of the upper pecos river basin | No | No | No | No | No | No | No | No | No | No | No | No | No | No | No | No | No |
| 646 | understanding the spatiotemporal pollution dynamics of highly fragile montane watersheds of kashmir himalaya, india | No | No | No | No | No | No | No | No | No | No | No | Yes | No | No | No | Yes | Yes |
| 651 | urban influences on stream chemistry and biology in the big brushy creek watershed, south carolina | NA | No | No | No | No | No | No | No | No | No | No | No | No | No | No | No | No |
| 653 | urban pollution of bagmati river corridor within the densely populated kathmandu valley in nepal | NA | No | Yes | No | No | No | Yes | Yes | No | Yes | No | Yes | No | No | No | Yes | No |
| 655 | using a weight-of-evidence approach for management of watersheds | NA | Yes | No | Yes | Yes | Yes | No | Yes | Yes | No | Yes | Yes | No | Yes | No | Yes | Yes |
| 658 | using microbiological tracers to assess the impact of winter land use restrictions on the quality of stream headwaters in a small catchment | NA | No | No | No | No | No | No | No | No | No | No | No | No | No | No | No | No |
| 659 | using multi-threshold regression techniques to assess river fecal pollution in the highly urbanized tamsui river watershed | NA | Yes | Yes | Yes | Yes | Yes | Yes | Yes | Yes | Yes | Yes | Yes | Yes | Yes | Yes | Yes | Yes |
| 660 | using radical terraces for erosion control and water quality improvement in rwanda: a case study in sebeya catchment | NA | No | No | No | No | No | No | No | No | No | No | No | No | No | No | No | No |
| 661 | using remote sensing to identify changes in land use and sources of fecal bacteria to support a watershed transport model | NA | No | No | No | No | No | No | No | No | No | No | No | No | No | No | No | No |
| 662 | using spatial-stream-network models and long-term data to understand and predict dynamics of faecal contamination in a mixed land-use catchment | NA | Yes | No | No | Yes | No | Yes | No | Yes | No | No | Yes | Yes | Yes | Yes | Yes | No |
| 663 | using watershed characteristics to enhance fecal source identification | NA | Yes | Yes | No | Yes | Yes | Yes | Yes | Yes | Yes | No | Yes | Yes | Yes | Yes | Yes | Yes |
| 665 | validating microbial source tracking markers and assessing the efficacy of culturable e. coli and enterococcus assays in ozark streams, usa | Yes | Yes | Yes | Yes | Yes | Yes | Yes | Yes | Yes | Yes | Yes | Yes | Yes | Yes | Yes | Yes | Yes |
| 666 | variability of e. coli density and sources in an urban watershed | No | No | No | No | No | No | No | No | No | No | No | No | No | No | No | No | No |
| 667 | variability of escherichia coli concentrations in an urban watershed in texas | NA | No | No | No | No | No | No | No | No | No | No | No | No | No | No | No | No |
| 668 | variability of indicator bacteria at different time scales in the upper hoosic river watershed | NA | No | Yes | Yes | No | No | Yes | No | No | Yes | No | No | No | Yes | No | Yes | Yes |
| 669 | verifying the applicability of swat to simulate fecal contamination for watershed management of selangor river, malaysia | NA | No | No | No | No | No | No | No | No | No | No | No | No | No | No | No | No |
| 670 | vertical flow constructed wetlands as green facades and gardens for on-site greywater treatment in buildings: two-year mesocosm study on removal performance | NA | No | No | No | No | No | No | No | No | No | No | No | No | No | No | No | No |
| 673 | water and sediment microbial quality of mountain and agricultural streams | Yes | Yes | No | Yes | Yes | Yes | Yes | Yes | Yes | Yes | No | Yes | No | Yes | Yes | Yes | Yes |
| 674 | water pollution and water quality assessment of the way kuripan river in bandar lampung city (sumatera, indonesia) | No | No | No | No | No | No | No | No | No | No | No | No | No | No | No | No | No |
| 675 | water quality and restoration in a coastal subdivision stormwater pond | NA | No | No | No | No | No | No | No | No | No | No | No | No | No | No | No | No |
| 677 | water quality and thermal regime of the motueka river: influences of land cover, geology and position in the catchment | NA | Yes | Yes | Yes | No | No | Yes | Yes | Yes | No | Yes | Yes | No | No | No | Yes | Yes |
| 680 | water quality assessment and the influence of landscape metrics at multiple scales in poyang lake basin | NA | No | No | No | No | No | No | No | No | No | No | Yes | No | No | No | Yes | No |
| 682 | water quality assessment with emphasis in parameter optimisation using pattern recognition methods and genetic algorithm | NA | No | No | No | No | No | No | No | No | No | No | No | No | No | No | No | No |
| 683 | water quality changes in a polluted stream over a twenty-five-year period | NA | No | No | No | No | No | No | No | No | No | No | No | No | No | No | No | No |
| 684 | water quality evaluation of two urban streams in northwest uruguay: are national regulations for urban stream quality sufficient? | NA | No | No | No | No | No | No | No | No | No | No | No | No | No | No | No | No |
| 687 | water quality in low-elevation streams and rivers of new zealand: recent state and trends in contrasting land-cover classes | NA | Yes | Yes | Yes | Yes | Yes | Yes | Yes | Yes | No | Yes | Yes | Yes | Yes | No | Yes | Yes |
| 688 | water quality in microbasins and springs of the mineral water region of minas gerais state, brazil | NA | No | No | No | No | No | No | No | No | No | No | No | No | No | No | No | No |
| 689 | water quality in new zealand rivers: current state and trends | NA | Yes | Yes | Yes | Yes | Yes | Yes | Yes | Yes | Yes | Yes | Yes | Yes | Yes | Yes | Yes | Yes |
| 690 | water quality in part of the submiddle san francisco region due to seasonality and land use and occupation | NA | No | No | No | No | No | No | No | No | No | No | No | No | No | No | No | No |
| 693 | water quality in various land cover type in nanggala sub watershed | NA | No | No | No | No | No | No | No | No | No | No | No | No | No | No | No | No |
| 694 | water quality in watershed of the jaboatao river (pernambuco, brazil): a case study | NA | No | No | No | No | No | No | No | No | No | No | No | No | No | No | No | No |
| 696 | water quality index in two land use situations in the mantiqueira range | NA | No | No | No | No | No | No | No | No | No | No | No | No | No | No | No | No |
| 698 | water quality indicators in the mantiqueira range region, minas gerais state | NA | Yes | Yes | Yes | Yes | Yes | Yes | Yes | Yes | Yes | No | Yes | Yes | Yes | Yes | Yes | No |
| 699 | water quality modification by land use types in watershed ecosystems of southwestern nigeria | NA | No | No | No | No | Yes | No | No | No | No | No | No | No | Yes | No | No | No |
| 703 | watershed land use, surface water vulnerability and public health risks of two urban rivers, ado-ekiti, south-west nigeria | NA | No | No | No | No | No | No | No | No | No | Yes | No | No | No | No | No | No |
| 707 | whole catchment land cover effects on water quality in the lower kaskaskia river watershed | NA | No | No | No | Yes | No | No | No | No | No | Yes | Yes | Yes | No | Yes | No | No |
| 709 | wildlife identified as major source of escherichia coli in agriculturally dominated watersheds by box a1r-derived genetic fingerprints | NA | No | No | No | No | No | No | No | No | No | No | No | No | No | No | No | No |
| 710 | year-long metagenomic study of river microbiomes across land use and water quality | No | No | No | No | No | No | No | No | No | No | No | No | No | No | No | No | No |
