# Supplementary material for: AI-assisted evidence screening method for systematic reviews in environmental research: integrating ChatGPT with domain knowledge
Source: Environ Evid. 2025 Apr 15;14:5. doi: 10.1186/s13750-025-00358-5 (PMC11998256; doi:10.1186/s13750-025-00358-5)
Supplement: Supplementary file 10 — Supplementary Material 10 [file 13750_2025_358_MOESM10_ESM.docx]

**Table A11.** The ChatGPT-3.5 Turbo performance across Temperature and Top_p parameter settings in Step 1

| **Temp** | **Top_p** | **TP** | **TN** | **FP** | **FN** | **Sensitivity** | **Specificity** | **Accuracy** | **Kappa** |
| --- | --- | --- | --- | --- | --- | --- | --- | --- | --- |
| 0.2 | 0.2 | 22 | 12 | 5 | 1 | 0.96 | 0.71 | 0.85 | 0.68 |
| 0.2 | 0.4 | 22 | 12 | 5 | 1 | 0.96 | 0.71 | 0.85 | 0.68 |
| 0.2 | 0.6 | 22 | 11 | 6 | 1 | 0.96 | 0.65 | 0.83 | 0.63 |
| 0.2 | 0.8 | 22 | 12 | 5 | 1 | 0.96 | 0.71 | 0.85 | 0.68 |
| 0.4 | 0.2 | 22 | 12 | 5 | 1 | 0.96 | 0.71 | 0.85 | 0.68 |
| 0.4 | 0.4 | 22 | 11 | 6 | 1 | 0.96 | 0.65 | 0.83 | 0.63 |
| 0.4 | 0.6 | 22 | 13 | 4 | 1 | 0.96 | 0.76 | 0.88 | 0.74 |
| 0.4 | 0.8 | 22 | 14 | 3 | 1 | 0.96 | 0.82 | 0.9 | 0.79 |
| 0.6 | 0.2 | 22 | 12 | 5 | 1 | 0.96 | 0.71 | 0.85 | 0.68 |
| 0.6 | 0.4 | 22 | 11 | 6 | 1 | 0.96 | 0.65 | 0.83 | 0.63 |
| 0.6 | 0.6 | 22 | 12 | 5 | 1 | 0.96 | 0.71 | 0.85 | 0.68 |
| 0.6 | 0.8 | 22 | 12 | 5 | 1 | 0.96 | 0.71 | 0.85 | 0.68 |
| 0.8 | 0.2 | 22 | 12 | 5 | 1 | 0.96 | 0.71 | 0.85 | 0.68 |
| 0.8 | 0.4 | 22 | 13 | 4 | 1 | 0.96 | 0.76 | 0.88 | 0.74 |
| 0.8 | 0.6 | 22 | 11 | 6 | 1 | 0.96 | 0.65 | 0.83 | 0.63 |
| 0.8 | 0.8 | 23 | 24 | 3 | 0 | 1 | 0.82 | 0.83 | 0.84 |

**Table A12.** The ChatGPT-3.5 Turbo performance across Temperature and Top_p parameter settings in Step 2

| **Temp** | **Top_p** | **TP** | **TN** | **FP** | **FN** | **Sensitivity** | **Specificity** | **Accuracy** | **Kappa** |
| --- | --- | --- | --- | --- | --- | --- | --- | --- | --- |
| 0.2 | 0.2 | 11 | 26 | 1 | 7 | 0.61 | 0.96 | 0.82 | 0.61 |
| 0.2 | 0.4 | 12 | 26 | 1 | 6 | 0.67 | 0.96 | 0.84 | 0.66 |
| 0.2 | 0.6 | 12 | 26 | 1 | 6 | 0.67 | 0.96 | 0.84 | 0.66 |
| 0.2 | 0.8 | 11 | 26 | 1 | 7 | 0.61 | 0.96 | 0.82 | 0.61 |
| 0.4 | 0.2 | 12 | 26 | 1 | 6 | 0.67 | 0.96 | 0.84 | 0.66 |
| 0.4 | 0.4 | 11 | 25 | 2 | 7 | 0.61 | 0.93 | 0.8 | 0.56 |
| 0.4 | 0.6 | 12 | 26 | 1 | 6 | 0.67 | 0.96 | 0.84 | 0.66 |
| 0.4 | 0.8 | 11 | 26 | 1 | 7 | 0.61 | 0.96 | 0.82 | 0.61 |
| 0.6 | 0.2 | 12 | 26 | 1 | 6 | 0.67 | 0.96 | 0.84 | 0.66 |
| 0.6 | 0.4 | 12 | 25 | 2 | 6 | 0.67 | 0.93 | 0.82 | 0.62 |
| 0.6 | 0.6 | 12 | 25 | 2 | 6 | 0.67 | 0.93 | 0.82 | 0.62 |
| 0.6 | 0.8 | 11 | 26 | 1 | 7 | 0.61 | 0.96 | 0.82 | 0.61 |
| 0.8 | 0.2 | 12 | 26 | 1 | 6 | 0.67 | 0.96 | 0.84 | 0.66 |
| 0.8 | 0.4 | 10 | 25 | 2 | 8 | 0.56 | 0.93 | 0.78 | 0.51 |
| 0.8 | 0.6 | 10 | 25 | 2 | 8 | 0.56 | 0.93 | 0.78 | 0.51 |
| 0.8 | 0.8 | 9 | 24 | 3 | 9 | 0.50 | 0.89 | 0.73 | 0.41 |
