# Supplementary material for: AI-assisted evidence screening method for systematic reviews in environmental research: integrating ChatGPT with domain knowledge
Source: Environ Evid. 2025 Apr 15;14:5. doi: 10.1186/s13750-025-00358-5 (PMC11998256; doi:10.1186/s13750-025-00358-5)
Supplement: Supplementary file 11 — Supplementary Material 11 [file 13750_2025_358_MOESM11_ESM.docx]

**Table A13.** The Cohen’s Kappa score of ChatGPT-3.5 Turbo in 15 runs at Step 1 and 2

| **Runs** | **Cohen’s Kappa Score of Step 1** | **Cohen’s Kappa Score of Step 2** |
| --- | --- | --- |
| 1 | 0.634 (p < 0.05) | 0.661 (p < 0.05) |
| 2 | 0.529 (p < 0.05) | 0.554 (p < 0.05) |
| 3 | 0.843 (p < 0.05) | 0.466 (p < 0.05) |
| 4 | 0.578 (p < 0.05) | 0.510 (p < 0.05) |
| 5 | 0.738 (p < 0.05) | 0.661 (p < 0.05) |
| 6 | 0.683 (p < 0.05) | 0.615 (p < 0.05) |
| 7 | 0.688 (p < 0.05) | 0.661 (p < 0.05) |
| 8 | 0.738 (p < 0.05) | 0.455 (p < 0.05) |
| 9 | 0.734 (p < 0.05) | 0.571 (p < 0.05) |
| 10 | 0.738 (p < 0.05) | 0.608 (p < 0.05) |
| 11 | 0.734 (p < 0.05) | 0.519 (p < 0.05) |
| 12 | 0.578 (p < 0.05) | 0.563 (p < 0.05) |
| 13 | 0.584 (p < 0.05) | 0.661 (p < 0.05) |
| 14 | 0.683 (p < 0.05) | 0.412 (p < 0.05) |
| 15 | 0.584 (p < 0.05) | 0.608 (p < 0.05) |
