# Supplementary material for: AI-assisted evidence screening method for systematic reviews in environmental research: integrating ChatGPT with domain knowledge
Source: Environ Evid. 2025 Apr 15;14:5. doi: 10.1186/s13750-025-00358-5 (PMC11998256; doi:10.1186/s13750-025-00358-5)
Supplement: Supplementary file 12 — Supplementary Material 12 [file 13750_2025_358_MOESM12_ESM.docx]

**Table A14.** The Cohen’s Kappa score of reviewers in different rounds at Step 1 and 2

| **Rounds** | **Cohen’s Kappa Score of R1 in Step 1** | **Cohen’s Kappa Score of R1 in Step 2** | **Cohen’s Kappa Score of R2 in Step 1** | **Cohen’s Kappa Score of R2 in Step 2** | **Cohen’s Kappa Score of R3 in Step 1** | **Cohen’s Kappa Score of R3 in Step 2** |
| --- | --- | --- | --- | --- | --- | --- |
| 1 | 0.222 (p < 0.05) | 0.595 (p < 0.05) | 1.000 (p < 0.05) | 0.595 (p < 0.05) | 0.545 (p < 0.05) | 0.737 (p < 0.05) |
| 2 | 0.658 (p < 0.05) | 0.348 (p < 0.05) | 0.800 (p < 0.05) | 0.737 (p < 0.05) | 0.186 (p < 0.05) | 0.464 (p < 0.05) |
| 3 | 0.646 (p < 0.05) | 0.842 (p < 0.05) | 0.702 (p < 0.05) | 0.842 (p < 0.05) | 0.648 (p < 0.05) | 0.842 (p < 0.05) |
| 4 | 0.658 (p < 0.05) | NA | 0.795 (p < 0.05) | NA | 0.585 (p < 0.05) | NA |
