# Supplementary material for: AI-assisted evidence screening method for systematic reviews in environmental research: integrating ChatGPT with domain knowledge
Source: Environ Evid. 2025 Apr 15;14:5. doi: 10.1186/s13750-025-00358-5 (PMC11998256; doi:10.1186/s13750-025-00358-5)
Supplement: Supplementary file 13 — Supplementary Material 13 [file 13750_2025_358_MOESM13_ESM.docx]

**Table A15.** The agreement of rounds between three reviewers in Step 1 and 2

| **Rounds** | **Fleiss’s Kappa score for Step 1** | **Fleiss’s Kappa score for Step 2** |
| --- | --- | --- |
| 1 | 0.358 (p < 0.05) | 0.555 (p < 0.05) |
| 2 | 0.352 (p < 0.05) | 0.271 (p < 0.05) |
| 3 | 0.432 (p < 0.05) | 0.800 (p < 0.05) |
| 4 | 0.439 (p < 0.05) | NA |
