# Supplementary material for: AI-assisted evidence screening method for systematic reviews in environmental research: integrating ChatGPT with domain knowledge
Source: Environ Evid. 2025 Apr 15;14:5. doi: 10.1186/s13750-025-00358-5 (PMC11998256; doi:10.1186/s13750-025-00358-5)
Supplement: Supplementary file 14 — Supplementary Material 14 [file 13750_2025_358_MOESM14_ESM.docx]

**Table A16.** The agreement of rounds between reviewer-pair in Step 1 and 2

| **Reviewer Piar** | **Steps** | **Rounds** | **Cohen’s Kappa** | **P-value** |
| --- | --- | --- | --- | --- |
| R-C | 1 | 1st | 0.222 | 0.886 |
| R-C | 1 | 2nd | 0.733 | <0.05 |
| R-C | 1 | 3rd | 0.369 | 0.211 |
| R-C | 1 | 4th | 0.583 | <0.05 |
| R-C | 2 | 1st | 0.464 | 0.100 |
| R-C | 2 | 2nd | 0.286 | 0.403 |
| R-C | 2 | 3rd | 0.700 | 0.080 |
| R-H | 1 | 1st | 0.545 | <0.05 |
| R-H | 1 | 2nd | 0.267 | 1.000 |
| R-H | 1 | 3rd | 0.557 | <0.05 |
| R-H | 1 | 4th | 0.366 | 0.289 |
| R-H | 2 | 1st | 0.602 | <0.05 |
| R-H | 2 | 2nd | 0.683 | 0.610 |
| R-H | 2 | 3rd | 1.000 | <0.05 |
| C-H | 1 | 1st | 0.308 | 0.176 |
| C-H | 1 | 2nd | 0.211 | 1.000 |
| C-H | 1 | 3rd | 0.388 | <0.05 |
| C-H | 1 | 4th | 0.366 | 0.289 |
| C-H | 2 | 1st | 0.602 | <0.05 |
| C-H | 2 | 2nd | 0.348 | 0.618 |
| C-H | 2 | 3rd | 0.700 | 0.080 |
| R-H | 2 | 3rd | 1.000 | <0.05 |
