# Supplementary material for: AI-assisted evidence screening method for systematic reviews in environmental research: integrating ChatGPT with domain knowledge
Source: Environ Evid. 2025 Apr 15;14:5. doi: 10.1186/s13750-025-00358-5 (PMC11998256; doi:10.1186/s13750-025-00358-5)
Supplement: Supplementary file 15 — Supplementary Material 15 [file 13750_2025_358_MOESM15_ESM.docx]

**Table A6.** The reviewers and ChatGPT screening result of test set 40-articles in Step 1

| **Unique ID** | **Title** | **Reviewers Consensus Decision** | **R1** | **R2** | **R3** | **Rounds** | **GPT Majority Answer** | **1** | **2** | **3** | **4** | **5** | **6** | **7** | **8** | **9** | **10** | **11** | **12** | **13** | **14** | **15** |
| --- | --- | --- | --- | --- | --- | --- | --- | --- | --- | --- | --- | --- | --- | --- | --- | --- | --- | --- | --- | --- | --- | --- |
| 463 | occurrence and distribution of viruses and picoplankton in tropical freshwater bodies determined by flow cytometry | No | No | No | Yes | 2 | No | No | Yes | No | Yes | No | No | No | No | No | No | Yes | No | No | Yes | Yes |
| 468 | particle-attached riverine bacteriome shifts in a pollutant-resistant and pathogenic community during a mediterranean extreme storm event | No | No | No | No | 2 | No | No | No | No | No | No | No | No | No | No | No | No | No | No | No | No |
| 469 | pathogen transport and fate modeling in the upper salem river watershed using swat model | No | No | No | No | 3 | Yes | Yes | Yes | Yes | Yes | Yes | Yes | Yes | Yes | Yes | Yes | Yes | Yes | Yes | Yes | Yes |
| 470 | patterns and drivers of fecal coliform exports in a typhoon-affected watershed: insights from 10-year observations and swat model | Yes | Yes | Yes | Yes | 4 | Yes | Yes | Yes | Yes | Yes | Yes | Yes | Yes | Yes | Yes | Yes | Yes | Yes | Yes | Yes | Yes |
| 499 | prevalence of antibiotic resistance in the tropical rivers of sri lanka and india | No | Yes | No | No | 3 | No | Yes | No | No | Yes | No | Yes | No | No | Yes | Yes | No | No | Yes | No | Yes |
| 502 | quantification of fecal coliform inputs to aquatic systems through soil leaching | Yes | No | Yes | No | 1 | Yes | Yes | Yes | Yes | Yes | Yes | Yes | Yes | Yes | Yes | Yes | Yes | Yes | Yes | Yes | Yes |
| 503 | quantification of human-associated fecal indicators reveal sewage from urban watersheds as a source of pollution to lake michigan | No | No | No | No | 2 | No | No | Yes | No | Yes | No | No | Yes | No | No | No | No | Yes | Yes | No | No |
| 504 | quantification of microbial source tracking and pathogenic bacterial markers in water and sediments of tiaoxi river (taihu watershed) | Yes | Yes | Yes | Yes | 3 | Yes | Yes | No | Yes | Yes | Yes | Yes | Yes | Yes | Yes | Yes | Yes | Yes | Yes | Yes | Yes |
| 506 | quantifying escherichia coli and suspended particulate matter concentrations in a mixed-land use appalachian watershed | Yes | Yes | Yes | No | 4 | Yes | Yes | Yes | Yes | Yes | Yes | Yes | Yes | Yes | Yes | Yes | Yes | Yes | Yes | Yes | Yes |
| 511 | quantitative pcr-based detection of pathogenic leptospira in hawai'ian coastal streams | No | No | No | No | 3 | No | No | No | No | No | No | No | No | No | No | No | No | No | No | No | No |
| 512 | rainfall driven e. coli transfer to the stream conduit network observed through increasing spatial scales in mixed land use paddy farming karst terrain | Yes | No | Yes | Yes | 1 | Yes | Yes | Yes | Yes | Yes | Yes | Yes | Yes | Yes | Yes | Yes | Yes | Yes | Yes | Yes | Yes |
| 513 | reach specificity in sediment e. coli population turnover and interaction with waterborne populations | Yes | Yes | Yes | Yes | 2 | Yes | Yes | Yes | Yes | Yes | Yes | Yes | Yes | Yes | Yes | Yes | Yes | No | Yes | Yes | Yes |
| 515 | real-time consequences of riparian cattle trampling for mobilization of sediment, nutrients and bacteria in a british lowland river | No | No | No | No | 3 | Yes | Yes | Yes | Yes | Yes | Yes | Yes | Yes | Yes | Yes | Yes | Yes | Yes | Yes | Yes | Yes |
| 516 | recreational disturbance of river sediments during base flow deteriorates microbial water quality | Yes | No | Yes | Yes | 4 | No | No | No | Yes | No | No | No | No | No | Yes | No | Yes | Yes | No | No | No |
| 533 | role of free-ranging mammals in the deposition of escherichia coli into a texas floodplain | No | No | No | No | 3 | No | No | No | No | No | No | No | No | No | No | No | No | No | No | No | No |
| 534 | sanitary analyses of runoff water a river | Yes | Yes | Yes | No | 1 | Yes | Yes | Yes | Yes | Yes | Yes | Yes | Yes | Yes | Yes | Yes | Yes | Yes | Yes | Yes | Yes |
| 535 | scale of analysis drives the observed ratio of spatial to non-spatial variance in microbial water quality: insights from two decades of citizen science data | No | Yes | Yes | Yes | 2 | Yes | Yes | Yes | No | Yes | Yes | Yes | No | Yes | Yes | Yes | Yes | Yes | Yes | Yes | Yes |
| 536 | scale-dependence of land use effects on water quality of streams in agricultural catchments | Yes | Yes | Yes | Yes | 3 | Yes | Yes | Yes | Yes | Yes | Yes | Yes | Yes | Yes | Yes | Yes | Yes | Yes | Yes | Yes | Yes |
| 537 | scenario-based hydrological modeling for designing climate-resilient coastalwater resource management measures: lessons from brahmani river, odisha, eastern india | No | No | No | No | 4 | No | No | Yes | No | No | No | Yes | Yes | No | No | No | No | Yes | No | Yes | No |
| 607 | sustenance of himalayan springs in an emerging water crisis | No | No | No | No | 3 | No | No | No | No | No | No | No | No | No | No | No | No | No | No | No | No |
| 609 | temporal stability of e. coli and enterococci concentrations in a pennsylvania creek | Yes | Yes | Yes | Yes | 1 | Yes | Yes | Yes | Yes | Yes | Yes | Yes | Yes | Yes | Yes | Yes | Yes | Yes | No | Yes | Yes |
| 610 | the 'black waters' of malaysia: tracking water quality from the peat swamp forest to the sea | Yes | Yes | Yes | Yes | 2 | Yes | Yes | Yes | Yes | Yes | Yes | Yes | Yes | Yes | Yes | Yes | Yes | Yes | Yes | Yes | Yes |
| 611 | the changing face of water: a dynamic reflection of antibiotic resistance across landscapes | Yes | No | Yes | Yes | 3 | Yes | Yes | Yes | Yes | Yes | Yes | Yes | Yes | Yes | Yes | Yes | Yes | Yes | Yes | Yes | Yes |
| 612 | the chao phraya river basin: water quality and anthropogenic influences | Yes | Yes | Yes | Yes | 4 | Yes | Yes | Yes | Yes | Yes | Yes | Yes | Yes | Yes | Yes | Yes | Yes | Yes | Yes | Yes | Yes |
| 642 | turbidity as an indicator of water quality in diverse watersheds of the upper pecos river basin | Yes | Yes | Yes | Yes | 3 | Yes | Yes | Yes | Yes | Yes | Yes | Yes | Yes | Yes | Yes | Yes | Yes | Yes | Yes | Yes | Yes |
| 646 | understanding the spatiotemporal pollution dynamics of highly fragile montane watersheds of kashmir himalaya, india | Yes | Yes | Yes | Yes | 1 | Yes | Yes | Yes | Yes | Yes | Yes | Yes | Yes | Yes | Yes | Yes | Yes | Yes | Yes | Yes | Yes |
| 647 | uptake of nutrients and organic c in streams in new york city drinking-water-supply watersheds | No | No | No | Yes | 2 | No | No | No | No | No | No | No | No | No | No | No | No | No | No | No | No |
| 648 | urban diffuse sources of faecal indicators | No | No | No | No | 3 | No | No | No | No | No | No | No | No | No | No | No | No | No | No | No | No |
| 650 | urban growth and water quality in thimphu, bhutan | No | No | No | No | 4 | No | Yes | No | No | No | Yes | No | No | Yes | Yes | No | No | No | No | No | No |
| 664 | utilization of tryptophan-like fluorescence as a proxy for e. coli contamination in a mixed-land-use karst basin | No | No | No | No | 3 | No | No | No | Yes | No | No | No | No | No | No | No | Yes | Yes | No | No | No |
| 665 | validating microbial source tracking markers and assessing the efficacy of culturable e. coli and enterococcus assays in ozark streams, usa | Yes | Yes | Yes | Yes | 1 | Yes | Yes | Yes | Yes | Yes | Yes | Yes | Yes | Yes | Yes | Yes | Yes | Yes | Yes | Yes | Yes |
| 666 | variability of e. coli density and sources in an urban watershed | Yes | Yes | Yes | Yes | 2 | Yes | Yes | Yes | Yes | Yes | Yes | Yes | Yes | Yes | Yes | Yes | Yes | Yes | Yes | Yes | Yes |
| 667 | variability of escherichia coli concentrations in an urban watershed in texas | Yes | Yes | Yes | Yes | 3 | Yes | Yes | Yes | Yes | Yes | Yes | Yes | Yes | Yes | Yes | Yes | Yes | Yes | Yes | Yes | Yes |
| 668 | variability of indicator bacteria at different time scales in the upper hoosic river watershed | Yes | Yes | Yes | Yes | 4 | Yes | Yes | Yes | Yes | Yes | Yes | Yes | Yes | Yes | Yes | Yes | Yes | Yes | Yes | Yes | Yes |
| 671 | vulnerability of himalayan springs to climate change and anthropogenic impact: a review | No | No | No | Yes | 3 | No | No | No | No | No | No | No | No | No | No | No | No | No | No | No | No |
| 672 | wastewater discharge through a stream into a mediterranean ramsar wetland: evaluation and proposal of a nature-based treatment system | No | Yes | No | No | 1 | No | No | No | No | No | No | No | No | No | No | No | No | No | No | No | No |
| 673 | water and sediment microbial quality of mountain and agricultural streams | Yes | Yes | Yes | Yes | 2 | Yes | Yes | Yes | Yes | Yes | Yes | Yes | Yes | Yes | Yes | Yes | Yes | Yes | Yes | Yes | Yes |
| 674 | water pollution and water quality assessment of the way kuripan river in bandar lampung city (sumatera, indonesia) | Yes | Yes | No | Yes | 3 | Yes | Yes | Yes | Yes | Yes | Yes | Yes | Yes | Yes | Yes | Yes | Yes | Yes | Yes | Yes | Yes |
| 675 | water quality and restoration in a coastal subdivision stormwater pond | Yes | No | Yes | No | 4 | Yes | Yes | Yes | Yes | Yes | Yes | Yes | Yes | Yes | Yes | Yes | Yes | No | No | Yes | No |
| 710 | year-long metagenomic study of river microbiomes across land use and water quality | Yes | Yes | Yes | Yes | 1 | Yes | No | No | Yes | No | Yes | Yes | No | Yes | Yes | Yes | Yes | Yes | Yes | Yes | No |
