# Supplementary material for: AI-assisted evidence screening method for systematic reviews in environmental research: integrating ChatGPT with domain knowledge
Source: Environ Evid. 2025 Apr 15;14:5. doi: 10.1186/s13750-025-00358-5 (PMC11998256; doi:10.1186/s13750-025-00358-5)
Supplement: Supplementary file 16 — Supplementary Material 16 [file 13750_2025_358_MOESM16_ESM.docx]

**Table A7.** The criteria’s different versions of three rounds in Step 2

| **Versions** | **Criteria Number** | **Criteria Information** |
| --- | --- | --- |
| 1st Round | Criteria 2.1 | The mention of multiple types of land use or land cover, or direct mention of “land use” or “land cover” in the result. |
|  | Criteria 2.2 | ‘Fecal coliform’, or ‘Fecal coli’, or ‘Fecal coliforms’, or ‘Faecal coliform’, or ‘Faecal coli’, or ‘Faecal coliforms’, or ‘E. coli’, or ‘Escherichia coli’, or ’Enterococcus’, or ‘Enterococci’. needs to be explicitly mentioned in the result. |
|  | Criteria 2.3 | The quantified relationship between land use, land cover, or multiple types of land use or land cover with ‘Fecal coliform’, or ‘Fecal coli’, or ‘Fecal coliforms’, or ‘Faecal coliform’, or ‘Faecal coli’, or ‘Faecal coliforms’, or ‘E. coli’, or ‘Escherichia coli’, or ’Enterococcus’, or ‘Enterococci’. needs to be demonstrated in the results. |
| 2nd Round | Criteria 2.1 | The research results should contain either the term ‘land use’ or the term ‘land cover’. This requirement can be also satisfied if the research results mention more than one types of land use or land cover. |
|  | Criteria 2.2 | The research results should contain exactly one of the terms in the Fecal Coliform Contamination List. |
|  | Criteria 2.3 | The research results should contain the statistical relationship between land use/land cover or the types of land use/land cover and Fecal coliform or one of the terms in the Fecal Coliform Contamination List. |
| 3rd Round | Criteria 2.1 | The research results should contain either the term ‘land use’ or the term ‘land cover’. This requirement can also be satisfied if the research results mention more than one type of land use or land cover. |
|  | Criteria 2.2 | The research results should contain exactly one of the terms in the Fecal Coliform Contamination List. |
|  | Criteria 2.3 | The research results should contain the statistical relationship between land use/land cover or the types of land use/land cover and Fecal coliform or one of the terms in the Fecal Coliform Contamination List. |
